# Communicating population health statistics through graphs: a randomised controlled trial of graph design interventions

**DOI:** 10.1186/1741-7015-4-33

**Published:** 2006-12-20

**Authors:** David J Muscatello, Andrew Searles, Robin Macdonald, Louisa Jorm

**Affiliations:** 1 Centre for Epidemiology and Research, New South Wales Department of Health, 73 Miller Street, North Sydney NSW 2060, Australia; 2 Hunter Valley Research Foundation, 55 Downie Street, Maryville NSW 2293, Australia

## Abstract

**Background:**

Australian epidemiologists have recognised that lay readers have difficulty understanding statistical graphs in reports on population health. This study aimed to provide evidence for graph design improvements that increase comprehension by non-experts.

**Methods:**

This was a double-blind, randomised, controlled trial of graph-design interventions, conducted as a postal survey. Control and intervention participants were randomly selected from telephone directories of health system employees. Eligible participants were on duty at the listed location during the study period. Controls received a booklet of 12 graphs from original publications, and intervention participants received a booklet of the same graphs with design modifications. A questionnaire with 39 interpretation tasks was included with the booklet. Interventions were assessed using the ratio of the prevalence of correct responses given by the intervention group to those given by the control group for each task.

**Results:**

The response rate from 543 eligible participants (261 intervention and 282 control) was 67%. The prevalence of correct answers in the control group ranged from 13% for a task requiring knowledge of an acronym to 97% for a task identifying the largest category in a pie chart. Interventions producing the greatest improvement in comprehension were: changing a pie chart to a bar graph (3.6-fold increase in correct point reading), changing the y axis of a graph so that the upward direction represented an increase (2.9-fold increase in correct judgement of trend direction), a footnote to explain an acronym (2.5-fold increase in knowledge of the acronym), and matching the y axis range of two adjacent graphs (two-fold increase in correct comparison of the relative difference in prevalence between two population subgroups).

**Conclusion:**

Profound population health messages can be lost through use of overly technical language and unfamiliar statistical measures. In our study, most participants did not understand age standardisation and confidence intervals. Inventive approaches are required to address this problem.

## Background

Local, regional, national and global public health authorities publish an ever-increasing number of reports that statistically describe the health of the populations they serve. Graphs form a large component of such reports, because they provide a visual means to summarise relationships between variables that influence health.

The relationship between the design of graphs and the ability of people to comprehend them has been extensively studied in the fields of cognitive psychology, education, ergonomics and statistics. However, little information is available on comprehension of population health statistics.

In 1999, the need to improve methods of communicating epidemiological and statistical concepts to lay audiences was recognised and incorporated into Australia's National Public Health Information Development Plan [1]. This led to a project aimed at assessing the available evidence on graph design and reader comprehension (see Additional file 1) and a study that could provide practical recommendations specific to graphs of population health statistics (see Additional file 2).

This report describes the results of a component of the project aimed at testing specific interventions that were identified as being particularly relevant to the types of graphs appearing in Australian population health publications, but for which strong evidence could not be found in the literature. A secondary aim was to assess whether comprehension of the data and the success of interventions varied by educational attainment.

## Methods

### Study design

This was a double-blind, randomised, controlled trial, with data collected through a self-completed questionnaire. Participants were randomly assigned to receive either a "control" or an "intervention" booklet of graphs. Both groups received an identical questionnaire that explored participants' understanding of the meaning of the graphs.

Study participants were blinded to their control or intervention status. Study personnel and researchers were blinded to the status of respondents until after data analysis occurred. Each respondent group was assigned an arbitrary group identifier that did not reveal their status, even during analysis of the results. Data entry personnel were blinded to the respondent status, as any graph booklets returned with questionnaires were discarded prior to data entry. The status of each group was revealed only after analysis was complete.

### Control and intervention graphs and questionnaire

The "control" booklet contained 12 graphs reproduced from an original Australian population health publication. They covered a range of different graph styles and numerical measures, including population size, disease incidence rates, disease prevalence, incidence rate ratios, and risk of developing disease. Statistical and epidemiological concepts, such as age standardisation and confidence intervals, were included in some graphs.

**Figure 1:**
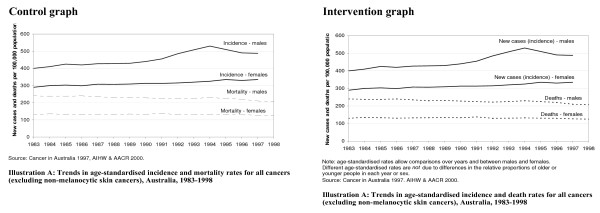
**Control (left) and intervention (right) graphs used to test comprehension after simplifying the series labels and a footnote explaining age standardisation**. Graphs are not shown at their original scale. Control graph reproduced with permission from the Australian Institute of Health and welfare.

**Figure 2:**
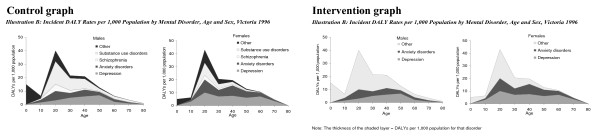
**Control (left) and intervention (right) graphs used to test comprehension after removing a layer from a stacked layer graph and including a footnote explaining how to interpret the thickness of a layer**. Graphs are not shown at their original scale. Control graph reproduced with permission from the Victorian Department of Human Services, Victoria, Australia.

**Figure 3:**
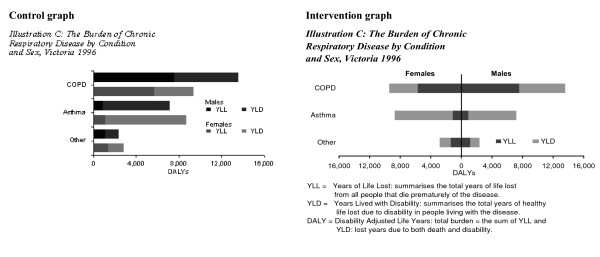
**Control (left) and intervention (right) graphs used to test comprehension after changing a horizontal bar graph to a side-by-side bar graph and including a footnote explaining acronyms used in the graph**. Graphs are not shown at their original scale. Control graph reproduced with permission from the Victorian Department of Human Services, Victoria, Australia.

**Figure 4:**
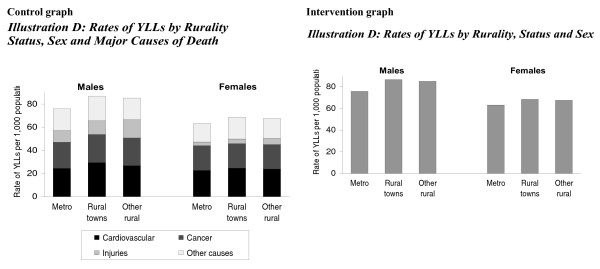
**Control (left) and intervention (right) graphs used to test comprehension after removing an independent variable from a graph**. Graphs are not shown at their original scale. Control graph reproduced with permission from the Victorian Department of Human Services, Victoria, Australia.

**Figure 5:**
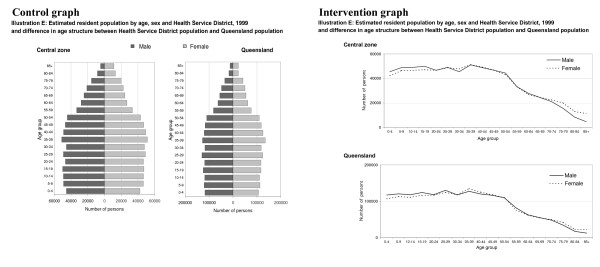
**Control (left) and intervention (right) graphs used to test comprehension after changing a population pyramid to a line graph**. Graphs are not shown at their original scale. Control graph reproduced with permission from the State of Queensland (Queensland Health), Australia.

**Figure 6:**
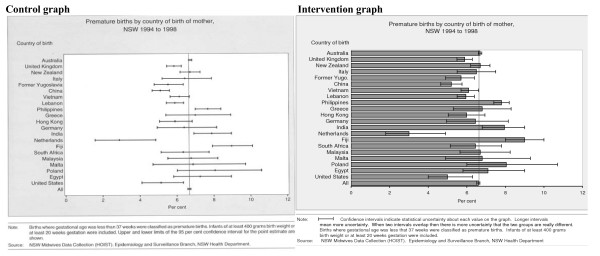
**Control (left) and intervention (right) graphs used to test comprehension after changing a dot graph with confidence intervals ("hi–lo–close" graph) to a bar graph and including a footnote describing how to interpret confidence intervals**. Graphs are not shown at their original scale. Control graph reproduced with permission from the New South Wales Department of Health, Australia.

**Figure 7:**
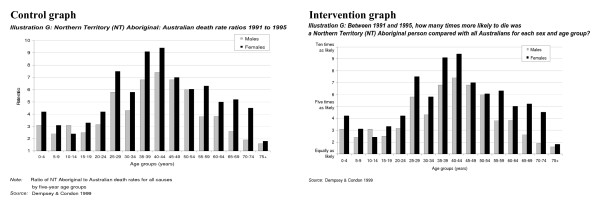
**Control (left) and intervention (right) graphs used to test comprehension after changing the graph title to a plain question that guided the interpretation of the graph, and changing some y axis labels to descriptive text to aid interpretation**. Graphs are not shown at their original scale. The footnote was removed from the intervention graph as it became redundant. Control graph reproduced with permission from the Northern Territory Department of Health and Community Services, Australia.

**Figure 8:**
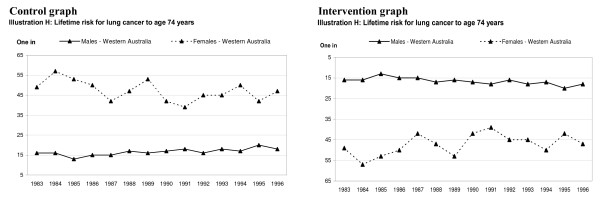
**Control (left) and intervention (right) graphs used to test comprehension after reversing the scale of the vertical axis to represent increasing risk in the upward direction**. Graphs are not shown at their original scale. Control graph reproduced with permission from the Health Department of Western Australia.

**Figure 9:**
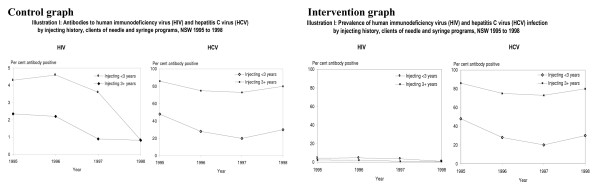
**Control (left) and intervention (right) graphs used to test comprehension after making the y axis range on two adjacent graphs match and simplifying the graph title slightly**. Graphs are not shown at their original scale. Control graph reproduced with permission from the New South Wales Department of Health, Australia.

**Figure 10:**
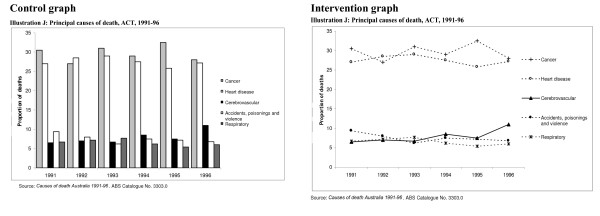
**Control (left) and intervention (right) graphs used to test comprehension after changing a vertical bar graph to a line graph**. Graphs are not shown at their original scale. Control graph reproduced with permission from the Australian Capital Territory Department of Health and Community Care.

**Figure 11:**
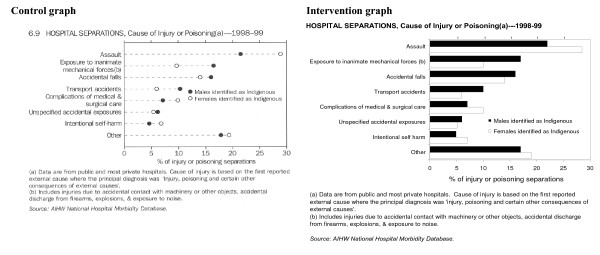
**Control (left) and intervention (right) graphs used to test comprehension after changing a dot graph to a bar graph**. Graphs are not shown at their original scale. Control graph reproduced with permission from the Australian Bureau of Statistics and the Australian Institute of Health and Welfare.

**Figure 12:**
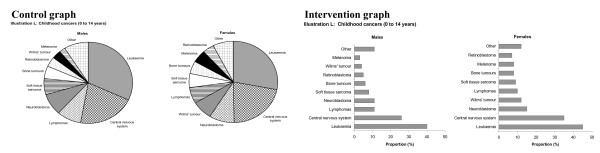
**Control (left) and intervention (right) graphs used to test comprehension after changing a pie chart to a horizontal bar graph**. Graphs are not shown at their original scale. Control graph reproduced with permission from the New South Wales Department of Health, Australia.

Graphs for the intervention booklet presented the same statistical information as those in the control booklet, but were subject to one or more changes. The changes were chosen in an effort to improve comprehension of the statistical information depicted in the graph. To limit the number of graphs and thus respondent workload, more than one change was made to some graphs. In some cases, changes were collectively intended to improve understanding, while in others, they were chosen to target specific aspects of comprehension within that graph. The control and intervention version of each graph is shown in Figures 1, 2, 3, 4, 5, 6, 7, 8, 9, 10, 11, 12.

The questionnaire contained several questions relating to each graph, 39 questions in all. Each question was framed in relation to the population health interpretation of the information presented in the graph rather than to extraction of information in isolation from its population health meaning. Questions were also designed to specifically assess the effect of changes made to the graphs for the intervention booklet. Prior to finalising the questionnaire, it was piloted with a convenience sample of 20 people. All but one returned the pilot survey. Pilot respondents were asked to comment on difficulties they had, and consequent changes were made.

The questionnaire also collected demographic details: education level, preferred language, age group, and sex. Respondents were also asked their work title, how frequently they used graphs, and to rate their visual ability to read the graphs presented.

The control and intervention graph booklets and the questionnaire are available as appendices to the project report (see Additional file 2).

### Study sample

The study population included employees of the public sector health system of the State of New South Wales (NSW), Australia, regardless of the nature of their work. The state health authorities administer the delivery of, and policies for, public hospital and other public health services for the population of NSW. The workforce therefore includes people with a broad range of job types, not just in medical and health fields. Personnel conduct a broad range of activities, including clerical, financial, policy, scientific, information technology, engineering, maintenance, cleaning, and facilities management. Regional public hospital and community health services are administered by regional health authorities that are in turn administered by the NSW Department of Health. The sampling frame included employees whose contact details were listed on one of five telephone directory databases for employees of the main NSW Department of Health administration (1159 employees), an urban regional health authority (9629 employees), a mixed urban/rural health authority (1840 employees), and two rural health authorities (3560 employees). At the time, there were 17 regional health authorities in NSW, of which five were urban, four were semi-rural, and eight were rural.

In total, 650 participants were randomly selected without stratification from the combined databases of 16188 employees, and these were randomly allocated into one of two groups of 325 participants each: the intervention and control groups. Each participant was posted a package containing a cover letter from the NSW Chief Health Officer inviting their participation, a questionnaire booklet, a control or intervention graph booklet, and a reply-paid envelope. Other than the letter from the Chief Health Officer, there was no incentive for participation. Up to six follow-up reminder calls were made to non-responders. These calls also allowed ineligible participants to be identified. Ineligible participants were those who no longer worked for the health service, who were unknown at the available contact address, or who were not on duty for the survey period.

### Analysis

Unanswered questions were treated as incorrectly answered. The comprehension rate was defined as the prevalence of correct answers within a respondent group. We categorised the comprehension rate according to the following scale: 0% to <20%, very low; 20% to <40%, low; 40% to <60%, moderate; 60% to <80%, high; and 80–100%, very high.

The effect of the interventions on each task was assessed by calculating the ratio of the comprehension rate in the intervention and control groups, with a 95% confidence interval (CI). To assess whether comprehension varied by educational attainment, separate analyses were conducted for subgroups of respondents categorised as university-qualified or non-university-qualified. Analysis was conducted using SPSS version 10.

## Results

### Response rate and study sample

Of the 650 participants selected, 543 were eligible, and of these, 187 control and 176 intervention participants returned completed, usable questionnaires, giving an overall response rate of 67% (intervention group 67%, control group 66%).

Sex, age, preferred language, education, and work position were similarly distributed between the control and intervention arms of the study. Intervention participants were somewhat more likely to rate themselves as frequent graph users than were control participants, and more likely to rate themselves as having good visual ability (Table 1).

The maximum proportion of missing answers for any comprehension task was 4% for the control group and 3% for the intervention group.

### Comprehension of the unaltered (control) graphs

In the control group, one of the 39 comprehension tasks had a very low comprehension rate and four tasks had low comprehension rates. Eight tasks had high and 17 had very high comprehension rates (Table 2).

The actual comprehension rates for each task for the 187 controls are shown in Table 3. The task with the very low comprehension rate of 13% required specific knowledge of an acronym (Figure 3). Tasks with a low level of comprehension included judging the direction of a trend in a line graph in which the y axis represented an increasing quantity in the downward direction (21% answered correctly) (Figure 8), estimating a point reading of a quantity from a pie chart (26%) (Figure 12), and those requiring an understanding of confidence intervals (32%) (Figure 6) and age standardisation (37%) (Figure 1).

The tasks with the highest comprehension rates included: choosing the largest (97% comprehension rate) and smallest (91%) categories, and comparing the magnitude of two categories (95%) from a pie chart (Figure 12); determining the largest category from a dot graph (94%) (Figure 11); choosing the category with the lowest value at a single point on the x axis from a vertical bar graph with bars grouped by category (94%) (Figure 10); and broad judgements of the relative magnitude by sex and rurality of bars on a vertical bar graph, grouped by rurality within each sex (93% for sex and 90% for rurality) (Figure 4).

### Effect of interventions

For all respondents, the interventions reduced the number of tasks with a very low comprehension rate from one to zero and those with a low comprehension rate from four to one. The number of tasks with a very high comprehension rate increased from 17 to 28 (Table 2).

Table 3 also shows the ratio of the comprehension rate among intervention participants to that of control participants. The tasks that benefited most from an intervention were:

• Changing a pie chart to a bar graph and point reading the magnitude of a single category (prevalence ratio 3.6; 95% CI 2.8–4.6) (Figure 12). This changed the comprehension rate from low to very high.

• Changing the y axis of a graph so that the upward direction represented an increase rather than a decrease in the plotted quantity when judging the direction of a trend (2.9; 95% CI 2.1–9.9) (Figure 8). This changed the comprehension rate from low to high.

• Including a footnote to explain an acronym and perform a task that requires knowledge of the meaning of the acronym (2.5, 95% CI 1.6–3.8) (Figure 3). This changed the comprehension rate from very low to low.

• Making the y axis range of two adjacent graphs match and comparing the size of a difference between the two series shown on each graph (2.0; 95% CI 1.7–2.4) (Figure 9). This changed the comprehension rate from moderate to very high.

Only one intervention resulted in a reduction in comprehension; describing the pattern of trend in one layer of a stacked-layer graph after removing one layer and adding a footnote for how to interpret a layer (0.8; 95% CI 0.7–0.9) (Figure 2). The comprehension rate decreased from very high to high, thus we speculate that the footnote confused rather than enhanced interpretation.

### Results by educational attainment

Success at comprehending the graphs was lower for the group of 57 control participants without university qualifications than for the group of 124 control participants with university qualifications. Those without a university qualification had a low or very low comprehension rate for 10 of the 39 tasks, compared with 3 tasks for those with a university qualification. Those without a university qualification had a high or very high comprehension rate for 23 tasks compared with 29 for university-qualified participants (Table 2).

Table 3 includes results by educational attainment. The largest differences in comprehension rates among control participants were: judging the statistical significance of the difference between two categories using confidence intervals (very low comprehension among non-university-educated controls versus moderate comprehension among university-educated controls) (Figure 6), understanding the influence of age standardisation on graph interpretation (low versus moderate) (Figure 1), and judging the relative magnitude of risk between two series on a graph when the upward direction on the y axis represents reducing risk (low versus moderate) (Figure 8). An exception was the pie chart, for which controls without a university qualification had a moderate comprehension rate for estimating the magnitude of a category within a pie chart compared with a very low comprehension rate for university-qualified controls (Figure 12). University-qualified participants may have been more likely to assume the task was too difficult and thus not attempt an accurate answer.

For participants without a university qualification, the generally lower success for the control charts was complemented by a generally greater relative benefit from the interventions. For the non-university-qualified participants, high or very high comprehension rates increased from 23 tasks for control participants to 34 tasks for intervention participants, and low or very low comprehension rates decreased from 10 to 2 tasks. For the university-qualified participants, high or very high comprehension rates increased from 29 to 37 tasks, and low or very low comprehension rates decreased from 3 to 1 task (Table 2).

The greatest differences by education level in the effect of interventions were for the dot graph with confidence intervals (a "hi-lo-close" graph), which was changed to a horizontal bar graph with confidence intervals and a footnote was included for interpreting the confidence intervals (Figure 6). The prevalence ratio for correctly interpreting the statistical significance of the difference between two categories on the graph was 2.5 (95% CI 1.3–4.9) for participants without compared with 1.6 (95% CI 1.2–2.0) for participants with a university qualification (Figure 6, Table 3). Nevertheless, this increased the comprehension rate only from very low to low among non-university-qualified participants. For university-qualified participants, the comprehension rate increased from moderate to high. For another task with the same graph requiring a judgement of whether a category was higher or lower than a reference line representing the average of all categories on this graph, the prevalence ratio was 2.3 (95% CI 1.6–3.3) for those without and 1.4 (95% CI 1.2–1.7) for those with a university qualification (Figure 6, Table 3). This had a dramatic improvement for non-university-qualified participants, taking the comprehension rate from low to very high. For university-qualified participants, the comprehension rate increased from moderate to very high. None of the differences in prevalence ratios between the two education groups was statistically significant.

## Discussion

To our knowledge, this is the first randomised, controlled trial assessing interventions to graph design aimed at increasing readers' ability to understand statistical information about population health. In fact, the evidence base for graph comprehension and related cognitive processes in general is largely limited to studies conducted in laboratory settings with small groups of participants, usually university students. We are aware of only one other study that randomly selected participants from a defined population, and it had a response rate of only 50% [2]. Furthermore, we found only a limited number of randomised, controlled study designs in the graph literature [2-4].

Our findings are of benefit from two perspectives. Firstly, we were able to quantify the proportion of readers who could extract some typical statistical interpretations from a sample of graphs used in Australian official population health publications. Depending on the graph and the specific interpretation sought, the proportion of readers able to interpret the graphs correctly ranged from as few as 13% to as many as 97%. Secondly, we were able to quantify the impact on comprehension levels achieved through the simple changes we applied to the graphs. This resulted in a maximum 3–4-fold increase in the proportion of readers who correctly extracted specific information from the graphs.

### Titles and labels

While recommendations have been made about graph titles or captions and labels [5-9], there is little evidence relating to techniques for making their content easily understood.

The most dramatic result of the study related to a vertical bar graph showing that Aboriginal people in a region of Australia had an increased risk of mortality at every age compared with the general population; in some age groups, the increase in mortality was almost 10-fold. More than 40% of control participants (60% of those without university qualifications) were unable to determine from the graph the simple fact that Aboriginal people had a higher risk of death. A combination of interventions that included a title plainly expressing the question that was answered by the graph and the addition of text labels on the vertical axis that directly related to the title, more than halved the proportion of participants who did not grasp this fact.

People working in public health and epidemiology regard the concept of disease incidence as quite commonplace. However, we found that <60% of all participants and less than half of non-university-qualified participants could answer a question that required an understanding that disease incidence refers to the rate of new cases of disease in a period of time. Changing the label on the incidence rate series from "Incidence..." to "New cases (incidence)..." had a statistically significant benefit for both university and non-university-qualified participants.

### Footnotes

To our knowledge, there is no literature on whether graph readers understand statistical concepts used in graphs, despite some recommendations being available [7,9]. Two statistical techniques and concepts occur frequently in population health graphs: age standardisation and confidence intervals. We hypothesised that interpretive tasks requiring an understanding of these concepts would be difficult for people without specialist knowledge. This was confirmed, with the effect of age standardisation being understood by only 23% and 44% of non-university-qualified and university-qualified participants respectively. For a task requiring the interpretation of overlapping confidence limits, the proportions were 16% and 40% respectively. We further hypothesised that a footnote providing a plain, practical explanation of the concepts and their interpretation, could improve the level of understanding, and this was also confirmed, with improvements of up to 2.5-fold in one of the tasks among non-university-qualified participants.

### Volume of information

Reducing information in graphs should improve reader performance [10-12], but by how much? We completely removed an independent (categorisation) variable from a vertical bar graph that originally presented results for a quantity against three independent variables within the one graph. Without the intervention, the graph was reasonably well understood, with the lowest proportion of correct answers being 72% among non-university-qualified participants for a task requiring the estimated total quantity represented by one of the bars. Despite this, the intervention raised the comprehension rate by 20% even for university-educated participants.

### Graph types

We investigated the relative value of line and bar graphs for displaying information that is plotted against a categorical x axis that represents a numerical quantity, such as year or age. A line graph and a grouped bar graph of multiple disease trends by year performed equally well for point-reading tasks, but the line graph produced a marginal improvement in trend judgement in participants without a university qualification. This is as expected; bar graphs encourage discrete rather than trend-based comparisons [13], although bar graphs have been found to be versatile [14,15].

The "population pyramid" is a popular choice for representing the age distribution by sex of a population. It is in fact a vertically oriented side-by-side bar graph. It can, however, also be represented as a horizontal format line graph with two series, each series showing the population size by age for each sex. To a greater extent, surprisingly, in university-educated participants, the line graph improved a broad comparison of the size of the male and female populations over a range of age groups. Interpretation of the broad shape of the population distribution was unaffected by the intervention.

Dot graphs have been proposed as an improvement on bar graphs [16]. We found that a bar graph with 95% confidence intervals clearly out-performed dot graphs with 95% confidence intervals (sometimes called "hi-lo-close" graphs), particularly among those without university qualifications. For another type of dot graph, which had each dot connected by a dashed line to the x axis, but had no confidence intervals, a horizontal bar graph performed equally well, and even showed a marginal improvement for those without a university qualification. We therefore recommend the use of bar graphs over dot graphs for the kinds of data presentations examined in this study. This recommendation is further supported by the likely greater familiarity of bar graphs for general readers and the ready availability of bar graphs in common, less sophisticated, statistical software products.

Pie charts are often derided because their non-linear format inhibits precise estimation of statistical quantities [17,18]. However, they do provide a visual representation of how each category contributes to the whole [7]. This is not easily achieved with other graph styles. The difficulty of estimating specific quantities or judging subtle differences from pie charts was confirmed in this study. For simple quantitative tasks such as identifying minimum and maximum categories or making comparisons where the differences were distinct, the pie chart performed as well as a bar chart. If an important aim is to visually represent how each category contributes to the whole, then a useful recommendation would be to use pie charts but ensure that the actual quantities are labelled on each segment of the pie chart.

### Scales and axes

Paired graphs showing a quantity separately for each sex, or for some other population characteristic, are common in population health publications. Several interpretation tasks explored the consequences of using differing scales in adjacent graphs. Many respondents, particularly those without university qualifications, appeared to answer questions based on visual relativities rather than from studying the labels on the axes. For tasks comparing the relative magnitude of quantities between the two graphs, a matching scale range on each graph greatly improved comprehension. If comparisons between adjacent graphs are important, then the same axis range should be used to avoid confusion. This is consistent with Kosslyn's recommendation [7], and should serve as a qualification of Cleveland's recommendation that data should fill the graph space [6]. If such comparisons are not important, then the two graphs should be presented with a distinct visual separation.

We found strong evidence for ensuring that higher values of the quantity presented on the graph are shown in the upward direction, even if this means the numerical labels are decreasing in the upward direction. This situation can arise when the risk of experiencing a disease is expressed as "1 in *x*", where *x *is the quantity graphed, because, for example, a 1 in 20 risk is larger than a 1 in 50 risk. Although this finding may be culturally specific, it would be reasonable to assume that for a horizontally oriented graph, the left-to-right direction should represent increasing values.

### Limitations of the study

Several issues need to be borne in mind when considering the findings of our study. Despite the randomised design, there were differences between the control and intervention groups in terms of self-rated visual ability and frequency of graph use. Intervention participants were somewhat more likely to rate themselves as frequent graph users than control participants and more likely to rate themselves as having good visual ability. However, the observed differences may reflect the fact that many of the intervention graphs were more easily understood than the control graphs. These questions were asked at the end of the questionnaire, and intervention participants may have felt more comfortable rating themselves more highly on these characteristics.

Because in some cases we made more than one change to the intervention graph, we could not completely attribute the impact of a single change to a single outcome. However, we aimed to minimise this difficulty by making the interpretation tasks as specific as possible to a specific intervention. This approach balanced respondent burden with the need to test many interventions for many graph styles.

The results we obtained are probably an overestimate of levels of comprehension that would be achieved in the general population. People working in public health and policy-related areas represented approximately one-fifth of respondents. These employees would be most likely to require information on population health statistics for their work. Many other people in the health system would have a professional understanding of health and medicine. Two-thirds of respondents in our study had university qualifications, compared with approximately one-fifth of the population aged 25–64 years in Australia [19].

The graphs we used were taken out of the context of their original report, and we recognise that much of the explanatory information required to understand the graph might have been contained in the surrounding text. However, if readers unfamiliar with the subject are required to hunt for explanatory information, they may weary of obtaining knowledge about population health. Publishers of scientific journals often require graphs to be able to "stand alone", and we support this objective, but would add that for documents intended for a public audience, the graphs should stand alone for a broad sector of the target readership.

Finally, in some cases we removed information contained in the control graph to test the effect of simplifying the graph. The information we removed may have been an important dimension that the original graph designers wanted to communicate. This study thus highlights the trade-off between detail and successful communication. An alternative to presenting multiple variables within one graph is to present a series of simpler graphs for each subgroup of an additional variable.

## Conclusion

Profound population health messages can be lost by the use of overly technical language and statistical measures that are unfamiliar to a general audience. This study provides new evidence to support a range of recommendations on how to improve the design of graphs. This represents a clear opportunity to improve delivery of public health messages through graphs to a wider sector of the population. However, it is clear that, regardless of graph design, concepts such as age standardisation and confidence intervals were not understood by the majority of participants, regardless of their level of education. This is a vexed problem, because these concepts are crucial to accurate interpretation of statistical information in population health and epidemiology. There remains, therefore, an opportunity for inventive solutions to deliver the messages implied by these manipulations without increasing the difficulty of interpreting the graph.

## Competing interests

The author(s) declare that they have no competing interests.

## Authors' contributions

DM managed the project, advised on the population health and epidemiological requirements of the project, and drafted the manuscript. AS and DM designed the survey. AS managed the survey and conducted the statistical analyses. RM and AS conducted the literature reviews for the project. LJ provided technical advice on the project and edited the manuscript. All authors agreed to the final version of the manuscript.

## Pre-publication history

The pre-publication history for this paper can be accessed here:



## Supplementary Material

Additional file 1 Full report of the literature reviews conducted for the project. Click here for file

Additional file 2 **Full report of the experimental study**. Contains the published report with complete results and appendices showing the control and intervention booklets and questionnaire Click here for file

## Figures and Tables

**Table 1:** Sample characteristics

	Intervention group		Control group	
	
Characteristic	Number (*n *= 176)	%	Number (*n *= 187)	%
Sex (male)	53	30.1%	47	25.1%
Age	37	21.0%	41	21.9%
< 34 years	109	61.9%	106	56.7%
35–54 years	27	15.3%	36	19.3%
≥55 years				
English as preferred language	171	97.2%	183	97.9%
Education (university qualification)	116	65.9%	124	66.3%
Work position*				
Clinical	61	34.7%	76	40.6%
Public health/policy	36	20.5%	35	18.7%
Other	72	40.9%	70	37.4%
Frequency of graph use				
Often	55	31.3%	44	23.5%
Occasionally or never	118	67.0%	141	75.4%
Self-rated visual ability				
Good	122	69.3%	110	58.8%
Average or poor	48	27.3%	74	39.6%

*Work position: clinical comprised doctors, nurses, allied health dealing with patients; non-clinical public health/policy comprised health-related but not dealing directly with patients; other included non-health admininistration, computing, clerical, maintenance.

Category totals may not add to 100% because of missing responses

**Table 2:** Counts of comprehension tasks by category of comprehension rate achieved, respondent status and educational attainment, for the 39 comprehension tasks included in the questionnaire

	All respondents		Non university-qualified		University-qualified	
	
Comprehension rate	Intervention (No. of tasks)	Control (No. of tasks)	Intervention (No. of tasks)	Control (No. of tasks)	Intervention (No. of tasks)	Control (No. of tasks)
Very low (0% to <20%)	0	1	0	3	0	2
Low (20% to <40%)	1	4	2	7	1	1
Moderate (40% to <60%)	3	9	3	6	1	7
High (60% to <80%)	7	8	13	10	6	11
Very high (above 80%)	28	17	21	13	31	18

**Table 3:** Prevalence of correct answers in the intervention ("Int.") and control ("Con.") group, and prevalence ratio of correct answers between the groups, for all respondents and by educational attainment

	**All respondents**				Non university-qualified*				University-qualified*			
			
**Interventions and interpretation tasks**	**Int. % (*n *= 176)**	**Con. % (*n *= 187)**	**Ratio**	**(95% CI)**	**Int. % (*n *= 56)**	**Con. % (*n *= 57)**	**Ratio**	**(95% CI)**	**Int. % (*n *= 116)**	**Con. % (*n *= 124)**	**Ratio**	**(95% CI)**
**Interventions: 1. Simplified series labels; 2. Footnote explaining age standardisation (see Figure 1)**												
Understand the meaning of a point reading of an incidence rate	80.7	57.2	1.4	(1.2–1.6)	76.8	45.6	1.7	(1.2–2.3)	81.9	62.9	1.3	(1.1–1.5)
Understand the influence of age standardisation on comparisons between incidence rates	58.0	36.9	1.6	(1.3–2.0)	42.9	22.8	1.9	(1.1–3.3)	65.5	44.4	1.5	(1.2–1.9)

**Interventions: 1. Removed one category from a stacked layer graph; 2. Footnote explaining how to interpret the thickness of a layer (see Figure 2)**												
For a single disorder, estimate the difference between incidence rates between two age points	57.4	57.8	1.0	(0.8–1.2)	51.8	47.4	1.1	(0.8–1.6)	60.3	63.7	0.9	(0.8–1.2)
Compare an incidence rate reading for a disorder by sex across adjacent graphs	85.2	88.2	1.0	(0.9–1.1)	83.9	82.5	1.0	(0.9–1.2)	87.1	90.3	1.0	(0.9–1.1)
Describe the trend by age along a layer in the graph	69.9	84.0	0.8	(0.7–0.9)	58.9	80.7	0.7	(0.6–0.9)	75.0	86.3	0.9	(0.8 – 1.0)
Broad comparison of the total rate (sum of all layers) within an age range by sex across adjacent graphs	89.2	85.6	1.0	(1.0–1.1)	89.3	87.7	1.0	(0.9–1.2)	90.5	83.9	1.1	(1.0–1.2)

**Interventions: 1. Changed a divided bar graph to a side-by-side divided bar graph; 2. Footnote explaining acronyms used in the graph (see Figure 3)**												
Compare the magnitude of YLL and YLD for a single disease category and sex	65.9	74.9	0.9	(0.8–1.0)	69.6	71.9	1.0	(0.8–1.2)	64.7	77.4	0.8	(0.7–1.0)
Know that YLD represents disability burden and select the disease with the highest value for a single sex	32.4	12.8	2.5	(1.6–3.8)	33.9	10.5	3.2	(1.4–7.5)	31.9	14.5	2.2	(1.3–3.6)
For a single disease, compare the magnitude of YLLs by sex	85.8	88.8	1.0	(0.9–1.1)	83.9	89.5	0.9	(0.8–1.1)	87.9	88.7	1.0	(0.9–1.1)
Select the disease with the highest number of DALYs for a single sex	83.0	67.9	1.2	(1.1–1.4)	80.4	61.4	1.3	(1.0–1.7)	85.3	71.8	1.2	(1.0–1.4)

**Intervention: Removed one of three independent variables from the graph so that bars became undivided and there was no need for a legend (see Figure 4)**												
Read the total rate of YLL for a single geographic category and sex	93.8	80.2	1.2	(1.1–1.3)	89.3	71.9	1.2	(1.0–1.5)	96.6	83.9	1.2	(1.1–1.3)
Broad comparison of the magnitude of YLL rates between two geographic categories, regardless of sex	94.9	90.4	1.1	(1.0–1.1)	94.6	84.2	1.1	(1.0–1.3)	95.7	94.4	1.0	(1.0–1.1)
Broad comparison of the magnitude of YLL rates between sexes, regardless of geographic category	92.6	92.5	1.0	(0.9–1.1)	89.3	84.2	1.1	(0.9–1.2)	94.8	96.0	1.0	(1.0–1.1)

**Intervention: Changed a population pyramid to a line graph (see Figure 5)**												
Broad comparison by sex of the overall population count across a range of age groups, for one geographic area	90.3	78.1	1.2	(1.1–1.3)	85.7	77.2	1.1	(0.9–1.3)	93.1	78.2	1.2	(1.1–1.3)
Broad comparison by geographic region across adjacent graphs of the total population size, regardless of age or sex	78.4	41.2	1.9	(1.6–2.3)	73.2	29.8	2.5	(1.6–3.8)	81.9	46.8	1.8	(1.4–2.2)
Broad comparison of the population size of younger and older segments of the population regardless of region	89.2	85.6	1.0	(1.0–1.1)	83.9	80.7	1.0	(0.9–1.2)	92.2	87.9	1.1	(1.0–1.1)

**Interventions: 1. Changed a dot graph with confidence intervals ('hi-lo-close') graph to a bar graph; 2. Footnote providing a simple practical description of confidence intervals (see Figure 6)**												
Interpret the statistical significance of the difference between two countries of birth in the proportion of premature births	54.5	31.6	1.7	(1.4–2.2)	39.3	15.8	2.5	(1.3–4.9)	62.9	40.3	1.6	(1.2–2.0)
Compare the relative magnitude of the proportion of premature births between two countries of birth represented by adjacent graph bars	91.5	84.5	1.1	(1.0–1.2)	92.9	71.9	1.3	(1.1–1.5)	91.4	90.3	1.0	(0.9–1.1)
Compare the relative magnitude of the proportion of premature births between two countries of birth represented by more distant graph bars	79.5	50.3	1.6	(1.4–1.9)	80.4	35.1	2.3	(1.6–3.3)	80.2	58.1	1.4	(1.2–1.7)

**Interventions: 1. Changed the title to a plain question that reflected the intepretation of the graph; 2. Changed some numeric y axis labels to descriptive explanations relating to the title; 3: Removed the footnote that had become redundant (see Figure 7)**												
Broad judgement of whether Aboriginal people had a higher risk of death than Australians overall	82.4	58.8	1.4	(1.2–1.6)	69.6	38.6	1.8	(1.3–2.6)	90.5	69.4	1.3	(1.2–1.5)
For one age group and sex, read the point estimate of the rate ratio	83.0	55.6	1.5	(1.3–1.7)	69.6	36.8	1.9	(1.3–2.8)	91.4	65.3	1.4	(1.2–1.6)
Understand the meaning of a death rate ratio for one age group and sex	84.7	59.9	1.4	(1.2–1.6)	71.4	42.1	1.7	(1.2–2.4)	92.2	69.4	1.3	(1.2–1.5)

**Intervention: Reversed the scale of the vertical axis to represent increasing risk in the upward direction (see Figure 8)**												
Judge the relative magnitude of risk between the sexes in a single year	79.5	48.7	1.6	(1.4–1.9)	66.1	31.6	2.1	(1.4–3.2)	87.1	58.1	1.5	(1.3–1.8)
For one sex, judge the direction of the trend over time	60.2	20.9	2.9	(2.1–9.9)	62.5	19.3	3.2	(1.8–5.7)	58.6	21.8	2.7	(1.9–3.9)
Read the point estimate of risk for a single sex in a single year	90.9	85.6	1.1	(1.0–1.1)	78.6	77.2	1.0	(1.0–1.4)	97.4	91.1	1.1	(0.9–1.1)

**Interventions: 1. Made the y axis ranges on two adjacent graphs match; 2. Slight simplification to the graph title (see Figure 9)**												
Broad judgment by virus across adjacent graphs of the relative difference in prevalence between the two subgroups	90.9	45.5	2.0	(1.7–2.4)	89.3	35.1	2.5	(1.8–3.7)	93.1	51.6	1.8	(1.5–2.2)
Broad judgement of which subgroup had a lower prevalence of HCV infection	80.7	75.9	1.1	(1.0–1.2)	78.6	66.7	1.2	(1.0–1.5)	81.9	79.8	1.0	(0.9–1.2)
Broad comparison by virus across the two graphs of the prevalence of infection in a single year, regardless of subgroup	92.0	63.6	1.5	(1.3–1.6)	87.5	47.4	1.9	(1.4–2.5)	94.8	73.4	1.3	(1.2–1.5)
Point reading of prevalence of HCV infection for a single year and subgroup	71.0	73.3	1.0	(0.9–1.1)	64.3	63.2	1.0	(0.8–1.3)	74.1	78.2	1.0	(0.8–1.1)

**Intervention: Changed the graph type from a vertical bar graph to a line graph (see Figure 10)**												
Point reading of the proportion of deaths caused by a disease in a single year	83.0	82.9	1.0	(0.9–1.1)	78.6	73.7	1.1	(0.9–1.3)	86.2	88.7	1.0	(0.9–1.1)
Judge which disease had the lowest proportion of deaths in a single year	96.6	94.1	1.0	(1.0–1.1)	96.4	87.7	1.1	(1.0–1.2)	97.4	97.6	1.0	(1.0–1.0)
Judge which disease had the most increasing trend in the proportion of deaths over the period shown	83.5	76.5	1.1	(1.0–1.2)	75.0	56.1	1.3	(1.0–1.8)	89.7	85.5	1.1	(1.0–1.2)

**Intervention: Changed a dot graph to a bar graph (see Figure 11)**												
Judge which sex had the greater proportion for a single injury category	93.8	89.3	1.1	(1.0–1.1)	92.9	78.9	1.2	(1.0–1.4)	94.8	95.2	1.0	(0.9–1.1)
Judge which injury category had the greatest proportion of hospital separations within a single sex	96.0	94.1	1.0	(1.0–1.1)	94.6	89.5	1.1	(1.0–1.2)	97.4	97.6	1.0	(1.0–1.0)

**Intervention: Changed the graph type from a pie chart to a horizontal bar graph (see Figure 12)**												
Identify the category accounting for the largest proportion of cancers in a single sex	97.7	96.8	1.0	(1.0–1.1)	96.4	93.0	1.0	(1.0–1.1)	99.1	100.0	1.0	(1.0–1.0)
Identify the larger of two categories for a single sex	96.6	95.2	1.0	(1.0–1.1)	94.6	93.0	1.0	(1.0–1.1)	98.3	97.6	1.0	(1.0–1.1)
Comparison by sex across adjacent graphs of the contribution of one cancer to all cancers in each sex	95.5	63.6	1.5	(1.3–1.7)	92.9	80.7	1.2	(1.0–1.3)	97.4	56.5	1.7	(1.5–2.0)
Identify the cancer accounting for the smallest proportion of all cancers in a single sex	96.6	90.9	1.1	(1.0–1.1)	94.6	91.2	1.0	(0.9–1.2)	98.3	91.9	1.1	(1.0–1.1)
Point reading of the proportion of all cancers contributed by a single cancer for a single sex	92.0	25.7	3.6	(2.8–4.6)	91.1	40.4	2.3	(1.6–3.1)	93.1	19.4	4.8	(3.4–6.9)

Con., control; DALY, disability-adjusted life year; HCV, hepatitis C virus; Int., intervention; YLD; years of living with a disease; YLL, years of life lost.

*The number of university and non university-qualified participants does not add to the number of all respondents because 2% of intervention participants and 3% of control participants did not report their educational attainment.
