# Supplementary material for: Communicating population health statistics through graphs: a randomised controlled trial of graph design interventions
Source: BMC Med. 2006 Dec 20;4:33. doi: 10.1186/1741-7015-4-33 (PMC1766925; doi:10.1186/1741-7015-4-33)
Supplement: Additional file 1 — Full report of the literature reviews conducted for the project. [file 1741-7015-4-33-S1.pdf]

NSW HEALTH

Better health graphs

Volume 2

The literature reviews

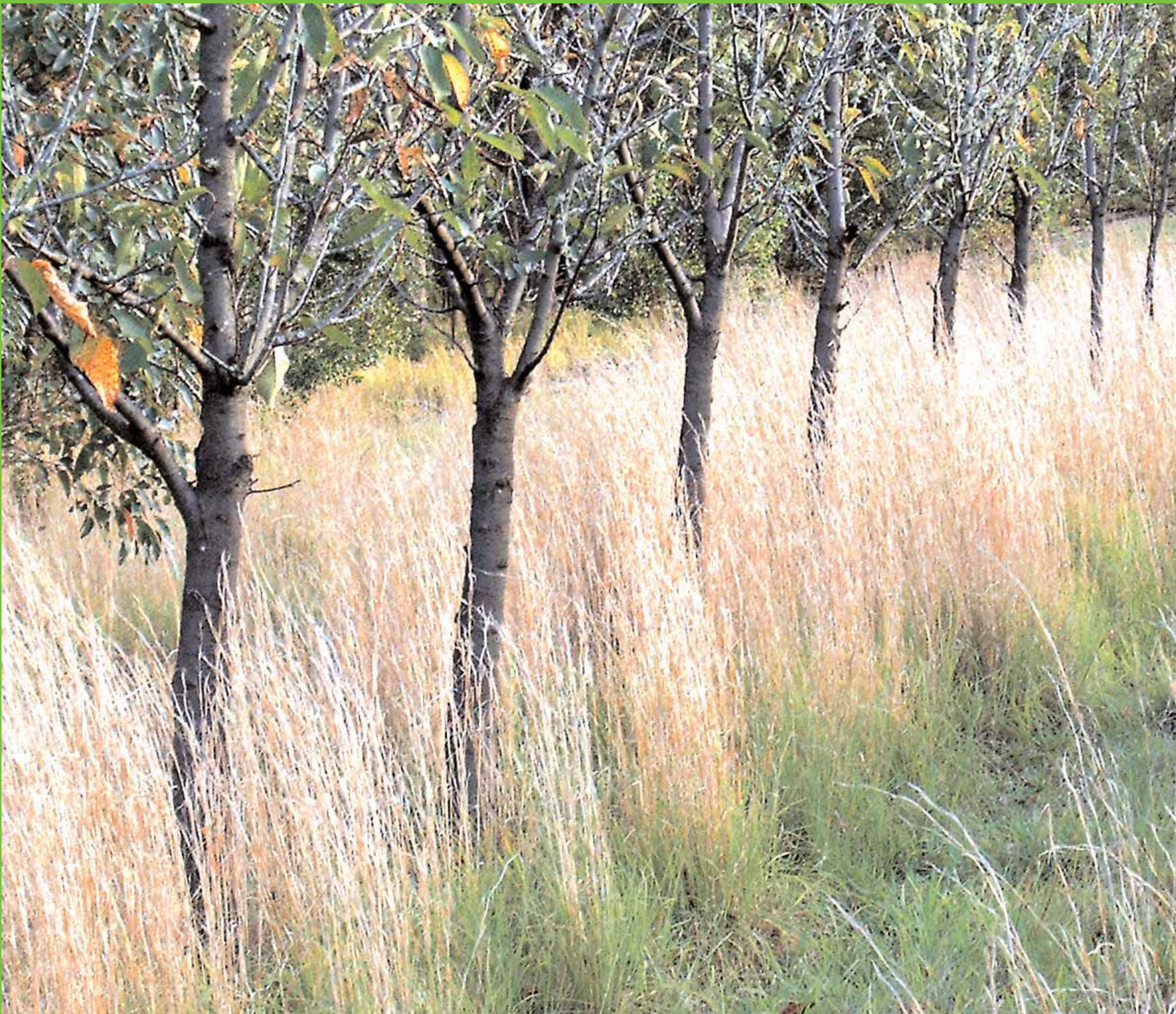

Copyright © NSW Department of Health, July 2006

This work is copyright. It may be reproduced in whole or in part, subject to the inclusion of an acknowledgment of the source and no commercial usage or sale.

State Health Publication No: HSP 060049
ISBN 0 7347 3923 0

suggested citation:
Centre for Epidemiology and Research and Hunter Valley Research Foundation, *Better Health Graphs (Volume 2): The Literature Reviews*. Sydney: NSW Department of Health, 2006.

produced by:
Centre for Epidemiology and Research
Population Health Division
NSW Department of Health
Locked Mail Bag 961
North Sydney NSW 2059 Australia
Tel: 61 2 9391 9408
Fax: 61 2 9391 9232

further copies of this publication can be obtained
from the NSW Department of Health website at:
www.health.nsw.gov.au

Contents

Executive summary..5

1 Introduction 10

2 Literature review: Documentation of graph styles.......................................12

2.1 Documentation of graph styles identified in Australian population health publications............12

2.2 Source material 12

2.2.1 Collection of population health publications for review 12

2.2.2 A graph classification system 14

2.2.3 Definitions of measures used for classifying graphs 14

2.2.4 Characteristics observed during the classification of graphs 15

2.2.5 Cataloguing graphs used in selected population health publications 16

2.2.6 Cataloguing graphs using the criteria of ‘measurement’ 16

2.3 Graphs displaying rates 16

2.4 Graphs displaying proportions............................20

2.5 Graphs displaying frequency 25

2.6 Graphs displaying central tendency 28

2.7 Graphs displaying ratios 31

2.8 Graphs displaying risk 34

2.9 Graphs displaying life expectancy........................36

Bibliography – graph styles..........................40

3 Literature review: Best practice principles for graph design..................42

3.1 Introduction – even the experts don’t get it right...42

3.2 Conventions used in this section 42

3.3 To graph or not to graph 43

3.4 The relevance of perceptual issues to graph construction – why do perceptual issues matter? 46

3.5 Founding members of the perceptual issues debate 47

3.6 Considerations of requisite task in determining preferred graph type 48

3.7 Users’ expertise in constructing and reading graphs...51

3.8 Guiding principles for graph design.....................54

3.9 Pie charts 63

3.10 Line graphs 65

3.11 Bar graphs 66

3.12 Scatter plots...68

3.13 The display of multiple graphs............................68

3.14 Integrating graphics and text 69

3.15 Typeface and size 69

3.16 The shape of a graph 70

3.17 Captions 71

3.18 Labels...71

3.19 Reference lines and error bars 72

3.20 Legends and keys...73

3.21 Axes, scales and tick marks 74

3.22 The use of colour in graphs................................76

3.23 Three dimensional graphs 77

3.24 Line and symbol weight and type........................78

3.25 Background features and grid lines 78

3.26 Conclusion – is there hope for the graphically challenged?...79

3.27 Summary of best practice principles 80

Bibliography – graph design 84

4 **Literature review: Techniques to test reader understanding of graphs 87**

4.1 Introduction 87

4.2 The study setting and implementation techniques 88

4.2.1 Some examples of techniques measuring accuracy and time 91

4.3 Selection of subjects 92

4.4 Training subjects in the experiment's tasks 94

4.5 Complexity in the graphical display 95

4.6 Questions and questioning techniques to evaluate comprehension 96

4.6.1 Questions using a holistic approach for evaluating graph comprehension 100

4.7 Analysis techniques 101

4.8 Potential interaction 102

4.9 Recommendations 103

4.9.1 Recommendations for study design 103

4.9.2 Recommendations for subjects and subject selection 104

4.9.3 Recommendations for questionnaire design 104

Bibliography – questionnaire design 106

List of figures

Figure 1 Count of graphs found in the reviewed publications displaying each of the statistical measures used for cataloguing 17

Figure 2 Example of graphs displaying rates: Sourced from 'Cancer in Australia 1997', Australian Institute of Health and Welfare, Australian Association of Cancer Registries, 2000, p. 19 18

Figure 3 Example of a graph displaying rates: Sourced from 'Health Indicators for Queensland: Central Zone', Public Health Services, Queensland Health, 2001, p. 50 18

Figure 4 Example of a graph displaying rates: Sourced from 'Demographic and Health Analysis of the Northern Regions', Report No. 4, Tasmanian Department of Health and Human Services, 2000, p. 10 19

Figure 5 Example of a graph displaying rates: Sourced from 'From Infancy to Young Adulthood, Health Status in the Northern Territory', Territory Health Services, 1998, p. 46 19

Figure 6 Example of a graph displaying rates: Sourced from 'Victorian burden of disease study, Mortality', Victorian Department of Human Services, 2000, p. 22 20

Figure 7 Example of a graph displaying proportions: Sourced from 'Heart, stroke and vascular diseases, Australian facts 2001, Australian Institute of Health and Welfare, National Heart Foundation of Australia, National Stroke Foundation of Australia', 2001, p. 59 21

Figure 8 Example of a graph displaying proportions: Sourced from 'Australia's Health 2000: the seventh biennial health report of the Australian Institute of Health and Welfare', Australian Institute of Health and Welfare, 2000, p. 156 22

Figure 9 Example of a graph displaying proportions: Sourced from 'The impact of Diabetes in South Australia 2000', South Australian Department of Human Services, South Australia, 2000, p. 10 22

Figure 10 Example of a graph displaying proportions: Sourced from 'The Health and Welfare of Australia's Aboriginal and Torres Strait Islander Peoples', ABS Cat. no. 4704.0, AIHW Cat. no. IHW 6, Canberra 2001 (www.abs.gov.au), p. 82 23

Figure 11 Example of a graph displaying proportions: Sourced from 'Health indicators in the ACT', Epidemiology Unit, ACT Dept of Health and Community Care: Health series No. 13, ACT Government Printer, 1999, p. 50 23

Figure 12 Example of a graph displaying proportions: Sourced from 'The health of the people of NSW – Report of the Chief Health Officer', NSW Health Department, Sydney, 2000, p. 89 24

Figure 13 Example of a graph displaying proportions: Sourced from 'The health and welfare of Territorians', Epidemiology Branch, Territory Health Services, 2001, p. 2 24

Figure 14 Example of a graph displaying frequency: Sourced from 'The health of the people of New South Wales- Report of the Chief Health Officer', NSW Health, 2000, p. 257 26

Figure 15 Example of a graph displaying frequency: Sourced from 'Victorian Burden of Disease Study: Morbidity' Public Health Division, Victorian Department of Human Services, 1999, p. 74...................................26

Figure 16 Example of graphs displaying frequency: Sourced from 'Health Indicators for Queensland: Central Zone', Public Health Services, Queensland Health, 2001 p. 14 27

Figure 17 Example of a graph displaying a measure of central tendency: means. Sourced from 'Health indicators in the ACT', Epidemiology Unit, ACT Department of Health and Community Care: Health series No. 13, ACT Government Printer, 1999, p. 30. ..29

Figure 18 Example of a graph displaying a measure of central tendency: means. Sourced from 'The health of the people of New South Wales, Report of the Chief Health Officer', NSW Health, 2000, p. 236 (left hand graph only) 29

Figure 19 Example of a graph displaying a measure of central tendency: means. Sourced from 'Health Measures for the Population of Western Australia: Trends and comparisons', Health Department of Western Australia, 2000, p. 26 30

Figure 20 Example of a graph displaying a measure of central tendency: medians. Sourced from 'Health Indicators for Queensland: Central Zone', Public Health Services, Queensland Health, 2001, p. 39....................30

Figure 21 Example of a graph displaying a measure of ratios: Sourced from 'Cancer in New South Wales: Incidence and mortality 1999 featuring 30 years of cancer registration', Cancer Council NSW, 2001, p. 129 32

Figure 22 Example of a graph displaying a measure of ratio: Sourced from 'Health Indicators for Queensland: Central Zone', Public Health Services, Queensland Health, 2001, p. 164 32

Figure 23 Example of a graph displaying a measure of ratios: Sourced from 'Health Measures for the Population of Western Australia: Trends and comparisons', Health Dept of Western Australia, 2000, p. 190..33

Figure 24 Example of a graph displaying a measure of ratios: Sourced from 'The Health and Welfare of Territorians', Epidemiology Branch, Territory Health Services, 2001, p. 192 33

Figure 25 Example of a graph displaying a measure of risk: Sourced from 'Health Measures for the Population of Western Australia: Trends and comparisons', Health Dept of Western Australia, 2000, p. 81 35

Figure 26 Example of a graph displaying a measure of risk: Sourced from 'Health Measures for the Population of Western Australia: Trends and comparisons', Health Dept of Western Australia, 2000, p. 130 36

Figure 27 Example of a graph displaying a measure of life expectancy: Sourced from 'The health of the people of New South Wales – Report of the Chief Health Officer', NSW Health, 2000, p. 63 37

Figure 28 Example of a graph displaying a measure of life expectancy: Sourced from 'Health Measures for the Population of Western Australia: Trends and comparisons', Health Dept of Western Australia, 2000, p. 70...38

Figure 29 Example of a graph displaying life expectancy: Sourced from 'Victorian burden of disease study, Mortality', Victorian Department of Human Services, 2000, p. 16 39

Figure 30 Example of a graph displaying life expectancy: Sourced from 'Victorian burden of disease study, Mortality', Victorian Department of Human Services, 2000, p. 17 39

Acknowledgments

This project was jointly funded by the Commonwealth Department of Health and Ageing and the Centre for Epidemiology and Research, NSW Department of Health. The project was conducted under the National Population Health Information Development Plan (Australian Institute of Health and Welfare, 1999). The Hunter Valley Research Foundation conducted the project under contract to the NSW Department of Health. These final reports represent the collaborative work of the Hunter Valley Research Foundation and staff from the Centre for Epidemiology and Research, in particular:

The Hunter Valley Research Foundation

- Mr Andrew Searles
- Ms Robin Mcdonald

Centre for Epidemiology and Research, NSW Department of Health

- Mr David Muscatello

The Centre for Epidemiology and Research would like to acknowledge the valuable assistance of the additional members of the project Steering Committee:

- Dr Tim Churches, Centre for Epidemiology and Research
- Dr Paul Jelfs, Australian Institute of Health and Welfare
- Dr Louisa Jorm, Centre for Epidemiology and Research
- Ms Jill Kaldor, Centre for Epidemiology and Research

Graphs in this publication were reproduced with permission from the following organisations and this permission is gratefully acknowledged:

Australian Bureau of Statistics (www.abs.gov.au)

Australian Capital Territory (ACT) Department of Health and Community Care

Australian Institute of Health and Welfare

Health Department of Western Australia

Queensland Health

New South Wales Department of Health

Northern Territory Department of Health and Community Services

South Australian Department of Human Services

Tasmanian Department of Health and Human Services

Victorian Department of Health and Human Services

Executive summary 1

Introduction

There is a widespread use of graphs in population health reports and there is little doubt that a well-constructed graph can go a long way to enhancing reader understanding of a set of data. Unfortunately the preparation of graphs is, at times, haphazard and a poorly designed graph can make comprehension by the reader more difficult. At worst, the reader can be misled and arrive at an incorrect conclusion.

Unlikely as it is that graph authors in population health publications would deliberately set out to complicate data, during the course of this study many examples were found of graphs that required considerable effort to interpret. Of course, there were also many examples of graphs at the other end of the spectrum: creatively prepared and conveying multiple layers of data with precision and efficiency.

Aim of the study

The study aimed to identify suggestions that would assist graph authors design better population health graphs. In this study, 'better' meant an improved level of reader comprehension.

Content of this report

The project had two parts: a set of literature reviews and an experimental study. This report discusses the literature reviews. A second report, which can be found at www.health.nsw.gov.au, presents the results of the experimental study, which was a randomised controlled trial of interventions to improve graph comprehension.

This report contains a review of the literature in three interrelated areas:

1 a summary of graphs used in Australian population health publications;

2 recommendations for designing graphs from the literature;

3 techniques that have been used to evaluate reader understanding of graphs.

Results

Twenty national and State reports were reviewed in Part 1 of the review to determine the range and style of graphs and statistics reported in Australia. The main statistical measures reported in graphs from these publications included: rates, proportions (or prevalence), frequencies, measures of central tendency (averages or means), ratios, risk and life expectancy. The results of this review provided an important input into determining which graphs and statistical measures to focus on in the experimental study.

For part 2 of the review, each principle identified in the literature was assigned a level of evidence as follows:

L1 Level 1: Tested experimentally in a large representative population; high level of evidence

L2 Level 2: Tested experimentally in a selected or small population or based upon established theory; medium level of evidence

L3 Level 3: Expert opinion; low level of evidence.

The main findings of the review of graph design principles are presented in the Table at the end of the Executive Summary, along with their level of evidence.

For part 3, techniques were reviewed that have been used to measure reader understanding in studies of graph comprehension. The recommended study design is the randomised controlled trial, which was used by several graph researchers in the late 1980s and early 1990s. In laboratory conditions, a computer display can be used to present graphs for the study in controlled conditions. However, in population-based studies, controlled conditions can best be met with printed booklets of graphs. In measuring comprehension, the important measure is the accuracy of the subjects' answers to closed-ended questions about the graphs. Questions should cover both 'global' or broad interpretations as well as 'local' or specific estimation questions.

Findings from the literature review of graph design principles

Graph design feature	Level of evidence
**General principles**	
1. Ensure that tasks supported by the graph constructed are consistent with the tasks which readers will be required to undertake.	L2
2. A single graph should be able to support 'global' interpretation tasks by being configured to produce *emergent features*, as well as 'local' interpretation tasks by emphasising its elemental properties. Emergent features are produced by the interactions among individual elements of a graph, for example, lines, contours and shapes which occur when two variables are mapped – one in the x axis and one in the y axis – to produce the emergent features of area.	L2
3. Use *common graphs* with which all readers are likely to be familiar: for example, line and bar graphs, pie charts and scatter plots.	L2
4. The conventions of a reader's culture should be obeyed: for example, the colour red should not be used to signify 'safe' areas, and green should not be used to signify 'danger'; numerical scales should increase going from left to right or bottom to top.	L2
5. Similarly, the appearance of words, lines and areas in a graph should be compatible with their meanings, for example, the word 'red' should not be written in blue ink, larger areas in the display should represent larger quantities, and faster rising lines should represent sharper increases.	L2
6. If there is a possibility that readers may lack the necessary background knowledge to interpret a chart, then a sufficient amount of *domain-specific* information should be included in the text to ensure adequate comprehension of the accompanying chart(s). Additionally, charts should be labelled to provide domain-specific information, including a full explanation of all abbreviations and acronyms: preferably, these should be avoided altogether.	L2
7. *Context-specific* support should also be provided by spelling out in the accompanying text the nature of the relationships illustrated in the graph so that the two reinforce the relevant message	L2
8. Graphs, relevant text and, where appropriate, statistical analysis in a document should be integrated in close physical proximity. The terminology used in the display should be the same as that in the text or presentation.	L2
9. Do not over adorn charts or include an excessive amount of information in them. This recommendation will necessarily involve judgments necessary to limit the amount of clutter in a chart, while at the same time ensuring that the intended message is clear and unambiguous, as well as being aesthetically appealing enough to be read. Probably the best and safest approach to take is to start with a relatively minimal presentation and include more only if a strong reason for doing it can be explicitly articulated.	L2
10. Visual clarity is essential. To this end, all 'marks' on a graph must have a minimal magnitude to be detected, and they must be able to be perceived without distortion. Marks must also be relatively discriminable: that is, two or more marks must differ by a minimal proportion to be discriminated. Design graphs so that the visual clarity is maintained if, in the future, they are copied.	L2
11. Construct graphs so the more important things are noticed first. That is, the main point of the graph should be its most visually salient feature, and one should avoid making the reader search for the main point in the details of the graph. Marks should be chosen to be noticed in accordance with their importance in the display, and the physical dimensions of marks should be used to emphasise the message; they should not distract from it. For example, inner grid lines should be lighter than content lines, and background patterns or colours should never be as noticeable as the content components of the graph itself.	L2
12. Include in a graph only (but at least) the necessary amount of data to make the relevant point(s). Excess or irrelevant data can hinder trend reading tasks.	L2
13. In general, lengths should be used, as opposed to areas, volumes or angles, to represent magnitudes wherever possible. Most preferable is the plotting of each measure as a distance from a common baseline so that *aligned lengths* are being compared. Note, however, that the use of pie charts for part-to-whole comparisons, discussed below, is an exception to this rule	L2
14. Data which are to be compared should be in close spatial proximity.	L2

Graph design feature	Level of evidence
**General principles**	
15. A related (or even the same) principle is that when there is more than one independent variable to be considered, the most important one should be on (and label) the x axis, and the others should be treated as parameters representing separate bars or lines. For example, if comparisons between categories *within a given year are* to be described, then an appropriate format would be sets of bars grouped such that each cluster consisted of x number of different categories within a given year. To describe comparisons of a *single category across years*, an appropriate format would be the use of dots connected by lines such that each line consists of a measurement of a single category across years.	L2
16. If there is no clear distinction between the importance of the variables, put an interval scaled independent variable (if there is one) on the x axis. The progressive variation in heights from left to right will then be compatible with variation in the scale itself. If there is more than one independent variable with an interval scale, put the one with the greatest number of levels on the x axis.	L2
17. When possible, use graphs that show the results of arithmetic calculations (for example, a stacked bar graph for addition). Otherwise, design graphs which minimise the number of arithmetic operations which readers must undertake to complete the required task. For example, where the focus is on the difference between two functions, a single line showing the difference should be drawn, rather than the two original functions. If the slope, or rate of change, of a function is most important, the rate of change should be plotted rather than the original data.	L2
18. Design graphs (especially graphs in a series) to have a consistent layout such that the location of indicators is predictable.	L2
19. Limit the number of lines on a chart and number of bars over each data point. Evidence suggests that where there are more than four groups of lines on a chart, or four bars over each point, it is best to divide the data into subsets and graph each subset in separate panels of a display. To aid search across multiple graphs with many levels of an independent variable:<br>• Place all graphs in one figure to facilitate search across graphs.<br>• Use spatial proximity for graphs so that the reader might search in sequence.<br>• Maintain visual consistency across graphs (for example, use the same size axes, the same types of indicators, and the same coding of indicators for the same variables).<br>• Maintain semantic consistency across graphs (for example, use the same scale on the y axis).<br>• Eliminate redundant labels (for example, in a series of horizontally aligned graphs, the labels for the Y axes should be placed only on the leftmost graph, and the label naming the x axis should be centred under the series).<br>• Avoid using a legend to label indicators (a legend requires multiple scans between the indicators and the legend, thereby disrupting visual search); label indicators (lines, bars, pie segments etc.) directly.<br>• Assign data that answer different questions to different panels.<br>• Assign lines that form a meaningful pattern to the same panel.<br>• Put the most important panel first.	L2

Graph design feature	Level of evidence
**Choice of graph type**	
20. Pie charts should be used for *part-to-whole* judgments involving comparison of one proportion of an item to its whole, never for *part-to-part* judgments involving a decision of what proportion a smaller value is of a larger, where the smaller value does not form part of the larger one (as would be the case when *changes* in the magnitude of a variable are to be detected).	L2
21. Line graphs are most appropriate for showing data trends and interactions, and identifying global patterns in data, though bar graphs also support these tasks. Note that trends can be described in terms of *rising*, *falling*, *increasing*, or *decreasing*. There is some evidence that line graphs are more biasing than bar graphs: that is they emphasise x-y relations. Consequently, if two independent variables are equally important, bar graphs should be used. If a particular trend is the most important information, then line graphs should be used.	L2
22. The literature is divided over the preference of lines or bars to facilitate the extraction of exact values, though it probably comes down in favour of bars.	L2
23. When *multiple trends* are to be compared, showing several trend lines on a *single* graph is superior to presenting single trend lines on several graphs. This type of 'layer' graph is useful in illustrating the relative change in one component over changes in another variable. Because the spaces between the lines can be filled, they can be seen as shapes, and the change in a single proportion can be easily seen. However, note that layer graphs should only be used to display continuous variables: that is, values on an interval scale. If the x axis is an ordinal scale (one that specifies ranks) or nominal scale (one that names different entities) the eye will incorrectly interpret the quantitative differences in the slopes of the layers as having meaning. In these cases the use of a divided bar graph is recommended.	L2
24. Single line graphs are also most effective for indicating data *limits* (maxima and minima – for example, the year in which product A's sales peaked; and *conjunctions* (the intersection of two indicators – for example, the year in which product A first sold more than product B).	L2
25. While line graphs are to be most preferred for showing trends, bar graphs run a close second, as long as they are vertical, not horizontal. Bar graphs are also preferred if precise values need to be detected, and they are a good 'compromise' if both local (or *discrete*) and global interpretations of the data need to be made. *Discrete comparisons* can be described in terms of *higher*, *lower*, *greater than*, or *less than*.	L2
26. Bar graphs are also useful for displaying data limits (maxima and minima), and *accumulation* (the summation indicators – for example, to determine which of several products sold the most overall).	L2
27. The balance of evidence supports the use of vertical, rather than horizontal bar graphs for discrete comparisons, though there is 'room' for subjective consideration of the data to determine a preference. When in doubt, use a vertical bar graph format, since increased height may be considered a better indicator of increased amount.	L2
28. 'Side-by-side' horizontal bar graphs which show pairs of values (values for two independent variables) that share a central y axis are recommended to show contrasting trends between levels of an independent variable, and comparisons between individual pairs of values.	L2

Graph design feature	Level of evidence
**Graph elements**	
29. Captions should be visually prominent, preferably centred at the top of the chart. They should describe everything that is graphed in terms of *what*, *where*, *who* and *when* and any other descriptors considered to be necessary..	L3
30. Placement of numerical values directly onto a graph will aid local interpretation tasks.	L2
31. Tick marks should be included on the axes, and labelled at regular intervals, using round numbers. Where the range of values on an axis dictates that labels should include decimal points to be meaningful, the number of decimal places should be kept to a minimum.	L2
32. Axis labels should be centred and parallel to their axis: that is, the y axis should not be labelled horizontally on the top left hand side of the data rectangle.	L2
33. For graphs that are wide, place y axes and the associated numerical labels on both sides of the data.	L2
34. Inclusion of a *reference line* is recommended when there is an important value that must be seen across the entire graph, as long as the line does not interfere with the data.	L2
35. The use of error bars to show variability in the data being graphed is also recommended. If the error bars on a line graph overlap so that they cannot be discriminated for data at the same level of the independent variable on the x axis, either or both of the following is suggested:<br>• Display the data in a bar graph (because the bar indicators for data at the same level of the independent variable are not vertically aligned, so the error bars will not overlap)<br>• Where confidence intervals are symmetric about the point estimate, show only the top half of the error bars on the upper line and the bottom half of the error bars on the bottom line.	L1
36. Wherever possible, directly label indicators in a graph rather than including a legend to explain their meaning. Labels should be in as close proximity as possible to their associated graph element.	L2
37. Where the use of a legend is unavoidable because the indicators are too close to be labelled unambiguously, it should be placed close to the indicators to reduce scanning distance, but not so close as to interfere with the indicators. The order of the symbols in the legend should match the order of the indicators in the graph.	L2
38. While a contentious issue, majority evidence suggests that starting the y axis visible scale at zero is not essential unless the zero value is inherently important. However, note that a zero value must be retained for divided bar charts because the main point of this type of chart is to convey information about the relative proportions of the different portions of the whole. Excising part of the scale on the y axis will alter the visual impression so that the sizes of the segments no longer reflect the relative proportions of the components.	L2
39. In general, the range of the scale should be chosen to illustrate the relevant point(s) the author wishes to make, with the visual impression produced by the display conveying actual differences and patterns in the data. Do not make the scale maximum any larger than necessary to accommodate all data points; otherwise, a portion of the upper plot area will be left empty.	L2
40. It is best to avoid the use of multiple scales on a chart (a separate one on each of the left and right Y axes). A possible exception to this rule is when the dependent variables are intimately related, and their interrelations are critical to the message being conveyed. In this case, plotting the data in the same display allows them to be perceived as a single pattern. Line graphs are usually the most appropriate for this purpose, and use of the same colour or pattern to plot the line and the corresponding y axis should be considered.	L2
41. Avoid the use of three dimensional graphs because perceptual biases may mean that comparisons of height or length at different depths are variable. If they are used, a good 3D chart should be viewed from the perspective of top looking down, so that the face of the bar or column represents the actual reading.	L2

Introduction 1

Why the interest in graphs?

> *There is no single statistical tool that is as powerful as a well-chosen graph. Our eye brain system is the most sophisticated information processor ever developed, and through graphical displays we can put this system to good use to obtain deep insight into the structure of data* (Chambers *et al.* 1983, p. 1).

There is a widespread use of graphs in population health reports and there is little doubt that a well-constructed graph can go a long way to enhancing reader understanding of a set of data. Unfortunately the preparation of graphs is, at times, haphazard and a poorly designed graph can make comprehension by the reader more difficult. At worst, the reader can be misled and arrive at an incorrect conclusion.

> *The aim of good data graphics is to display data accurately and clearly. ….. The definition has three parts. These are (a) showing data, (b) showing data accurately, and (c) showing data clearly.* (Wainer, 1984 p. 137).

Unlikely as it is that graph authors in population health publications would deliberately set out to complicate data, during the course of this study many examples were found of graphs that required considerable effort to interpret. Of course, there were also many examples of graphs at the other end of the spectrum: creatively prepared and conveying multiple layers of data with precision and efficiency.

Content of this report

The results of this study are presented in two reports. This report contains a review of the literature in three interrelated areas:

1 A summary of graphs used in current Australian population health publications.

2 A review of the literature on graph design principles and techniques.

3 Techniques that have been used to evaluate reader understanding of graphs.

The second report presents the results of an experimental study, conducted as a randomised control trial to test elements of graph design for population health graphs. That report can be found at www.health.nsw.gov.au.

The literature reviews

The literature review had three aims. Firstly to identify the types of graphs used in Australian population health reports. To undertake this task, a classification system was developed to identify broad graph categories. These categories became the primary unit for grouping like graphs together. Examples of each group were selected and their characteristics recorded and, where appropriate, critiqued.

The second aim of the literature review was to evaluate journal papers and textbooks that discussed aspects of 'best practice' graphing. A systematic approach was adopted when writing the results to this part of the review. The approach attempted to disassemble elements of the graph and identify the 'best' way of re-constructing that element. Naturally, this was not always straightforward because of the element's impact on the whole graph, which had to be taken into account. There was also considerable overlap between graph elements that made this 'in isolation' prescription difficult. Nonetheless, a systematic methodology was required and without an obvious alternative, this was the approach taken.

The third aim of the literature review was the identification of techniques to evaluate reader comprehension of graphs. Many of the techniques identified during the review were applied in controlled laboratory conditions with small samples. In contrast, the current project used combined mail and telephone data collection and had by comparison, a large sample size. Despite the differences in study design, many of the tests found in the literature provided material for assessing reader comprehension of graphs in the current study.

Conventions in this report

Quotations

Direct quotations from authors are provided in *italics*, with an accompanying page number reference. Italic type is also used occasionally for emphasis.

Reduced scale of graphs

The second section in this report documents examples of graph types found in Australian health publications. These figures have been rescaled from tto fit into the available space in this report and therefore, they are not direct representations.

Bibliographies

Each of the literature reviews and the write-up of the survey results is followed by its own bibliography.

Literature review: Documentation of graph styles

2

2.1 Documentation of graph styles identified in Australian population health publications

A review of the use of graphs in Australian population health publications was undertaken to help guide the development of the study with regard to the types of 'measures' that were being communicated. These publications were sourced from government departments at the State, Territory and Commonwealth level. Once obtained, the graphs used in these publications were examined to identify their main characteristics. These characteristics were compared to a classification system developed as part of the study and were used to place the graph into one of seven identified categories.

The characteristic on which the classification was based was the 'measure' displayed by the graph. Seven measures and, therefore, seven categories of graph were identified: those displaying frequencies, proportions, rates, central tendency (means and medians), ratios, life expectancy and risk.

As the volume of publications that could have been considered for this part of the review was beyond the study's time resources, the publications chosen for examination were confined to examples of population health publications produced either for, or by, health departments in each Australian State and Territory as well as relevant Commonwealth Departments. Graphs used in journal articles were not a component of this examination.

This section also documents the methodology used to identify the publications which formed part of the review, the classification system developed for the study, the subsequent cataloguing of graphs according to the classification system and the results of this part of the literature review. The methodology was not based on a census of all population health publications nor was it based on *random* samples of available publications. Therefore, no claims are made of a review based on representative data. However, all of the publications reviewed had similarities in the type of statistical concepts and measures that were presented in graphical form. If other Australian population health publications are typified by the sample used, it would be reasonable to assume that the output of this part of the review represents Australian population health publications in general.

2.2 Source material

A selection of literature published by Commonwealth, State and Territory health and health related departments was used to provide a source of graphs that were subsequently catalogued according to a classification system, designed as part of the study. The methods described below include the techniques used to collect population health publications, the basis of the classification system and the output of classification which was a summary of graph types discussed in this section

2.21 Collection of population health publications for review

The selection of population health publications from Commonwealth, State and Territory health and related departments was made by a study researcher contacting each government department and asking for a recommendation of documents for review. A web search was also conducted using key words and sites that were recommended by the government departments contacted as part of this study.

Initially, a contact list was drawn up of Commonwealth, State and Territory departments that might have or use population health reports. At the Commonwealth level the Australian Institute of Health and Welfare (AIHW) was the primary contact although the Australian Bureau of Statistics (ABS) also provided a publication for review. The health department was the primary contact within each State and Territory Government. One exception to the methodology of contacting government departments was the inclusion of the non-government Cancer Council of New South Wales. When each organisation was contacted, the study researcher asked to speak with a representative who would be aware of population health publications produced or available through that organisation. Once a suitable representative had been reached, the study researcher provided a brief description of the project and then requested suitable publications.

Where possible a bound publication was obtained, however there were instances where the researcher was directed to a web address to access a specific report which was then downloaded and printed. The preference of a bound version to a web version of reports was due to quality issues such as the use of colour in graphs, lost in downloaded files.

A bound report was obtained from NSW Health called: 'The health of the people of New South Wales, Report of the Chief Health Officer'. The Australian Capital Territory provided a bound report called: 'Health indicators in the ACT'. Two bound reports were obtained from the Health Department of Western Australia: 'Child and adolescent health in Western Australia: An overview' and 'Health measures for the population of Western Australia: Trends and comparisons'. The Tasmanian Department of Health and Human Services provided two bound publications: 'First results of the healthy communities survey 1998' and 'Demographic and health analysis of the Northern Region'. The Cancer Council of NSW provided a bound version of: 'Cancer in NSW, incidence and mortality 1999' and the Australian Bureau of Statistics publication: 'The health and welfare of Australia's Aboriginal and Torres Strait Islander peoples' was also bound.

Downloaded versions of publications were obtained after contact with a representative of the Victorian Public Health Division, Department of Human Resources who subsequently directed the researcher to the Department's web site where two reports were downloaded: 'Victorian burden of disease study: Mortality, 2000' and 'Victorian burden of disease study: Morbidity, 1999'. A continued search of the same web site provided the report titled: 'Mental health promotion benchmark survey 2001'. Recommendations for a web site were provided by the Territory Health Services for their reports: 'The health and welfare of Territorians' and 'From infancy to young adulthood: Mental health status in the Northern Territory'. The AIHW provided a web site for the download of the following reports: 'Cancer in Australia 1997, incidence and mortality data for 1997 and selected data for 1998 and 1999', 'Australia's health 2000: The seventh biennial health report of the Australian Institute of Health and Welfare' and 'Heart, stroke and vascular diseases, 2001'.

The complete list of reports included for the cataloguing component of the study follows:

Australian Bureau of Statistics (ABS) and the Australian Institute of Health and Welfare (AIHW) 2001, *The health and welfare of Australia's Aboriginal and Torres Strait Islander peoples*, ABS Cat. no. 4704.0, AIHW Cat. no. IHW 6, Canberra.

Australian Institute of Health and Welfare (AIHW), Australasian Association of Cancer Registries 2000, *Cancer in Australia 1997, Incidence and mortality data for 1997 and selected data for 1998 and 1999*, AIHW Cat. no. CAN 10, Canberra.

Australian Institute of Health and Welfare (AIHW) 2000, *Australia's health 2000: the seventh biennial health report of the Australian Institute of Health and Welfare*, AIHW, Canberra.

Australian Institute of Health and Welfare (AIHW) 2001, *Heart, stroke and vascular diseases, Australian facts 2001*, AIHW, National Heart Foundation of Australia, National Stroke Foundation of Australia (Cardiovascular Disease Series No. 14), Canberra.

Coats MS, Tracey EA 2001, *Cancer in NSW: Incidence and mortality 1999 featuring 30 years of cancer registration*, Cancer Council NSW, Sydney.

Condon JR, Warman G, Arnold L (editors) 2001, *The health and welfare of Territorians*, Epidemiology Branch, Territory Health Services, Darwin.

Department of Human Services 1999, *Victorian burden of disease study: Morbidity*, Public Health Division, Victorian Department of Human Services, Melbourne.

Department of Human Services 2000, *Victorian burden of disease study: Mortality*, Public Health Division, Victorian Department of Human Services, Melbourne.

d'Espaignet EJ, Kennedy K, Paterson BA and Measey ML 1998, *From infancy to young adulthood: Mental health status in the Northern Territory*, Territory Health Services, Darwin.

Kee C, Johanson G, White U, McConnell J 1998, *Health indicators in the ACT*, Epidemiology Unit, ACT Dept of Health and Community Care: Health series No. 13, ACT Government Printer, ACT.

NSW Health 2000, *The health of the people of NSW – Report of the Chief Health Officer 2000*, NSW Health Department, Sydney.

Parsons J, Wilson D and Scardigno A 2000, *The impact of diabetes in South Australia 2000*, South Australian Department of Human Services, South Australia.

Queensland Health 2001, H*ealth indicators for Queensland: Central Zone 2001*, Public Health Services, Queensland Health, Brisbane.

Ridolfo B, Sereafino S, Somerford P and Codde J 2000, *Health measures for the population of Western Australia: Trends and comparisons*, Health Department of Western Australia, Perth.

Silva DT, Palandri GA, Bower C, Gill L, Codde JP, Gee V and Stanley FJ 1999, *Child and adolescent health in Western Australia – an overview*, Health Department of Western Australia and TVW Telethon Institute for Child Health Research, Western Australia.

South Australian Department of Human Services 1999, *Interpersonal violence and abuse survey*, South Australian Department of Human Services, South Australia.

Tasmanian Department of Health and Human Services 1999, *First results of the healthy communities survey 1998*, Tasmanian Department of Health and Human Services, Research and Analysis Report, Tasmania.

Tasmanian Department of Health and Human Services 2000, *Demographic and health analysis of the Northern Region*, Tasmanian Department of Health and Human Services, Research and Analysis Report No. 4, Tasmania.

Taylor A, Dal Grande E and Wilson D 1996, *SA Country health survey: March-April 1996*, The Country Health Services Division, South Australia 1996

The Wallis Group 2001, *Mental health promotion benchmark survey*.

2.2.2 A graph classification system

Early in the study development, thought was given to the basis that would be used for classifying graphs into categories. Initially these thoughts focused on classifying by the type of graph; for example, a classification based on whether the graph was a bar, line, area or pie graph. However, during the development of this system, a high degree of overlap was noticed in the represented statistical 'measures'. For example, proportions were represented by pie, bar and line graphs. This overlap was believed to hinder the study because it did not assist with obtaining a broader understanding of the type of graphs used in population health publications. Therefore, a second classification option was developed: differentiation based on the statistical 'measure' used in the graph. Overlap between categories was reduced but not eliminated. The overlap was mainly in the style of graph used to relate the statistical measures. For example, bar graphs represented simple counts and were used to represent rates and proportions. Despite this issue, the second system was seen as an improvement over the first and after discussions with the Progress Working Group (PWG) it was adopted as a basis for the catalogue system. This classification framework is in Appendix 1.

2.2.3 Definitions of measures used for classifying graphs

The measures selected for the study (frequencies, rates, proportions, ratios, risk, central tendency and life expectancy) were not discrete and several could have been condensed into a single measure. However, there was a trade-off to collapsing: some basic framework for classification was needed to order the graph examples collected in the reviewed publications. This classification was an artificial construct to simplify the task of grouping like graphs together and the use of 'measure' was an attempt to introduce some objectivity into the classification. Nevertheless, a level of subjectivity was still required, meaning that another researcher may have allocated graphs differently. There are two reasons why this is not believed to be a weakness of the study. First, the raison d'être of the classification was primarily to allow sorting of graphs into groups which, in turn, might simplify the broader task of understanding the type of graphs used in population health reports. Second, the study did not aim to refine definitions of the identified 'measures'. They were merely a convenient method of grouping like with like. Another method of classification was considered: grouping according to type of graph e.g. line, bar, pie etc. Though discussed above, this was abandoned because the resulting groups were thought to have *too much* overlap to be useful.

Therefore, while classification of graphs according to 'measure' is not perfect and is not without overlap, it was chosen because it provided a better solution than the identified alternative.

The definition of the statistical measures used in this study were:

A **rate** was defined as a ratio representing *relative changes (actual or potential) in two quantities* (Last *et al.* 1995, p. 140).

A **proportion** is a type of ratio where the numerator is included in the denominator. The proportion is then the expression of the part to the whole (Last *et al.* 1995).

A **ratio** is the division of one quantity by another (Last *et al*. 1995). The more refined version used in this study was that the two quantities are *distinct and separate, neither being included in the other*. Ratios can be expressed as percentages and in *these cases, unlike the special case of a proportion, the value may exceed 100* (Last *et al.* 1995, p. 141).

Life expectancy is the *average number of years an individual of given age is expected to live if current mortality rates continue to apply. … Life expectancy is a hypothetical measure and indicator of current health and mortality conditions* (Last *et al.* 1995, p. 59).

Risk is the probability of an event occurring (Last *et al*. 1995).

Frequency is the number of times an event or disease occurs. It does not distinguish between incidence and prevalence (Last *et al.* 1995).

Central tendency describes the typical or average score. Three measures of central tendency are the mode, mean and median. The mode is the most frequently occurring score in a distribution, a mean is the arithmetic average of all scores and the median is the middle score in a distribution where the scores have been arranged from highest to lowest (Graziano *et al.* 1989).

2.2.4 Characteristics observed during the classification of graphs

The classification system ultimately recorded twenty characteristics of graph design. These are identified and discussed in Appendix 1. As noted, the main characteristic used for classifying of graphs into one of seven sub-groups was the 'measure' displayed in the graph; that is, whether the graph reflected frequencies, rates, proportions, ratios, risk, central tendency or life expectancy.

One of the examined characteristics of the graphs sourced from the reviewed publications was the type of graph used to present the data. Bar, column, line, pyramid, pie and dot graphs were found. A definition of each graph type follows:

Bar graph

Bar graphs expressed the data as a horizontal bar against perpendicular axes. Schmid (1983, p. 39) identified and described multiple types of bar chart that were also found in the reviewed health publications. Starting with a *simple bar* graph where horizontal bars compare two or more coordinate items: the comparison is made on direct linear values, as the length of the bar reflects the value of each category. A more complex *subdivided bar* graph is one where each bar is divided into segments and the scaled value of each bar segment is shown as an absolute value. A *subdivided one hundred per cent bar* graph is where each bar is divided into segments and the total for each bar is one hundred per cent. In all bar graphs the length of the bar is determined by the value or amount in each category.

Column graph

Column graphs are similar to bar graphs except that the data is expressed in a vertical representation against perpendicular axes. Schmid (1983, p. 43) also identified different types of column charts. Starting with a *simple column* chart that makes a comparison with two or more coordinate items using vertical columns: again the comparison is made on direct linear values as the length of the column reflects the value of each category. A *connected column* graph is also a histogram. A *subdivided column* graph is one where the scale value of each column segment is shown as an absolute value. A subdivided one hundred per cent column graph is one where each column has two or more segments where the total for each column is one hundred per cent. In all column graphs the length of the bar is determined by the value or amount in each category.

Line graph

A line graph *is derived by plotting figures in relation to two axes formed by the intersection of two perpendicular lines* Schmid (1983, p. 17). The horizontal line is the 'x' axis and the vertical line, the 'y' axis. The point of intersection is called the "origin" and the scales are arranged in both directions, horizontally and vertically. Measurements to the right and above are positive whereas measurements to the left and below the origin are negative (Schmid 1983).

Pyramid graph

Also called a paired bar chart, pyramid graphs are a two-way chart where the horizontal axis is positive in both directions from a central point. The left and right measurements from the central point are frequently used to compare age structures.

Pie graph

A pie graph is a circle divided into segments; each segment represents a sub-category of the outcome variable. Although they do not have to be labelled as a proportion, each segment represents a proportional amount of the total. Therefore the sum of all segments total to one hundred per cent.

Dot graph

Dot graphs or plots are similar to line graphs except that the plotted figures are represented by a dot, or other symbol, which is in relation to two axes. The axes are formed by the intersection of two perpendicular lines: the 'x' axis and the 'y' axis. As with line graphs, measurements to the right and above are positive whereas measurements to the left and below the origin are negative.

2.2.5 Cataloguing graphs used in selected population health publications

The publications identified in Section 2.2.1 were used as the source of graphs, from which examples were extracted and included in this document. The examples' characteristics were catalogued according to the classification system outlined in Section 2.2.2.

2.2.6 Cataloguing graphs using the criteria of 'measurement'

Seven main 'measures' were used for cataloguing graphs in Australian health publications: frequencies, rates, proportions, ratios, central tendency (means and medians), life expectancy and risk. The category of risk was included although only one of the reviewed publications used this measure. However, as nineteen examples of this measure were found it was considered a valuable inclusion. Graphs in each of the reviewed publications were classified into these groups and then counted to obtain an indication of frequency of usage. As a quality control measure, a second researcher independently counted and classified the graphs. Differences between the first and second researcher were discussed and allocations of graphs into final categories were made by mutual consent.

The cataloguing of graphs according to these measures formed the basis of the following sections. The frequency of graphs in each of the seven measures is illustrated in *Figure 1.*

2.3 Graphs displaying rates

Overall comment on the measure

Graphs showing the measure of rates included examples of population incidence rates, prevalence rates, crude, age specific and age standardised rates. The frequency of using graphs displaying rates was in the 'high' range; that is more than one hundred and one examples were found. The general aim of these graphs was to compare how fast an event was occurring in a population in relation to one or more independent variables. Rates were used to represent prevalence (existing cases) and incidence (new cases). If the rate is measuring incidence, the numerator is the *number of new* cases occurring in the population during a specified period divided by the people who are at risk of becoming a case. If the rate is measuring prevalence, the numerator is the *number of cases present* in the population at a specified time divided by the number of persons in the population at that specified time (Gordis 2000).

Type of graph used

The graphs displaying rates used bar, line, pie and area graphs. Examples were also found of confidence intervals around point estimates: see the bar graph in *Figure 2*. The line graphs often used point markers to represent the point estimate at each interval that were joined by either solid or dashed lines (see *Figure 3*). Although area graphs in the reviewed publications were not often used to display any statistical measure, an example was found of a stacked area graph displaying rates.

Statistical concepts covered

Rates are a form of ratio with an additional element: time. Rates can be categorised into crude rates, specific rates and standardised rates. Regardless of the category, all rates relate to a time period although examples were found where the period was not explicitly mentioned in any of the text supporting the graph. Crude rates use the population as a whole without any sub-classifications. With specific rates, some subdivision of the population has occurred, such as division by age, sex or race. Standardised rates provide a summary figure for comparison by removing the impact of different

Figure 1. Count of graphs found in the reviewed publications displaying each of the statistical measures used for cataloguing

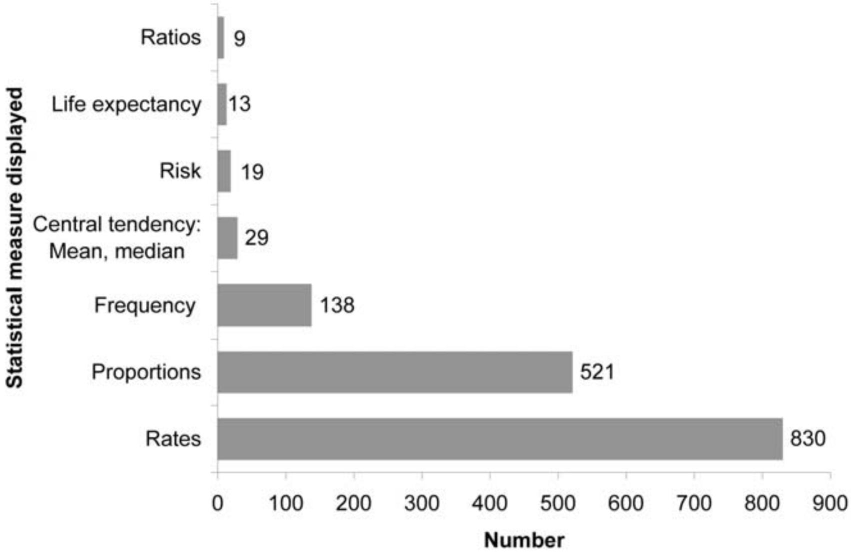

distributions within populations and are frequently used when comparing populations with different age structures (Gordis 2000). Confidence intervals for standardised rates were also found (see *Figure 2*).

Type of comparison

Graphs displaying rates often compared the outcome variable to two or more independent variables and/or two or more sub-categories of the outcome variable. For example, in *Figure 2* the comparison is between two independent variables: rates of new cases of melanoma (per 1,000 population) between males and females and in each State and Territory. An example of comparisons between sub-categories is shown in *Figure 6* where a comparison can be made between categories of the outcome variable (cause of death) as well as comparisons between independent variables of gender and rurality.

Comment on text interpretations

Text interpretations of graphs displaying rates were not found in any examples; that is, readers were not instructed on how to interpret the graphs. However, the text frequently referred to information displayed in the graph usually by stating the main point or trends that could be inferred from the graph.

Consistency between the sampled health publications

The graphs were not consistent between publications. Differences mainly occurred in the presentation of data (e.g. bar, pie and scatter plot graphs).

Other notes

Of all measures represented in graphs in the reviewed publication, those displaying rates were the most frequently used, despite some publications not using rates at all. Generally, many graph styles were used to display rates.

Some of the more difficult graphs to interpret were those which used multiple categories of the outcome variable. For example, in *Figure 6* four categories were compared in a stacked bar graph. In this style of graph Kosslyn (1994) recommends that the independent variable with least variation be at the base of the bar so that comparisons are easier for the reader. Additionally, these examples fall into the 'too much information' basket Kosslyn (1985) warns against, because the reader must memorise the key before interpreting the graph. Earlier work by Shultz (1961) also showed that too much information degraded the reader's abililty to decode the graph.

As incidence is a time based measure, the reference period should always be identified. For example, in *Figure 2* the reference period is unclear; it might be incidence per 100,000 population per year or incidence per 100,000 population for the five year period 1993 to 1997.

Examples

Figure 2. Example of graphs displaying rates: Sourced from *'Cancer in Australia 1997'*, Australian Institute of Health and Welfare, Australian Association of Cancer Registries, 2000, p. 19

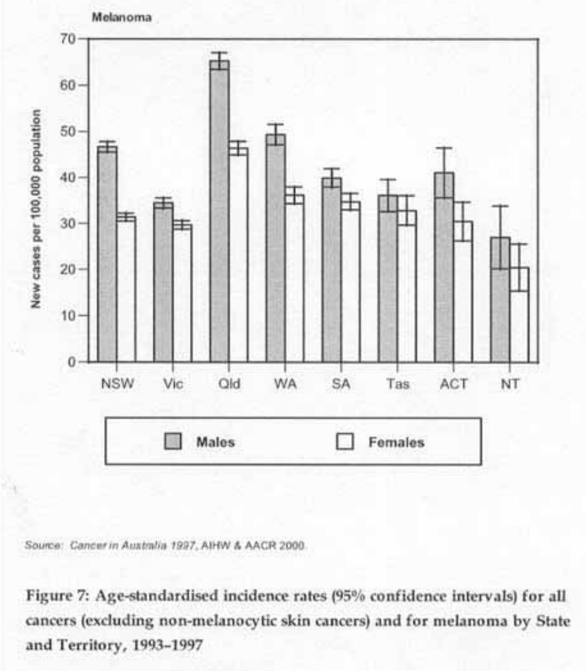

Reproduced with permission from the Australian Institute of Health and Welfare.

Figure 3. Example of a graph displaying rates: Sourced from *'Health Indicators for Queensland: Central Zone'*, Public Health Services, Queensland Health, 2001, p. 50

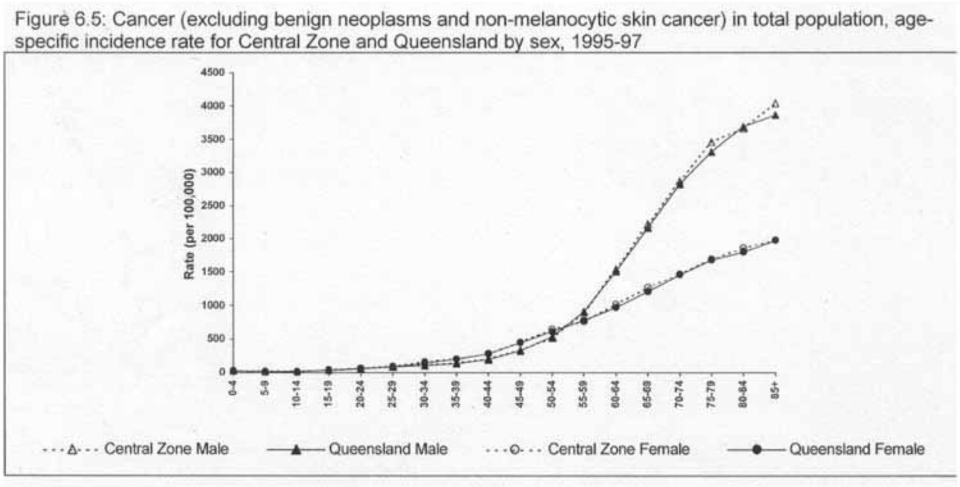

Graph copyright State of Queensland (Queensland Health) 2001. Reproduced with permission.

Figure 4. Example of a graph displaying rates: Sourced from *'Demographic and Health Analysis of the Northern Regions'*, Report No. 4, Tasmanian Department of Health and Human Services, 2000, p. 10

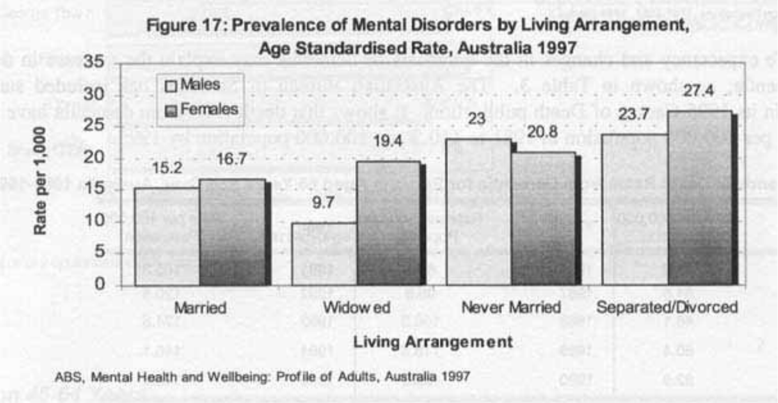

Reproduced with permission from the Tasmanian Department of Health and Human Services.

Figure 5. Example of a graph displaying rates: Sourced from *'From Infancy to Young Adulthood, Health Status in the Northern Territory'*, Territory Health Services, 1998, p. 46

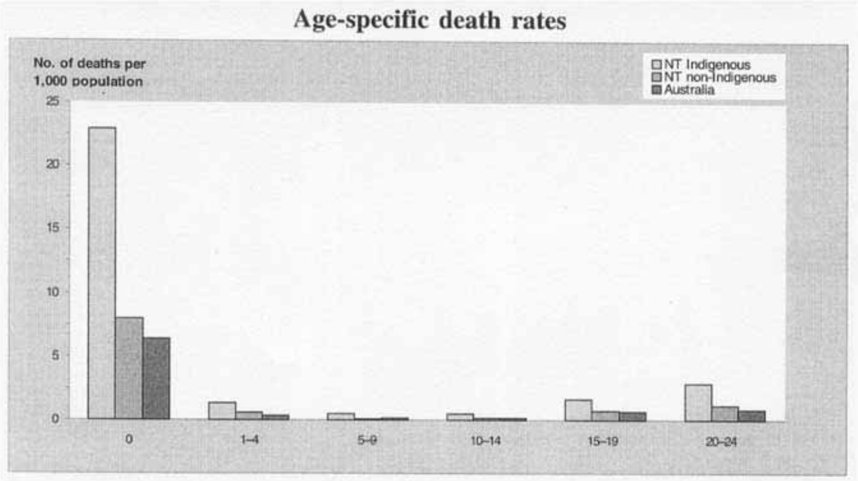

Age group (years)	NT Indigenous	NT non-Indigenous	Australia
	No. of deaths per 1,000 population		
0	22.5	7.1	6.4
1–4	1.3	0.6	0.4
5–9	0.5	0.1	0.2
10–14	0.5	0.2	0.2
15–19	1.6	0.7	0.6
20–24	2.9	1.1	0.8

Note: 1. Data presented here are for the years 1991–95.
2. All rates expressed as the number of deaths per thousand population, except for the infant mortality rate (at age 0) where the rate measures the number of infant deaths per thousand livebirths.

Reproduced with permission from the Northern Territory Department of Health and Community Services.

Figure 6. Example of a graph displaying rates: Sourced from *'Victorian burden of disease study, Mortality'*, Victorian Department of Human Services, 2000, p. 22

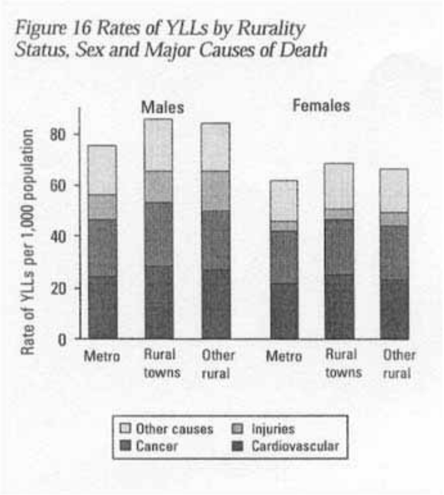

Reproduced with permission from the Victorian Department of Human Services.

2.4 Graphs displaying proportions

Graphs displaying proportions all displayed the outcome variable as a proportion of the total population.

Overall comment on the measure

The frequency of graphs displaying proportions in the reviewed publications was 'high', that is more than one hundred and one examples were found. These graphs all expressed proportions as percentages and could be used to represent probability. The general aim of these graphs was to describe how the proportion of the population exhibiting some characteristic (the outcome variable) varied in relation to the independent variable(s). Some graphs showing proportions were also used to represent the prevalence of an event or disease, relative frequency and probability.

Type of graph used

The graphs displaying proportions used bar, line, pie and scatter plot layouts. Variations in the design of bar graphs were identified. Population pyramids were commonly used with demographic data to show the distribution of population over all ages and by sex. Sub-groups of pie graphs were found: individual pieces of an exploded pie graph were plotted as a separate pie. As shown in *Figure 11* an exploded piece of the pie graph was associated with a single bar graph, which, representing the single pie segment, totalled to one hundred per cent. In *Figure 12* a dot graph was used with upper and lower confidence intervals. In *Figure 10* another variation of a dot graph was used: solid and empty dots were used to represent males and females and each point estimate was plotted back to the 'y' axis with a dotted line.

Statistical concepts covered

Graphs displaying proportions showed the frequency of a variable as a proportion of some 'total' and were used to display *relative frequency*, which could also be expressed as a *probability* or *prevalence*. The point estimates were sometimes plotted with confidence intervals.

Type of comparison

The graphs displaying proportions showed the frequency of an outcome variable as a proportion of a total; the outcome was often displayed against categories of the independent variable. For example, in *Figure 8* the outcome variable being 'prevalence of sun protection', was related to (i) frequency of behaviour and (ii) age. In this example, the use of a stacked bar graph where each column totalled to one hundred per cent allowed the comparison of *relative frequency* of sun protection behaviours within each age category and between each age category. In *Figure 9* the comparisons were between the outcome variable of 'prevalence of risk factor' and the presence or absence of diabetes.

Comment on text interpretations (if used)

Text interpretations were not found in the examples. However, as with graphs displaying all of the measures, the document's text often referred to information displayed in the graph although this referencing was not always explicit. For example, in *Figure 7* the surrounding text made reference to the information displayed in the graph (waist circumference) but did not explicitly refer to a figure number for the reader. The graph was located next to the paragraph describing the proportion of adults with a given waist size. This lack of explicit referencing was not common as the usual methodology was to describe the findings and cite the figure number displaying the results.

Consistency between the health publications sampled

The graphs were not generally consistent between publications. As with other types of measure, differences occurred in the presentation of data (eg bar, pie, dot and scatter plot graphs), the inclusion of gridlines, font size and explanations of the main points represented in the graph in the surrounding text.

Other notes

Generally, many graph styles (bar, pie, line etc.) were used to display proportions. The graph shown in *Figure 11* was notable because it combined two graph styles: a pie graph to represent neoplasms as a proportion of 'other disorders' and a single bar graph to represent sub-types of neoplasm. This bar graph also used labels to identify the numeric proportion of each sub-type of neoplasm as a percentage of 'total neoplasms'.

Examples

Figure 7. Example of a graph displaying proportions: Sourced from *'Heart, stroke and vascular diseases, Australian facts 2001*, Australian Institute of Health and Welfare, National Heart Foundation of Australia, National Stroke Foundation of Australia', 2001, p. 59

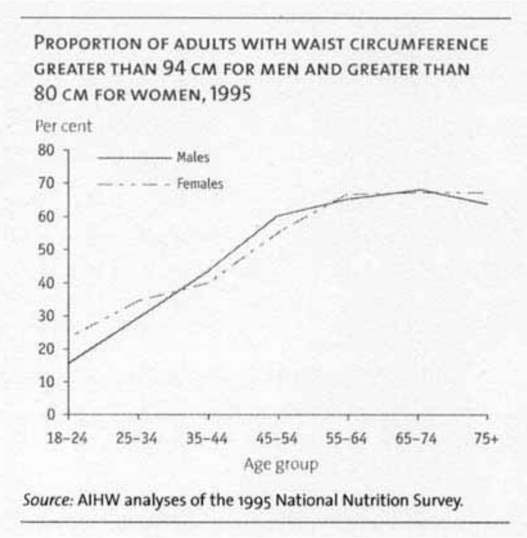

Reproduced with permission from the Australian Institute of Health and Welfare.

Figure 8. Example of a graph displaying proportions:
Sourced from *'Australia's Health 2000: the seventh biennial health report of the Australian Institute of Health and Welfare'*, Australian Institute of Health and Welfare, 2000, p. 156

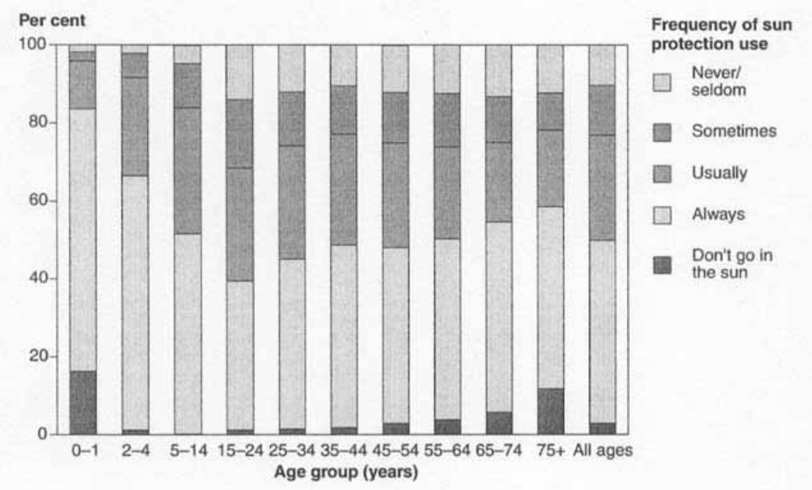

Note: See Box 3.4 for comment on the accuracy of the estimates.
Source: 1995 National Health Survey.

Figure 3.8: Use of sun protection measures, 1995

Reproduced with permission from the Australian Institute of Health and Welfare.

Figure 9. Example of a graph displaying proportions:
Sourced from *'The impact of Diabetes in South Australia 2000'*, South Australian Department of Human Services, South Australia, 2000, p. 10

Figure 3: Prevalence of risk factors for the development of diabetes

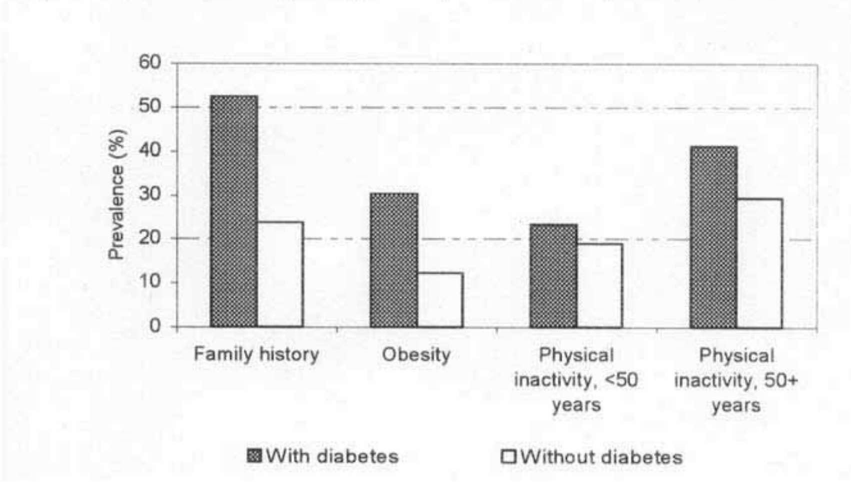

Reproduced with permission from the South Australian Department of Human Services.

Figure 10. Example of a graph displaying proportions:
Sourced from *'The Health and Welfare of Australia's Aboriginal and Torres Strait Islander Peoples'*, ABS Cat. no. 4704.0, AIHW Cat. no. IHW 6, Canberra 2001 (www.abs.gov.au), p. 82

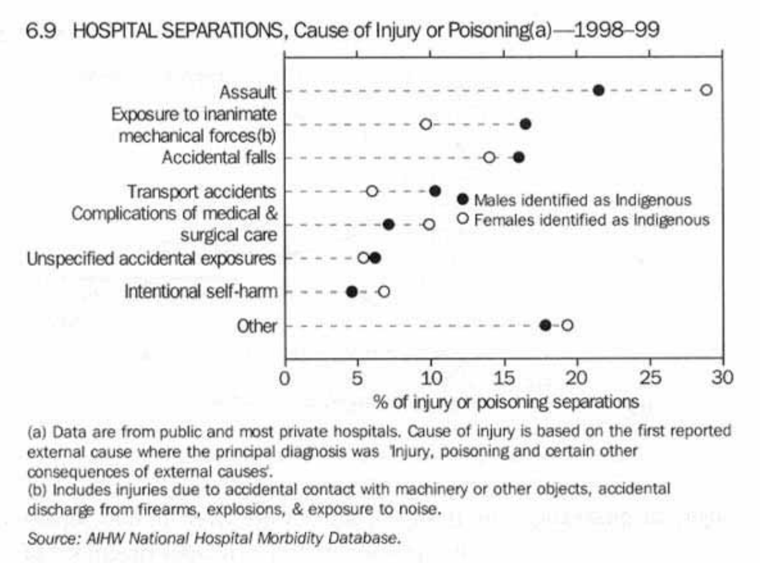

Reproduced with permission from the Australian Institute of Health and Welfare and the Australian Bureau of Statistics.

Figure 11. Example of a graph displaying proportions:
Sourced from *'Health indicators in the ACT'*, Epidemiology Unit, ACT Dept of Health and Community Care: Health series No. 13, ACT Government Printer, 1999, p. 50

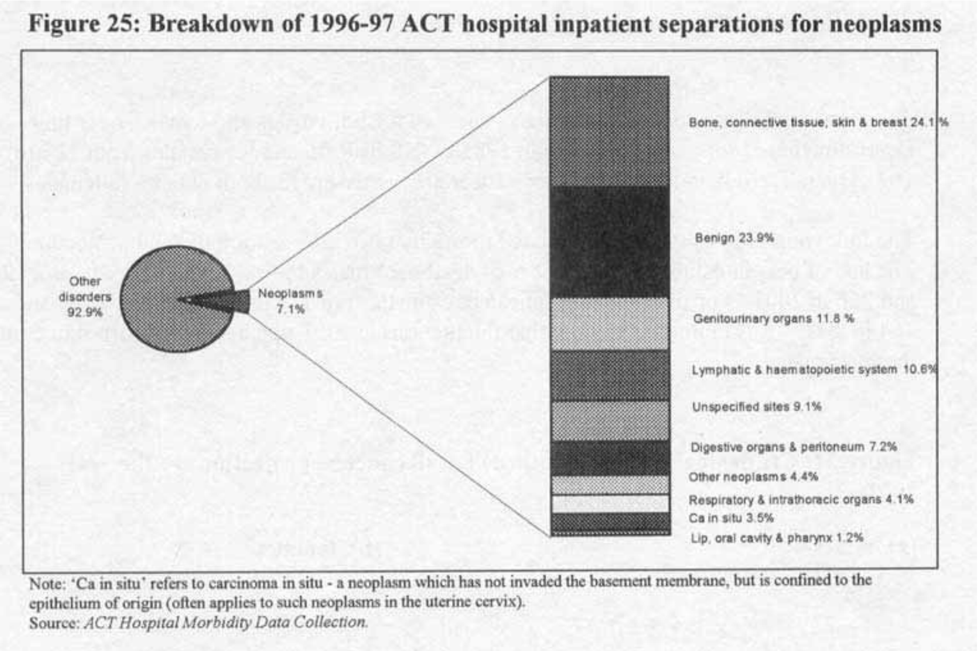

Reproduced with permission from the ACT Dept of Health and Community Care.

Figure 12. Example of a graph displaying proportions:
Sourced from *'The health of the people of NSW – Report of the Chief Health Officer'*, NSW Department of Health, Sydney, 2000, p. 89

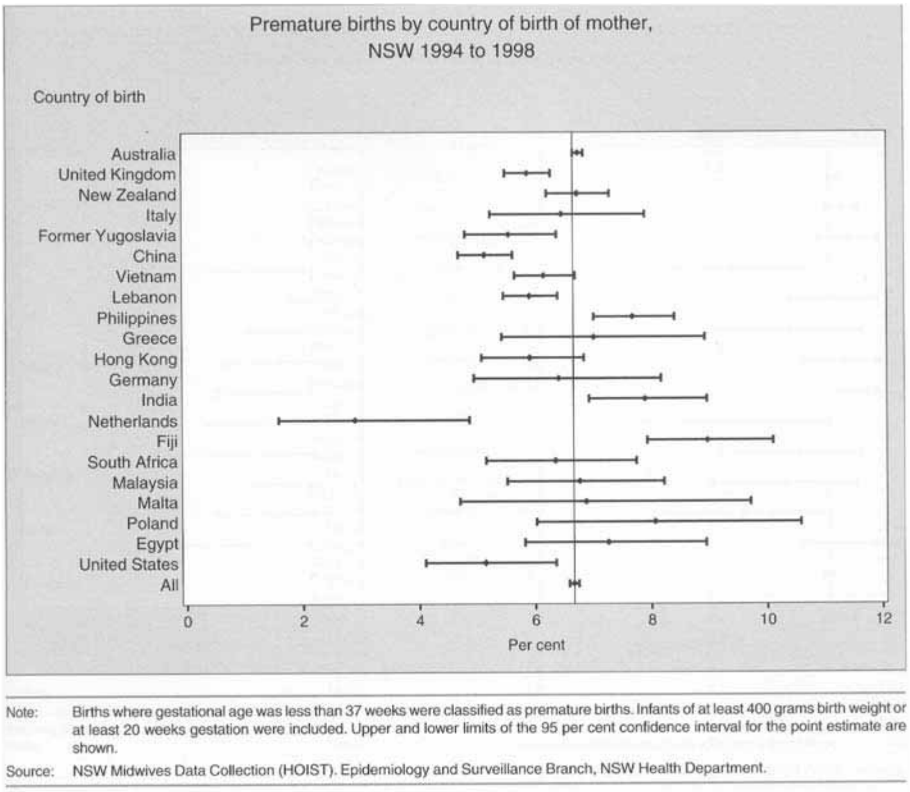

Reproduced with permission from the NSW Department of Health.

Figure 13. Example of a graph displaying proportions:
Sourced from *'The health and welfare of Territorians'*, Epidemiology Branch, Territory Health Services, 2001, p. 2

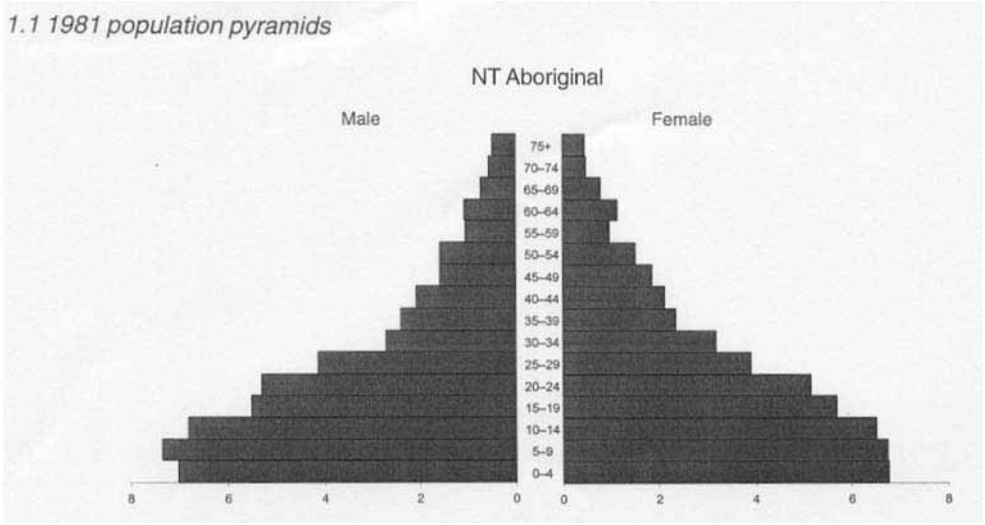

Reproduced with permission from the Northern Territory Department of Health and Community Services.

2.5 Graphs displaying frequency

Overall comment on the measure

Frequency graphs show counts of data at a single point in time. The use of these graphs in the reviewed publications was 'medium', that is between thirty-one and one hundred examples were found. In these graphs the outcome variable was usually compared to categories of the independent variable.

Type of graph used

Bar graphs were mainly used for showing frequencies. Sub-groups of this type of graph were found; examples included: the use of vertical or horizontal bars, pyramid and stacked bar graphs. One publication 'The health of the people of NSW, Report of the Chief Health Officer, 2000' exclusively used horizontal bars for frequencies even in cases where there were few categories, and therefore labels, required on the 'y' axis (see *Figure 14*). The use of horizontal bars allows more space for labels when there are many categories of the independent variable. This standardised formatting procedure reduces the reader's effort to decode the data.

Statistical concepts covered

Frequency graphs represented simple counts of the outcome variable either at a single point in time or in a time series.

Type of comparison

The comparisons were between counts of the outcome variable and, usually, categories of the independent variable. Time was used as the independent variable (as in time series data).

Comment on text interpretations (if used)

Text interpretations of frequency graphs were not found in any of the reviewed publications. For *Figure 16* the text interpretation identified different scales on the 'x' axis between population pyramids. Some graphs were associated with tabular information that duplicated the information in the graph. The additional information provided in these tables were usually cells containing the 'total' for the variable. In *Figure 14*, the male and female totals used in the frequency graph were provided in an adjoining table, which also provided time series data (1992 to 1998), a rate of hepatitis notifications per million people and annual age adjusted figures.

Some graphs used abbreviated terminology that required additional reader effort for decoding. For example, in *Figure 15* the concepts of DALY (Disability Adjusted Life Years) , YLD (Years Lost due to Disability) and YLL (Years of Life Lost) were described in the introduction to the report but they were not repeated near the placement of the graph.

Consistency between the health publications sampled

The graphs were not consistent between publications. Differences occurred in the presentation of clustered data (e.g. pyramid style (*Figure 16*) v. stacked bars (*Figure 15*)), the use of vertical or horizontal bars, placement of legends, the use of series labels, the inclusion of gridlines (used by only one publication) and font size.

Other notes

Bar graphs were used extensively in 'The health of the people of NSW, Report of the Chief Health Officer, 2000' although their use was not restricted to frequency graphs. The formatting was standardised throughout the publication: horizontal bars were always used with the numeric value plotted on the 'x' axis. Additional independent variables (e.g. gender) were plotted using a pyramid style graph, with central labels.

'The health of the people of NSW, Report of the Chief Health Officer, 2000' also used tables under the graph that sometimes repeated the graph data and sometimes extended the data by providing totals (see *Figure 14*). While this might be repetitious, Mahon (1977) has commented that this practice might have appeal: particularly if the graph will have an audience who might be better able to interpret a table and noted that there is no reason not to use both a graph and a table, if it helps.

Examples

Figure 14. Example of a graph displaying frequency:
Sourced from *'The health of the people of New South Wales – Report of the Chief Health Officer'*, NSW Health, 2000, p. 257

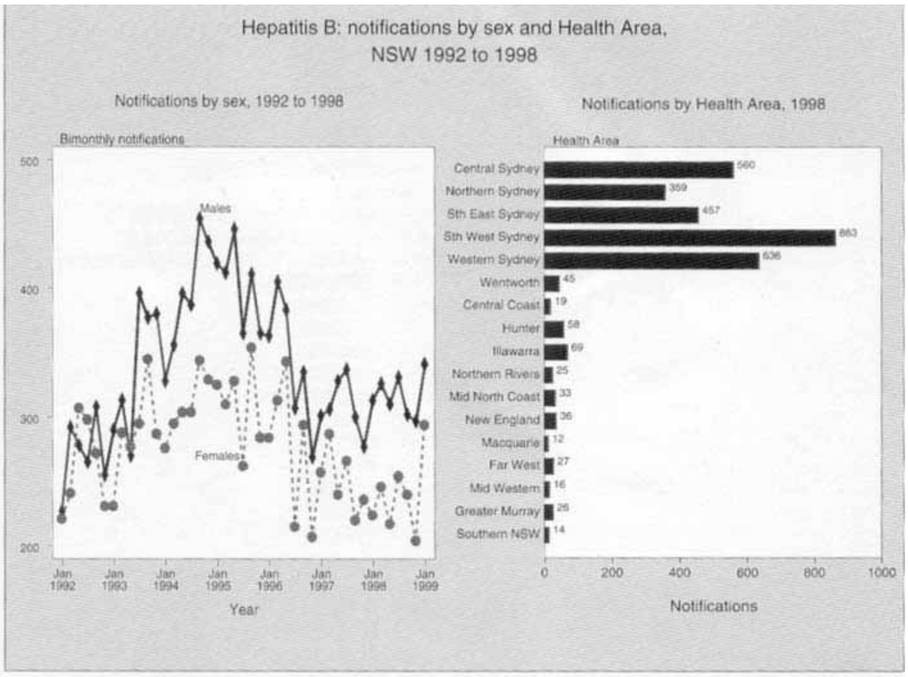

Notifications	Sex	1992	1993	1994	1995	1996	1997	1998
Number	Males	1625	2028	2357	2418	2062	1849	1877
	Females	1570	1724	1851	1864	1655	1504	1381
	Persons	3195	3752	4208	4282	3717	3353	3258
Rate per million	Males	545.4	676.2	779.1	793.5	668.1	594.2	597.9
	Females	537.1	586.2	626.5	623.1	547.2	492.1	444.9
	Persons	540.6	630.7	702.0	707.7	607.1	542.8	521.1

Note: Data as at 8 December 1999. Rates were age-adjusted using the Australian population as at 30 June 1991.
Source: NSW Notifiable Diseases Database and ABS population estimates (HOIST). Epidemiology and Surveillance Branch, NSW Health Department.

Reproduced with permission from the NSW Department of Health.

Figure 15. Example of a graph displaying frequency:
Sourced from *'Victorian Burden of Disease Study: Morbidity' Public Health Division'*, Victorian Department of Human Services, 1999, p. 74

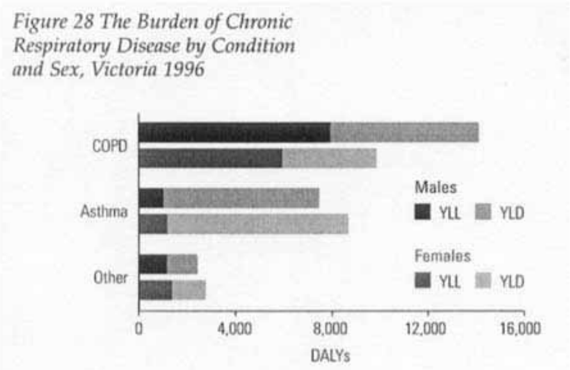

Reproduced with permission from the Victorian Department of Human Services.

Figure 16. Example of graphs displaying frequency:
Sourced from *'Health Indicators for Queensland: Central Zone'*,
Public Health Services, Queensland Health, 2001 p. 14

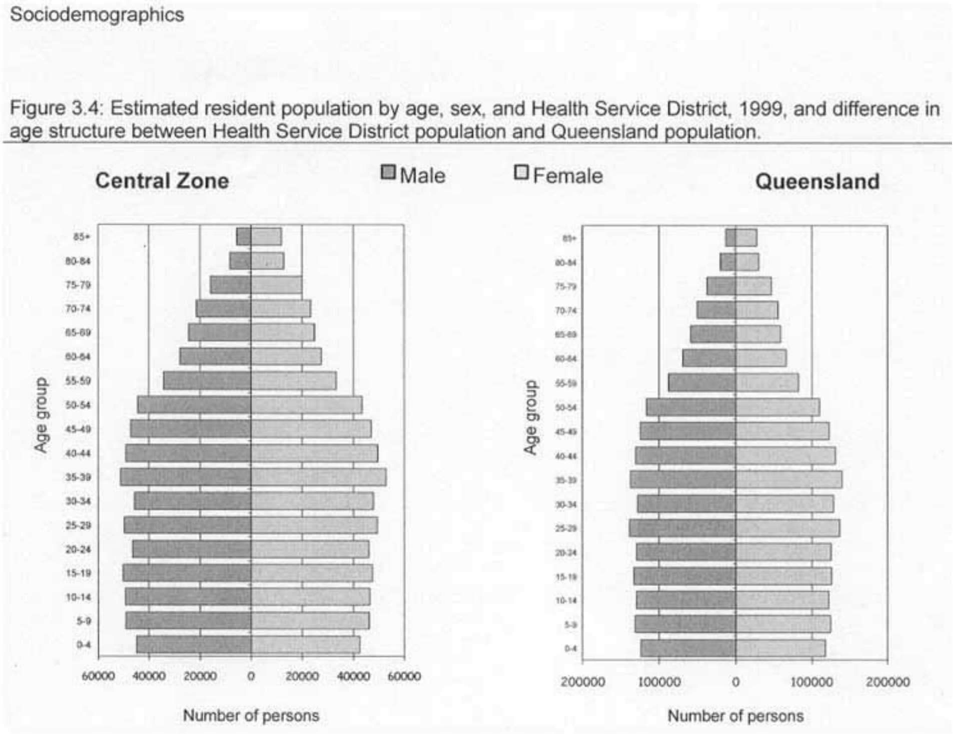

Graph copyright State of Queensland (Queensland Health) 2001. Reproduced with permission.

2.6 Graphs displaying central tendency

Overall comment of the measure

Graphs displaying measures of central tendency used either means or medians to represent point estimates. No examples were found of graphs displaying a mode. Some of the graphs included ninety-five per cent confidence intervals while one example displayed only the ninety-five per cent confidence interval without any point estimate. The frequency of use of these graphs in the reviewed publications was ‘medium’, that is between thirty-one and one hundred examples were found.

Type of graph used

The graphs displaying proportions used bar, line, and dot graphs. One of the examples displaying means used a dot plot that also included the ninety-five per cent confidence interval (*Figure 20*). The bar graph used in *Figure 20* also incorporated a ninety-five per cent confidence interval on *some* of the graphed point estimates. An interval was provided for indigenous males and females, however the confidence interval was not provided for non-indigenous females or non-indigenous males in the State. Only the upper confidence interval was provided for non-indigenous males in Central Zone.

Statistical concepts covered

Graphs displaying a measure of central tendency commonly used a mean or median to represent the highest frequency of a value in a distribution. No examples were found of modes.

Type of comparison

The examples of graphs displaying measures of central tendency made comparisons between the outcome variable and independent variables, which included time, location, race, gender and age group.

A comparison based on time was shown in *Figure 17* where the outcome variable (average length of stay in ACT hospitals) was displayed using the mean for each year. The resulting time series provided a trend over a nine-year period.

A comparison between geographic area and disease characteristic was shown in *Figure 18*. In this example the comparison was between the independent variable (location) and between two outcome variables (mean DMFT and decay free proportion). The comparison between the two outcome variables (disease characteristics and status) was achieved by the use of two graphs sharing the same vertical axis. The ordering of the point estimates appeared to be based on the ordering of health areas, starting with Sydney and followed by rural areas.

A comparison based on independent variables of age group, time and gender was shown in *Figure 19*. In this example the multiple comparisons were made by plotting age group on the ‘x’ axis and using separate time series for males and females and the two years of data (1980 and 1983).

A comparison between an outcome variable of median age of death, and race, gender and location was shown in *Figure 20*. In this example the ‘x’ axis plotted race by categories of gender. The vertical bars represented the geographic areas of Queensland and Central Zone.

Comment on text interpretations (if used)

Text interpretations of graphs displaying measures of central tendency were not found in any of the examples, however the text often referred to information displayed in the graph. The text interpretation of the graph in *Figure 17* referred to the graph and provided some exact point estimates (average number of days of hospital stay).

Consistency between the health publications sampled

The graphs were not consistent between publications. Differences occurred in the presentation of data (eg bar, line and scatter plot graphs) and the ordering of point estimates on the graph. The information included in titles tended to be similar, although one example omitted the inclusion of the reference period, place and population.

Other notes

Graphs displaying central tendency used means and medians that were sometimes accompanied by a ninety-five per cent confidence interval. Mode was not used as a measure of central tendency.

Examples

Figure 17. Example of a graph displaying a measure of central tendency: means. Sourced from *'Health indicators in the ACT'*, Epidemiology Unit, ACT Department of Health and Community Care: Health series No. 13, ACT Government Printer, 1999, p. 30.

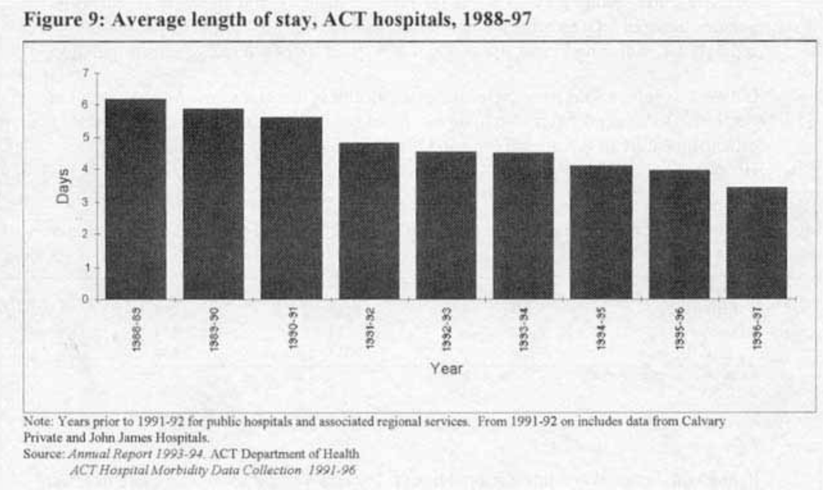

Reproduced with permission from the ACT Department of Health and Community Care.

Figure 18. Example of a graph displaying a measure of central tendency: means. Sourced from *'The health of the people of New South Wales, Report of the Chief Health Officer'*, NSW Health, 2000, p. 236 (left hand graph only)

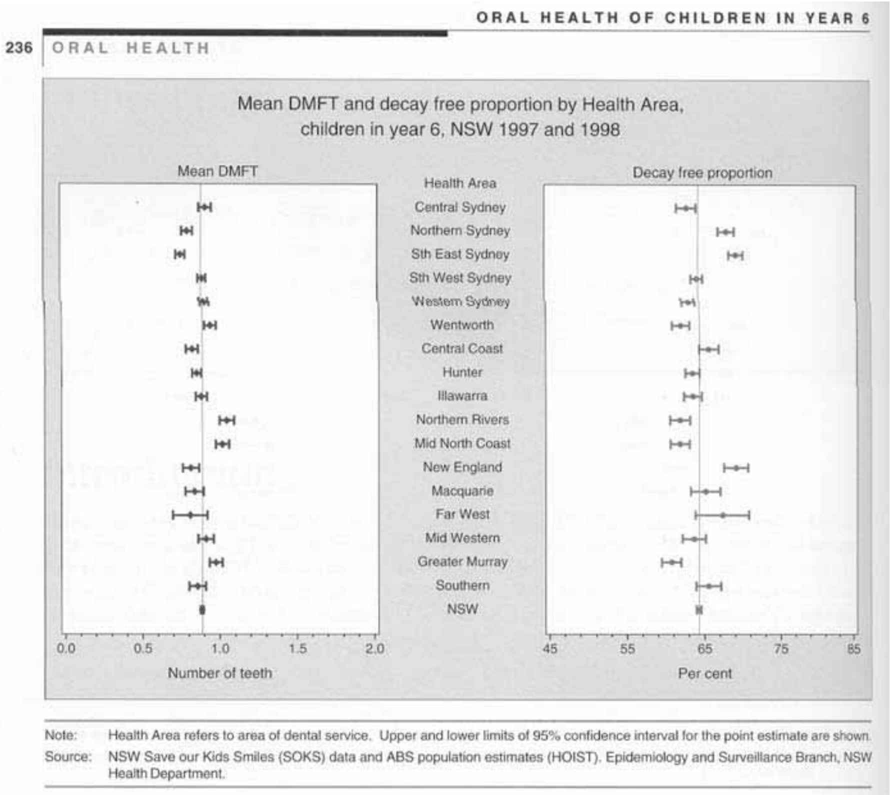

Reproduced with permission from the NSW Department of Health.

Figure 19. Example of a graph displaying a measure of central tendency: means.
Sourced from *'Health Measures for the Population of Western Australia: Trends and comparisons'*, Health Department of Western Australia, 2000, p. 26

Figure 17: Mean diastolic blood pressure in 25-64 year olds

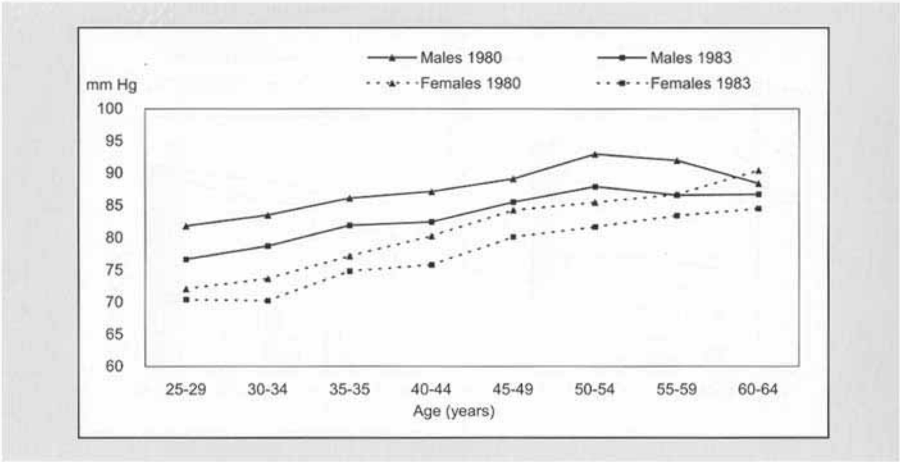

Western Australia	25-29	30-34	35-35	40-44	45-49	50-54	55-59	60-64
Males - 1980	81.85	83.48	86.12	87.13	89.11	92.94	91.96	88.40
Males - 1983	76.68	78.72	81.93	82.44	85.49	87.90	86.59	86.73
Females - 1980	72.08	73.67	77.17	80.28	84.25	85.47	86.69	90.43
Females - 1983	70.40	70.25	74.87	75.81	80.13	81.69	83.42	84.51

Reproduced with permission from the Western Australia Department of Health.

Figure 20. Example of a graph displaying a measure of central tendency: medians.
Sourced from *'Health Indicators for Queensland: Central Zone'*, Public Health Services, Queensland Health, 2001, p. 39

Figure 5.5: All causes, median age at death (95%CI) in Indigenous and non-Indigenous population in Central Zone and Queensland, 1996-98

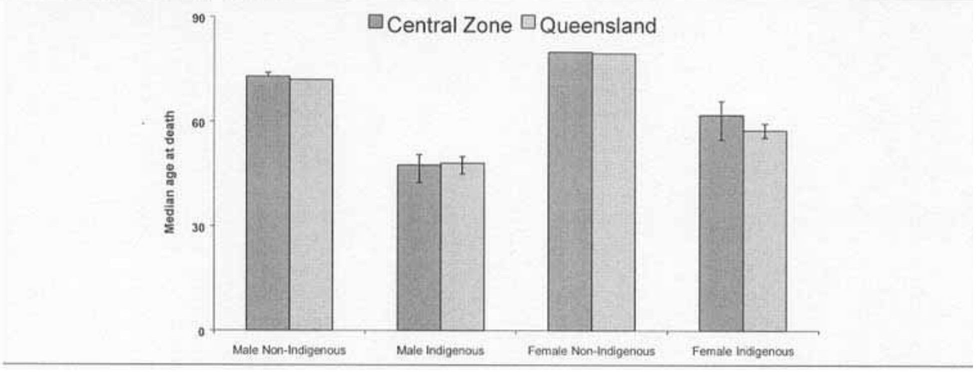

Graph copyright State of Queensland (Queensland Health) 2001. Reproduced with permission.

2.7 Graphs displaying ratios

Graphs displaying ratios used a single figure to represent the relationship between the proportional occurrence of some event between two groups. In the reviewed publications, the 'two groups' tended to be males and females. However, an example was found of a ratio that compared mortality to incidence. In this example, the ratios were displayed separately for males and females so that a gender comparison could be made. Graphs displaying ratios were not frequently used and as only seven examples were found, their use was categorised as 'low'. Graphs displaying ratios in the reviewed publications generally used line graphs although one example was found of a bar graph.

Graphs displaying ratios used a single series to represent a comparison between two groups or variables. A ratio is calculated by dividing the numerator by the denominator and, therefore, interpretation of the ratio is dependent upon knowing which variable is the numerator and which is the denominator. The expression of the ratio was generally as a number although an example was found of a ratio expressed as a percentage.

Type of graph used

The graphs displaying ratios generally used line graphs (with multiple lines), although one example used a bar graph. Usually the number of lines was less than four, however, one graph, *Figure 22*, displayed seven series of ratios. All of the examples that were line graphs used markers for each point estimate.

Statistical concepts covered

Graphs displaying a measure of ratios used a numeral to show the division of two variables and one example was found of the division expressed as a percentage. The use of a ratio meant that the relative size of two variables could be displayed as a single variable and although each ratio can only represent two variables, the graphs in the reviewed publications frequently plotted multiple ratios.

Type of comparison

The examples of graphs displaying the measure of ratio compared the male and female ratios of some event, such as injury and poisoning deaths as shown in *Figure 21*. Comparisons were also made between locations (*Figure 22* and *Figure 23*). Detailed comparisons in *Figure 22* between locations were difficult to undertake due to the large number (7) of locations plotted. Most of the comparisons in the examples were made over a time period, so the comparison of points over a single time series and between time series was also characteristic of these graphs.

Understanding a ratio depends on knowing the variables that take the place of the numerator and the denominator. The correct interpretation of ratios cannot be made without this knowledge. For example, the male to female ratio would be calculated by dividing the male outcome variable by the female outcome variable. If readers were unaware of this order, an interpretation assuming the female variable was divided by the male variable, would give an incorrect result. In all of the examples, the reader could identify the order in which the variables were used from the title. However, this information was never explicitly provided.

Comment on text interpretations (if used)

Text interpretations of graphs displaying ratio measures were found. This was unusual for an example of any of the measures considered in this review. The text accompanying *Figure 21* provided instructions on how to interpret the ratio and provided a definition of a 'steady state' which occurs when the ratio is equal to one. The text provided more detail than was found in the graph because the graph showed overall trends for the ratio of mortality to incidence for all cancers while the text provided detail for mortality to incidence for specific cancers.

A text interpretation was provided for *Figure 22*, noting the mortality rates for males were approximately twice those for females. The text interpretation provided for *Figure 23* only referred to a conclusion that could be drawn from the graph (that the ratio showed that the male mortality rate was higher than the female rate). In *Figure 24* no interpretation of ratios was provided, however the text reference provided several conclusions about the relative size of ratios for particular age groups and the meaning of these differences (between races). This provided the reader with sufficient knowledge to interpret the ratio for other age groups.

Consistency between the health publications sampled

The graphs were not consistent between publications. Differences occurred in the presentation of data (eg bar and line graphs) and the content of the ratio (eg male to

female, Aboriginal population to Australian population, mortality to incidence). Also differences occurred in the expression of the ratio: most examples used the result of the division and one example (*Figure 21*) expressed the ratio as a percentage.

The information included in titles tended to be similar, particularly with regard to the identification of the order in which the ratio components were allocated to either the numerator or the denominator. Ratios were usually expressed as a number although one example was found of a ratio expressed as a percentage.

Other notes

Graphs displaying ratios were conceptually more difficult to understand than the other measures. Knowing that the ordering of variables in the graph titles coincided with the order for division was essential for reader interpretation. The examples of ratio graphs contained the only text reference where the reader was provided with some assistance in interpretation.

Examples

Figure 21. Example of a graph displaying a measure of ratios:
Sourced from *'Cancer in New South Wales: Incidence and mortality 1999 featuring 30 years of cancer registration'*, Cancer Council NSW, 2001, p. 129

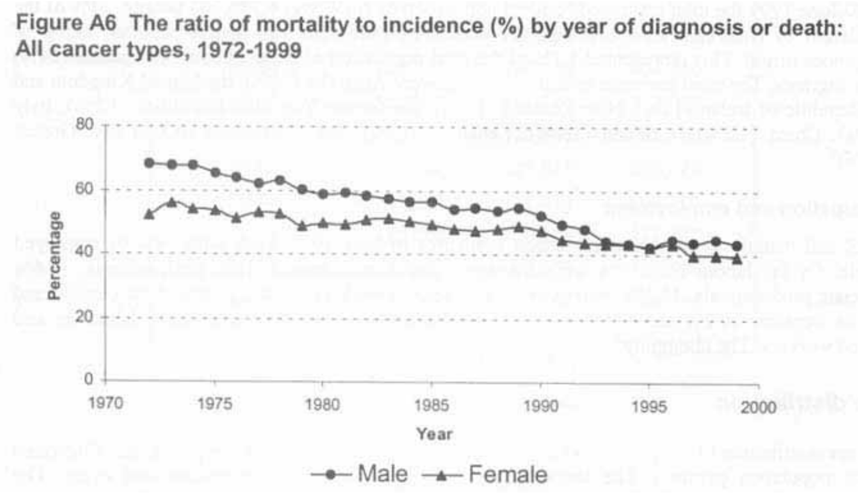

Reproduced with permission from the NSW Department of Health.

Figure 22. Example of a graph displaying a measure of ratio:
Sourced from *'Health Indicators for Queensland: Central Zone'*,
Public Health Services, Queensland Health, 2001, p. 164

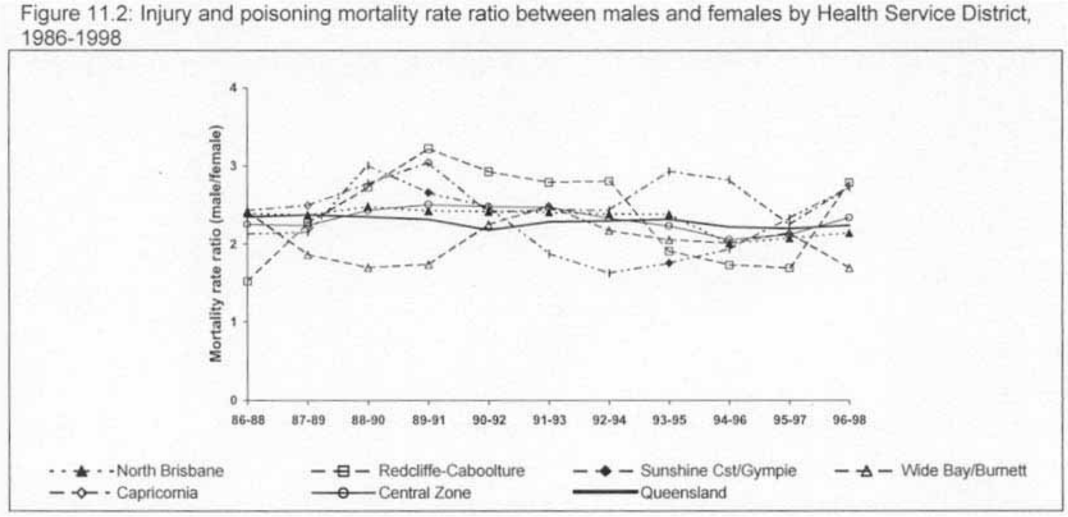

Graph copyright State of Queensland (Queensland Health) 2001. Reproduced with permission.

Figure 23. Example of a graph displaying a measure of ratios:
Sourced from *'Health Measures for the Population of Western Australia: Trends and comparisons'*, Health Dept of Western Australia, 2000, p. 190

Figure 130: Rate ratio male to female injury and poisoning deaths

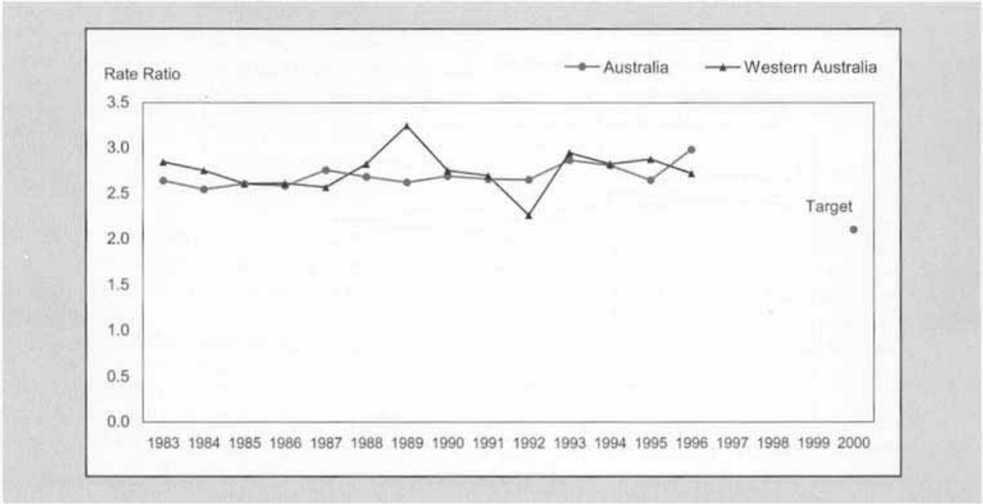

	1983	1984	1985	1986	1987	1988	1989	1990	1991	1992	1993	1994	1995	1996	**2000**
Australia	2.6	2.5	2.6	2.6	2.8	2.7	2.6	2.7	2.7	2.6	2.9	2.8	2.6	3.0	2.1
Western Australia	2.8	2.8	2.6	2.6	2.6	2.8	3.2	2.8	2.7	2.3	2.9	2.8	2.9	2.7	

Notes: 1 Injury and poisoning deaths are selected by ICD-9 external cause codes: E800-E869, E880-E929, E950-E999.
2 Western Australian rate and Australian rate from 1991 to 1996 were based on year of occurrence of death.
3 The death rates used to calculate rate ratios were age-adjusted using the total Australian population as at 30 June 1991.

Sources: 1 Australian Institute of Health and Welfare and Commonwealth Department of Health and Family Services 1997. First Report on National Health Priority Areas.1996. AIHW Cat. No. PHE 1. Canberra: AIHW and DHFS.
2 Health Information Centre 1998. ABS WA mortality database. Health Department of Western Australia.

Reproduced with permission from the Health Department of Western Australia.

Figure 24. Example of a graph displaying a measure of ratios:
Sourced from '*The Health and Welfare of Territorians'*, Epidemiology Branch, Territory Health Services, 2001, p. 192

19.8 NT Aboriginal:Australian death rate ratios 1991 to 1995

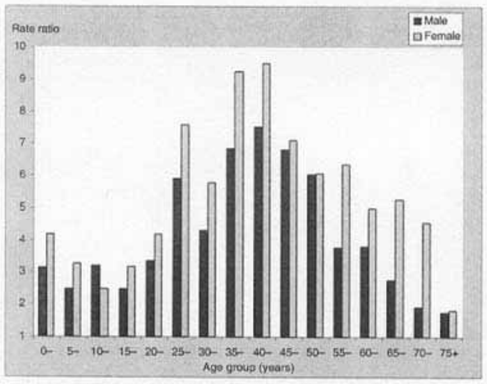

Note: Ratio of NT Aboriginal to Australian death rates for all causes by five-year age groups

Source: Dempsey & Condon 1999

Reproduced with permission from the Northern Territory Department of Health and Community Services.

2.8 Graphs displaying risk

Graphs displaying risk expressed the probability of an event occurring in a *given number of people*. An outcome variable displayed the probability of an event: all graphs showing risk indicated the probability of developing a disease. The expression of probability was one in the number of people at risk. Only one publication used this measure: 'Health Measures for the Population of Western Australia'. In this one publication, nineteen examples were found of this type of graph.

Type of graph used

The graphs displaying risk mainly used line graphs although one column graph was found. The line graphs used markers for each point estimate.

Statistical concepts measured

Graphs displaying a measure of risk used a numerical axis to represent the probability of an event occurring in a given number of people. A time series of probability was also a characteristic of these graphs.

Type of comparison

The examples of graphs displaying the measure of risk often compared the male and female risks of developing some outcome, such as cancer. Comparisons were also made between locations.

Comment on text interpretations (if used)

Text interpretations of risk graphs were not found although each graph was accompanied by text indicating the main points. In all instances it was assumed that the reader knew the meaning of probability and, therefore, could make correct interpretations.

Consistency between the health publications sampled

The graphs were only found in one publication and within this publication the graphs were generally uniform. The two examples selected show that column graphs and line graphs were chosen to illustrate risk, although the document predominantly used line graphs.

Other notes

Graphs displaying risk were not used widely as they were found in only one of the reviewed publications.

Examples

Figure 25. Example of a graph displaying a measure of risk:
Sourced from *'Health Measures for the Population of Western Australia: Trends and comparisons'*, Health Dept of Western Australia, 2000, p. 81

Figure 52: Lifetime risk up to age 74 years for all cancer deaths

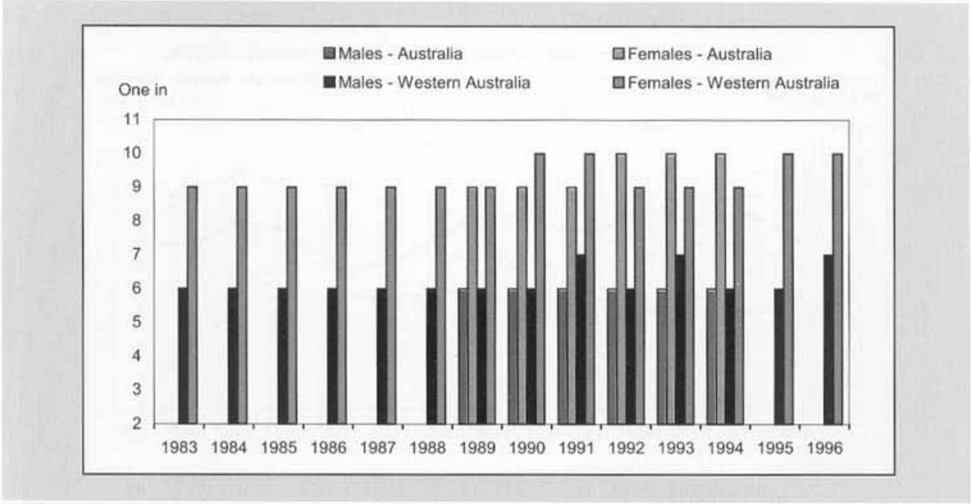

	1983	1984	1985	1986	1987	1988	1989	1990	1991	1992	1993	1994	1995	1996
Australia - Males[1]							6	6	6	6	6	6		
Australia - Females[1]							9	9	9	10	10	10		
WA - Males[2]	6	6	6	6	6	6	6	6	7	6	7	6	6	7
WA - Females[2]	9	9	9	9	9	9	9	10	10	9	9	9	10	10

Notes: 1 Australia: Lifetime risk ICD-9: 140-208, excludes non-melanocytic skin cancers ICD-9: 173. Source 1 (1991-94), AIHW unpublished data (1989-90).
2 WA: Lifetime risk ICD-9: 140-208, includes non-melanocytic skin cancers ICD-9: 173. Source 2 (1983-96)

Sources: 1 Australian Institute of Health and Welfare and Australasian Association of Cancer Registries 1998. Cancer in Australia 1991-94 (with projections to 1999). Canberra: Australian Institute of Health and Welfare (Cancer Series No. 7).
2 WA Cancer Registry, Health Information Centre. Health Department of Western Australia.

Reproduced with permission from the Health Department of Western Australia.

Figure 26. Example of a graph displaying a measure of risk:
Sourced from *'Health Measures for the Population of Western Australia: Trends and comparisons'*, Health Dept of Western Australia, 2000, p. 130

Figure 130: Rate ratio male to female injury and poisoning deaths

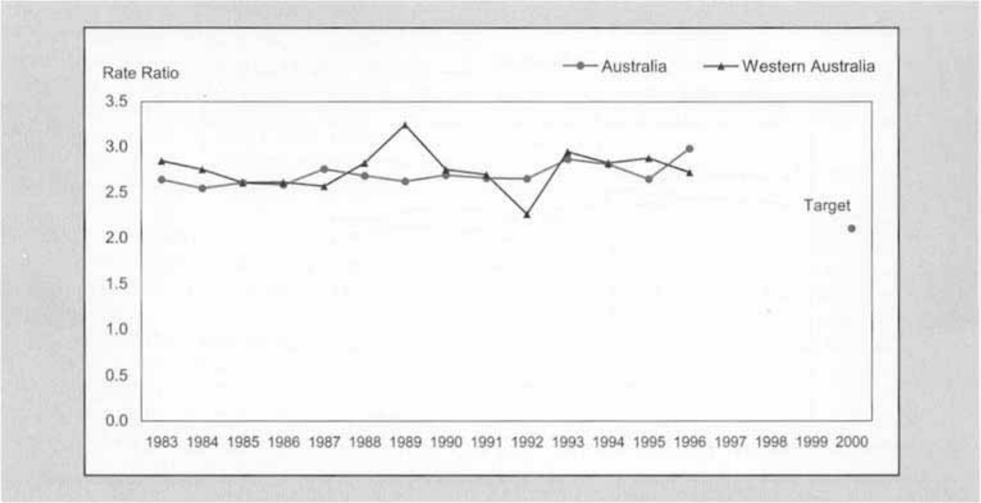

	1983	1984	1985	1986	1987	1988	1989	1990	1991	1992	1993	1994	1995	1996	**2000**
Australia	2.6	2.5	2.6	2.6	2.8	2.7	2.6	2.7	2.7	2.6	2.9	2.8	2.6	3.0	2.1
Western Australia	2.8	2.8	2.6	2.6	2.6	2.8	3.2	2.8	2.7	2.3	2.9	2.8	2.9	2.7	

Notes: 1 Injury and poisoning deaths are selected by ICD-9 external cause codes: E800-E869, E880-E929, E950-E999.
2 Western Australian rate and Australian rate from 1991 to 1996 were based on year of occurrence of death.
3 The death rates used to calculate rate ratios were age-adjusted using the total Australian population as at 30 June 1991.

Sources: 1 Australian Institute of Health and Welfare and Commonwealth Department of Health and Family Services 1997. First Report on National Health Priority Areas.1996. AIHW Cat. No. PHE 1. Canberra: AIHW and DHFS.
2 Health Information Centre 1998. ABS WA mortality database. Health Department of Western Australia.

Reproduced with permission from the Health Department of Western Australia.

2.9 Graphs displaying life expectancy

Graphs displaying life expectancy expressed the average length of time a person might live from birth. Although these graphs could have been classified as a measure of central tendency, they were not always identified in the reviewed publications as 'averages'. Therefore, they have been classified as a separate measure. The use of these graphs in the reviewed publications was 'low'.

Type of graph used

The graphs displaying life expectancy generally used line graphs, although one graph (not included in the examples) used a bar graph. A variation of the standard graph styles was in *Figure 30* that only showed the confidence interval – without the point estimate. In this graph, a thick vertical line showed the ninety-five per cent confidence interval for the State (Victoria). Although this example did not show the actual point estimate, by definition the estimate is within the upper and lower bounds of the interval.

The line graphs mostly used markers for each point estimate that differentiated between the independent variables. In *Figure 28* two graphs were used allowing more independent variables to be included.

Statistical concepts covered

Graphs displaying a measure of life expectancy used a numerical display of number of years of life. Life expectancy is an average although this was not always indicated in the reviewed publications. The graphs were usually presented as a time series to show how life expectancy changed over time. *Figure 30* is an example of a graph showing life expectancy with *only* the confidence interval displayed (no point estimate was shown).

Type of comparison

The examples of graphs displaying life expectancy always compared males and females. Most of these graphs also compared life expectancy over time. Other comparisons were made between race, location and disability. In *Figure 29* nine locations were compared for males and females resulting in 18 series lines plotted on the graph. In *Figure 30* the geographic comparison was made between categories of the independent variable of geographic location (Victorian Local Government Areas and the State). The Local Government Areas (LGA) were ordered in this graph, although the method of ordering was not obviously apparent; it appears to have been based on the point estimate.

Comment on text interpretations (if used)

Text interpretations of life expectancy graphs were found. In particular, NSW Health's 'Report of the NSW Chief Health Officer 2000' provided a definition of life expectancy at birth and explained how the statistic was calculated. Other examples referred to the graph and identified highlights.

Other notes

Graphs displaying life expectancy were not used widely and could probably have been catalogued under measures of central tendency. However, a separate category was created because not all of these graphs were clearly identified as averages in the reviewed publications. They also displayed information that was different from many other types of health graphs in that life expectancy graphs show an expectation of life that is calculated using historical death rates.

Examples

Figure 27. Example of a graph displaying a measure of life expectancy: Sourced from *'The health of the people of New South Wales – Report of the Chief Health Officer'*, NSW Health, 2000, p. 63

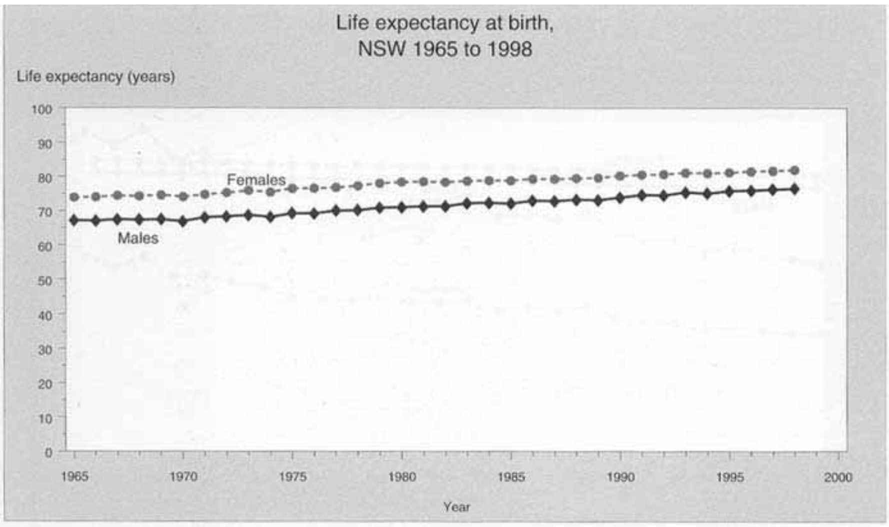

	Sex	1965	1970	1975	1980	1985	1990	1995	1998
NSW	Males	67.1	66.9	69.2	70.9	72.1	73.9	75.7	76.5
	Females	73.7	73.9	76.4	78.3	78.7	80.1	81.1	81.9
AUSTRALIA	Males	67.8	67.4	69.5	71.2	72.5	74.3	75.8	76.5
	Females	74.5	74.3	76.6	78.4	78.9	80.3	81.3	81.9

Note: Abridged current life tables with Greville's correction for neonatal mortality used to calculate life expectancy. This may result in slight differences from life expectancy figures published by ABS.

Source:, ABS mortality data and population estimates (HOIST). Epidemiology and Surveillance Branch, NSW Health Department.

Reproduced with permission from the NSW Department of Health.

Figure 28. Example of a graph displaying a measure of life expectancy:
Sourced from *'Health Measures for the Population of Western Australia: Trends and comparisons'*, Health Department of Western Australia, 2000, p. 70

Figure 44: Expectancy of years of life with disability and handicap at birth

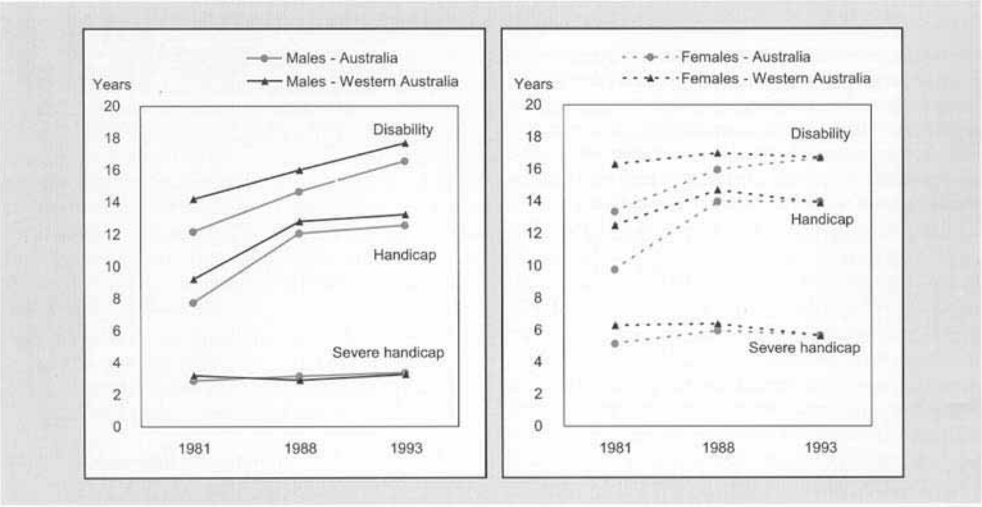

Expected years of life	Australia						Western Australia					
	Males			Females			Males			Females		
	1981	1988	1993	1981	1988	1993	1981	1988	1993	1981	1988	1993
with severe handicap	2.9	3.2	3.4	5.2	6.0	5.7	3.2	2.9	3.3	6.3	6.4	5.7
with handicap	7.8	12.1	12.6	9.8	14.0	14.0	9.2	12.8	13.2	12.5	14.7	13.9
with disability	12.2	14.7	16.6	13.4	16.0	16.7	14.2	16.0	17.7	16.3	17.0	16.7
free of disability	59.2	58.4	58.4	65.0	63.4	64.2	57.9	57.8	57.4	63.0	63.2	64.4
Total life expectancy	71.4	73.1	75.0	78.4	79.5	80.9	72.1	73.8	75.1	79.3	80.1	81.1

Note: 1 Total life expectancy = expected years of life with disability + expected years of life free of disability.

Sources: 1 Mathers C (1991). Health Expectancies in Australia 1981 and 1988. Australian Institute of Health: AGPS, Canberra.
2 Mathers C (1996). Trends in Health Expectancies in Australia 1981-93. Journal Australian Population Association, 13 (1): 1-15.
3 Mathers C (1998). Disability free and handicap free life expectancy in WA 1993 (Unpublished data) Australian Institute of Health and Welfare. Canberra.

Reproduced with permission from the Health Department of Western Australia.

Figure 29. Example of a graph displaying life expectancy: Sourced from *'Victorian burden of disease study, Mortality'*, Victorian Department of Human Services, 2000, p. 16

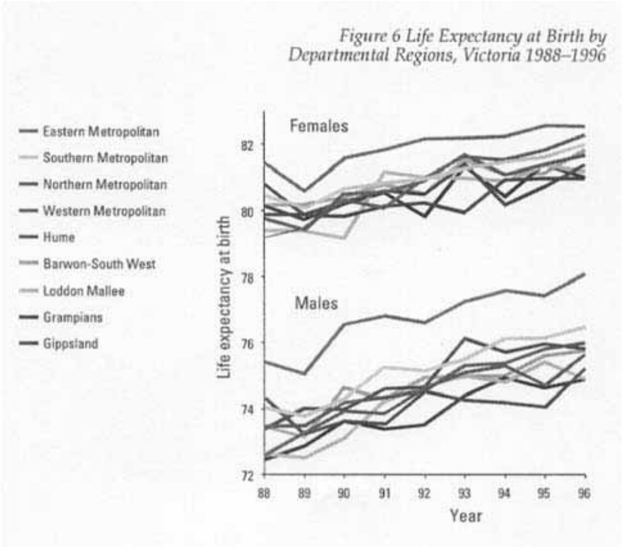

Reproduced with permission from the Health Department of Western Australia.

Figure 30. Example of a graph displaying life expectancy: Sourced from '*Victorian burden of disease study, Mortality',* Victorian Department of Human Services, 2000, p. 17

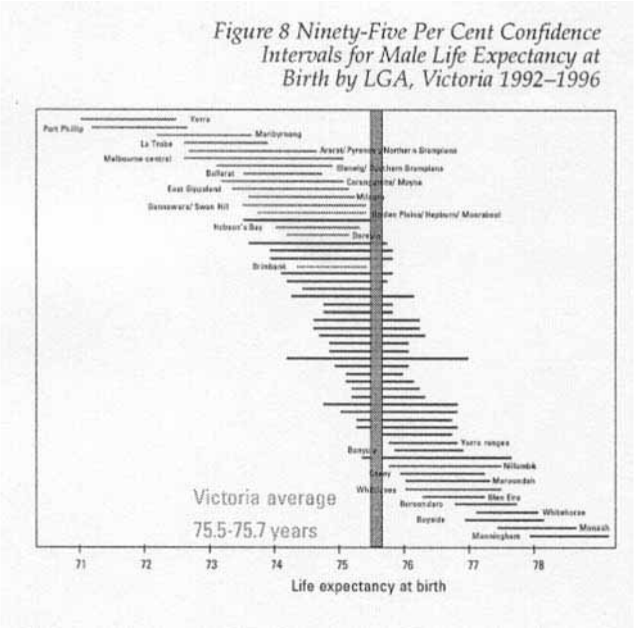

Reproduced with permission from the Victorian Department of Human Services.

Bibliography

Australian Bureau of Statistics (ABS) and the Australian Institute of Health and Welfare (AIHW) 2001, *The health and welfare of Australia's Aboriginal and Torres Strait Islander peoples*, ABS Cat. no. 4704.0, AIHW Cat. no. IHW 6, Canberra (www.abs.gov.au).

Australian Institute of Health and Welfare (AIHW) 2000, Australasian Association of Cancer Registries, *Cancer in Australia 1997, Incidence and mortality data for 1997 and selected data for 1998 and 1999*, AIHW Cat. no. CAN 10, Canberra.

Australian Institute of Health and Welfare (AIHW) 2000, *Australia's health 2000: The seventh biennial health report of the Australian Institute of Health and Welfare*, AIHW, Canberra.

Australian Institute of Health Welfare (AIHW) 2001, National Heart Foundation of Australia, National Stroke Foundation of Australia (2001) *Heart, stroke and vascular diseases, Australian facts 2001*, AOHW Cardiovascular Disease Series No. 14, Canberra.

Coats MS, Tracey EA 2001, *Cancer in NSW: Incidence and mortality 1999 featuring 30 years of cancer registration*, Cancer Council NSW, Sydney.

Cleveland WS 1994, *The Elements of Graphing Data,* AT & T Bell Laboratories: Murray Hill NJ.

Condon JR Warman G Arnold L (editors) 2001, *The health and welfare of Territorians*, Epidemiology Branch, Territory Health Services, Darwin

Department of Human Services 1999, Victorian burden of disease study: Morbidity, Public Health Division, Victorian Department of Human Services, Melbourne

Department of Human Services 2000, *Victorian burden of disease study: Mortality*, Public Health and Development Division, Department of Human Service, Melbourne.

d'Espaignet EJ, Kennedy K, Paterson BA and Measey ML 1998, From infancy to young adulthood: Mental health status in the Northern Territory Territory Health Services, Darwin.

Gordis L 2000, *Epidemiology*, 2nd edition, WB Saunders Company.

Graziano AM, Raulin ML 1989, *Research Methods: A Process of Inquiry*, Harper & Row Publishers, New York.

Kee C, Johanson G, White U, McConnell J 1998, Health indicators in the ACT, Epidemiology Unit, ACT Dept of Health and Community Care, *Health series No 13*, ACT Government Printer, ACT.

NSW Health 2000, *The health of the people of NSW – Report of the Chief Health Officer 2000*, NSW Health Department, Sydney.

Kosslyn SM 1994, *Elements of Graphing,* New York: Freeman.

Kosslyn SM 1985, Graphics and human information processing, *Journal of the American Statistical Association* 80: 499-512.

Last JM, Abramson JH, Friedman GD, Porta Miquel, Spadoff RA, Thuriaux M 1995, *A Dictionary of Epidemiology*, Oxford University Press.

Mahon BH 1977, Statistics and Decisions: The Importance of Communication and the Power of Graphical Presentation, *Journal of the Royal Statistics Society,* 140, Part 3, pp. 298-323.

Parsons J, Wilson D and Scardigno A 2000, *The impact of diabetes in South Australia 2000*, South Australian Department of Human Services, South Australia.

Queensland Health 2001, *Health indicators for Queensland: Central Zone 2001*, Public Health Services, Queensland Health, Brisbane.

Ridolfo B, Sereafino S, Somerford P and Codde J 2000, *Health measures for the population of Western Australia: Trends and comparisons,* Health Department of Western Australia, Western Australia.

Schmid CF 1983, *Statistical graphics: Design principles and practices*, John Wiley and Sons Inc.

Schutz HG 1961, An evaluation of methods for presentation of graphic multiple trends, *Human Factors* 3: 108-119.

Silva DT, Palandri GA, Bower C, Gill L, Codde JP, Gee V and Stanley FJ 1999, *Child and adolescent health in Western Australia – An overview*, Health Department of Western Australia and TVW Telethon Institute for Child Health Research, Western Australia.

South Australian Department of Human Services 1999, *Interpersonal Violence and Abuse Survey,* South Australian Department of Human Services, South Australia.

Tasmanian Department of Health and Human Services 1999, *First Results of the Healthy Communities Survey 1998*, Tasmanian Department of Health and Human Services, Research and Analysis Report, Tasmania.

Tasmanian Department of Health and Human Services 2000, *Demographic and Health Analysis of the Northern Region*, Tasmanian Department of Health and Human Services, Research and Analysis Report No. 4, Tasmania.

Taylor A, Dal Grande E and Wilson D 1996, *South Australian country health survey: March-April 1996*, The Country Health Services Division, South Australia.

The Wallis Group 2001, *Mental Health Promotion Benchmark Survey*.

Wainer H 1984, How to Display Graphs Badly, *The American Statistician*, May Vol 38 No. 2 137 – 147.

Literature review: Best practice principles for graph design

3

3.1 Introduction – even the experts don't get it right

> *Drawing graphs, like motor car driving and love making, is one of those activities which almost every psychologist thinks he can do well without instruction. The results are, of course, usually abominable* (Margerison 1965, cited in Wainer and Thissen 1981, p.234).

The appropriate presentation of data in graphical form has been a contentious issue since William Playfair began developing the practice around 1750 (see Tufte 1983). While the volume of literature in the field is considerable, knowledge of it is, apparently, not widespread. In the thirty-odd years since Margerison's presumptuous (but nevertheless funny) quotation, and despite the relative ease and sophistication with which graphs can now be compiled, even academics (and to be fair, not just psychologists) are still struggling with the problem. This was explicitly acknowledged just a few years ago by the editor of the *Human Factors* journal when several well known researchers in the area were invited to prepare a set of guidelines to assist would-be authors:

> *Whereas one would expect a discipline that claims special expertise in information display to be exemplary in presenting its own graphic material for publication, we find quite the contrary. The illustrations accompanying the typical manuscript to* Human Factors *border on the abysmal* (editorial comment in Gillan, Wickens, Hollands and Carswell 1998, p.28).

This section presents a review of literature in respect of the appropriate presentation of data in graphical format. Related issues include reasons for using graphs rather than some other method of presenting data, such as a table, and ways in which the human visual system perceives and interprets graphical presentations. The ultimate aim of the review is to compile a set of 'best practice principles' to guide practitioners in the compilation of graphs which will stand the greatest chance of being looked at and understood by readers – including those who have technical expertise in the relevant field as well as those who do not.

3.2 Conventions used in this section

1 General principles

This project has been undertaken with the aim of enhancing the effectiveness of graphs in Australian population health publications. However, the review that follows seeks to determine best practice principles for the design of commonly used graphs *in general*.

2 Level of evidence

Multiple best practice recommendations have been derived from references cited in this review, and listed in the bibliography at the conclusion of this section. Findings derived from this review have been categorised as follows:

Level 1 – Tested experimentally in a large representative population: high level of evidence [L1]

Henry (1993) is the only reference in this category. For the experimental testing of his theories, Henry identified and randomly sampled from five population groups. This was the only study in which response rates were cited, for each of the five samples and for all samples combined. Outcome measures were defined in advance of the sampling.

Level 2 – Tested experimentally in a selected or small population or based upon established theory: medium level of evidence [L2]

All of the references, except those specifically noted in categories [1] or [3], fall into this middle category, indicating that some form of field experimentation was undertaken to measure defined outcome variables. This included the coding of answers to open-ended questions in qualitative studies.

Note that Kosslyn (1985, 1989 and 1994) and Gillan, Wickens, Hollands and Carswell (1998) are included here. The recommendations offered by Kosslyn are based upon his own extensive reading of the associated literature and/or established theory or principles (including those from the physical sciences, optometry and psychology). Most of his recommendations have been individually substantiated with specific bibliographic references. Suggestions by Gillan et al. (1998) are based upon their individual and combined

experimental studies in previous years, some of which are separately referenced in this document. Note, however, that it is not clear whether *every* recommendation made by this team has been experimentally substantiated.

Also included are literature reviews by Casali and Gaylin (1988) and Spence and Lewandowsky (1990), though note that these authors also tested their own recommendations experimentally and report the results in these papers.

Comments and suggestions from Lovie and Lovie (1991), Sumner (internet site reference), Wainer (1980 and 1984), Wainer and Thissen (1981) and Tukey (1993) are all substantiated with bibliographic references, though these papers do not explicitly report experimental studies by the authors. Tukey's writings on the subject since the 1960s have formed the basis for much of the theory and experimental testing of graphical perception developed by other authors (notably Cleveland and McGill 1984 and 1985 – see Section 3.5 in particular).

Demana and Waits (1988) approach the issue as mathematicians, considering the potential of computer generated graphs to lead to incorrect interpretations of mathematical functions. Their comments are based upon established mathematical theory.

Level 3 Expert opinion: low level of evidence [L3]

References with a low rating for the level of evidence indicate that the authors did not conduct any experimentation to substantiate their recommendations. Bertin (1983, cited and critiqued in Kosslyn 1985 and Spence and Lewandowsky 1990) and Tufte (1983) are the only authors who fall into this category.

Bertin is considered only in passing. However, a substantial number of the recommendations made by Tufte (which he refers to as *guiding principles* for graph design) are included in this review, even though they are based solely upon his experience as a designer, with no experimental validation. Since Tufte is a well known, highly respected and frequently cited author in this field, it would be imprudent *not* to include him in this literature search. Moreover, many of his suggestions have provided the impetus for extensive debate, and the development and experimental testing of best practice principles.

The levels assigned to each principle are shown against the table of recommendations in Section 3.27.

3.3 To graph or not to graph

To graph or not to graph is not *exactly* the question to be answered in this review since the use of graphs in population health publications is a foregone conclusion. However, decisions still have to be made in respect of determining when a graph will be useful, the type of graph which would be most appropriate, the amount of information to include in the graph, and so on. The literature on these issues is extensive, and the jury is still out on a number of aspects.

1 Tables or graphs?

> *Getting information from a table is like extracting sunlight from a cucumber (*Farquhar and Farquhar 1891, cited in Wainer and Thissen 1981 p.236).

While the utility of, and preference for, graphs over tables has long been recognised, the precise circumstances in which the use of graphs is relevant are disputed, with many studies on the relative merits of tables and graphs providing conflicting results (see, for example, literature reviews by Remus 1987 and Casali and Gaylin 1988). Remus (1987) and later in their review Meyer, Shinar and Leiser (1997) concluded that the relative efficacy of tables and graphs *depends* on multiple factors.

Nevertheless, the general consensus appears to be that a *table* should be used when:

1 Consideration is being given to *a few* data points that have a simple relationship; alternatively, the data/relationship could be placed in the body of the text (Tufte 1983, Tukey 1993 and Gillan et al. 1998).

   Tufte, (1983, p.56) goes so far as to define '*a few*': *tables usually outperform graphics in reporting on small data sets of 20 numbers or less. The special power of graphics comes in the display of large data sets.*

2 The reader needs precise values (Tufte 1983, Spence and Lewandowsky 1990, Meyer 1997 referring to one of the first studies in this field conducted by Washburne in 1927, Gillan et al. 1998, Cleveland and Fisher 1998 internet site reference).

3 Many localised comparisons are required (Tufte 1983). In describing a table he designed for the *New York Times* to show how different people voted in presidential elections in the United States, Tufte (1983, p.179), with typical immodesty, says: *this type of elaborate table, a supertable, is likely to attract and intrigue readers through its organised, sequential detail and reference-like quality. One super table is better than a hundred little bar charts*.

4 Relations in the data do not lend themselves to visualisation: for example, an irregular pattern of means (Gillan et al. 1998).

Beyond these simple criteria the decision about whether to use a table or graph becomes more complicated. Benbasat and Dexter (1985), Remus (1987), Meyer et al. (1997) and Carswell and Ramzy (1997) concentrated on both the task which users are required to perform and the complexity of the data.

Benbasat and Dexter (1985, p.1348) concluded that the graphic, rather than tabular, presentation of data will not improve decision making unless it is in a form to *directly* assist the decision task: *it is not correct to expect graphical reports to be better for any and all problem contexts…[graphs are relevant] to task environments where there is a clearly defined rationale for the potential benefits of graphics usage and where graphical reports are organised in such a way to best support the task at hand.*

From experiments contrasting the effectiveness of graphical and tabular displays in making production scheduling decisions, Remus (1987) found that tabular aids outperform the graphical aids in environments with low complexity, while in intermediate complexity environments the graphical aids outperform the tabular aids.

In addition to task and complexity of data, Meyer et al. (1997) also evaluated users' experience with the displays and their familiarity with the data in a series of experiments to evaluate the relative efficacy of tables, bar graphs and line graphs in terms of response times and accuracy of response. They found a systematic advantage of tables over graphs, and demonstrated the existence of complex interations between performance and the complexity of the displayed data and users' experience with the display.

Carswell and Ramzy (1997) found in favour of graphical presentations, even for small data sets. They note the general assumption that the larger the data set, the greater the value of graphical formats, but contend that if the utility of graphs does decrease with the size of the data set, then graphical advantages found for small data sets provide particularly strong evidence for graph usage in general. Specifically, in consideration of tables, bar graphs and line graphs for displaying data sets of varying sizes and complexity, the authors found that the amount of *integrative* (or overall global) content extracted from the displays was lowest for tables, and highest for bar graphs. Additionally in respect of tables, increased complexity led to progressively longer study times but with little change in the amount of content extracted to show for it.

The most relevant point here is, it seems, that one should carefully consider whether the inclusion of a graph really will increase readers' understanding of the points being made: it should not be assumed that a graph will automatically be of benefit. Multiple factors should be considered, including the characteristics of the potential users of the document and their familiarity with both the data and various types of graphical displays, the type and complexity of the data being graphed, and the information which the author would like the reader to extract form the chart. These points are developed further in the following sections. Of course, both a graph *and* a table of the same data set could be used if they help to communicate the message (see, for example, Mahon 1977).

2 The advantages of graphs

*A good graph forces us to notice what we never expected to see (*Tukey 1977, cited in Wainer and Thissen 1981, p.235).

Despite reservations regarding the precise circumstances under which the use of graphs is appropriate, that they may be useful tools is, of course, not disputed. And academics agree that they are *most* useful in illustrating relationships among data, even in small amounts of data (see, for example, Kosslyn 1994, p.20).

Tufte (1983, p.51) contends that graphical excellence consists of (amongst other things) *complex ideas communicated with clarity, precision and efficiency...[it] is that which gives to the viewer the greatest number of ideas in the shortest time with the least ink in the smallest space*.

After many years of writing on the subject, Tukey (1993, p.2) distilled four basic functions of graphs, at the same time indicating purposes for which he believed graphs should not be used:

1 *Graphics are for the qualitative/descriptive – conceivably the semi-quantitative – never for the carefully quantitative (tables do that better).*

2 *Graphics are for comparison – comparison of one kind or another – not for access to individual amounts.*

3 *Graphics are for impact – interocular impact if possible, swinging-finger impact if that is the best one can do, or impact for the unexpected as a minimum – but almost never for something that has to be worked at hard to be perceived.*

4 *Finally, graphics should report the results of careful data analysis – rather than be an attempt to replace it...*

Casali and Gaylin (1988) also note studies in which experimental subjects indicated a (subjective) *preference* for graphics over tabular or textual formats. This point, in conjunction with Tukey's point (iii) above about the impact of graphics, reinforces the view that a good graph will attract readers' attention, enticing them to consider something they may otherwise have skimmed over. In promoting the use of graphics to aid communication, Mahon (1977, p.298) reminds us that *no matter how excellent [the statistician's] experimental design and analysis, and no matter how sound his conclusions... if the message still does not get through he has failed.*

To test the assumption that only the most simple graphics should be used in reports, and the remaining evaluation information should be relegated to tables, Henry (1993) used quite complex graphics for his subjects to carry out relatively complex comparisons. Their success led him to conclude (p.76) that *we should use graphic displays to encourage analysis by practitioners and the public*.

In addition to, or perhaps because of, the ability of graphs to illustrate data relationships, displays in general and graphical presentations in particular have been examined in the context of facilitating understanding and memory. The augmentation of text with *pictorial imagery* is regarded as a *richer cognitive representation* of student material (Sparrow 1989, p.44), and experiments have confirmed our extraordinary capacity to remember pictorially presented information and demonstrated that memory for pictures is superior to memory for text (Spence and Lewandowsky 1990). It is not surprising then that students have been found to learn more effectively when verbal explanations and illustrations are presented *together* rather than in a form which summarises the explanation only in words or only in pictures (Mayer, Bove, Mars and Tapangco 1996). Ferry, Hedberg and Harper (1999) reported that most of their student subjects thought that associated text, numbers and graphs were mutually reinforcing.

Even though, as noted above, the use of tables rather than graphs has been espoused for the determination of precise values, much time and effort have been devoted not to the tables versus graphs debate, but to the graph versus task debate. A task often considered when evaluating the effectiveness of graphical presentations is point reading, the implicit assumption being that graphs *are* suitable for this function, and the relevant question for research is *which type of graph best facilitates it?* Certainly this question is considered in the current review. In a relatively recent study Meyer, Shamo and Gopher (1999) provide empirical support for the preference of graphs over tables on the basis that the *actual* use of displays differs from the use made of graphs in laboratory experiments in at least one respect: outside the laboratory the displayed information usually *has meaning* and therefore holds some kind of structure – it is *non*-random. Structured data in this sense are data in which consistent patterns appear because of correlations between data points. Meyer et al. (1999) demonstrate that since structured data will generate regular visual patterns, this property of graphs – the transformation of regularities in the data into consistent visual patterns – is the major advantage of graphs, particularly so when the task that has to be performed requires the use of this structure, or strongly benefits from it.

While Tukey (1983) cautioned that graphical analysis should not *substitute for* careful statistical analysis, he nevertheless keenly promotes the technique for use *in conjunction with* traditional mathematical techniques to gain insights into the structure of data. Chambers, Cleveland, Kleiner and Tukey (1983), Cleveland and McGill (1988) and Cleveland (1993) devote entire texts

to this issue, including the use of graphs in association with multiple data structures to reveal aspects of data which may not otherwise become obvious. In the same vein, Lovie and Lovie (1991) note that, properly designed, graphs should not only summarise results but should also highlight in a direct fashion any anomalous data or pattern of results. They too explore the use of multiple graphical formats (box plots, scatter plots, sunflower plots, smoothed plots and others) to assist with statistical analysis.

Demana and Waits (1988) sound a note of caution in relation to their concern about the relative ease with which computer graphics can now be generated, and emphasise the view that graphs should not *substitute* for statistical analysis. By presenting multiple computer generated graphs which have the potential to mislead unless care is exercised when they are interpreted, Demana and Waits (1988, p.177) make the point that, *while the traditional approach [to mathematical study] uses careful numerical and algebraic investigation to produce the graph of a function, computer generated graphs of a given function can be used to determine its important numeric and algebraic properties. However, students and teachers must make sure that a computer generated graph of a given function is valid.*

A related issue is the use of graphs to either intentionally or unintentionally mislead. This is discussed further in Section 3.8, guideline (vi).

3.4 The relevance of perceptual issues to graph construction – why do perceptual issues matter?

> *Traditionally, the study of visual learning has not received the same kind of attention as the study of verbal learning. Although educators recognise the potential value of graphics in instruction, there has been little evidence that graphics are living up to their potential* (Mayer 1993, p.241).

Perceptual issues matter if we are to make graphics live up to their potential. At the heart of much of the literature is the notion that preferred graphical styles are related to the way in which the human visual and cognitive systems process and interpret the information in graphs. Because of latent biases and abilities within these systems, some types of graphs will be more easily interpreted than others.

Multiple theories of the way in which we perceive graphs have been developed on the basis that cognitive processes may be supported or inhibited by specific graphical renderings (for example, the form of the graph – line, bar, pie etc., location of labels, presence of tick marks on axes etc.) that clearly influence the utility of different forms of data representation (Wickens, Merwin and Lin 1994). An appreciation of these cognitive processes allows us to make scientifically well founded judgments in order to optimally design and employ graphs. In other words, stylised models of perception have implications for graph design. By understanding the ways in which we perceive graphs we can begin to determine the way in which, and the conditions under which, graphics are processed such that adequate comprehension ensues.

Mayer (1993, p.241) summarises the relationship between understanding the cognitive processes behind the interpretation of graphs, and the optimal use of graphs as a learning tool as follows:

> The 'traditional' approach to evaluating the effectiveness of graphics is to ask *do graphics affect how much students learn?* In this case the model is:
>
> *Graphics and text* ➔ *performance*
>
> The 'cognitive' approach asks *how and when do students learn from graphics?* In this case the model is:
>
> *Graphics and text* ➔ *cognitive processing* ➔ *mental representation* ➔ *performance*
>
> The cognitive approach seeks to describe the cognitive processes that are used in comprehending graphics, as well as the mental representations that are constructed and used to answer questions. The research question posed is *how does a learner process visual and verbal information in order to build a mental model of the material?*

Differences in theories lie in the way in which proponents model the cognitive and visual processes which occur. Related aspects include:

- The intended purpose(s) for which the graph is constructed by its author or, alternatively, the task(s) which users of the graph may be required to undertake.
- The level of expertise and experience which potential users of the graph may have in the relevant subject area, and their relevance to the type of display presented.

As the literature has progressed, these aspects have been incorporated into cognitive process models and have, in turn, influenced recommended ways in which data should be graphed.

In order not to get too bogged down in the perceptual theories underlying best practice principles for graph design specified in Section 3.8 and those following, the next three Sections (3.5, 3.6 and 3.7) provide a brief overview of major issues which have emerged from the perception literature. It is noted that many models of graphical perception have been developed. This document is mainly concerned with categorising their implications, rather than identifying and reviewing each of the models. However, a brief exposition of some follow below because they are considered significant in the development of the literature, and/or they provide a useful means by which to illustrate some of the recommendations being made.

3.5 Founding members of the perceptual issues debate

> *… in choosing, constructing and comparing graphical methods we have little to go on but intuition, rule of thumb, and a kind of master-to-apprentice passing along of information…there is neither theory nor systematic body of experiment as a guide* (Kruskal 1975, cited in Cleveland and McGill 1984, p.531).

Cleveland and the two McGills were among the first to systematically link theories of cognitive process and visual perception to the comprehension of graphic presentations. Their model is a cornerstone of much of the research on graphical perception discussed below, and is based on a mixture of theoretical and empirical findings.

Cleveland and McGill (1984 and 1985) suggest that visual dimensions can be ordered in terms of how well people use them to compare quantitative variations. Building on Gestalt principles and the work of Pinker, Kruskal and Tukey, among others, they make considerable use of the concept of preattentive vision – the instantaneous and effortless part of visual perception that the brain performs without focusing attention on local detail – as well as Weber's Law and Stevens's Law from sensory psychophysics, and assert that graphs should be constructed to accommodate preattentive vision as much as possible. Kosslyn (1989, p.195) cites references to Gestalt Laws that describe how forms are perceptually organised and notes that the more important laws in respect of preattentive vision (though he did not explicitly use this term) can be summarised by four general principles:

1. *Good continuity – marks that suggest a continuous line will tend to be grouped together. So, a series of marks such as '— — — — — — ' are seen as forming a single line, not a series of isolated dashes.*
2. *Proximity – marks near each other will tend to be grouped together. So 'xxx xxx' is seen as two units, whereas 'xx xx xx' is seen as three.*
3. *Similarity – similar marks will tend to be grouped together. So, '!!!OOO' is seen as two units.*
4. *Good form – regular enclosed shapes will be seen as single units. So '[ ]' is seen as a unit, whereas '[l' is not.*

Cleveland and McGill (1984 and 1985) contend that a graphical form which *involves* **elementary perceptual tasks** *that lead to more accurate judgments than another graphical form (with the same quantitative information) will result in better organisation and increase the chances of a correct perception of patterns and behaviour* (Cleveland and McGill 1984, pp.535-536, emphasis added). Furthermore, *when a graph is constructed, quantitative and categorical information is encoded, chiefly through position, shape, size, symbols, and colour. When a person looks at a graph, the information is visually decoded by the person's visual system. A graphical method is successful only if the decoding is effective…Informed decisions about how to encode data can only be achieved through an understanding of this visual decoding process, which we call graphical perception* (Cleveland and McGill 1985, p.828).

Cleveland and McGill (1984 and 1985) identified certain elementary graphical perception tasks that are performed in the visual decoding of quantitative information from graphs, and experimental data was then used to order the tasks on the basis of accuracy. The tasks below are ordered from most to least accurate:

Rank	Aspect judged
1	Position along a common (aligned) scale
2	Position on identical but non-aligned scales
3	Length
4	Angle<br>Slope
5	Area
6	Volume<br>Density<br>Colour saturation
7	Colour hue

The implication of this ordering is that data should be encoded (that is, the graphs drawn) so that the visual decoding (perception of them) involves tasks as high in the ordering as possible (tasks performed with greater accuracy).

This model is sometimes referred to as the *basic tasks approach*. It implies that judgments of linear extent are made more accurately than judgments of area or volume, which are systematically underestimated, with the effect greatest for volume. Cleveland and McGill (1984 and 1985) therefore recommend that lengths be used, as opposed to areas or volumes, to represent magnitudes wherever possible. Most preferable is the plotting of each measure as a distance from a common baseline so that *aligned lengths* are being compared.

This ordering is implicitly supported by the experimental work of Culbertson and Powers (1959) and others reported in Spence and Lewandowsky's (1990) literature review. To show individual components in addition to the total it was found that a *grouped graph* (one with separate graphical elements originating from a *common baseline* to represent both the components and the total) is superior to a segmented or divided graph (where the total is represented by a single graphical element, for example, a divided bar graph, with subdivisions representing the constituent components). This superiority was determined to be independent of the particular choice of graph: a grouped bar chart was superior to a divided bar chart and a grouped line graph (where each line is drawn with reference to the abscissa as baseline) outperformed a segmented line graph (where each line s drawn with the line below as the baseline).

In a meta analysis of 39 published experiments, Carswell (1992a) found some support for the task ordering, though her findings suggest not so much an ordering as two categories, with the members of one – area and volume – being *inferior* to members of the other. In addition, she determined that the ordering of visual dimensions is largely dependent on the type of task to be undertaken using the graph. This issue is considered in more detail in the following section.

The Cleveland and McGill model has proven particularly useful in directing subsequent research, which has resulted in developments described immediately below. However, one way in which the basic tasks approach is particularly limited is in respect of the preference for the *position on a common aligned scale* type of graphical configuration. While this is fine as far as it goes, position on common aligned scales can be configured in many ways. No guidance is given for choosing between these types of graphs in general, or in relation to their component elements in particular. This review attempts to delve further into the issue.

3.6 Considerations of requisite task in determining preferred graph type

1 Task versus structure

One of the main reasons why Cleveland and McGill's basic tasks approach is contentious is because it assumes that one type of graph is always, under all circumstances, preferred to another. However, it has been established that the emphasis on structural characteristics alone is insufficient to account for many aspects of graphical communication. A significant finding in the field of comparative graphics is that the efficacy of any graphical format is highly task dependent. This strongly suggests that we need to take note not only of how our graphs are composed (their structural characteristics), but also of the processing demands of the tasks the graphs subserve (what readers are doing with the graphically presented information). It is essential to describe ways in which processing demands interact with structural characteristics to determine the overall performance of the user (Carswell 1992b).

Sparrow (1989) suggested the notion of *task/display compatibility*: the degree to which the relevant information for the task appears directly in the display so that no computations and transformations have to be performed. This notion is further developed by Carswell (1992a, p.3698) who notes that, *though a host of structural-organisational features probably influence graphical efficacy, the relative importance of any of these may vary with the graph user's specific goals... the benefits that accrue to specific formats are largely task dependent...as well as [being dependent on] the graph user's exposure to various graphical formats*.

Associated with this issue are the related theoretical issues of:

- The *local* and *global* nature of graph reading tasks, and graphical *proximity*
- The *configurality* of elements and *emergent features* in graphic displays.

2 The local and global nature of graph reading tasks, and graphical proximity

The interpretation of graphs can be a *local* process (for example, one requiring point by point attention) or a more *global* one (for example, determining trends in, or identifying interaction among, the data). There are many global features of a graph that can be interpreted, including the general shape of the graph, intervals of increase or decrease (first derivative) and intervals of extreme increase or decrease (second derivative). A qualitative interpretation of a graph in its fullest sense requires looking at the entire graph and gaining an understanding of the relationship between the variables and, in particular, their pattern of covariation.

The *proximity compatibility principle* developed predominantly by Wickens and Carswell and associates is one framework which has been proposed specifically in response to the need to understand structure-process interactions in the reading of graphs. The principle is based on the notion of *integral* and *separable* dimensions of graphs. It predicts that tasks requiring the integration of information across data points (global interpretation) will be better performed with integrated displays: that is, displays that are high in *structural proximity*. Conversely, for tasks requiring the focus of attention on one variable (local interpretation), performance is better served by more separate displays: that is, those which are low in structural proximity. The proximity compatibility principle is related to the concept of directness: when there is compatibility between the task and the display, perception of the judged characteristic is direct, requiring simpler or fewer mental operations.

Carswell (1992b) provides a thorough exposition of the principle and its subtleties, as well as empirical support for it. Subsequent empirical support came from Henry (1993) who determined that proximity is important to facilitate comparisons, the implication being that authors must consider formats which will bring the data they anticipate being most useful for comparison into close proximity. Similarly, in recapitulating and critiquing a study by Washburne in 1927, Meyer (1997) suggests that the relative efficacy of displays for tasks (other than reading values) depends on the logical ordering of data *within* a display, and that the visual features of displays are often less important than the correspondence of the display's structure with the structure of the information the user attempts to gain. Using Washburne's original data, Meyer (1997) found that when data elements were ordered such that those which had to be compared were in close spatial proximity, the display was more efficient (led to more accurate recall) than when the logical ordering placed the elements further away from each other.

3 The configurality of elements and emergent features in graphic displays

The proximity compatibility principle was refined by further considerations of the meaning of display, or structural, proximity. As Carswell (1992b) makes clear, a variety of graphic formats may be considered to be high in structural proximity, including displays in which the various specifiers are contained in a single unitary object, and those composed of multiple heterogeneous specifiers which configure to create *emergent features*. The latter type of display is referred to as a *configural* display. A configural relationship refers to an intermediate level of interaction between separable and integral perceptual dimensions: each dimension maintains its unique perceptual identity, but new emergent properties are also created as a consequence of the interaction between them. Emergent features are high level, global perceptual features that are produced by the interactions among individual elements of a graph (for example, lines, contours and shapes which

occur when two variables are mapped – one in the x axis and one in the y axis – to produce the emergent features of area). Emergent features are dependent upon the identity and arrangement of component elements, but not identifiable with any single element. Different configural displays will produce different emergent features, and the number and quality of them will contribute to its effectiveness.

The issue of configurality has generated much discussion, with Carswell and Wickens at times appearing to get bogged down in their own debate, and having difficulty in classifying various types of displays according to their own definitions. Carswell and Wickens (1990) evaluated 13 combinations of perceptual dimensions, considered to be representative of those used in graphic displays. They found no stimulus sets that satisfied all of the operational definitions for integrality, two that satisfied definitions of separability, and two that satisfied definitions of configurality. It was concluded that, for the design of graphic displays, separable and integral dimensions may represent idealist end points and a continuum of configurality exists. Carswell (1992b) continued with the classification dilemma, finding it not as straightforward as she had originally thought, and having to modify her definitions of integral and configural dimensions on the basis of further complex criteria (see Carswell 1992b, pp.627-632). It is eventually concluded that *a processing taxonomy composed of characteristics other than the simple presence of absence of integration demands is necessary if the efficacy of both homogeneous and heterogeneous displays is to be adequately predicted. Also, while configurality is certainly important in determining graphical efficacy, it is not a sufficient explanation for the performance enhancement that usually, but not always, occurs when graphical specifiers are combined into a single object*. (Carswell 1992b, p.639). Others have taken up the charge from this point.

Bennett and Flach (1992) come to much the same conclusion. In making use of Kosslyn's (1989) approach to graphic comprehension, they contend that consideration of the principles of configurality are necessary, but not sufficient, for effective display design. They acknowledge that graphical elements of a display will interact to produce emergent features, and that this is critical for the design of graphic displays, especially those intended to support performance at integration tasks (in which more than one variable must be considered). Their twist to the debate is that a single display can, and should, support performance at *both* integration and focused attention tasks (where only one variable is considered).

According to Bennett and Flach (1992), graphic displays map information from an underlying *domain* into visual features: in complex dynamic domains, individuals must consider a continuum of information ranging from high-level constraints (for example, the status of processes, or properties defined by the relationship between variables) and low level data (measured values of individual variables). The task(s) to be completed using the graph are defined in terms of its visual features *as well as* the domain. In the model espoused by Bennett and Flach (1992), the terms integral, separable and configurable are not specific to any visual form, but refer to the mapping of the domain semantics onto the visual form. Thus, the same graph format can be either separable, configural or integral depending on the mapping to the process variables. They contend that objects can be considered as a set of hierarchical features (including elemental, configural and global features) that can vary in their relative salience (visual prominence). Observers may focus attention at various levels of the hierarchy at their discretion, and there may be no inherent cost associated with focusing attention on elemental features. The implication is that a single display format is capable of achieving the dual design goals of supporting performance in both integrated and focused tasks, and there is *no* trade-off between integrated and focused attention.

Bennett and Flach (1992) support their claims by a review of experimental results of performance with configural and separate displays. They found improved performance for integrated tasks with configural displays, but few differences between display formats for focused tasks. This suggests that there is little or no cost involved (in terms of the sacrifice of focused attention tasks) if information is displayed in a more configural type of graph. Their conclusion is that parts never completely lose their identity relative to the whole. When the parts are configured to produce emergent features, information related to the parts is available alongside the high level emergent features, and can be focused on when so desired. Bennett and Flach (1992, p.529) suggest that the perceptual salience of the

elemental features may be increased to support the extraction of low level data (values of individual variables) through a variety of techniques including:

1 *Colour coding the graphical elements*
2 *Maintaining and emphasising scale*
3 *Spatially separating the graphical elements*
4 *Augmenting graph forms with digital values.*

Bennett, Toms and Woods (1993, pp.95-96) provide further theoretical and empirical support for the notion of dual purpose displays, and stress that failure to achieve either of the following requirements will reduce the effectiveness of the display:

1 *The semantics of the domain must be determined...[in terms of] the high level constraints of interest, the low level data that are relevant to those constraints, the relationships between these low level data, and the relevant goals and constraints*
2 *A display must be designed that produces emergent features that directly reflect the domain semantics...[and] highlight the critical data relationships in the domain.*

While it is conceded that these requirements are not easily achieved, and problems associated with their achievement are discussed, few suggestions to overcome the difficulties are provided beyond those already noted relating to colour coding etc. to facilitate extraction of low level data.

Greaney and MacRae (1997) provide an excellent practical example, in the form of check reading a display for the presence of an abnormal reading, of dual local and global processing using a single display. Their visual search paradigm focuses on *preattentive* (or *parallel*) and *serial* processing. For the former, the time needed to decide whether or not a multi-element display contains a particular 'target' element does not depend on the number of displayed elements since all elements are processed in parallel. Response times that increase as a function of the number of elements indicate serial processing (or search) requiring focused attention.

In their experiments Greaney and MacRae (1997) evaluated the properties of (integral) polygon displays and (separable) bar graphs as fault indicators for systems with many parameters. When the task was fault detection, the subjects performed equally well with both types of displays. When the task was counting the number of abnormalities, performance with the bar graphs was independent of the number of abnormalities, but performance with the polygon display was poor overall and deteriorated with larger numbers of abnormalities. Identifying fault states is a high-proximity task, requiring integration of all the available information to generate a single response. Identifying the number of faults is a relatively low proximity task requiring attention to individual elements of the display to select from a larger number of response categories. Both tasks were performed as well or better with the low proximity bar graph than with the high proximity polygon display. These results contradict either the proximity compatibility hypothesis of Wickens, or the traditional classification of polygons and bar graphs as typical integral and separable displays respectively. They support the contention of Bennett et al. (1992 and 1993) that a single display can support multiple tasks because of its emergent features. It might be expected that a bar graph, because it is separable rather that integral, would be processed serially, giving search times that increase as a function of the number of displayed parameters. However, the bar graph has emergent properties: the tops of the bars form a contour which is distorted by an abnormal bar, and if a fixed line marks the limit of normality, a bar that is out of limits is cut by it, forming a new rectangle. This is an instance of an ostensibly 'separable' display being processed in parallel.

As a final note on the issue of matching a chart to the requisite task, Meyer, Shamo and Gopher (1999) emphasise that displays should be analysed vis-à-vis the task as well as the *structure* of the data being presented, and chosen in order to maximise the salience of the task relevant structure. That is, we have to understand the *properties* of the displayed information, *and* how it will be used, in order to determine how to present it.

3.7 Users' expertise in constructing and reading graphs

Not only will requisite tasks, or the purposes for which a graph is constructed, vary, but graph users will be more or less practised in the art of reading graphs, and more or less expert in the field to which the graph relates.

Roth and McGinn (1997, p.93) complain that a frequent claim in the education literature, often based on age related differences in test results, maintains that *students' difficulties in graphing tasks arise from deficiencies in logical reasoning ability, including spatial thinking and proportional reasoning. Younger students and anyone else who is not a 'formal thinker' cannot be expected to graph properly*. They contend that this view does not account for variations in performance across contexts and tasks, and that difficulties should not be attributed to students' *deficient cognitive apparatus*.

The main problem Roth and McGinn (1997) see with cognitive models of graph interpretation is the concept of a graph as something that exists in itself, and has more or less unambiguous meanings. From this perspective, they contend, one immediately focuses on students' errors. On the other hand, if constructing and interpreting graphs is regarded as but one of a range of discursive practices, the focus begins to shift toward students' experience and use of graphing. Consequently, the authors view graphing as one of many elements in constructing and re-presenting phenomena, and suggest that students need to participate actively in the development and maintenance of the skill. Rather than being an abstract ability, graphing is considered a practice in which one is more or less competent, with competence partly a function of the extent of experience. In experimental work with Year 1 and Year 3 students, pre-service teachers and science graduates, Ferry et al. (1999) found that pre-service teachers who had completed an undergraduate science degree prior to commencing the teacher education program were better at interpreting graphs than those who had not completed such a degree. This finding may support Roth and McGinn's (1997) argument that graph construction and interpretation is a substantially learned behaviour, given that people with science backgrounds have more exposure to it than those with less technical educational backgrounds.

Roth and McGinn (1997) were, perhaps, a little hard on the cognitive school. Kosslyn (1985 and 1989) recognised, at least implicitly in his discussion of long term memory processing, that understanding graphic displays was learned behaviour. Henry (1993, p.76) specifies that, *as more complex graphical forms become stored in long-term memory through their use in conveying information, the theory of graphical perception tells us that the facility for comprehending the information will be enhanced*. Meyer (1998, cited in Meyer, Shamo and Gopher 1999) demonstrated that task performance with information displays generally improves as users gain experience.

In relation to their model of graphical comprehension, Guthrie, Weber and Kimmerly (1993) suggest that students' interpretation of graphs may be inhibited if they have not adequately learned one of the key components of the model, referred to as *abstraction*: the forming of relationships among categories of information. Abstraction may consist of comparing trends or spatial patterns in different sections of the graph, and entails formulating higher order interpretations from the basic data displayed, and combining generalisations that have been formed from details in the different sections. In experimentation requiring students to locate specific information as well as perceive trends and patterns, Gutherie et al. (1993) inferred that performance on trend recognition benefited from the abstraction processes, and that these processes were relatively independent of the components of search needed for performance on the information extraction tasks. The level of competence in searching for trends was significantly lower than in searching for specific information, suggesting that the abstraction process had not been learned by a substantial number of students.

Still on the subject of the didactic potential of graphs, Wickens, Merwin and Lin (1994) built into their experimental design the issues of short-term performance with various types of displays, and their effectiveness for longer-term comprehension. Interestingly, they found that what is optimal for short term performance gains may not be ideal for long term knowledge acquisition.

One of the best demonstrations of the effect of experience on people's ability to comprehend graphs in general, and in the particular field to which the graphs relate, is provided by Lowe (1993). In line with Kosslyn (1989), Lowe (1993) contends that a chart or diagram may not serve its intended instructional functions if there are information processing constraints that limit the way it is perceived. If a graph is to be an effective instructional resource, the assumption is that the reader will be able to build a meaningful and appropriate mental

representation of the *scientific system* it portrays. If the mental processes necessary for conceptual recall and problem solving lack an appropriate mental model upon which they could operate, then the reader will fail to comprehend the graph. Lowe further hypothesises that it may be difficult for novices in a particular area to develop an appropriate mental model of the system depicted.

The crux of Lowe's (1993) argument is that the nature of the mental representation constructed from a display during its initial visual processing can be characterised as a function of the interaction between information provided in the display and the person's background knowledge. When the display is an abstract technical diagram, two types of background knowledge are central to the construction of an appropriate mental representation from the diagram:

1 *Domain-general knowledge* which is applicable across a wide range of visual stimuli and possessed by people at large ('everyday' visual knowledge).
2 *Domain-specific knowledge* which has quite restricted applicability and is largely confined to those who have expertise in the technical domain depicted by the diagram. This knowledge can be an important influence on how new information from a particular domain is represented mentally, with a high level of background knowledge found to facilitate the processing of new domain-related information.

For experimentation with drawing and recall of weather maps, Lowe (1993) uses a model of visual processing developed by Humphreys and Bruce (1989, cited in Lowe 1993, p.160) consisting of three components:

1 *Perceptual classification*
2 *Semantic classification*
3 *Naming.*

Lowe postulates that while perceptual classification involves structural descriptions only of object appearance (such as shape and position), semantic classification involves associative and functional aspects: for example, one of the composite symbols that is commonly found on weather maps might be characterised at a perceptual level as a group of triangles attached to a curved line, whereas at a semantic level it would be characterised as a cold front. Generally, semantic classification is required for diagrams to be effective. With an abstract technical diagram (or graph) this may be an especially challenging task for individuals who lack domain-specific background knowledge in the subject matter depicted. As a result, the mental representation they construct would be unlikely to progress beyond the visuo-spatial characteristics of the diagram, and this representation may be incapable of serving the purpose intended by the diagram.

In the experiments, meteorologists (experts) appeared to process the material in terms of high level abstract relations and meteorological generalisations. In contrast, non meteorologists' processing appeared to be derived largely from low level visuo-spatial characteristics of the display. These results were interpreted as evidence of different bases for the construction of a mental representation of the weather map information by the two subject groups. Lowe (1993) concludes that if a diagram intended as an aid to learning leads to the construction of a mental representation that fails to capture properly the aspects of the subject matter which have central semantic significance, it is unlikely that the desired learning will be facilitated.

In their model, Carpenter and Shah (1998) contend that graph comprehension involves *bottom-up* processes in which people extract *visual chunks* from the display that are characterised by Gestalt principles, as well as *top-down* processes in which knowledge of semantic content influences viewers' interpretations. Individual differences in expertise (graph reading skills and domain knowledge) interact with bottom-up and top-down influences to affect the kinds of interpretations viewers can make. They found that expert viewers are more likely to make general inferences than novice graph viewers, and novice graph viewers are more influenced by format than expert graph viewers.

While all of this may seem to boil down to a statement of the blatantly obvious, one of the main points in relation to population health publications for wide distribution is that, if charts are to be included, authors should be cognisant of the possibility that readers may lack the necessary domain-specific knowledge to interpret them. If this seems likely, then a sufficient amount of domain-specific information should be included in the text to ensure adequate comprehension of the accompanying chart(s). Additionally, charts should be labelled to provide domain-specific information, including a full explanation of all abbreviations and acronyms: preferably, these should be avoided altogether.

As well as domain-specific support, readers may also benefit from *context-specific* support. For relatively unaccomplished graph readers, simple guidance in reading a chart may be all that is required to ensure that it is understood. Ferry et al. (1999) found that some of their subjects misinterpreted graphs because they did not realise that a relationship existed, and often did not read the graph axes. However, once the researcher directed their attention to the relevant local or global feature, most could correctly answer the questions presented. The implication (again, perhaps obvious) is that accompanying text should spell out the relationships illustrated in the graph so that the two reinforce the relevant message.

The following sections of this review provide specific prescriptions for the effective graphical presentation of data based on consideration of the cognitive and visual processes involved in the interpretation of graphical displays.

3.8 Guiding principles for graph design

> *On rare occasions graphical architecture combines with the data content to yield a uniquely spectacular graphic. Such performances can be described and admired but there are no compositional principles on how to create that one wonderful graphic in a million* (Tufte 1983, p.177).

Tufte is nothing if not passionate about graphics, and perhaps no collation of guiding principles, regardless of how extensive it is, will guarantee the production of that one wonderful graphic in a million. However, for mere mortals struggling with less lofty aims in respect of their graphics, clearly there are empirically tested principles which will substantially improve the chances of a graph being read and understood. And they include some initially mooted by Tufte (1983), in spite of his apparent, and paradoxical, disdain for the practice.

This section presents some general, or over-arching, principles for graphic presentation. Following sections discuss the preferred types of graphs for specific functions, and then individual elements of graphs are dealt with separately.

As a preliminary note, the reader is urged to refer to Kosslyn (1989). This article provides the most thorough and systematic consideration of graphic design found in this literature review, and the principles developed are incorporated into those described below. Kosslyn (1989) develops a method of analysing the information in charts or graphs that reveals the design flaws in the display. The analytic scheme requires isolating four types of constituents in a display, and specifying their structure and interrelations at a *syntactic, semantic and pragmatic* level of analysis. The syntactic rules for constructing useable graphs include a description of the necessary visual elements (e.g. lines, dots and bars) and their perceptual relations, the semantic rules focus on the meanings of the elements of a graph, and the pragmatic rules describe the relation between the information in the graph and the information needs of the reader. As the description is constructed, one checks for violations of *acceptability principles*, derived from established theories about human visual information processing and analysis of symbols. Violations of these principles reveal the sources of potential difficulties in using a display.

The complexity of the article makes it not for the faint hearted, and some brief comments on the difference between the approaches developed by Kosslyn and Tufte may be in order to explain the presentation which follows below in this section. Tufte is very prescriptive: he makes dogmatic assertions regarding the appropriate presentation of data in graphical format, and judgments about the effectiveness of different kinds of graphs. Kosslyn, in general, makes *no* prescriptions, leaving these decisions up to the author who makes judgments about the effectiveness/acceptability of a given display according to whether it adheres to, or violates, multiple specified principles.

For the current purposes of compiling a set of best practice recommendations, a ‘thou shalt’/‘thou shalt not’ approach more in line with Tufte has been taken for the following reasons:

1 Judgments required about the adherence to the specified principles, and the answers to specified questions in relation to each principle, will necessarily be subjective; different individuals may disagree on which principles are violated.

2 Kosslyn’s approach is probably too complex to be practical, even though a practised drawer of graphs may undertake a lot of the recommended tasks anyway – reasonably quickly, though probably largely unconsciously and not as systematically as advocated by Kosslyn.

However, using his own and others' work in the field, Kosslyn's (1994) book rectifies the practicality issue beautifully. Of all texts cited in the bibliography following this review, it contains the most pragmatic and comprehensive guide to constructing graphs and, after struggling through the 1989 analysis, provides the comfort of being easily understood for those of us with considerably less intellect than the obviously very clever Mr Kosslyn.

Another different approach has been undertaken by Bertin (1983, cited and critiqued in Kosslyn 1985 and Spence and Lewandowsky 1990). Bertin developed a comprehensive taxonomy of graphical components and the properties of the perceptual system, and introduced a grammar for the description of graphs. Any graph can be unambiguously reduced to, and subsequently reconstructed from, a description that relies on a small number of grammatical elements. Elements consist of symbols that record the type of variable (continuous or discrete), how it is plotted (line chart, bar chart etc.), whether or not it is cumulative, and so on. An unambiguous description of this type permits efficient storage and transmission of graphical information, and may facilitate predicting performance if the psychological correlates of each symbol can be established. Both Kosslyn (1985) and Spence and Lewandowsky (1990) commend this approach, but agree that Bertin's taxonomy is not exhaustive.

Sumner's internet site (part of the New Mexico State University's home page) provides extensive bibliographic references for *experts* and *newcomers,* as well as practical guidelines for compiling graphs. She too adopts a question and answer type approach to constructing a graph, though it is not directly underpinned by psychological theory in the manner of Bertin (1983), Kosslyn (1989) and others.

Notwithstanding these preliminaries, the following guidelines will facilitate the effective presentation of data in graphical form.

1 Consider the suitability of the data for graphical presentation

As noted in Section 3.3, graphs may not always be the most effective manner in which to present data. The suitability of graphical presentation will depend on multiple factors including the size of the data set, its complexity and structure, the intended use of the display and the characteristics of the audience for whom it is intended.

2 Understand as much as possible about the intended graph users

The underlying design philosophy promulgated by Gillan et al. (1998, p.29) is that graphs should be designed with the potential readers' experience, knowledge and expectations in mind:

1 *Know your users' tasks*

2 *Know the operations supported by your displays*

3 *Match users' operations to the ones supported by your display.*

Gillan et al. (1998) note that software applications for the production of graphs may not support the design of a graph which is adequate for the users' tasks, particularly if the designer uses default settings. In this case they should either learn to use the optional settings on the software, or use a different application that provides the necessary control.

3 Allow graphs to be read on multiple levels by the same, or different, users

Of course, it may not always be possible to be familiar with the characteristics of all of one's users, particularly when the publications in which the graphs are reproduced are widely distributed. In this case Gillan et al. (1998, p.29) contend that it is at least necessary to acknowledge that different readers will have different needs for information as they read a text and look at graphs and tables. *For example, someone with a general interest in a topic but no specific interest may examine a graph holistically to get the main idea or ideas. For that person, the graph serves a communicative function. In contrast, readers who have done extensive work on a topic may examine the data in detail. For them the graph should both communicate the major point and allow them to explore the data to generate their own hypotheses. In addition, some readers may change strategies – from a quick perusal to a detailed examination – during the course of reading. Ideally, the design of a data display should support readers' comprehension of its messages and exploration of its details.*

Tufte (1983, p.13) made the same point earlier, and more succinctly, including in his checklist for the production of graphical displays the requirement that they should *reveal the data at several levels of detail, from a broad overview to the fine structure.*

A key issue here is that a single graph can be designed to support global, integrative tasks by virtue of its emergent features, as well as local, focused attention tasks by emphasising its elemental properties. This is discussed above in Section 3.6.

4 Use graphs, concepts and conventions with which users are likely to be familiar

For documents in wide distribution, Gillan et al. (1998, p.29) recommend the use of *common graphs* with which all readers are likely to be familiar, for example, line and bar graphs, pie charts and scatter plots, *except* when:

1. *The data are normally shown in a certain format...*
2. *Readers are familiar with a certain type of graph...*
3. *Readers need to accomplish a specific task for which the graph is well suited, for example, a stacked bar graph for determining the sum value of several conditions.* (In a **stacked** bar graph the cumulative total of the values shown by the individual components of each bar varies. In a **divided** bar graph the individual components add to 100 per cent).

In consideration of the intended readership, Kosslyn (1989, p.205) specifies that:

1. *Information should be presented in a graph type that is familiar to a given readership...*
2. *A chart or graph should make use of concepts that are likely to be possessed by the intended readership...*
3. *The conventions of a reader's culture should be obeyed: for example, in the western world the colour red should not be used to signify 'safe' areas, and green should not be used to signify 'danger'; time should increase going from left to right or bottom to top...*

Carpenter and Shah (1998) provide direct theoretical and empirical support for point (iii) in respect of the use of colour in graphs. With reference to their own model of graphical perception, they suggest the use of symbols or features that are already associated with a particular referent of value: for example, blue and red colours to represent cold and hot temperatures. See also Section 3.22 on the use of colour in graphs.

In the same vein, Gillan et al. (1998, p.37) recommend that:

1. *The numerical scale on the y axis should go from the lowest number at the bottom to the highest number at the top.*
2. *The numerical scale on the x axis should go from the lowest number at the left of the axis to the highest number at the right.*
3. *The scaling values (i.e. minimum and maximum values and spacing between them) on two adjacent graphs depicting the same variables should be the same.*
4. *Whenever exceptions to the above conventions must be made, they should be clearly and explicitly stated when the figure is first introduced in the text and in the figure caption.*

5 Reinforce the graphical message with accompanying text

In the textual material accompanying a graph readers may benefit from both context-specific and domain-specific support. Each of these types of support has been considered above in Section 3.7. For completeness, major conclusions drawn from that section are restated here:

1. If charts are to be included in publications for wide distribution, authors should be cognisant of the possibility that readers may lack the necessary *domain-specific* knowledge to interpret them. If this seems likely, then a sufficient amount of domain-specific information should be included in the text to ensure adequate comprehension of the accompanying chart(s). Additionally, charts should be labelled to provide domain-specific information, including a full explanation of all abbreviations and acronyms: preferably, these should be avoided altogether.
2. As well as domain-specific support, readers may also benefit from *context-specific* support. For relatively unaccomplished graph readers, simple guidance in reading a chart may be all that is required to ensure that it is understood. This can be provided by spelling out in the accompanying text the nature of the relationships illustrated in the graph so that the two reinforce the relevant message.

Tufte (1983), Cleveland (1994) and Gillan et al. (1998) are strong supporters of thorough explanations to accompany a graph, either in the text or in the caption. Gillan et al. (1998, p.39) stress that *textual descriptions should be clear, accurate, and consistent with the visual representation in the graph*. Specific recommendations in respect of integrating text and graphics are provided in Section 3.14 below.

6 Ensure the information in charts is not ambiguous and does not lead to incorrect inferences

Long term memory is described by Kosslyn (1985, p.507) as *the repository of all that one knows...Hence, displays should not be ambiguous (that is, subject to more than one interpretation) and should not lead one to access inappropriate information (that is, such as occurs when one is led to draw incorrect inferences)*.

To ensure the clarity of a graph Kosslyn (1989) specifies that:

1 Every meaningful difference in the value of a variable is detectable by differences in 'marks' on the graph.

2 Every mark has one and only one meaning.

In spite of his multiple prescriptions, Kosslyn (1989, p.212) acknowledges that *although a chart or graph may convey the correct information...it may invite us to misread it anyway.* Misrepresentation may result from, for example:

- Truncating scales so that small differences appear larger
- Varying the type of scale used
- Using inferred three dimensional properties of a display so that we see bars as bigger than they are.

Tufte (1983) presents and discusses many examples of misleading graphs, pointing out the 'tricks' which have been used to inappropriately illustrate various arguments (see Tufte 1983, Chapter 2, pp.53-77). In so doing, he develops several *principles to enhance graphical integrity* (p.77):

1 *The representation of numbers, as physically measured on the surface of the graph itself, should be directly proportional to the numerical quantities represented.*

2 *Clear, detailed and thorough labelling should be used to defeat graphical distortion and ambiguity. Write out explanations of the data on the graphic itself. Label important events in the data.*

3 *Show data variation, not design variation.*

4 *In time series displays of money, deflated and standardised units of monetary measurement are nearly always better than nominal units.* The point here is that data needs to be comparable. When comparing money measurements over time all values need to be expressed in terms of the same unit (for example, US dollars rather than a combination of, say, Australian, New Zealand and US dollars). Furthermore, the effects of inflation on monetary values needs to be eliminated by deflating them by a relevant price index. In population health statistics the practice of standardising measurement units is relatively common. For example, age-standardised incidence rates allow comparisons over years and between males and females; differences in rates will then *not* be due to differences in the relative proportions of older or younger people in each year or sex. Any adjustments made to the original data for the purposes of comparison and presentation will generally require domain-specific knowledge which, as noted above, should be made available to the reader.

5 *The number of information carrying (variable) dimensions depicted should not exceed the number of dimensions in the data* (the example on page 71 shows the use of three dimensional oil towers to show one dimensional data – oil production).

6 *Graphics must not quote data out of context.*

Some of these points are repeated below in relation to specific graphic elements. Reinforcing his final principle, and Tukey's (1993) point about the use of graphs for the purposes of comparison, Tufte (1983, p.74) states that *to be truthful and revealing, data graphics must bear on the question at the heart of quantitative thinking: "Compared to what?"*

Wainer (1984) also reviews various methods of graphical trickery, effectively illustrating what *not* to do when constructing a graph.

A related issue is that of visual illusions, discussed in relation to axes and scales in Section 3.21.

7 Ensure charts do not contain an excessive amount of information

Kosslyn (1985, p.505) refers to capacity constraints on our short term memory. *Short term memory is defined as working memory in which information can be reorganised and reinterpreted. It can only be stored for a matter of seconds, and only a very limited amount of information can be stored. Thus short term memory is an important bottleneck in information processing, and its limitations must be respected by display designers*. Consequently, he recommends that:

1 *Too much material is not placed in the display.*

2 *Too much material is not in the key, forcing the reader to engage in an arduous memorisation task.*

Schmid (1983, p.33) notes that *a chart overloaded with curves or other symbols can turn out to be self-defeating and worthless, or even worse than worthless, as a medium of visual communication.*

8 Do not over adorn charts

Related to the issue noted immediately above is the need to limit the amount of clutter in a chart, while at the same time ensuring that the intended message is clear and unambiguous, as well as being aesthetically appealing enough to be read.

One of Tufte's (1983, p.96) most basic tenets is to minimise the amount of material in a graph – to show only that which is absolutely necessary for the intended message to be conveyed. Anything else appearing on a graph he describes as *chart junk*, declaring that *the larger the share of a graphic's ink devoted to the data, the better (other relevant matters being equal). Every bit of ink on a graphic requires a reason. And nearly always that reason should be that the ink presents new information*.

Tufte (1983, p.93) defines the *data ink ratio* as *the proportion of a graphic's ink devoted to the non-redundant display of data*. From this syntactic rule he derives two further principles: *erase non-data ink, within reason* and *erase redundant data ink, within reason* (Tufte 1983, p.96 and p.100). As an example of redundant data ink, Tufte (1983) claims that a bar graph containing patterned bars with numerical labels above the bar indicates the value of a variable in similar ways: the height of the right line of the bar; the height of the left line of the bar; the height of the pattern enclosed within the bar; the position of the top line of the bar; the position of the label; and the value of the label. Just one of these, he contends, would be sufficient.

Tufte's recommendation to eliminate *non-data ink* is one of his most contentious, though it certainly has its supporters, notably Wainer (1980) and Cleveland (1994). In reviewing the design of common newspaper graphs used to display statistical information Wainer (1980, p.137), notes that *adorning a graph with chart junk may contribute to its being read, but opens the way to its being misunderstood as well*. Cleveland (1994) stresses the need to make data stand out and avoid superfluity, and warns not to allow other elements of the graph to interfere with the data.

Carswell's (1992a) meta analysis found no evidence to support Tufte's data ink rule. (Meta-analysis refers to the analysis of analyses – the statistical analysis of a large collection of analysis results from individual studies for the purpose of integrating the findings.) However, the study included a variety of experiments in which the data ink ratio may have varied, but was not the focus of the research, and these data ink ratios may have varied sufficiently to produce observable effects on performance. Consequently, Carswell's failure to find support for the data ink principle does not constitute sufficient evidence to reject it.

Nevertheless, specific research in the area of visual search, interaction with graphs, and interaction with quantitative displays, suggests that non-data ink and redundant data ink might improve a graph reader's performance in some instances, but be detrimental to performance in other instances. Criticisms of the principle stem from three sources:

1 The need to entice readers to consider a graph

2 The elimination of all but data ink may inhibit the way in which a chart is perceived

3 Non-data ink may be essential for the graph to fulfil its intended function.

In respect of the first point, Spence and Lewandowsky (1990, p.31) stress that people are more likely to be drawn to attractive, appealing graphs and, conversely, be repelled by dry, sterile depictions of the data: *it is a simple truth that if a graph is not examined, it might as well not have been drawn*. They go on to note that a decorated graph may be better remembered than

a minimalist chart, an issue not considered by Tufte. However, it is also agreed that at a certain point the addition of non-data ink serves no useful purpose, and may actually be harmful, but it is difficult to know precisely when this point has been reached.

In his consideration of perceptual issues associated with graph comprehension and the Gestalt principles of psychology, Kosslyn (1985) points out that graphs which lack chart junk and have a high data ink ratio sometimes violate well known perceptual principles: for example, when axes are not connected to form a frame around a scatterplot, or when box plots retain the whiskers but dispense with the box, the graph does not form an easily comprehended Gestalt image.

Regarding the intended function of a graph, Kosslyn (1985, p.504) notes that *the framework and tick marks [for example] are considered non-data ink, even though they are essential if one wants to know a specific value or magnitude of a difference; both the point on the function and the labelled axes are needed to convey precise data.* From his empirical examinations of the data ink principle, Henry (1993, p.76) urges caution in displaying quantities with empty space, contending that *maximizing the data ink ratio and eliminating as much non-data ink as possible at some point seems to affect the audience's ability to use the graphic accurately*.

In Section 3.6 above a brief exposition of the work of Carswell, Wickens and associates is provided. These authors have examined the effects of integral and separable dimensions in a graphic context, as well as the effects of configural dimensions: that is, perceptually separate dimensions that produce emergent features based on the relationship between the dimensions. Gillan and Richman (1994) cite prior research which suggests that if the dimensions of an indicator are perceptually separable (as in a bar graph), redundant data ink will probably have little effect on a reader's performance under normal viewing conditions. However, if the dimensions are integral (for example, the height and width of a bar), redundancy will probably improve performance, but if the integral dimensions vary independently, it will negatively affect performance. In other words, Tufte's minimilisation principle is not applicable under all circumstances.

In further experiments designed specifically to empirically test the principle in relation to common graphical tasks, Gillan and Richman (1994) found that the effects of ink in the syntactic elements of a graph depend on the location and function of the elements (ink), the users' task, the type of graph, and the physical relations among the graphical elements. Redundant ink in the indicators had limited effects on performance, pictorial background generally increased response time and decreased accuracy, y axis tick marks generally increased response time, and the y axis line (no tick marks) and the x axis generally decreased response time. They concluded that, *rather than indicating that all non-data ink be erased...graph designers need to determine whether ink in a given location will facilitate or interfere with reading the graph. Because...the effects of ink are highly conditional on the features of the graph and task, simple rules like Tufte's will not suffice. The data suggest that a more complex model of the perceptual effects of the elements in graphs will be needed for development of valid prescriptive rules for graphical syntax* (Gillan and Richman 1994, p.639). A perceptual model is developed by the authors and its implications for various graphical features specified. These implications, and the issue of data ink in relation to individual elements of a graph are discussed below under separate headings for the various elements.

Clearly, the literature does not support a universal move toward graph minimalisation but, instead, supports an examination of the functions of graphical ink in the specific task context: judgement calls, it seems, are unavoidable on this issue. Tufte (1983, p.24) does appear to (ultimately) make this acknowledgement, conceding that …*the choice of the best overall arrangement naturally also rests on statistical and aesthetic criteria.* Probably the best and safest approach to take is to start with a relatively minimal presentation and include more only if a strong reason for doing it can be explicitly articulated.

9 Ensure that all 'marks' on a graph are able to be seen, and seen correctly

All marks on a graph must have a minimal magnitude to be detected, and they must be able to be perceived without distortion (Kosslyn 1985 and 1989). Issues of perceptual distortion are considered in Section 3.21 in relation to axes and scales.

Marks must also be relatively discriminable: that is, two or more marks must differ by a minimal proportion to be distinguishable. This is especially important when marks are superimposed on each other (for example, when labels are placed directly on bars) or are in close proximity (for example, when bars are divided into segments, each corresponding to a different independent variable). If colours or different symbols (for example, different sorts of dashed lines) are used to discriminate among bars, lines or wedges, they must be clearly discriminable from each other (Kosslyn 1985 and 1989).

Gillan et al. (1998, p.32) make the following recommendations:

1 *Indicators, verbal labels, and quantitative labels should be made salient in relation to the background by contrasting a light background with dark symbols and characters.* (Note that the default settings for graphics using the Microsoft Excel computer package include a *grey* background. A *verbal label* is a 'written' or 'text' label, and *indicators* are the elements in a graph that express the value of the dependent variable for a given value or category of an independent variable. Examples include plotting symbols and lines in a line graph, bars in a bar graph and pie segments in a pie chart.)

2 *If the lines or plotting symbols in a graph intersect with the axes, consider offsetting the axes to reduce perceptual clutter and impaired symbol detection*. (In this case the axes do not intersect. However, as noted above in guideline (viii), Kosslyn (1985) was not in favour of having axes fail to intersect.)

3 *Make all indicators discriminable from one another by selecting symbols or textures with different features.*

4 *Use large geometric shapes as plotting symbols. Small shapes are difficult to discriminate, especially if the paper has been photocopied.*

Cleveland (1994) also stresses visual clarity. He too suggests the use of different symbols but cautions that they should not overlap; if they do overlap they must be visually distinguishable. The use of a logarithmic scale is suggested to reduce this problem. Both Cleveland (1994) and Kosslyn (1994) also advocate and illustrate the use of a logarithmic scale when it is important to understand percentage change or multiplicative factors, among others. However, log scales may be difficult for the non-mathematically inclined to conceptualise. Consequently, if the author is not *sure* that the audience will understand the technique, it is probably best avoided.

Cleveland (1994) and Gillan et al. (1998) caution authors to consider the potential effects of reproduction and reduction on their graphs: they should be designed with forethought to future copying so that visual clarity is maintained. Cleveland (1994) suggests that shaded areas do not copy well. Gillan et al. (1998) advise avoiding long written labels that extend horizontally beyond the graph.

See also Section 3.15 below dealing with typeface and size, Section 3.24 on line and symbol weight, and Section 3.25 in relation to background features and gridlines.

10 The appearance of words, lines and areas in a graph should be compatible with their meanings

This principle was specified by Kosslyn (1989, p.204) who provided the following examples:

- *The word 'red' should not be written in blue ink*
- *Larger areas in the display should represent larger quantities*
- *Faster rising lines should represent sharper increases*
- *If colours are used, intensity or saturation should covary with hue so that lighter colours correspond to higher values*

The relevance of English language conventions in graph construction is considered in Section 3.18 on labelling, and the use of colour in graphs is discussed further in Section 3.22.

11 Construct graphs so the more important things are noticed first

Kosslyn (1985 and 1989) notes that our visual system detects differences in line weight, orientation and length, shading, colours, and other visual properties, and that larger differences are more easily detected than smaller ones (up to an asymptote). This characteristic leads us to detect:

- Brighter colours before dimmer ones
- Larger bars before more slender ones
- Heavier lines before light ones.

Therefore, graphs should be constructed so that one notices the more important things first. Marks should be chosen to be noticed in accordance with their importance in the display, and the physical dimensions of marks should be used to emphasise the message;

they should not distract from it. For example, inner grid lines should never be darker than content lines, and background patterns should never be as noticeable as the content components of the graph itself.

Gillan et al. (1998, p.33) stress that the *main point* of the graph should be its most visually salient feature, and one should *avoid making the reader search for the main point in the details of the graph.* The reader's attention should be attracted to the data most relevant to the message of the paper.

12 Include only (but at least) the necessary amount of data to make the relevant point(s)

Schutz (1961a), in studying the preferred graph type to illustrate a single trend, found that too many data points, and missing data, on graphic trend displays were important factors in the degradation of operator performance of the required tasks. The implication is to show only the minimal number of data points that will be needed in looking for a trend. In a survey of literature to date, Casali and Gaylin (1988) reported that irrelevant or missing data can significantly increase task completion time and degrade decision accuracy in trend reading tasks. Kosslyn (1989, p.211) also specifies that: *no more or less information should be provided than is needed by the reader.*

13 Require only elementary perceptual tasks

A common finding in the perception literature is that a graph will be less well understood if one has to 'work hard' at understanding it. This principle gets to the heart of issues associated with the nature of cognitive and visual processing, and their application to graphic displays. As such, multiple recommendations are made under this one heading.

From the discussion in Sections 3.5, 3.6 and 3.7 it is noted that the following principles enjoy broad support under most circumstances although, as in any theoretical debate, there are always circumstances under which the rule may not apply:

1. Lengths should be used, as opposed to areas, volumes or angles, to represent magnitudes wherever possible. Most preferable is the plotting of each measure as a distance from a common baseline so that *aligned lengths* are being compared.
2. The task and the display should be compatible in order that perception of the judged characteristic(s) is direct, requiring simpler or fewer mental operations. Global interpretation tasks will be better performed with integrated displays (displays that are high in structural proximity), while tasks requiring local interpretation will be better served by more separate displays (those which are low in structural proximity). In general, the data which are to be compared should be in close spatial proximity.
3. Charts should be configured to produce emergent features to support global interpretation, and the perceptual salience of the elemental features should be increased to support local interpretation.
4. Sufficient context-specific and domain-specific information should be provided for a complete global interpretation of graphs relating to a specific technical field.

Following the work of Kosslyn, Cleveland, the McGills, Carswell, Wickens and others, Gillan and Neary (1992) and Gillan (1994) constructed their *Mixed Arithmetic-Perceptual* (MA-P) model of graph perception. This model clearly illustrates the principle of keeping perceptual tasks as easy as possible, and good practical suggestions for graphic design have emanated from it. It also has the added bonus of *not* stimulating agonising debates about its finer points (the nature of integral, separable and configural displays being a case in point).

The MA-P model proposes that people interacting with common graphs to answer common questions apply a set of common processes: searching for indicators, encoding the value of indicators, performing arithmetic operations on the values, making spatial comparisons among the indicators, and responding. The type of graph and user's task determine the combination and order of the processing steps applied. The model was empirically tested using line graphs, scatter plots and stacked bar charts. In all cases, subjects' response time was linearly related to the number of processing steps according to the MA-P model.

In relation to graph design, the basic implication of the model is to construct graphs which minimise the time required for each of the component processes. Suggested ways of doing this are noted in the sections below dealing with individual graphical elements. Some general suggestions (specified in Gillan 1994, pp.438-439) are as follows:

1 *Design graphs (especially graphs in a series) to have a consistent layout such that the location of indicators will be predictable.*

2 *When possible, use graphs that show the results of arithmetic calculations (for example, a stacked bar graph for addition).*

3 *Organise the task so that users do not have to keep many partial results in working memory, or provide the user with automatic capability for recording and displaying partial results.*

3 *Design the graph or organise the task to minimise the number of arithmetic operations.*

4 *Place indicators in close spatial proximity in the graph, as noted above following the proximity compatibility principle.*

5 *Place the indicators to be compared in the same spatial orientation.*

Using a similar model, Shah, Mayer and Hegarty (1999) propose that graph interpretation involves relatively *simple pattern perception and association processes* in which viewers can associate graphic patterns to quantitative referents, and more complex and error prone *inferential processes* in which viewers must mentally transform data. Their experimentation established that graphs can be redesigned to improve viewers' interpretations by minimising the inferential processes and maximising the pattern association processes required to interpret the relevant information. In addition they found that if relevant quantitative information is perceptually grouped to form *visual chunks* (because relevant data points are either connected in line graphs or close together in bar graphs), then viewers describe relevant trends. If relevant information is not perceptually grouped, viewers are less likely to comprehend relevant trends. In sum, the implication is that data needs to be presented in a way that makes the trends more salient and requires less mental computation by the readers.

(xiv) Graph what is most relevant

This point is really a continuation of the one above. While Gillan et al. (1992) and Gillan (1994) suggest *minimising* arithmetic operations, ideally they should be avoided altogether. Kosslyn (1989, p.197) made this generalisation more eloquently: *displays should not require the reader to decompose perceptual units in order to extract specific points of information.*

An important way in which to make the graph users' task as simple as possible is to ensure that the issue in question is graphed directly rather than indirectly. In the empirical validation of their model, Shah, Mayer and Hegarty (1999) found that charts were relatively inaccurately or incompletely interpreted when subjects had to rely on complex inferential processes which involved quantitatively transforming the information in the display: for example, calculating the differences between two or more data points. In this case, where the focus is on the difference between two functions, a single line showing the difference should be drawn, rather than the two original functions. In his textbook, Cleveland (1993, p.22) advocates the use of a *Tukey mean-difference plot* to assist the visual evaluation of two distributions. In this type of chart the difference between the two data sets is plotted against their means, and has the perceptual advantage of allowing relatively easy judgement of deviations from a horizontal line.

Cleveland, McGill and McGill (1988) demonstrate that the slope of a curve will be influenced by the overall *shape* of the graph. Therefore, if the slope, or rate of change, of a function is most important, the rate of change should be plotted rather than the original data.

Tversky and Schiano (cited in Spence and Lewandowsky 1990) found that subjects remembered almost symmetric curves as being more symmetric than they actually were. This, they contend, probably reflects the fact that, since we often view symmetric objects off centre, we have a tendency to correct our perception toward symmetry. Thus, if the important thing is the departure from symmetry, it may be better to display the deviation rather than the asymmetric curve itself.

When there is more than one independent variable to be considered, the most important one should be on (and label) the x axis, and the others should be treated as parameters representing separate bars or lines. The major design principle identified in studies by Carswell, Wickens, Gillan, Shah and others is that, to use Shah's terminology, visual chunks in graphs should relate the data points that the author wishes the reader to compare. For example, if comparisons between categories *within a given year* are to be described, then an appropriate format would be sets of bars grouped such that each cluster (that is, each visual chunk) consisted of x number of different categories within a given year. To describe comparisons of a *single category across years*, an appropriate format would be

the use of dots connected by lines such that each line (each visual chunk) consists of a measurement of a single category across years. In other words, perceptual organisation of the data is an important factor that influences viewers' spontaneous interpretations and understanding of it, even when the data and tasks are relatively complex and the domains are unfamiliar. Instructive examples relating to this issue are provided in Kosslyn (1994, p.11 and p.71) and Shah et al. (1999, pp.691-692).

If there is no clear distinction between the importance of the variables, Kosslyn (1994) recommends putting an interval scaled independent variable (if there is one) on the x axis. (An interval scaled variable conveys information about the ordering of magnitude of the measures and about the distance between the values, but does not have a true zero point. For example, the measurement of temperature on either the Fahrenheit or Celsius scales and IQ test scores.) The progressive variation in heights from left to right will then be compatible with variation in the scale itself. If there is more than one independent variable with an interval scale, Kosslyn (1994) suggests that it is best to put the one with the greatest number of levels on the x axis. Paraphrasing his example, if the task is to graph the number of people of different ages who voted Democrat in a specified electorate in particular years, year should be shown on the x axis if there are ten years and only two age groups, and vice versa if there are two years and ten age groups. *This procedure cuts down the number of separate content elements and thereby reduces the load on our short term memory capacities* (Kosslyn 1994, p.72). Where none of the foregoing recommendations apply to a particular case, the final suggestion is to put on the x axis the independent variable that allows the simplest pattern of content elements.

Depending on the purpose for which graphs are designed, different graph formats are more or less appropriate. The use of specific types of graphs for specific tasks is discussed in the following three sections, dealing with the most common types of graphs, and those most frequently used in population health publications: pie charts, line graphs and bar graphs. Brief mention is then made of scatter plots.

The remaining sections consider individual elements of a graph. Kosslyn (1994, p.276) cites multiple references for the discussion of less well known, or special purpose, displays, but notes that:

- *Although some of these exotic displays may be better than conventional displays in laboratory tasks, they may fall short in more natural settings...*
- *… Conventional displays have survived a kind of Darwinian winnowing process, and the mere fact that they continue to be employed over so many years...is itself evidence of their utility.*

3.9 Pie charts

1 The indigestible pie

The pie chart is probably the most maligned of all graphical forms, particularly by Tufte (1983, p.179) who described it as *dumb*. He is both contemptuous and scathing in criticising its use, contending that *the only worse design than a pie chart is several of them…Given their low data-density and failure to order numbers along a visual dimension, pie charts should never be used* (Tufte 1983, p.178). Others agree, notably Cleveland and McGill (1984 and 1985) who demonstrated that perception of position along a common scale (bar charts) is ranked higher than perception of length (divided bar charts) which in turn is ranked higher than perception of angle (pie charts). See Section 3.5 above on perceptual issues relating to graph comprehension.

2 Slices of the pie

Some, however, contend that the pie chart is nowhere near as stale as others would have us believe. While Kosslyn (1985 and 1994) acknowledges that the utility of pie charts is limited because the human visual system distorts area and imprecisely registers angle, he nevertheless promotes their limited use, particularly to provide an immediate impression of how parts form a whole. Examples of how to, and how *not* to use pie charts are provided in Kosslyn (1994, pp. 24-28).

The issue of how parts form a whole is taken up in experimental work by Culbertson and Powers (1959), Simpkin and Hastie (1987), Hollands and Spence (1992) and Hollands (1992) in consideration of different types of tasks which graph users may undertake. They distinguish between *part-to-whole* judgments involving

comparison of one proportion of an item to its whole, and *part-to-part* judgments involving a decision on what proportion a smaller value is of a larger, where the smaller value does not form part of the larger one. Pie charts (as well as divided bar graphs – bars of equal height with proportions summing to 100 per cent) were demonstrated to be *more* effective than line or bar graphs for tasks requiring part-to-whole judgments: that is the Cleveland and McGill ordering is *not* appropriate for this particular type of task. Hollands and Spence (1992) reasoned that, in making part-to-whole judgments using a pie chart, two physical objects are being compared – the slice and the pie. The pie chart is effective because both quantities are immediately available to the perceptual system. They note that most other graphical forms do not allow the observer to view proportion directly – it can only be deduced after some intermediate and mental computation. Zacs and Tversky (1999) confirmed this finding, both empirically and through a search of the literature.

On the other hand, Simpkin and Hastie (1987) and Hollands and Spence (1992) found that pie charts were not effective for part-to-part comparisons, as would be the case when *changes* in the magnitude of a variable are to be detected. In the Hollands and Spence (1992) experiments, change was judged more quickly and accurately with line and bar graphs than with a sequence of pie charts or tiered bar graphs (separate charts, vertically aligned below each other). This difference was larger when the rate of change was smaller. In this case the authors reasoned that observers can directly perceive change from the slope of a line. Although the direction and magnitude of change could be deduced from a sequence of pies by discriminating the sizes of segments, change could not be perceived directly.

In terms of the proximity compatibility principle, judging proportion (part-to-whole comparisons) requires extracting one piece of information from the graph and is thus a focused attention task, best served by a separated display. Part-to-part comparisons effectively involve making judgments of change: an integration task requiring an integrated display such as a line graph.

Schmid (1983) cautions that the more the form of a pie chart deviates from a circular shape the more likely there will be distortions in viewing the various sectors.

3 Freshening up the pie

In their consideration of part-to-whole judgments, Simpkin and Hastie (1987) introduced the notion of *anchoring* to construct a model of graphical perception. Anchoring involves segmenting a component of the image that is a standard for some estimate; this segment will then act as an anchor, providing an initial value that is adjusted to yield the part-to-whole estimate. When making a part-to-whole judgement, the more accurate anchoring possible with position along a common scale (simple bar chart) and angle (pie chart) visual codes accounts for their superiority over the length (divided bar chart) code. Although processing angles is more difficult than processing linear aspects, this judgement for the pie chart is a special case in which the anchors are the perceptually salient angles of 0º, 90º and 180º. When making a part-to-part judgement, the position code is superior to the other two codes. Length again suffers from less accurate anchoring. Angles provide the least accurate estimates because of the inferior anchoring when these anchors are no longer at perceptually salient angles.

Gillan and Callahan (2000) built on the model of graphical perception developed by Simpkin and Hastie (1987) to propose a useful method for freshening up the pie chart, and thereby increasing its useability. They found clear support for a three component model of reading a pie graph to estimate the size of a specified, or target, segment. The proposition is that the reader will:

1. *Select* a mentally represented anchor segment – people use 25 per cent, 50 per cent or 75 per cent as these translate into pie segments that have either straight lines (50 per cent) or lines at right angles (25 per cent and 75 per cent). This anchor segment is compared with the target segment.
2. Mentally *align* representations of the anchor and target segments – this might involve mentally *rotating* the anchor segment to align it with a target segment to facilitate the comparison. Both Simpkin and Hastie (1987) and Hollands (1992) observed a greater response time when participants compared segments in two pie graphs if the segments were unaligned rather than if they were aligned. Gillan and Callahan (2000) contend that the extra time is consistent with the need for a pie graph reader to mentally rotate one segment to put it into alignment with another. A similar rotation, or *superimposition,* process was proposed by Simpkin and Hastie (1987).

3 Mentally *adjust* the size of the anchor to match the target, and so estimate the size of the target segment.

The model was supported by empirical testing, first by examining performance using regular pie graphs, and then by creating modified (or as Gillan and Callahan (2000) rather more pretentiously prefer – *cognitively engineered*) pie graphs based on the model and testing specific predictions regarding performance with one type of graph compared with the other.

In using regular pie charts, subjects were better able (in terms of accuracy and speed) to estimate the size of segments the closer in size they were to the 25 per cent and 50 per cent hypothesised mental anchor sizes, and the more vertically aligned they were. The implication is that, as suggested by the model, we view pie segments by aligning the target and anchor segments using mental rotation, and mentally adjusting the size of the target to match the anchor (or vice versa).

Estimation times were reduced for cognitively engineered pie charts which consisted of a series of aligned pie segments (the whole pie was *not* shown), all vertically aligned. The theory is that, because all of the segments were aligned in the vertical position, the need to apply the align component was eliminated. Moreover, the data suggested that pie graph readers do not need configural cues which may be present with the *whole* pie to estimate the proportional size of a segment. Nor do they need an additional time consuming processing step that serves to sum the various segments or reorganise them into a whole: they either accepted that the pie segments summed to a whole, or could rapidly determine that the segments combined to a whole pie.

Gillan and Callahan (2000) cite Hollands and Spence (1992) in noting that participants using separated bars in a bar graph to estimate a proportion *did* apply a summation processing step. However, they caution that their results do not suggest an advantage for the aligned pie graph over the regular pie graph for all tasks: the aligned pie may produce poorer performance in tasks in which global processing of the pie is critical, or that explicitly require the reader to make part-to-whole judgments.

3.10 Line graphs

1 Line graphs for showing single trends

Line graphs have been found to be most appropriate for showing data trends and interactions, though bar graphs also receive some support for this task. Zacs and Tversky (1999, p.1074) describe *trends* in terms of *rising, falling, increasing, or decreasing.* In terms of the proximity compatibility principle, judging change is an information integration task since it requires comparing different quantities and integrating that information. Since the line graph is an integrated display, the task and the display are compatible. The literature is divided over the preference for lines or bars to facilitate the extraction of exact values, though it probably comes down in favour of bars.

The use of line graphs in preference to bar graphs for showing current, and predicting future, trends, displaying interactions among variables, and identifying global patterns in data is supported by Washburne (1927, cited in Meyer 1997), Schutz (1961a), Pinker (in press at the time, cited in Kosslyn 1985), Kosslyn (1989 and 1994), Sparrow (1989), Gillan et al. (1998), Zacs and Tversky (1999), and Shah et al. (1999), and in literature reviews by Casali and Gaylin (1988) and Spence and Lewandowsky (1990).

Generally, line graphs will depict *continuous independent variables* such as time or age (see Kosslyn 1989 and 1994, Gillan et al. 1998 and Zacs and Tversky 1999). However, Kosslyn (1989 and 1994) contends that most people find line graphs better than bar graphs for the portrayal of *interactions* and *meaningful patterns* when the explanatory variable is *categorical*, even though the use of a line may be said to imply continuity of the explanatory variable (for examples see Kosslyn 1994, p.32). Gillan et al. (1998) support the use of a line graph when interaction is the main focus of the chart, particularly if the independent variable contains more than two levels.

Shah et al. (1999, p.701) determined that line graphs emphasise x-y trends, so that *if there are three or more variables in a data set, then the most important relationship should be plotted as a function of the x and y axes*. However, because line graphs were found to emphasise x-y relations they were said to be *more biasing* than bar graphs. Consequently, *if two independent variables are equally important, bar graphs should be used. If a particular trend is the most important information, then line graphs should be used.*

Carswell and Ramzy (1997) found that line graphs tend to show the greatest sensitivity to increases in data set complexity, and the greatest overall trade-off of local for global content with increased departures from linearity in the data.

2 Line graphs for showing multiple trends

When *multiple trends* are to be compared, Schutz (1961b) determined that showing several trend lines on a *single* graph was superior to presenting single trend lines on several graphs. For reading exact points, the multiple line or multiple graph display were found to be equally effective. Where both comparison and point reading tasks were involved, then the multiple line display yielded superior overall performance.

Both Casali and Gaylin (1988) and Spence and Lewandowsky (1990) also conclude that the literature supports the use of multiple line (single) graphs when several time series are to be compared simultaneously. Casali and Gaylin (1988) and Sparrow (1989) opt for multiple graphs with single lines on each for point reading tasks, though generally the literature supports the use of bar graphs if specific quantities must be estimated.

Kosslyn (1994) suggests that layer graphs are most useful to illustrate the relative change in one component over changes in another variable. Because the spaces between the lines can be filled, they can be seen as shapes, and the change in a single proportion can be easily seen. He cautions that layer graphs should only be used to display continuous variables: that is, values on an interval scale. If the x axis is an ordinal scale (one that specifies ranks) or nominal scale (one that names different entities) the eye will incorrectly interpret the quantitative differences in the slopes of the layers as having meaning. In these cases the use of a divided bar graph is recommended.

In addition to displaying trends Sparrow (1989) advocates the use of single, multiple line graphs for indicating data *limits* (maxima and minima – for example, the year in which product A's sales peaked; and *conjunctions* (the intersection of two exemplars – for example, the year in which product A first sold more than product B).

3.11 Bar graphs

Consensus appears to be that line graphs are to be most preferred for showing trends, but bar graphs run a close second, as long as they are vertical, not horizontal. Bar graphs are also preferred if precise values need to be detected, and they are a good 'compromise' if both local (or *discrete*) and global interpretations of the data need to be made. Zacs and Tversky (1999, p.1074) describe *discrete comparisons* in terms of *higher, lower, greater than, or less than.*

1 Vertical and horizontal bar graphs

While Schutz (1961a) determined that line graphs were preferred for showing trends, *vertical* bar graphs were also considered to be effective. However, *horizontal* bar graphs were found to be relatively ineffective for depicting trends. Schutz speculated that the superiority of the line and vertical bar graphs over the horizontal bar graph may be partly attributed to the fact that the two axes are reversed for the horizontal version: that is, time is on the vertical axis and number is on the horizontal axis.

Culbertson and Powers (1959) found a slight preference for vertical over horizontal bar charts in their experiments involving discrete comparisons. Casali and Gaylin (1988) also cite evidence to support the contention that vertical bar graphs are faster to use and result in higher accuracy than horizontal bar graphs for determining trend data, and are faster to use for the detection of certain types of out-of-tolerance conditions. Their literature search also indicated that modified, or *stroke graphs*, showing the top line of the bars in a bar graph, are faster to use, and in some instances, result in higher accuracy than T-type or bar-type graphs for the detection of out-of-tolerance data.

While Kosslyn (1994, p.38) favours subjective consideration of the data to determine a preference for either horizontal or vertical bars, his final recommendation is: *when in doubt, use a vertical bar graph format*, since increased height may be considered a better indicator of increased amount.

Kosslyn (1994) also considers the use of 'side-by-side' horizontal bar graphs which show pairs of values (values for two independent variables) that share a central y axis. He recommends the use of this type of chart to show contrasting trends between levels of an independent variable, and comparisons between individual pairs of values, and provides some 'do and don't'

recommendations for their construction (Kosslyn 1994, pp.40-43). These types of graphs are frequently used in population health statistics in the form of 'population pyramids' which show, for example, the distribution males in a given population (on one side) and females (on the other side) in each of specified age groups.

2 Bar graphs for detecting specific quantities

Bar graphs are generally regarded as superior for depicting *categorical independent variables* and making discrete comparisons of absolute or relative amounts (Washburne 1927, cited in Meyer 1997, Culbertson and Powers 1959, Kosslyn 1989 and 1994, Spence and Lewandowsky 1990, Greaney and MacRae 1997, Gillan et al. 1998 and Zacs and Tversky 1999).

Gillan et al. (1998) also specify the use of a bar graph if readers need to determine the difference between the means of the dependent variable across different levels of the independent variable, and either a bar graph or a line graph to represent an *ordinal* independent variable. Sparrow (1989) demonstrated the effectiveness of bar graphs (along with line graphs) for displaying data limits (maxima and minima), and the advantages of some types of divided bar charts for displaying *accumulation* (the summation for each exemplar of one variable across another – for example, to determine which of several products sold the most overall).

Gillan et al. (1998) also advocate the use of a line graph for determining absolute or relative amounts, but this is a minority opinion.

3 Bar charts for showing proportion

Cleveland and McGill (1984 and 1985) criticised pie charts because they require judgments of angle, and divided bar charts because they require judgments of length and position, none of which can be judged on a common scale: angle, length and position on identical but non-aligned scales are relatively less preferred in their rank ordering of graphical specifiers (see Section 3.5). In the first instance they advocate replacement of a pie chart with a divided bar chart, thus replacing angle judgments by position judgments. They suggest (though the suggestion is not empirically tested) that in most cases the scale should go from 0 per cent to 100 per cent so the viewer can more readily appreciate the fraction that each bar is of 100 per cent, but also say that 0 per cent to 25 per cent, or 0 per cent to 50 per cent, are also reasonable ranges.

Most preferred by Cleveland and McGill (1984 and 1985) are dot charts to pie charts, and grouped dot charts to divided bar charts, because they display position along a common scale and eliminate the less accurate length judgments. (Examples of dot charts and grouped dot charts are provided in Cleveland and McGill 1984, pp.547-548, Cleveland and McGill 1985, p.829 and p.831 and Cleveland 1994, pp.149-151, but note that the efficacy of this type of chart was not tested experimentally by the authors.)

Hollands and Spence (1992) and Hollands (1992), found that divided bars graphs (bars of equal height with proportions summing to 100 per cent) were *more* effective than line or *typical* pie charts for task requiring part-to-whole judgments. In making general recommendations, Gillan et al. (1998) cite pie charts and divided bar charts as being equally effective in determining proportions. See Section 3.9 above on pie charts.

4 Bar graphs for displaying trends

While Hollands and Spence (1992) found judgments of change to be more effective with lines than bars, bar charts performed much more effectively than a series of pies. They hypothesised that judgments of change were faster and more accurate with bars than with pies because subjects imagined a *virtual line* connecting the tops of the bars, the slope of which could be used to judge change in the same way the slope of a physical line is used in a line graph. In terms of the proximity compatibility principle, an aligned bar graph which is otherwise considered a separated display, can serve as a useful display for an integration task because its virtual lines serve as emergent features which can be viewed directly. This view is supported by further theoretical development and empirical testing of the principle by Bennett, Toms and Woods (1993) and Greaney and MacRae (1997).

Shah et al. (1999) determined that bar graphs emphasise comparisons which are closer together on the display, so if there are three or more variables, the most relevant trends should be plotted closer together along the axes when using bar graphs. They also concluded that bar graphs may be better suited when the relationships of two independent variables are to be equally emphasised.

5 Bar graphs for multiple functions

From the review of the literature it appears that bar graphs are an adaptable type of display, which may be used either to provide specific information about individual data values (local content) or for more global interpretive purposes. Carswell and Ramzy (1997) suggest that this may be the case because bar graphs support the development of very different types of graph reading strategies. They hypothesise that some subjects tend to read the individual data values and then mentally compare or integrate them in some way: that is, they adopt a point reading strategy followed by mathematical operations. For such subjects graphs that promote access to individual data values may be used more effectively. Line graphs would not be well suited for those adopting this strategy since lines are generally used less efficiently for point reading tasks than other, more separable, formats.

3.12 Scatter plots

A scatter plot is generally used if readers need to determine the degree of *correlation* between two variables (Gillan et al. 1998).

Experiments by Spence and Lewandowsky (1990) on the perception of scatter plots suggest that human observers are conservative judges of correlation, tending to estimate the square of the correlation rather than the correlation itself. Reducing the size of the point cloud relative to the axes will lead to less conservative – and therefore more accurate – judgments, as will even limited training.

Multiple groups, or strata, are often shown together in a single scatter plot to allow comparison of different subgroups with respect to a common set of variables. An observer must be able to discriminate the strata if the display is to be effective, and intuitive impressions that some types of symbols are easier to discriminate than others are strong. Cleveland and McGill (1984) proposed a rank ordering of symbol types, suggesting that different colours produce optimal performance, followed by amounts of fill, then different shapes, and finally letters. Lewandowsky and Spence, cited in Spence and Lewandowsky (1990) recommend different colours, followed by equally discriminable sets of letters (those with few shared perceptual features), shapes or amounts of fill.

In contrast, if the points in a scatter plot overlap Kosslyn (1994, p.154) favours the use of larger dots, rather than a different symbol, on the basis that *a larger dot better conveys the impression that there is more of something than does the difference between a triangle and a circle.* He also suggests (p.46) that it is usually better to plot different independent variables in *different* scatter plots. The only exceptions to this recommendation are:

1 *Where the purpose of the chart is to show that the data from two independent variables are intimately related, and so the fact that the points cannot be distinguished is itself compatible with the measure being displayed.*

2 *If the clouds formed by different sets of points can be easily distinguished because they are different in different parts of the display.*

In addition, Kosslyn (1994) recommends the inclusion of a line of best fit in scatter plots to assist the reader in discerning the degree of correlation between the variables.

3.13 The display of multiple graphs

Kosslyn (1994) suggests (and cites supporting references on p.281) that, because of our limited short term memory capacity, where there are more than **four** groups of lines on a chart, or **four** bars over each point, it is best to divide the data into subsets and graph each subset in separate panels of a display.

To aid search across multiple graphs with many levels of an independent variable, Gillan et al. (1998, pp.33-34) suggest, and provide examples of, the following:

1 *Place all graphs in one figure to facilitate search across graphs.*

2 *Use spatial proximity for graphs so that the reader might search in sequence...*

3 *Maintain visual consistency across graphs (e.g, use the same size axes, the same types of indicators, and the same coding of indicators for the same variables).*

4 *Maintain semantic consistency across graphs (e.g, use the same scale on the y axis).*

5 *Eliminate redundant labels (eg in a series of horizontally aligned graphs, the labels for the y axes should be placed only on the leftmost graph, and the verbal label naming the x axis should be centred under the series).*

6 *Avoid using a legend to label indicators (a legend requires multiple scans between the indicators and*

the legend, thereby disrupting visual search).

Direct labelling in preference to a legend is discussed further below in Section 3.20.

Kosslyn (1994, pp.194-204) adds the following, summarised here but substantially elaborated upon in his textbook:

1 Assign data that answer different questions to different panels.

2 Assign lines that form a meaningful pattern to the same panel.

3 Put the most important panel first.

It should be noted that these recommendations are relevant in the absence of any other overriding considerations. For example, the logical ordering of panels may be suggested by categories of the data being displayed, such as chronological age categories.

The remaining sections consider individual elements of a graph.

3.14 Integrating graphics and text

There is no dispute that graphs, relevant text and, where appropriate, statistical analysis in a document should be integrated in close physical proximity. Tufte (1983, p.181) spells out the following recommendations:

1 *Data graphics are paragraphs about data and should be treated as such...Tables and graphics should be run into the text wherever possible, avoiding the clumsy and diverting segregation of "See Fig. 2" …If a display is discussed in various parts of the text, it might well be printed afresh near each reference to it, perhaps in reduced size in later showings.*

2 *The same typeface [should] be used for text and graphic.*

Kosslyn (1989, p.212) explicitly supports these recommendations, adding *that the terminology used in the display should be the same as that in the text or presentation*.

Based on his *model of meaningful learning* as well as experimental studies over a number of years, Mayer's (1993) findings are also consistent with Tufte's recommendations. Mayer (1993, p.243) repeatedly found what he calls *a contiguity effect – students tend to build useful mental models (as measured by good problem solving performance) when the visual and verbal information is presented contiguously in space (for example, words are present in the illustrations) or time (for example, verbal speech is simultaneous with animation).*

Gillan et al. (1998, p.38) specify that information in the graph should be consistent with that in the text, and make the following recommendations (specifically intended for authors submitting articles for publication in scientific journals):

1 *Label the y axis with the name of the dependent variable used in the text.*

2 *Label the x axis with the name of the relevant independent variable used in the text.*

3 *Labels for indicators should use the same names as those in the text for the levels of the relevant independent variable.*

4 *The relations shown between the independent and dependent variables should both relate to the hypothesis described in the paper's introduction and reflect the analyses described in the results section.*

5 *Refer to the graph in the text early in the section in which the data are being described. Do not cite it only after describing the data.*

6 *To the extent possible, coordinate the text and graph so that the same printed page will contain the graph(s) and text that describe the same data...* This is in accordance with Tufte's recommendation noted above.

The authors make further recommendations in relation to consistency for articles which present a series of related experiments, as well as non-related experiments. The intention is to better allow the reader to perceive the meaning of the experiments and facilitate relevant comparisons (Gillan et al. 1998, pp.38-39).

3.15 Typeface and size

Tufte (1983, pp.180-181) believes that *the size of the type on and around the graphics can be quite small, since the phrases and sentences are usually not too long – and therefore the small type will not fatigue viewers the way it does in lengthy texts*. His guiding principles (p.183) in this respect are:

1 *Type is clear, precise, modest...*

2 *Type is upper and lower case, with serifs.*

The serif font currently in most frequent use is Times New Roman, as used in this sentence. Arial, as used in this sentence, is a sans-serif font, characterised by less 'decorative' letters.

On the upper/lower case, serif/sans-serif issue, Albers 1936 (cited in Tufte 1983, p.183): *the more the letters are differentiated from each other, the easier the reading…words consisting of only capital letters present the most difficult reading – because of their equal height, equal volume and, with most, their equal width. When comparing serif letters with sans-serif, the latter provide an uneasy reading. The fashionable preference for sans-serif in text shows neither historical nor practical competence*. Kosslyn (1994) provides recommendations in respect of lettering consistent with those of Tufte and Albers, though he notes (with supporting references) that *there is no good evidence that serifs consistently aid or impair reading under normal reading conditions* (Kosslyn 1994, p.283). A section on 'Guidelines and Checklists' for evidence based web design, listed on a website for the National Cancer Institute (date unknown), supports Kosslyn's assertion.

Since judgments in respect of *small* and *large* typeface are both relative and subjective, recommendations in these terms are not immediately helpful. In contrast to Tufte (1983), Gillan et al. (1998) say that indicators, verbal labels, and quantitative labels should be *large*. They recommend avoiding thick bold lines or unusual fonts that might interfere with readers' ability to discriminate between the letters. They also support the use of upper and lower case letters so that readers can make use of shape cues to recognise words.

Little work appears to have been done in respect of the *precise size* of typeface. In a field study involving some 2,000 measures for over 300 printed displays, Smith (1979) tested the legibility of letter size in terms of *radians*: a measure of the subtended visual angle calculated by dividing the letter height by the viewing distance. He found a mean letter height of .0019 radians at the limit of legibility, with over 90 per cent legibility at .003 radians and virtually 100 per cent at .007 radians. He contended that letter sizes corresponding with the lower end of this range could be used with little loss in legibility when more compact display formats are required. However, no guidance was given regarding the translation of these findings into specified font sizes.

See also Section 3.8, guideline (ix) concerning the visibility of 'marks' on a graph.

3.16 The shape of a graph

Tufte (1983) felt that graphics should tend toward the horizontal, greater in width than in height (various reasons for this preference are expounded on pp.186-190). He contended (p.190) that *if the nature of the data suggests the shape of the graphic, then that suggestion should be followed; otherwise it is preferable to move toward horizontal graphics about 50 per cent wider than tall.*

Cleveland and the McGills reach pretty much the same conclusion, but not before torturing the reader in the process. Cleveland and McGill (1987) note that our perception of the slope of a line can vary with the shape of a graph, and refer to its *shape parameter*: defined by the rectangle enclosing the data display and calculated as the ratio of the graph's height and width (or the slope of a line joining the lower left corner and the upper right corner of the data rectangle). They note that there is no consensus on what the ratio should be and, after a very complex theoretical analysis, come to the rather unsatisfying general conclusion that *where the analysis depends heavily on making slope judgments, these judgments are enhanced by the choice of the scales and the shape parameter* (Cleveland and McGill 1987, p.208).

Not content to leave it there, the intellectual capacity of the other McGill is added to the team (Cleveland, McGill and McGill 1988). After much ado, a criterion by which to specify the optimum shape parameter to use in a two variable graph (which shows the dependence of y on x) is determined: the orientation of two line segments with positive slopes is maximised when the shape parameter is chosen to make the average of the two orientations, which they call the *orientation mid angle*, equal to 45° (or -45° for two line segments with negative slopes). Little practical advice is given as to how to determine the orientation mid angle when graphing, for example, an extended non-linear time series, except to say that *it would probably even suffice to make a rough choice visually as the shape is varied*. In the end they conclude that *at best we can only get a rough visual estimate of rate of change from slope judgments, even in the best of circumstances...when the orientations are optimised. In examples where studying the rate of change with more accuracy is needed, it is important to graph rate of change directly so that values can be visually decoded by more accurate judgments of position along a common scale* (Cleveland, McGill and McGill 1988, p.299). This had already been determined in earlier work

by the same the authors in 1984 and 1985. Cleveland (1993) continues this discussion in his textbook, referring to the general issue of selecting an appropriate shape for a graph as *banking.*

Kosslyn (1994, p.66) offers the most pragmatic advice and, like Tufte (1983), leaves decisions about the shape of a graph up to its author. He advises that the shape parameter (or what he refers to as the *aspect ratio*) should be adjusted so that *actual differences in the data produce corresponding visible differences in the display*.

A related issue is that of graphing the item which is of most interest, elaborated upon in Section 3.8, guideline (xiv).

3.17 Captions

Leading writers in the field have generally recommended that chart captions (titles) fully describe the contents of the chart, though note, however, that of the three considered in this section, none cite experimental validation of their suggestions.

Specifically, Schmidt (1983) prescribes that the chart caption (title) should answer three questions relating to: what, where, and when. Cleveland (1994, pp.54-55) recommends making captions *comprehensive and informative,* and suggests that they should:

1 Describe everything that is graphed
2 Draw attention to the important features of the data
3 Describe the conclusions that are drawn from the data on the graph.

These recommendations are largely consistent with those of Kosslyn (1994, p.21), who believes that the caption should answer two questions:

1 *What do you want your readers to know after examining the display?*
2 *What information will they need?*

He also suggests that formulating the title *prior* to constructing the graph is a useful way to decide on its contents.

In respect of the appearance of the caption, Kosslyn (1994) recommends that the title should be:

1 The most salient element of the display. Therefore, a larger or different font should be used to set it apart.
2 Centred at the top of the display.

3.18 Labels

1 Text labels

Tufte (1983) was very much a proponent of labelling graphs, though he did not consider the issue of whether labels could be considered non-data ink (see Section 3.8, guideline (viii)). Both Tufte (1983) and Kosslyn (1985) favour the use of clear labels to explain the data, or indicate important events in the data, as well as to make the graph clear and unambiguous.

2 Numerical labels

Culbertson and Powers (1959), Bennett and Flach (1992) and Gillan (1994) recommend placing numerical values directly onto a graph to aid focused attention tasks. (Numerical labels also include scale values associated with the axes. These are discussed immediately below and in Section 3.21.)

3 Suggestions in respect of labelling

Kosslyn (1985) and (1989) applied Gestalt laws to make recommendations in respect of labelling. He suggests that a label should be *closest* to the part it is labelling. In noting that, for line graphs, content lines that cross can sometimes be confused if the segment from one flows naturally from the segment of another, he suggests that lines should be labelled at the ends, and the label should be a continuation of the line itself.

In respect of labelling in general, and labelling axes in particular, Gillan (1994, pp.438-439) recommends the following (based on the MA-P model – see Section 3.8, guideline (xiii)) to minimise the time required to search for an indicator and/or encode the value of an indicator:

1 *Clearly associate verbal labels with indicators (or axes) by use of proximity or, if proximity is not possible, by similarity.* Gillan et al. (1998) elaborate with the recommendation to use similar patterns or shapes to indicate data from similar conditions in a study.
2 *If the users' tasks involve arithmetic operations, place the indicators close to the numerical labels. If the tasks involve frequent arithmetic operations, place the numerical values of the indicators immediately above or next to the indicators. Otherwise if the tasks will involve less frequent arithmetic operations, place the indicators close to the y axis and associated numerical labels.*

3 *For graphs that are wide, place Y axes and the associated numerical labels on both sides of the data. Users will be able to encode the value of indicators on the right side of the graph by using the right-hand y axis.*

4 *Have the scale of numbers next to the y axis finely graded to allow users to estimate values easily. However, do not have the numbers so finely graded that one number interferes with reading another or that there is not a clear relation between a point on the axis and the number.*

Gillan et al. (1998, p.34) provide good visual examples of these recommendations.

The suggestion to *finely grade* the y axis scale (point (v) above) is perhaps an over prescription if it is not essential to estimate values. On this issue, Kosslyn (1994, p.97) is less prescriptive, specifying that tick marks should be labelled at *regular intervals*, using *round numbers*. Where the range of values on an axis dictates that labels should include decimal points to be meaningful, the number of decimal places should be kept to a minimum. He also suggests that axis labels should be centred and *parallel* to their axis. That is, the y axis should *not* be labelled horizontally on the top left hand side of the data rectangle.

It is noted that Cleveland (1994) cautions that we should not allow data labels in the interior of the scale-line rectangle to interfere with the quantitative data or to clutter the graph; if they are unavoidable, they should not dominate the pattern of data. He recommends abbreviations inside the graph area if they can reduce clutter in the graph. However, this is contrary to the recommendation made above that domain-specific technical terms should not be abbreviated if the graph reader is not an expert in that domain (see Section 3.7).

4 English language conventions

Kosslyn (1989, p.204) and (1994, pp.88-100) has made multiple recommendations in respect of labelling the elements of a graph. While some of them are pretty much statements of the obvious (for example, that the labels should be large enough to be read), and/or have been covered in other sections of this document (see Sections 3.15 on typeface and size, 3.17 on captions, 3.20 on legends and keys, and 3.21 on axes, scales and tick marks) those relating to English language conventions are as follows:

1 *Pairs of words should be ordered in accordance with natural usage: for example, 'bread and butter' not 'butter and bread'. The shorter, less stressed word goes first; if it does not, the phrase will not correspond to a stored memory.*

2 *The 'unmarked' term should be used to label the dimension. The term that implies a specific value is called the marked term, and should not be used to label the dimension itself – if it is, it will mislead the reader. For example, we say 'how high is that' without necessarily implying it is high, but if we say 'how low is that' we imply that it is low; 'low' is the marked term.* (In his textbook Kosslyn (1994, p.94) changes terminology, and refers to the marked term as the *loaded* term.)

The advice Kosslyn (1994, p.94) offers in respect of his second point is that *when you describe the two ends of the visual continuum, think about the words; is either loaded? Use the term that does not commit you to a particular end of the continuum.* In other words, terms should not be used which imply a predetermined outcome to the reader.

See also Section 3.14 in relation to integrating graphics and text and Section 3.22 on the use of colour in graphs.

3.19 Reference lines and error bars

Cleveland (1994) suggests the use of a reference line when there is an important value that must be seen across the entire graph, as long as the line does not interfere with the data.

The use of error bars to show variability in the data being graphed is also advocated by Cleveland (1994), Kosslyn (1994) and Gillan et al. (1998). This is done by placing an "I" bar on each plotting symbol in a line graph or on the topmost horizontal of a bar in a bar graph.

Cleveland (1994, p.59) notes that error bars should be unambiguous, and clearly explained in either the text or the caption. He suggests that they can be used to show:

1 *The standard deviation of the sample*

2 *An estimate of the standard deviation or standard error of data*

3 *A confidence interval.*

Gillan et al. (1998, pp.32-33) suggest that error bars typically indicate plus or minus one standard error of the mean. Alternatively, they frequently represent 95 per cent confidence limits.

If the error bars on a line graph overlap so that the reader cannot discriminate the error bars for data at the same level of the independent variable on the x axis, they recommend either of the following:

1 *Display the data in a bar graph (because the bar indicators for data at the same level of the independent variable are not vertically aligned, so the error bars won't overlap)*
2 *Show only the top half of the error bars on the upper line and the bottom half of the error bars on the bottom line.*

Kosslyn (1994) supports the use of half "I" bars in all bar and line graphs, though recommends full "I" bars in scatter plots (examples of their use are provided on pp.124-125; 144-145; and 156-157). It is noted that confidence intervals are not always symmetric about the point estimate, often the case in the analysis of population health data. In this instance half error bars may not always show the full picture. No authors of the literature reviewed tested subjects' *understanding* of error bars.

3.20 Legends and keys

Tufte (1983, p.183) prefers the use of labels on a graph, rather than use of a legend. His principles in this respect are:

1 *Words are spelt out, mysterious and elaborate encoding avoided.*
2 *Words run from left to right, the usual direction for reading occidental languages.*
3 *Little messages help explain data.*
4 *Elaborately encoded shadings, cross hatching, and colours are avoided; instead labels are placed on the graphic itself; no legend is required.*

The preference of labels over a legend is supported in earlier experimental work by Culbertson and Powers (1959) and Milroy and Poulton (1978). In the latter study the authors considered directly labelling the indicators in a graph, or including a legend to explain their meaning, inserted either on the graph field below the functions, or below the field in the position of the figure caption. For both a separate groups and a subsequent within subjects comparison in which subjects were required to determine exact values from line graphs, direct labelling produced the best results in terms of speed and accuracy. Reading the labels directly appeared to involve fewer steps and depend less upon short term memory.

In their literature searches to date, Casali and Gaylin (1988) and Spence and Lewandowsky (1990) all found in favour of the placement of labels directly on the graphical elements in preference to the inclusion of a legend. The disadvantage associated with using a legend was found to be independent of its location – either within the graph or below the axes. The preferred location of labels is in as close proximity as possible to their associated graph element. Relatively recent empirical work by Carpenter and Shah (1998) and Gillan et al. (1998) further supports labelling lines directly and avoiding the use of legends. Culbertson and Powers (1959) determined that pictorial symbols on graphical elements were almost as effective as written labels.

Kosslyn (1994) and Gillan et al. (1998) concede the use of a legend only when:

1 The indicators are too close to be labelled unambiguously, as in the case of a single graph containing multiple lines.
2 The same entities appear in more than two graphs of a multi-panel display (Kosslyn 1994 only).

Gillan et al. (1998, p.34) prescribe the following in respect of legends in graphs:

1 *The legend should show symbols for all indicators clearly and, for each indicator, should show the entire symbol (e.g. both the redundantly coded plotting symbol and the line for each line graph indicator) with the label in close proximity.*
2 *Separate the legend visually from the indicators by placing it in a box.*
3 *Put the legend close to the indicators to reduce scanning distance, but not so close as to interfere with the indicators. The order of the symbols in the legend should match the order of the indicators in the graph.*

Where a legend must be used, Kosslyn (1985) cautions the author against including too much material in it, and so forcing the reader to engage in an arduous memorisation task.

Cleveland (1994) again further contradicts majority consensus by suggesting that one should avoid putting notes and legends inside the graph. He advises placement of a key outside the rectangle, with notes in the caption or in the text.

It is noted that most graphics software programs incorporate a disincentive to directly label indicators since the programs will automatically designate them by use of a legend; direct labelling requires manual intervention.

3.21 Axes, scales and tick marks

1 Relative importance

If precise values are to be detected then labels (scales) on the axes are essential. However, if the main purpose of the graph is to illustrate underlying trends in the data then axes and scales are of relatively low importance. While it is generally agreed that axes and scales on graphs can serve useful purposes and should not be omitted, Tukey (1993, p.3) makes the excellent point that *if we find looking at scales essential in viewing a graphic, we ought to ask whether the graphic is either apt or adequate for its presumed purpose.*

2 The minimalist approach

Tufte (1983), in his bid to remove as much non-data ink as possible from graphs, advocates the exclusion of a frame, vertical axis and tick marks from typical histograms, bar charts and box plots although, for bar type charts, he concedes that *a thin baseline looks good* (Tufte 1983, p.128). For bar graphs, a white background grid (which appears as horizontal lines through the vertical bars), with corresponding quantitative labels indicated on the side of the chart, is preferred in place of the vertical axis and tick marks.

For scatter plots and line graphs Tufte (1983, pp.130-131) prefers a *range-frame*: *one which extends only to the measured limits of the data rather than, as is customary, to some arbitrary point like the next round number...By trimming off that part of the frame exceeding the limits of the observed data, the range frame explicitly shows the maximum and minimum of the variable[s] plotted*. Other variations of the frame are also discussed (Tufte 1983, pp.133-135).

Tufte, however, is not well supported in his quest to, wherever possible, dispense with axes, frames and tick marks on graphs.

3 Functions served by axes, scales and tick marks

The existence of optical illusions is a long known phenomenon, and the potential for them to be incorporated in graphs and maps has also been demonstrated. Poulton (1985) showed that the relationship of sloping lines to the vertical and horizontal axes of graphs can produce reading errors that increase with distance from the axes (the Poggendorf illusion). To mitigate this bias he suggested that graphs should show all four axes (not just two), and that all axes should be calibrated. Schmid (1983), Gillan (1994), Cleveland (1994) and Gillan et al. (1998) also advocate the use of Y axes on each side of the chart. It is noted, however, that in their literature search to date Spence and Lewandowsky (1990) reported that four axes are rarely used in graphic displays in scientific journals.

Gillan et. al (1998, p.37) provide further details of the types of errors (under and over estimations) that can result from different types of illusions, and Kosslyn (1994, p.276 and p.282) provides multiple references for the discussion of the role of such illusions in graphs.

Kosslyn (1989) proposed that although ink in the axes does not represent data, it may help the reader to organise the visual space of the graph into two major constituents:

1 The quantitative and verbal labels located outside the axes that describe the categories of data.

2 The indicators located inside the axes that specify the data.

Bennett and Flach (1992) recommend maintaining and emphasising scale to aid focused attention tasks which concentrate on just one of the variables in the chart.

Hollands and Spence (1992) found that judgments of proportion using line and bar graphs were improved by the presence of a scale. Without a scale these types of graphs were found to be much less effective than pie charts and divided bar charts (bars of equal height with proportions summing to 100 per cent) in making judgments of proportion. They contend that the presence of a scale reduces the advantage of pies and divided bars in making proportion judgments since the scale provides reference points against which subjects could compare segments in bars and line graphs.

Gillan and Richman (1994) cite prior human factor literature on reading dials which suggests that tick marks might be beneficial. Findings indicate that dials should include a scale marker for every unit to be read, and that people read a moving pointer, fixed-scale dial faster when the pointer is exactly on a scale marker than when the pointer is between markers.

In empirical research, based on their own model of graphical perception, to test Tufte's (1983) data ink principle, Gillan and Richman (1994) found that the ink in the form of axis *lines* improves performance, and so recommended that practitioners make use of axis lines. A major exception to this rule was the use of the y axis line with bar graphs. Because the bars may perform the function of the axis line, eliminating it appears to improve performance.

Gillan and Richman (1994) also determined that *tick marks* on the y axis of bar and line graphs frequently decreased performance, so they recommend that tick marks are *not* included unless other factors warrant them (for example, few numerical labels). This, they postulate, may indicate that the effects of tick marks on graphs are different from those of marks on dials. They suggest that, if users need to know the precise values of indicator(s), then these values should be placed directly above the indicator(s).

Gillan et al. (1998, pp.35-36) expand on these recommendations in respect of tick marks and provide instructive graphical examples. Their general conclusion is that *tick marks will be of no value if they provide information also provided by the quantitative labels of the scale values. Placing tick marks only next to each label is redundant and can increase the time to read a graph. Accordingly, either use tick marks between infrequent scale values on the y or x axis or use frequent scale values and no tick marks.* Kosslyn (1994) and Cleveland (1994) do not share this aversion to tick marks in conjunction with labels. While Cleveland (1994) advocates the use of tick marks, he cautions not to overdo the number of them, and suggests that they should point outward. Kosslyn (1994) believes they should point inward. The lack of a majority view on this aspect of graph construction appears to suggest that the inclusion of tick marks with labels, the number of tick marks and their orientation are all matters for personal judgement depending on the intended purpose of the graph.

4 Range of, and breaks in, the scale

Kosslyn (1994, pp.78-83) provides multiple examples of, and recommendations for, choosing the range for a graph scale. All are based on the general principle that the range should be chosen to illustrate the relevant point(s) the author wishes to make, with the visual impression produced by the display conveying actual differences and patterns in the data. Consistent with this approach is his recommendation to display only the *relevant* range of the scale along the y axis: starting the visible scale at zero is not essential unless the zero value is inherently important.

Cleveland (1994), shares this view and suggests choosing the scales on a graph so that the data rectangle fills up as much of the scale-line rectangle as possible. He prefers to avoid scale breaks by *not* insisting that the scale should start at zero. If a break is unavoidable he stresses that scales should not change *within* the graph; the same scale should be used before and after the break in the axis.

Schmid (1983), on the other hand supports the practice of placing a *break* in the y axis scale if it is deemed necessary to portray the data, but believes that it is imperative to retain the point of origin (zero). Kosslyn (1994) concedes that a zero value must be retained for divided bar charts because the main point lof this type of chart is to convey information about the relative proportions of different parts of the whole. In population health divided bar charts are frequently used to show the prevalence of a condition in the population: excising part of the scale on the y axis will alter the visual impression so that the sizes of the segments no longer reflect the relative proportions of the components.

5 The use of multiple scales

The use of multiple scales on a chart (a separate one on each of the left and right Y axes) are sometimes used to graph different dependent variables in the same display. Schmid (1983, p.19) considers this practice to be *dangerous* but suggests it may be useful for comparing:

1 *Functions that vary in magnitude*

2 *Two or more variables measured in different units*

3 *Two or more series without computation.*

Kosslyn (1994) also recommends against using multiple scales since it forces the reader to keep track of what goes with what, taxing our limited processing capacity. The only exception to this recommendation conceded is when the dependent variables are intimately related, and their interrelations are critical to the message being conveyed, because plotting the data in the same display allows them to be perceived as a single pattern. He suggests that line graphs are usually the most appropriate for this purpose, and recommends use of the same colour or pattern to plot the line and the corresponding y axis.

3.22 The use of colour in graphs

The use of colour in graphics has been found to offer some, though not overwhelming, advantages. It may also cause problems if not used judiciously.

Schutz (1961b) found that colour coding multiple line graphs displaying trend data improved performance slightly, though concluded that it should only be used if the cost or time to process visual materials are not important factors. Bennett and Flach (1992) determined that the colour coding of graphical elements aided focused attention tasks which concentrate on just one of the variables in the chart.

In their literature search Casali and Gaylin (1988) found that colour coding can reduce task completion time and is often preferred (subjectively) over black and white symbolic codes. However, in their own experiments they found no differences in subjects' error scores between colour and monochrome coding for any of the tasks associated with any of the various types of graphs used.

In his textbook, Cleveland (1994) supports the use of colour as a tool for data encoding.

Kosslyn (1985, 1989 and 1994) makes multiple recommendations in respect of the use of colour in graphs. The following is a summary of those discussed in detail in Chapter 7 of his 1994 textbook:

1 Use colours that are well separated in the colour spectrum.

2 Adjacent colours should have different brightnesses.

3 Make the most important content element the most salient.

4 Use warm colours to define a foreground.

5 Avoid using red and blue in adjacent regions since they will appear to shimmer if juxtaposed.

6 As noted above in Section 3.8, guideline (iv), respect conventions of colour: for example, in western cultures red symbolises stop or danger; blue: coolness, cleanliness, or safety; green: life. Colours may also have political connotations.

7 Use colour to group elements, for example, when two or more elements are to be compared in different places.

8 Avoid using hue to represent quantitative information, for example, shifting from red to violet does not suggest more of something, in the same way that shifting from a small dot to a large dot does. In a similar vein, Cleveland and McGill (1984) advocate (and illustrate) the use of *framed rectangles* rather than colour saturation or colour hue for showing density (for example, of population, or murder rates). The perceptual task of judging shading is at the bottom of a *perceptual hierarchy* defined by the authors and, they contend, one can move further up the hierarchy by using framed rectangles.

9 If practical considerations force the use of hue to convey quantitative information, then use deeper saturations (more colour) and greater intensities (more light) for hues that indicate greater amounts. That is, intensity or saturation should covary with hue so that lighter colours correspond with higher values.

Possibly related to point (vi) above, the use of colour has been shown to inhibit the correct interpretation of data if the colours are not judiciously chosen. Cleveland and McGill (1983) and Cleveland, Harris and McGill (1983) showed that the use of saturated colours can cause optical illusions in the apparent sizes of objects. In *map* reading experiments the use of high saturation red and green for colouring two regions caused the bright red areas to be judged larger, even though the regions were the same size. No such consistent distortion occurred when the low saturation brighter or pastel colours were used.

Tufte (1983) cautioned that, if colours are used, they should be chosen so that the colour deficient and colour blind can make sense of the graphic. He noted that blue can be distinguished from other colours by most colour deficient people.

Finally on this point, and as specified above in Section 3.8, graphs should be designed with forethought to future copying in order that visual clarity is maintained. Therefore, colours should not be used when a document in which the graphs are contained will be copied in black and white.

3.23 Three dimensional graphs

The literature is ambivalent about the use of three dimensional (3D) graphs. The safest conclusion to draw from it is to avoid their use because perceptual biases may mean that comparisons of height or length at different depths are variable. If they are used, a good 3D chart should be viewed from the perspective of top looking down, so that the face of the bar or column represents the actual reading (Schmid 1983, p.166). Kosslyn (1994, pp.178-181) provides additional recommendations.

The representation of uni-dimensional quantities by higher dimensions, such as boxes, is anathema to Tufte (1983) since it violate ones of his principles to enhance graphical integrity (see Section 3.8 guideline (vi)). Wainer (1984) also recommends against the use of 3D on the basis that human perception of areas is not consistent. Certainly, some studies have found that adding apparent depth to a graph offers, at best, no advantages over other two dimensional formats and, at worst, may inhibit the graph reading task

Casali and Gaylin (1988) demonstrated that, for four types of tasks that subjects were required to complete, 3D bar graphs were less effective than point plots, line graphs and conventional (two dimensional) bar graphs for the point reading task, and their use may result in confusion regarding trend interpretation and point comparison.

Carswell (1991) and Carswell, Frankenberger and Bernhard (1991) evaluated performance using common graphical formats (bar graphs, line graphs and pie charts) constructed with and without the 3D look. The addition of depth was associated with less accurate performance for subjects attempting to estimate the relative magnitude of displayed values, classify and describe trends, and recall quantitative information about both specific values and trends. Line graphs more than bar graphs or pie charts were susceptible to the impact of decorative depth on performance. However, the use of 3D designs tended to influence the attitudes formed by subjects toward the information presented in graphs: in general they felt more positive about the subject in the 3D depictions.

Schmid (1983, p.154) recognises that the *eye appeal and novelty* of 3D charts are positive characteristics, but stresses that these are not sufficient reasons to justify use of the technique. He claims that the overall effectiveness of graphical communication depends on its clarity, simplicity, accuracy, forcefulness and interpretability, and recommends that if precision of measurement is required 3D charts should be avoided.

An implication of Gillan's (1994) MA-P model (elaborated upon in Section 3.8, guideline (xiii)) is that graphs should not include features that mask the indicator, including three dimensionality. Such features may help attract users' interest in the graph but can make it difficult for the user to discriminate the indicator from the background.

Others have obtained more positive results, though not unequivocally in favour of adding apparent depth.

Barfield and Robless (1989) investigated the relationship between 2D and 3D graphs displayed on paper and computer screen, and the problem solving performance of experienced and novice managers. They examined the effects of these variables on solution times, confidence in answers and effectiveness of solutions for a production management case. Solution times were found to be slower using the 3D presentations of graphs, but the use of 3D graphs on computer led to the most effective answers. Novice subjects produced more accurate answers using 2D paper presentations of graphs while experienced managers produced more accurate answers when provided with 3D graphs on computer. Both experienced and novice managers were more confident of their answers when provided with 2D graphs as decision aids than with any other mode of presentation. Note, however, that in these experiments it is not clear how the confounding effects of the medium of display (computer or paper) on subjects' performance with the type of display (2D or 3D) were controlled.

Wickens, Merwin and Lin (1994) found that perspective (3D) representations supported superior performance than planar (2D) representations, but only for more integrative questions considering the overall content of the graph.

Spence and Krizel (1994) determined that younger children can be misled by irrelevant dimensions of objects used to portray magnitudes and proportions, but older children could make their judgments like adults, ignoring or accommodating the additional dimension.

Spence, cited in Spence and Lewandowsky (1990, p.25) determined that the addition of extra dimensions is *not harmful* provided that the base size of the graph remains constant. He concluded that *the presence of irrelevant dimensions makes for a more attractive display that is processed more quickly, with no concomitant loss of judgemental accuracy, provided the extra dimensions are purely decorative and carry no information.*

Gillan et al. (1998, pp.36-37) make the following recommendations:

1 *A depth axis (i.e. a Z dimension) might be used only if it portrays two levels and if readers will not need to use the graph to determine the amount of difference in the Z dimension.*

2 *Rather than using a 3D graph to show the relation between two independent variables, consider using multiple lines in a line graph, one line for each level of the second predictor variable, or multiple bars with pattern coding. Rather than using a 3D graph to show the relations among three independent or predictor variables, consider using multiple graphs, one for each level of the third predictor variable.* Examples of these types of graphs are provided.

3.24 Line and symbol weight and type

Not a lot has been written *specifically* on line weight and type. In general, both Tufte (1983) and Kosslyn (1985 and 1989) agree that the weights assigned to items in graphs should be proportional to their importance in the display, with more important items emphasised by heavier line weights. Tufte (1983) specifies a preference for *thin* lines as a starting point, which can then be varied in weight according to their function in the chart.

With specific reference to line type (solid, dotted, dashed etc.) and plotting symbol (or marker) type (various geometric shapes, filled or unfilled), Schutz (1961b) concluded that the styles selected should be *maximally discriminable*, resulting in the *least amount of confusion* (Schutz 1961b, p.111). From a set of 25 types, all drawn manually, four non-solid lines with standard geometric plotting symbols were selected as optimally non-confusing, though he was quick to point out that they did not represent the only such set of lines. Since the introduction of computer generated graphics an almost infinite set of line type and symbol combinations is available, but the recommendation to maintain maximum discriminability in resect of all 'marks' on a chart still holds (see Section 3.8 guideline (ix)).

In order that the reader will be attracted to the main point of the graph, Gillan et al. (1998, p.33) suggest the following in relation to different types of graphs:

1 *In bar graphs, use dark, heavy lines as the pattern code of a bar if the reader should pay attention to those data.*

2 *In line graphs, use dark, filled plotting symbols or dark, thick lines if the reader should pay attention to those data.*

3 *In scatter plots that show the best fitting line that summarises the data, make the best fitting line thick and dark relative to the data points so the reader will pay attention to the summary.*

4 *In line or bar graphs that use error bars to show variability, do not make the error bars thick and dark relative to the indicator...*

3.25 Background features and grid lines

A common background in graphs is the extension of the tick marks from one or both axes, forming a grid that appears behind the indicators. The main function of grid lines is to facilitate the extraction of specific values, which can be read off the y axis more easily if one can trace along the grid lines. Kosslyn (1994) illustrates the usefulness of gridlines to help the eye focus on relevant detail (for example, the vertical distance between two lines rising exponentially) when our visual system distorts images and optical illusions are created. See also Section 3.21 point (iii).

According to Tufte (1983), ink that displays a picture or illustration in the background of a graph, but does not represent data, would be non-data ink and should not be included (though he did suggest use of a white horizontal grid behind bar charts – see Section 3.21 point (ii)).

As noted above in Section 3.8 guideline (xi), Kosslyn (1985) specified that graphs should be constructed so that one notices the more important things first, and marks should be chosen to be noticed in accordance with their importance in the display. The physical dimensions of marks should be used to emphasise the message; they should not distract from it. On this basis,

inner grid lines should be lighter than content lines, and background patterns should never be as noticeable as the content components of the graph itself.

Schmid (1983) advocates the use of gridlines, but stresses they should be kept to a minimum and be drawn lighter than the plotted functions.

A picture in the background might make a chart more attractive, or help to reinforce the main point of the display. However, the picture might also make it more difficult to perceive the data ink in the graph if it:

1 Produces a simultaneous masking, thereby reducing the visibility of the elements in the graph which indicate values: for example, the bars in a bar graph and the points and connecting lines in a line graph (Olzak and Thomas 1986, cited in Gillan and Richman 1994, Gillan 1994, Kosslyn 1994).
2 Functions as a distractor. The greater the similarity of the visual features in the distractor and indicators, the greater the interference in search. The more ink in the background of a graph, the greater the likelihood that some of the features would resemble those in the indicator (Neisser 1963 and 1964, cited in Gillan and Richman 1994).

In their experimental work Culbertson and Powers (1959) found that numerical labels on elements better facilitated focused attention tasks than the inclusion of a grid.

3.26 Conclusion – is there hope for the graphically challenged?

What combination of features produces a good graph? The answer from the literature is fairly clear, though not immediately helpful, and that is *it depends*.

There is, of course, hope for the graphically challenged, but the guidelines specified and discussed in the preceding pages are just that: guidelines to aid in the construction of a good graph. Just how good a graph will be ultimately rests on judgments made by its author. Many simple rules have been proposed, appealing because of their simplicity, but not universally applicable. The more complex principles and procedures are generally not practical when there is a limit to the amount of time which can be spent on any one task, including the graphical presentation of data. In any case, adhering to a principle involves making judgments which may be difficult, and/or made inconsistently by different people – or even the same person.

In coming up with a set of best practice principles which are both widely, if not universally, applicable, and suitable for practical application, the necessity of authors to make their own value judgments about the content of specific graphs is unavoidable. The trick is to make the right judgments. If principles and recommendations are at least considered, then hopefully the final judgment decisions will not be too far wrong.

Individual judgments will be particularly critical when deciding the *aim* of the graph, the *selection of dependent and independent variables*, and the *organisation* of these variables in the graph. Each of the latter two decisions will be related to the aim of the graph. Once these decisions have been made, the specific best practice recommendations will be relevant. For practical convenience, they have been summarised immediately below.

Tufte (1983, p.177) clearly recognised the need for judgement and, even though his recommendations are not evidence based, his eloquency has earned him the last say:

> *Design is choice. The theory of the visual display of quantitative information consists of principles that generate design options and that guide choice among options. The principles should not be applied rigidly or in a peevish spirit; they are not logically or mathematically certain; and it is better to violate any principle than to place graceless or inelegant marks on paper.*

3.27 Summary of best practice principles

Graph design feature	Level of evidence
**General principles**	
1. Ensure that tasks supported by the graph constructed are consistent with the tasks which readers will be required to undertake.	L2
2. A single graph should be able to support 'global' interpretation tasks by being configured to produce *emergent features*, as well as 'local' interpretation tasks by emphasising its elemental properties. Emergent features are produced by the interactions among individual elements of a graph, for example, lines, contours and shapes which occur when two variables are mapped – one in the x axis and one in the y axis – to produce the emergent features of area.	L2
3. Use *common graphs* with which all readers are likely to be familiar: for example, line and bar graphs, pie charts and scatter plots.	L2
4. The conventions of a reader's culture should be obeyed: for example, the colour red should not be used to signify 'safe' areas, and green should not be used to signify 'danger'; numerical scales should increase going from left to right or bottom to top.	L2
5. Similarly, the appearance of words, lines and areas in a graph should be compatible with their meanings, for example, the word 'red' should not be written in blue ink, larger areas in the display should represent larger quantities, and faster rising lines should represent sharper increases.	L2
6. If there is a possibility that readers may lack the necessary background knowledge to interpret a chart, then a sufficient amount of *domain-specific* information should be included in the text to ensure adequate comprehension of the accompanying chart(s). Additionally, charts should be labelled to provide domain-specific information, including a full explanation of all abbreviations and acronyms: preferably, these should be avoided altogether.	L2
7. *Context-specific* support should also be provided by spelling out in the accompanying text the nature of the relationships illustrated in the graph so that the two reinforce the relevant message	L2
8. Graphs, relevant text and, where appropriate, statistical analysis in a document should be integrated in close physical proximity. The terminology used in the display should be the same as that in the text or presentation.	L2
9. Do not over adorn charts or include an excessive amount of information in them. This recommendation will necessarily involve judgments necessary to limit the amount of clutter in a chart, while at the same time ensuring that the intended message is clear and unambiguous, as well as being aesthetically appealing enough to be read. Probably the best and safest approach to take is to start with a relatively minimal presentation and include more only if a strong reason for doing it can be explicitly articulated.	L2
10. Visual clarity is essential. To this end, all 'marks' on a graph must have a minimal magnitude to be detected, and they must be able to be perceived without distortion. Marks must also be relatively discriminable: that is, two or more marks must differ by a minimal proportion to be discriminated. Design graphs so that the visual clarity is maintained if, in the future, they are copied.	L2
11. Construct graphs so the more important things are noticed first. That is, the main point of the graph should be its most visually salient feature, and one should avoid making the reader search for the main point in the details of the graph. Marks should be chosen to be noticed in accordance with their importance in the display, and the physical dimensions of marks should be used to emphasise the message; they should not distract from it. For example, inner grid lines should be lighter than content lines, and background patterns or colours should never be as noticeable as the content components of the graph itself.	L2
12. Include in a graph only (but at least) the necessary amount of data to make the relevant point(s). Excess or irrelevant data can hinder trend reading tasks.	L2
13. In general, lengths should be used, as opposed to areas, volumes or angles, to represent magnitudes wherever possible. Most preferable is the plotting of each measure as a distance from a common baseline so that *aligned lengths* are being compared. Note, however, that the use of pie charts for part-to-whole comparisons, discussed below, is an exception to this rule	L2

Graph design feature	Level of evidence
**General principles**	
14. Data which are to be compared should be in close spatial proximity.	L2
15. A related (or even the same) principle is that when there is more than one independent variable to be considered, the most important one should be on (and label) the x axis, and the others should be treated as parameters representing separate bars or lines. For example, if comparisons between categories *within a given year* are to be described, then an appropriate format would be sets of bars grouped such that each cluster consisted of x number of different categories within a given year. To describe comparisons of a *single category across years*, an appropriate format would be the use of dots connected by lines such that each line consists of a measurement of a single category across years.	L2
16. If there is no clear distinction between the importance of the variables, put an interval scaled independent variable (if there is one) on the x axis. The progressive variation in heights from left to right will then be compatible with variation in the scale itself. If there is more than one independent variable with an interval scale, put the one with the greatest number of levels on the x axis.	L2
17. When possible, use graphs that show the results of arithmetic calculations (for example, a stacked bar graph for addition). Otherwise, design graphs which *minimise* the number of arithmetic operations which readers must undertake to complete the required task. For example, where the focus is on the difference between two functions, a single line showing the difference should be drawn, rather than the two original functions. If the slope, or rate of change, of a function is most important, the rate of change should be plotted rather than the original data.	L2
18. Design graphs (especially graphs in a series) to have a consistent layout such that the location of indicators is predictable.	L2
19. Limit the number of lines on a chart and number of bars over each data point. Evidence suggests that where there are more than four groups of lines on a chart, or four bars over each point, it is best to divide the data into subsets and graph each subset in separate panels of a display. To aid search across multiple graphs with many levels of an independent variable:<br>• Place all graphs in one figure to facilitate search across graphs<br>• Use spatial proximity for graphs so that the reader might search in sequence<br>• Maintain visual consistency across graphs (for example, use the same size axes, the same types of indicators, and the same coding of indicators for the same variables)<br>• Maintain semantic consistency across graphs (for example, use the same scale on the y axis)<br>• Eliminate redundant labels (for example, in a series of horizontally aligned graphs, the labels for the Y axes should be placed only on the leftmost graph, and the label naming the x axis should be centred under the series)<br>• Avoid using a legend to label indicators (a legend requires multiple scans between the indicators and the legend, thereby disrupting visual search); label indicators (lines, bars, pie segments etc.) directly<br>• Assign data that answer different questions to different panels<br>• Assign lines that form a meaningful pattern to the same panel<br>• Put the most important panel first.	L2

Graph design feature	Level of evidence
**Choice of graph type**	
20. Pie charts should be used for *part-to-whole* judgments involving comparison of one proportion of an item to its whole, never for *part-to-part* judgments involving a decision of what proportion a smaller value is of a larger, where the smaller value does not form part of the larger one (as would be the case when *changes* in the magnitude of a variable are to be detected).	L2
21. Line graphs are most appropriate for showing data trends and interactions, and identifying global patterns in data, though bar graphs also support these tasks. Note that *trends* can be described in terms of *rising, falling, increasing, or decreasing.* There is some evidence that line graphs are more biasing than bar graphs: that is they emphasise x-y relations. Consequently, if two independent variables are equally important, bar graphs should be used. If a particular trend is the most important information, then line graphs should be used.	L2
22. The literature is divided over the preference of lines or bars to facilitate the extraction of exact values, though it probably comes down in favour of bars.	L2
23. When *multiple trends* are to be compared, showing several trend lines on a *single* graph is superior to presenting single trend lines on several graphs. This type of 'layer' graph is useful in illustrating the relative change in one component over changes in another variable. Because the spaces between the lines can be filled, they can be seen as shapes, and the change in a single proportion can be easily seen. However, note that layer graphs should only be used to display continuous variables: that is, values on an interval scale. If the x axis is an ordinal scale (one that specifies ranks) or nominal scale (one that names different entities) the eye will incorrectly interpret the quantitative differences in the slopes of the layers as having meaning. In these cases the use of a divided bar graph is recommended.	L2
24. Single line graphs are also most effective for indicating data *limits* (maxima and minima – for example, the year in which product A's sales peaked; and *conjunctions* (the intersection of two indicators – for example, the year in which product A first sold more than product B).	L2
25. While line graphs are to be most preferred for showing trends, bar graphs run a close second, as long as they are vertical, not horizontal. Bar graphs are also preferred if precise values need to be detected, and they are a good 'compromise' if both local (or *discrete*) and global interpretations of the data need to be made. *Discrete comparisons* can be described in terms of *higher, lower, greater than, or less than.*	L2
26. Bar graphs are also useful for displaying data *limits* (maxima and minima), and *accumulation* (the summation indicators – for example, to determine which of several products sold the most overall).	L2
27. The balance of evidence supports the use of vertical, rather than horizontal bar graphs for discrete comparisons, though there is 'room' for subjective consideration of the data to determine a preference. When in doubt, use a vertical bar graph format, since increased height may be considered a better indicator of increased amount.	L2
28. 'Side-by-side' horizontal bar graphs which show pairs of values (values for two independent variables) that share a central y axis are recommended to show contrasting trends between levels of an independent variable, and comparisons between individual pairs of values.	L2

Graph design feature	Level of evidence
**Graph elements**	
29. Captions should be visually prominent, preferably centred at the top of the chart. They should describe everything that is graphed in terms of *what, where, who and when* and any other descriptors considered to be necessary.	L3
30. Placement of numerical values directly onto a graph will aid local interpretation tasks.	L2
31. Tick marks should be included on the axes, and labelled at regular intervals, using round numbers. Where the range of values on an axis dictates that labels should include decimal points to be meaningful, the number of decimal places should be kept to a minimum.	L2
32. Axis labels should be centred and parallel to their axis: that is, the y axis should *not* be labelled horizontally on the top left hand side of the data rectangle.	L2
33. For graphs that are wide, place y axes and the associated numerical labels on both sides of the data.	L2
34. Inclusion of a *reference line* is recommended when there is an important value that must be seen across the entire graph, as long as the line does not interfere with the data.	L2
35. The use of error bars to show variability in the data being graphed is also recommended. If the error bars on a line graph overlap so that they cannot be discriminated for data at the same level of the independent variable on the x axis, either or both of the following is suggested:<br>• Display the data in a bar graph (because the bar indicators for data at the same level of the independent variable are not vertically aligned, so the error bars will not overlap)<br>• Where confidence intervals are symmetric about the point estimate, show only the top half of the error bars on the upper line and the bottom half of the error bars on the bottom line.	L1
36. Wherever possible, directly label indicators in a graph rather than including a legend to explain their meaning. Labels should be in as close proximity as possible to their associated graph element.	L2
37. Where the use of a legend is unavoidable because the indicators are too close to be labelled unambiguously, it should be placed close to the indicators to reduce scanning distance, but not so close as to interfere with the indicators. The order of the symbols in the legend should match the order of the indicators in the graph.	L2
38. While a contentious issue, majority evidence suggests that starting the y axis visible scale at zero is not essential unless the zero value is inherently important. However, note that a zero value must be retained for divided bar charts because the main point of this type of chart is to convey information about the relative proportions of the different portions of the whole. Excising part of the scale on the y axis will alter the visual impression so that the sizes of the segments no longer reflect the relative proportions of the components.	L2
39. In general, the range of the scale should be chosen to illustrate the relevant point(s) the author wishes to make, with the visual impression produced by the display conveying actual differences and patterns in the data. Do not make the scale maximum any larger than necessary to accommodate all data points; otherwise, a portion of the upper plot area will be left empty.	L2
40. It is best to avoid the use of multiple scales on a chart (a separate one on each of the left and right Y axes). A possible exception to this rule is when the dependent variables are intimately related, and their interrelations are critical to the message being conveyed. In this case, plotting the data in the same display allows them to be perceived as a single pattern. Line graphs are usually the most appropriate for this purpose, and use of the same colour or pattern to plot the line and the corresponding y axis should be considered.	L2
41. Avoid the use of three dimensional graphs because perceptual biases may mean that comparisons of height or length at different depths are variable. If they are used, a good 3D chart should be viewed from the perspective of top looking down, so that the face of the bar or column represents the actual reading.	L2

Bibliography

Attali Y and Goldschmidt C 1996, The Effects of Component Variables on Performance in Graph Comprehension Tests, *Journal of Educational Measurement* 33(1): 93-105.

Barfield W and Robless R 1989, The Effects of Two- and Three-dimensional Graphics on the Problem-Solving Performance of Experienced and Novice Decision Makers, *Behaviour and Information Technology* 8(5): 369-385.

Benbasat I and Dexter AS 1985, An Experimental Evaluation of Graphical and Color-Enhanced Information Presentation, *Management Science* 31(11): 1348-1364.

Bennett KB and Flach JM 1992, Graphical Displays: Implications for Divided Attention, Focused Attention and Problem Solving, *Human Factors* 34(5): 513-533.

Bennett KB, Toms ML and Woods DD 1993, Emergent Features and Graphical Elements: Designing More Effective Configural Displays, *Human Factors* 35(1): 71-97.

Carpenter PA and Shah P 1998, A Model of the Perceptual and Conceptual Processes in Graph Comprehension, *Journal of Experimental Psychology* 4(2): 75-100.

Carswell CM 1991, Boutique Data Graphics: Perspectives on Using Depth to Embellish Data Displays in *Proceedings of the Human Factors Society 35th Annual Meeting* Human Factors and Ergonomics Society, Santa Monica CA: 1532-1536.

Carswell CM 1992a, Choosing Specifiers: an Evaluation of the Basic Tasks Model of Graphical Perception, *Human Factors* October 34(5): 535-554.

Carswell CM 1992b, Reading Graphs: Interactions of Processing Requirements and Stimulus Structure, B Burns (ed), *Percepts, Concepts and Categories,* Elsevier Science Publications, Amsterdam: 605-645.

Carswell CM and Ramzy C 1997,Graphing Small Data Sets: Should We Bother?, *Behaviour and Information Technology* 16: 61-71.

Carswell CM and Wickens CD 1990, The Perceptual Interaction of Graphical Attributes: Configurality, Stimulus Homogeneity and Object Integration, *Perceptions and Psychophysics* 47: 157-168.

Carswell CM, Frankenberger S and Bernhard D 1991, Graphing in Depth: Perspectives on the Use of Three-Dimensional Graphs to Represent Lower Dimensional Data, *Behaviour and Information Technology* 10(6): 459-474.

Casali JG and Gaylin KB 1988, Selected Graph Design Variables in Four Interpretation Tasks: A Microcomputer-Based Pilot Study, *Behaviour and Information Technology* 7(1): 31-49.

Chambers MJ, Cleveland WS, Kleiner B and Tukey PA 1983, *Graphical Methods for Data Analysis,* Bell Laboratories Duxbury Press, Boston MA.

Cleveland WS 1993, *Visualizing Data*, AT and T Bell Laboratories, Murray Hill NJ.

Cleveland WS 1994, *The Elements of Graphing Data* AT and T Bell Laboratories, Murray Hill NJ.

Cleveland WS and Fisher NI 1998, *Good Graphs for Better Business* http://www.cmis.csiro.au/statline/1998/aug98.htm

Cleveland WS and McGill R 1983,A Color-Caused Optical Illusion on a Statistical Graph, *The American Statistician* 37(2): 101-105.

Cleveland WS and McGill R 1984, Graphical Perception: Theory, Experimentation and Application to the Development of Graphical Methods, *Journal of the American Statistical Association* 79(387): 531-554.

Cleveland WS and McGill R 1985, Graphical Perception and Graphical Methods for Analyzing Scientific Data, *Science* 229: 828-833.

Cleveland WS and McGill R 1987, Graphical Perception: the Visual Decoding of Quantitative Information on Graphical Displays of Data, *Journal of the Royal Statistical Society* 150(3): 192-229.

Cleveland WS and McGill R (eds) 1988, *Dynamic Graphs for Statistics,* Wadsworth and Brooks, Belmont CA.

Cleveland WS, Harris CS and McGill R 1983, Experiments on Quantitative Judgments of Graphs and Maps, *Bell System Technical Journal* 62 (6): 1659-1674.

Cleveland WS, McGill ME and McGill R 1988, The Shape Parameter of a Two-Variable Graph, *Journal of the American Statistical Association* 83(402): 289-300.

Culbertson HM and Powers DR 1959, A study of Graph Comprehension Difficulties, *Audio-Visual Communication Review* 7: 97-100.

Demana F and Waits BK 1988, Pitfalls in Graphical Computation, or Why a Single Graph Isn't Enough, *College Mathematics Journal* 19(2): 177-183.

Ferry B, Hedberg J and Harper B 1999, *Developing Computer-Based Cognitive Tools That Assist Learners to Interpret Graphs and Tables,* http://www.swin.edu.au/aare/99pap/fer99092.htm

Gillan DJ 1994, A Componential Model of Human Interaction with Graphs: I Linear Regression Modelling, *Human Factors* 36(3): 419-440

Gillan DJ and Callahan AB 2000, A Componential Model of Human Interaction with Graphs: VI, Cognitive Engineering of Pie Graphs, *Human Factors* 42(4): 566-591

Gillan DJ and Neary M 1992, A Componential Model of Human Interaction with Graphs: II. The Effect of Distance Between Graphical Elements,*Proceedings of the Human Factors Society 35th Annual Meeting,* Human Factors and Ergonomics Society, Santa Monica CA: 365-368.

Gillan DJ and Richman EH 1994, Minimalism and the Syntax of Graphs, *Human Factors* December 36(4): 619-644.

Gillan DJ, Wickens CD, Hollands JC and Carswell CM 1998, Guidelines for Presenting Quantitative Data in HFES Publications, *Human Factors* 40(1): 28-41.

Greaney J and MacRae AW 1997, Visual Search and the Detection of Abnormal Readings in Graphical Display, *Acta Psychologica* 95(2): 165-179.

Guthrie JT, Weber S and Kimmerly N 1993, Searching Documents: Cognitive Processes and Deficits in Understanding Graphs, Tables and Illustrations, *Contemporary Educational Psychology,* 18: 186-221.

Henry GT 1993, Using graphical displays for evaluation data, *Evaluation Review* 17(1): 60-78.

Hollands JG 1992, Alignment, Scaling, and Size Effects in Discrimination of Graphical Elements, *Proceedings of the Human Factors Society 35th Annual Meeting,* Human Factors and Ergonomics Society, Santa Monica CA: 1393-1397.

Hollands JG and Spence I 1992, Judgments of Change and Proportion in Graphical Perception, *Human Factors* 34(3): 313-334.

Kosslyn SM 1985, Graphics and Human Information Processing, *Journal of the American Statistical Association*, 80 (391): 499-512.

Kosslyn SM 1989, Understanding Charts and Graphs, *Applied Cognitive Psychology*, 3: 185-226.

Kosslyn SM 1994, *Elements of Graph Design,* WH Freeman and Company, New York NY.

Lee JM and MacLachian J 1986, The Effects of 3D Imagery on Managerial Data Interpretation, *MIS Quarterly* September: 257-269.

Lovie AD and Lovie P 1991, Graphical methods for exploring data, Lovie P and Lovie AD (eds), *New Developments in Statistics for Psychology and the Social Sciences Volume 2,* Taylor and Francis/Routledge, Florence KY: 19-48.

Lowe RK 1993, Constructing a Mental Representation from an Abstract Technical Diagram, *Learning and Instruction* 3: 157-179.

Mahon BH 1977, Statistics and Decisions: The Importance of Communication and the Power of Graphical Presentation, *Journal of the Royal Statistical Society* 140(3): 298-323.

Mayer RE 1993, Comprehension of Graphics in Texts: an Overview, *Learning and Instruction* 3: 239-245.

Mayer RE, Bove W, Mars R and Tapangco L 1996, When Less Is More: Meaningful Learning From Visual and Verbal Summaries of Science Textbook Lessons, *Journal of Educational Psycholog,* 88(1): 64-73.

Meyer J 1997, A New Look at an Old Study on Information Display: Washburne (1927) Reconsidered, *Human Factors* September 39(3): 333-340.

Meyer J, Shamo MK and Gopher D 1999, Information Structure and the Relative Efficiency of Tables and Graphs, *Human Factors* December 41(4): 570-587.

Meyer J, Shinar D and Leiser D 1997, Multiple Factors that Determine Performance with Tables and Graphs, *Human Factors* June 39(2): 268–286.

Milroy R and Poulton EC 1978, Labelling Graphs for Improved Reading Speed, *Ergonomics* 21(1): 55-61.

National Cancer Institute (date unknown) *Usability.gov* http://usability.gov/guides/index.html

Poulton EC 1985, Geometric Illusions in Reading Graphs, *Perceptions and Psychophysics* 37: 543-548.

Remus W 1987, A Study of Graphical and Tabular Displays and Their Interaction with Environmental Complexity, *Management Science* 33(9): 1200-1204.

Roth WM and McGinn MK 1997, Graphing: Cognitive Ability or Practice?, *Science Education* January 81(1): 91-106.

Schmid CF 1983, *Statistical Graphs Design Principles and Practices,* John Wiley and Sons, New York NY.

Schnotz W 1993, Introduction, *Learning and Instruction* 3: 151-155.

Schutz HG 1961a, An Evaluation of Methods for Presentation of Graphic Multiple Trends, *Human Factors* 3: 108-119.

Schutz HG 1961b, An Evaluation of Formats for Graphic Trend Displays – Experiment III, *Human Factors* 3: 99-107.

Shah P, Mayer RE and Hegarty M 1999, Graphs as Aids to Knowledge Construction: Signaling Techniques for Guiding the Process of Graph Comprehension, *Journal of Educational Psychology* December 91(4): 690-702.

Simkin D and Hastie R 1987, An Information-Processing Analysis of Graph Perception, *Journal of the American Statistical Association* 82: 454-465.

Smith SL 1979, Letter Size and Legibility, *Human Factors* 21(6): 661-670.

Sparrow JA 1989, Graphical displays in information systems: some properties influencing the effectiveness of alternate forms, *Behaviour and Information Technology* 8(1): 43-56.

Spence I and Krizel P 1994, Children's Perception of Proportion in Graphs, *Child Development* August 65(4): 1193-1213.

Spence I and Lewandowsky S 1990, Graphical Perception, J Fox and S Long (eds) *Modern Methods of Data Analysis,* Sage Publications, Newbury Park: 13-57.

Sumner DK (date unknown), *An Introduction to Graphs: General Overview.* http://www.nmsu.edu/

Tufte ER 1983, *The Visual Display of Quantitative Information,* Graphics Press, Cheshire CT.

Tukey JW 1993, Graphic Comparisons of Several Linked Aspects: Alternatives and Suggested Principles, *Journal of Computational and Graphical Statistics* 2(1): 1-33.

Wainer H 1980, Making Newspaper Graphs Fit to Print, PA Kolers, ME Wrolstad and H Bouma (eds) *Processing of Visible Language 2,* Plenum Press, New York NY.

Wainer H 1984, How to Display Graphs Badly, *The American Statistician* 38(2): 137-147.

Wainer H and Thissen D 1981, Graphical Data Analysis, *Annual Review of Psychology* 32: 191-241.

Wickens CD, Merwin DH and Lin EL 1994, Implications of Graphics Enhancement for the Visualisation of Scientific Data: Dimensional Integrity, Stereopsis, Motion and Mesh, *Human Factors* 36: 44-61.

Zacs J and Tversky, B 1999, Bars and lines: A study of graphic communication, *Memory and Cognition* November 27(6): 1073-1079.

Literature review: Techniques to test reader understanding of graphs 4

4.1 Introduction

> *When a graph is made, information is encoded on the graph by a variety of aspects ... When a graph is studied, the encoded information is visually decoded. This decoding process, which is called graphical perception, is a controlling factor in the ability of a graph to convey information* (Cleveland and McGill 1987, p. 150).

This section of the literature review outlines the studies that attempted to determine the reader's level of successful *decoding* or comprehension of graphical information. Very few studies were found that aimed to identify the successful decoding of an *entire* graph; most aimed to identify the decoding of an element *within* a graph, such as differences in the expression of a proportion between pie and bar charts (see Hollands 1992). Many studies used controlled laboratory conditions to examine response times (a measurable outcome variable) between showing a display graph and the subject's answer. Accuracy of response was also a common measure (for examples of accuracy and time measures see Cleveland and McGill 1984, Meyer *et al.* 1997, Gillan and Lewis 1994). Accuracy was determined by both quantitative assessments of the proportion of correct answers to specific questions (with right and wrong answers) and qualitative assessments rated by an evaluator. Although accuracy was frequently used as a measurement of graph understanding, not all authors were comfortable with its use. Cleveland and McGill (1984, p. 535) wrote that care should be used when using accuracy as a criterion of graph comprehension because the power of a graph was the ability it gave the reader to see trends and patterns not revealed by other data presentation techniques.

Qualitative measurements used directed questions requiring the reader to evaluate trends and relationships (Carswell and Ramzy 1997). Frameworks identifying aspects of a 'correct' open-ended response were also used to grade the answers (Carswell and Ramzy 1997, Carpenter and Shah 1998). In the course of this review, experiments were also identified using sophisticated electronic equipment, including those which used monitored eye movements to detect the location of the subject's gaze on the graph's surface (Carpenter and Shah 1998). Although the most frequently used outcome measurements were accuracy of response and time taken to register a correct answer, the review also found great variation in experimental designs for testing these and other outcomes. The variety of study designs was a significant finding because the documentation of these designs provides directional assistance to the development of *new* protocols for evaluating reader comprehension of graphs. The advantages of hybrid designs are well known.

> *…there are a number of ways that one can probe aspects of graphical perception. However, to make meaningful progress on a particular issue, one almost always has to invoke all of them* (Cleveland and McGill 1987, p. 195).

Given the range of studies in the literature, it was difficult to decide upon a logical framework for grouping these tests without duplication. Techniques to evaluate graph comprehension may have been grouped based on the cognition model they purport to evaluate. However, many of the tests were not derived from any model of cognitive behaviour while others were only loosely associated with a model of cognition. Techniques to evaluate comprehension may also have been grouped based on the method of implementing the test (study design), for example, whether subjects were recruited using random procedures, the type of instrument used to measure the subject's responses or the situation of the experiment (controlled laboratory situations versus less controlled complete-at-home surveys). Again, this was impractical: only one study was found where testing was based on population sampling techniques while the majority used some form of controlled laboratory/classroom based experiment.

The chosen framework for evaluating graph comprehension is based on the logical sequence of a process to evaluate comprehension. The sequence started with the study setting and implementation of evaluative techniques followed by the issues of subject selection and characteristics, controls for the level of complexity in the graph display, questioning techniques including the measured outcome variables, the type of analysis and, finally, the interactions identified by other investigations.

4.2 The study setting and implementation techniques

All of the studies outlined in this part of the review were based on field-tested experiments. Many were conducted on university campuses using volunteer students and controlled environments, with the investigator providing subjects with graphical display materials and instructions on how to proceed. Common to all studies were standardised methods for monitoring subjects and measuring a pre-determined outcome variable. Sampling issues were often not considered in the studies; however, a rare finding was a single population based study using random selection techniques (see Henry 1993). More on sampling issues can be found in section 4.3.

Cleveland and McGill (1987) conducted several experiments to evaluate the graph reader's understanding of a display. They synthesised the experimental process into two components: informal and formal experimentation. According to Cleveland and McGill, an example of informal experimentation would be to change one aspect of a graph and *compare the new with the old. Such a process is helpful for building intuition and can often answer questions about the ease of detection, that is, whether it is easier or harder to detect certain behaviour in the data as a result of the change in the graph. The reason why this informal process works at all is that there is a reasonable amount of uniformity in the human visual system* (Cleveland and McGill 1987, p. 195).

Formal experimentation, wrote Cleveland and McGill, is necessary when measurements for accuracy or efficiency are required. Under controlled conditions the investigator can regulate the graphical displays and make precise recordings of outcome measurements such as the length of time taken to complete a task or the length of time a graph is displayed to subjects. Specifically they had in mind experiments in visual research that show *displays for a short time period; for example displays appear on a computer screen for about 2 1/4 seconds* (1987, p. 195). In the same article they discussed an experiment to identify a subject's ability to judge slope. Using formal experimentation, their trial used a microcomputer to display two lines with positive slopes from which subjects had to detect the proportional differences in slope, using a keyboard to type in their answers (1987, p. 201). Many of the experimental designs fell into the second category of experimental design. In many ways Cleveland and McGill's (1987) computer based experiments are indicative of many trials found during this review.

One of the advantages of using computers to generate displays is the number of graphs that can be shown during a single trial session. This in turn increases the potential for testing multiple elements within the graph by showing the same data presented with changes to one element of the graph (see Simkin and Hastie 1987, below). The differences in subject response can then be evaluated.

Meyer *et al.* (1997) used computer-displayed graphics shown to their undergraduate subjects for set time intervals. Using this display technique, Meyer *et al.* were able to use one dependent variable as a function of two independent variables in each display. Simkin and Hastie's (1987) computer controlled environment was similar to Cleveland and McGill's (1985) trial in that they tested their subjects' ability to accurately judge proportions. Four groups of university students were shown pie and bar graphs and asked to identify the percentage of the larger piece represented by the smaller: the values used in the graphs were randomly generated. In total ninety graphs were generated and presented in random order to each subject.

Wickens *et al.* (1994) conducted two experiments to compare understanding between two and three-dimensional graphs. The graphs and evaluation questions were shown on a computer. After seeing the display, the subject used the keyboard to enter a response. In the first experiment subjects were asked to play the role of an economist when analysing graphs from a database. Comprehension was tested by a series of questions presented on-screen and answered using the keyboard. The evaluation also required the subject to detect a change in the data and, as an indicator of recall, subjects were finally given a written test asking them to provide detailed knowledge about the data they had viewed.

Hollands (1992) aimed to measure reader ability to identify proportions using pie and bar graphs. The experiment also used a computerised setting that served to both display graphs and record the subjects' answers. The task given to subjects was simple: identify whether the right or left hand graph showed the larger

proportion. Subjects were told that accuracy was paramount and they could take as much time as needed. Depending on the answer – entered on the keyboard – the computer responded with a chime: low for an incorrect answer and high for a correct answer.

Printed presentation booklets for displaying graphs and questions were used in several studies (see examples in Sparrow 1989 and Hollands and Spence 1992). While this mode of presentation could be expected to reduce the number of displays given to subjects, some studies, through the use of more than one experimental arm, managed to use large numbers of displays (Hollands and Spence 1992). Sparrow's (1989) experiment used a simple technique to present graphs to undergraduate subjects who were expected to be familiar with the topic of the trial: basic accountancy. Printed presentation booklets were firstly given to the subjects and, as the experiment contained an element of recall, ten minutes were allocated to inspect the display material before it was collected. Subjects then answered specific questions about the graphs. The displays were designed, not to test for differences in comprehension between graph elements but, rather, to detect overall differences between types of graph (pie, line and bar charts). Sparrow suggested that while this design might be appropriate for detecting 'overall' differences (between graph types) a finer structure would be better for identifying difference caused by changing graph elements.

A few years later Hollands and Spence (1992) conducted an experiment by preparing booklets of seventy-two printed graphs that were used for questions dealing with (i) variation in proportions and (ii) differences in rates (increasing, staying the same or decreasing). Twenty-four students were used in the trial: twelve on the proportion experiment and twelve on the rate experiment. The proportion task used six different booklets: each used only one type of graph and depicted one rate of change. The rate task was a within subject experiment using three graph types and two rates of change: 1 per cent and 2 per cent per unit time. The order of presentation of graphs was equalised across subjects.

The recording of verbalised thought patterns and answers was used in several designs (Gillan and Lewis 1994, Schnotz *et al.* 1993, Shah *et al.* 1999). Gillan and Lewis (1994) conducted an experiment with subjects selected from professional positions (engineers and researchers) and university students (psychology). The authors were investigating the processes used by people when reading graphs, hypothesising that *when people read graphs, they make use of both perceptual and arithmetic processes depending on the task and graph* (1994, p. 420).

Apart from recording the subject's verbal answers to tasks and questions, a difference in Gillan and Lewis's (1994) approach compared with most other study designs was the inclusion of naturalistic observation. As subjects interacted with the displayed graphs they were directed to give detailed verbal descriptions identifying the processes used to extract information. However, out of the twenty subjects participating in this experiment, five provided descriptions that were not sufficient for inclusion in the analysis. After describing the processes, subjects were then observed as they answered questions relating to graphs presented in print form and on computer screen. Subjects' hand movements such as pointing to elements of the graph as well as the use of pencils and spoken comments were noted.

Gillan and Lewis's (1994) experiments then used computers to (i) display three types of graph and (ii) record the subjects' answers. Subjects were told explicitly that speed and accuracy were important. Both variables were measured as part of the study's analysis (see section 4.7).

Schnotz *et al.* (1993) aimed to test the difference between how successful and unsuccessful students using text and graphs 'learned'. The measurement used in the experiment's design was the recorded verbal reasoning and referrals to the graphic given by each subject as the tasks were performed. The verbal component of the trial was followed by a quantitative method of assessing the proportion of correct answers.

Shah *et al.* (1999) presented subjects with a graphic display and then asked them to describe what they saw; these spoken comments were recorded for analysis. The graphs shown to each subject were sourced from printed literature and presented to the subjects in both their original form and in modified versions using the same data. The original version was as the graph had been published while the altered versions modified the presentation by, for example, showing proportions instead of actual values or expressing the relevant

trend as a 'data chunk' (1999, p. 693). As the experiment used the subject's verbal descriptions as an outcome measure, a coding frame was used to classify the answers based on content into four categories:

1 Within year comparisons.

2 Across years trend comparisons.

3 Mixed description.

4 Other.

Protocols were developed for coding (see 1999 p. 693) in each category. Shah *et al.* followed this experiment by a second using essentially the same format, however this time subjects were presented with a series of statements to which they were required to provide 'true' or 'false' answers.

Guthrie *et al.* (1993) aimed to identify and explore 'local' and 'global' search frameworks in two experiments. In the first, subjects were presented with four graphs and illustrations from which they could provide answers to local and global questions. Respondents were prompted to provide a verbal response to their thought patterns and answers. In the second, subjects were exposed to twenty-four tasks, with a measurement of the time required for responding to the task and the correctness of response. In each trial all subjects received the same material.

Controlled trials grouping subjects according to exposure to a changed graph element and non-exposure formed a substantial component of the studies. Often the displays were printed or shown on computer screen, however, Poulton's (1985) experiment projected graphs onto a screen approximately 1.3 by 1.3 metres. The aim of this study was to identify illusions in graphs created under certain conditions. Subjects were tested in groups of between nine and sixteen people and were asked to identify the value of specific points on the displayed graph to the nearest decimal point. Specifically the trial was identifying the illusionary effect of sloping lines in graphs with and without calibrated axes; the exposed and non-exposed subjects were therefore shown different versions of the same graph.

Random presentation of the test materials was the basis of an experiment conducted by Meyer and Shinar (1992), which aimed to identify differences in the ability to detect correlation in scatter plots between people familiar with statistics and those who were not. The experimental design involved non-random samples drawn from people with extensive statistical training (statistics lecturers and fourth year students) and people with limited statistical knowledge (undergraduates with one or two year of statistics and high school students). Booklets of scatter plots were distributed to each group. Within each booklet half of the plots included regression lines. The incorporation of these lines in plots was randomised, as was the distribution of booklets to subjects. The order of the scatter plots was also randomised within and between subjects. One subject group (lecturers) was asked to complete the task in their free time while the student group was asked to complete the task in a single classroom session.

University members (without identification of whether they were students or staff) participated in a randomised trial conducted by Greaney and MacRae (1997). The experiment was laboratory based using computer equipment to display blocks of graphs; each block contained a combination of graphs (polygon, bar etc.). Although each respondent was given the same sequence of blocks, each subject's starting point in the sequence was randomised. The computer recorded the response period from the time the presentation was displayed to the time the subject recorded an answer. Accuracy, shown by the error rate was also a measured variable.

The experiment designed by Ritter and Coleman (1995) was based on an educational test for graph understanding. The subjects were student teachers and the test questions had two versions: one said to represent a 'higher order' thought process than the other. Students were randomly selected to receive a version of the test.

The experimental design used by Lee and MacLachlan (1986) was a cross-over trial using four arms. Each arm received two versions of the test presented in controlled conditions, in this case a series of graphs in 3D and a series of graphs in 2D. The tests were provided using scattergrams and block graphs so that four combinations were possible: 3D block graphs, 3D scattergrams, 2D block graphs and 2D scattergrams. Each arm received two tests; the combinations were equalised so that each arm contained one 3D and one 2D test.

Only one population based study design was found in the literature. Henry's (1993) study involved graphs relating to educational statistics and used subjects who were randomly selected from lists of school board members, teachers, principals, school superintendents and print journalists in Virginia, USA. Four questionnaire packets were prepared and randomly assigned to the subjects. The allocation of questionnaire packets was similar to a Randomised Control Trial (RCT). The variation between the treatment arms was the content on the display for univariate and multivariate indicators (graphs and graphs; graphs and tables; tables and graphs; and tables and tables). While the author did not specify the data collection technique, it appears a mail survey was used. The overall response rate was reported as 50 per cent although no information was provided on the calculation of this rate.

Although a primary consideration in Henry's (1993) study was reader accuracy with graphs and tables (therefore not pure to graph comprehension) the questioning techniques were interesting. This was one of the few trials where the questioning technique took a holistic approach, by asking subjects to make comparisons and interpretations based on the displays they were provided. Task orientated questions were also used eg *Based on this report, does this school division do a better job of preparing students for work or preparing students for college* (Henry 1993, p. 68) as were multiple-choice questions requiring comparisons of data. Subjects were also asked for their opinion of the display's format in an open-ended question.

4.2.1 Some examples of techniques measuring accuracy and time

Kruskal's (1982) writings were based on his own literature review with no additional experimentation. He wrote that the standard techniques of measuring perception involved the recording of response time, error rate and recall. He also notes that aesthetic criteria can be used to evaluate a graph (Do you like it?), as can be the level of *insight, understanding, and discovery* (1982, p. 282), although he acknowledges they are difficult concepts to measure precisely. Although on this occasion Kruskal's comments were not put into practice, two of his criteria (time and error rate) have been used in numerous studies. 'Time' was usually measured as the period over which a specified task was completed. While Kruskal acknowledged that time was a standard measuring technique for perception, he doubted the legitimacy of its use because *one usually has plenty of time to inspect a piece of statistical graphics* (1982, p. 290). The error rate or accuracy was defined as either a deviation from the correct answer (a measurable variable) or as a correct / incorrect dichotomous variable.

The use of time as an outcome variable required a controlled testing space where accurate measurements could be made. The tests in the experiments outlined below were conducted using graphical displays, often on a computer screen. The researcher either regulated the length of time the graph was displayed for every subject or left this variable to the subject's discretion, in which case the length of time between displaying the graph and the subject's answer became the measured variable.

Cleveland and McGill (1984) provided an outline of two experiments that used accuracy to evaluate the effectiveness of particular elements of a graph eg length of a bar segment, percentage represented by a pie segment etc. The tests involved reader judgements about the graph in each display, which were printed in a booklet separate from the questionnaire. Although subjects were asked to make judgements about the graph elements and accuracy was an outcome, they were specifically instructed to make a *quick visual judgement and not try to make precise measurements, either mentally or with a physical object such as a pencil* (1984, p. 539).

As the subject was questioned on a particular column, bar segment or pie segment, the experiments identified the element (with a dot or an *x*) on which the subject was to focus. The outcome measurements were made based on accuracy and bias. Accuracy was determined by:

$$\log^2 [|\text{judged percent} - \text{true percent}| + {}^1/_8]$$

The addition of 1/8 was made to prevent distortion in the lowest errors because, in some cases, the absolute error approached zero. In a later study by Simkin and Hastie (1987) the outcome for accuracy was made using the same approach. Bias was included as a measure because the authors pointed out *subjective estimates of physical magnitudes can have systematic bias* (Cleveland and McGill 1984, p. 542). Bias was determined by:

Judged percentage – true percentage

More detail is provided on the analysis performed on these measurements in Cleveland and McGill (1984, pp. 539-44).

Meyer *et al.* (1997) also used outcome variables that were based on the subject's reaction time and number of correct answers. Reaction time was measured from the time the data appeared on screen until the subject articulated a response. Unlike Cleveland and McGill's (1984) study that measured the size of the error variable, Meyer *et al.* assessed accuracy as a yes / no dichotomous variable, except when answers could identify the size of the error, in which case the analysis was as implemented by Cleveland and McGill (1984).

Explicitly informing subjects of the measured variables was a characteristic of Gillan and Lewis's (1994) experiment where subjects were told that speed and accuracy were important. While both variables were measured as part of the study's descriptive analysis, the regression analysis used only speed as the dependent variable.

Poulton's (1985) trial, using projected displays, was specifically interested in accuracy. The investigator identified a point on a plotted function and subjects were asked to determine the value of the point to the nearest decimal place. Accuracy was measured by the difference between the actual value of the point and the subject's answer. A measurement was also made for the time each slide was required to be projected for each group. A second similar experiment used display cards from which the subject was asked to identify the 'y' and 'x' axis values.

Despite the effort they placed into measuring and analysing accuracy, Cleveland and McGill (1984, p. 535) stated:

> *One must be careful not to fall into a conceptual trap by adopting accuracy as a criterion. We are not saying that the primary purpose of a graph is to convey numbers with as many decimal places as possible. We agree with Ehrenberg (1975) that if this were the only goal, tables would be better. The power of a graph is its ability to enable one to take in the quantitative information, organize it, and see patterns and structure not readily revealed by other means of studying the data* (Cleveland and McGill 1984, p. 535).

In other words accuracy in interpreting specific data is not the only way to judge a graph's effectiveness. Being able to identify trends and patterns is also important.

Cleveland and McGill (1984) also highlighted another danger that can occur when evaluating the reader's understanding of a graph: that is, subject's performance under the conditions of the trial might be different from the way they would behave in the absence of those conditions. They argued that:

> *One substantial danger in performing graphical perceptual experiments is that asking people to record judgements will make them perform judgements differently from the way they perform them when they look at graphs in real life* (Cleveland and McGill 1984, p. 553).

To guard against this occurrence, they suggest encouraging *subjects to work quickly, much as they might in looking at a graph in real life* (Cleveland and McGill 1984, p.553).

4.3 Selection of subjects

The selection of subjects for experiments involved issues of (i) the pool from where subjects were selected (usually university), (ii) the required physical requirements (eyesight), and (iii) previous experience interpreting graphs.

Pool for subject selection

Most studies were conducted using subjects who were attending university; usually psychology or science students (see Meyer *et al.* 1997, Sparrow 1989, Schnotz *et al.* 1993, Carpenter and Shah 1998, Shah *et al.* 1999, Wickens *et al.* 1994, Poulton 1985, Hollands 1992, Guthrie *et al.* 1993, Meyer and Shinar 1992, Lee and MacLachlan 1986, Hollands and Spence 1992).

A smaller number of studies selected samples from outside of student populations. Gillan and Lewis (1994) conducted their experiment with at least some subjects selected from professional positions (engineers and researchers) and university students (psychology). Other studies using broader sample bases included Cleveland and McGill (1984) and Henry (1993). Both are discussed below.

Large samples were not common, with most studies using between ten and twenty subjects. Some notable exceptions were Poulton's (1985) trial that used ninety-four subjects drawn from Cambridge University's Applied Psychology Unit subject panel. No information was provided on the characteristics of the sample (e.g. students, staff or other volunteers). Holland's (1992) experiment used thirty-two undergraduate students; Guthrie *et al.* (1993) conducted two studies, the first using sixteen students and the second, designed to test the outcomes of the first, used fifty-five undergraduates. The experimental design used by Lee and MacLachlan (1986) used forty-five students who were randomly divided into one of four arms. Culbertson and Powers' (1959) study with three-hundred and fifty students (see below) had the largest sample size of any experiment in the review.

Meyer and Shinar (1992) recruited from four populations in their experiments. Their first experiment used ten lecturers and nineteen first and second year students. A follow-up experiment used 49 students drawn from fourth year university and high school: the results of each group were compared, however the authors did not identify the individual numbers for fourth year university or high school students.

Leinhardt *et al.* (1990) conducted a study dealing with learning and testing for comprehension using a target group of school students aged nine to fourteen. Despite the young age group, the study was included in this review because of the questioning technique they employed, particularly for qualitative issues (see section 4.6).

Culbertson and Powers' (1959) study used one hundred students on a Farm Short Course (aged 18 to 24) and a second group of two hundred and fifty school students. All subjects were tested for verbal reasoning, numerical reasoning and abstract reasoning; the results to these tests were used as variables in the analysis. Wickens *et al.* (1994) also used indicators of ability, not only for use as a covariate but for subject selection. After the administration of standardised tests on spatial abilities, subjects scoring below a certain level were excluded from the trial.

Cleveland and McGill's (1984) two experiments evaluated the effectiveness of elements of a graph eg length of a bar segment, percentage represented by a pie segment etc. Their study was one of few that selected subjects based on their familiarity with graphs, resulting in groups of technical and non-technical subjects. The non-technical subjects were predominantly female and mostly housewives; the technical subjects had employment that required frequent use of graphs and were a mixed group of males and females. As there were no statistically significant differences in the measurement of accuracy between the technical and non-technical subjects, they were ultimately combined and treated as a homogenous sample.

Henry's (1993) study testing comprehension of educational graphs was one example where subjects were randomly selected from five defined populations. Each population group was identified from available lists that also provided the sample frame for random selection. The groups were: school board members, teachers, principals, school superintendents and print journalists in Virginia, USA. These five groups were also included as a variable during analysis to test for differences between groups. Henry's study was also in the minority of studies (the only one) that included a response rate for each group of subjects and for the study overall (50 per cent). As might be expected, the lowest response rate among the five groups was obtained from the group most removed from the education system (print journalists).

Physical requirements of subjects

The selection of subjects for experiments usually started with basic physical requirements of normal vision or corrected to normal vision (Carswell 1991, Carswell *et al.* 1991). For studies using colour displays, subjects had normal colour vision (Casali and Gaylin 1998). In one trial (Wickens *et al.* 1994) subjects who required eyeglasses were excluded from the trial because the use of three-dimensional test equipment was compromised by eyewear.

Prior knowledge of the subject area and/or prior experience using graphs

Study subjects were frequently university students. Of these most were undergraduates, but sometimes postgraduates were used. The distinction between the two was the level of experience in using graphs. In the studies that treated experience as a variable, a higher level of education was assumed to assist the graph

interpretation tasks. However, Cleveland and McGill (1984) included 'experience' as a variable by sampling technical and non-technical subjects. They found no statistically significant differences between the two groups based on accuracy and ultimately joined the sample (see section 4.8 on confounders). Meyer and Shinar (1992) also selected their sample based on the level of prior knowledge. In the first of two experiments the subject groups were chosen based on the level of statistical knowledge: those familiar with statistics (lecturers) and those who were unfamiliar (students). The analysis (see section 4.7) provided a comparison of results between the two groups. In a follow-up experiment two other groups were introduced, also based on statistical experience: fourth year students (with significant statistically based coursework) and high school students.

Instead of selecting subjects based on their prior experience with graphs or data, Carpenter and Shah's (1998) study used graphical information that was not common knowledge so that subjects' prior experience with the data was unlikely. Rather than use an unknown topic for the test graphs, Culbertson and Powers (1959) used a topic that was familiar to the subjects (farming). However, the test graphs contained fictitious data and subjects were told to answer from the graphs, not from their own knowledge of the topic.

Eggan *et al.* (1978) partially controlled the level of prior knowledge in their evaluation by presenting information to subjects that was 'reasonable' but also 'counter intuitive'. This was specifically done to prevent subjects answering the questions based on prior knowledge.

4.4 Training subjects in the experiment's tasks

Training was an element in several studies to ensure all subjects understood the requirements of the experiment. As part of Cleveland and McGill's (1987) experiment they commented on the issue of training: specifically that suitable instruction might help to reduce the variability of response within the sample. They stated:

> *an experimental protocol that provides careful instructions and thorough training to subjects and that motivates them to concentrate on the task can reduce the noise substantially* (Cleveland and McGill 1987, p. 195).

They also provided advice on how to encourage subjects by:

> *conveying a sense of importance of the experiment to them, rewarding them for participation, and introducing a competitive aspect of the tasks in which each subject competed against his or her earlier performance in the experiment* (Cleveland and McGill 1987, p. 196).

In 1961 Schutz's experiment for detecting trends in graphs set a high standard for ensuring all subjects were instructed on the experiment's rules. Testing in Schutz's (1961) design did not start until subjects achieved 100 per cent accuracy in knowledge of the rules. Additionally they could ask questions at any time during the experiment. This level of instruction would be difficult to replicate outside a controlled environment, so its use in a mail or telephone based survey would be limited. Nonetheless, it provides an important reminder that the subject's awareness of the experiment's instructions may be a factor influencing the variability of responses.

Casali and Gaylin (1988) provided pre-trial training of subjects in their experiment's interpretation tasks. Training took place before the trial and each subject received feedback from the training questions as to whether their answers were correct or incorrect.

Ferry *et al.*'s (1999) experiment found that subjects' required basic help when interpreting graphs during the experiment. However, they found that context-specific help was sufficient. While the study provided 'on-site' help, text accompanying a graph might also provide the required 'context-specific' assistance that some graph readers could utilise. They found that pre-service teachers misinterpreted graphs because they did not realise that a relationship existed and often did not read the graph axes.

> *However, once the researcher directed their attention to the relevant local or global feature most could choose the correct answer. Thus in many cases context-specific support was all that was needed* (Ferry *et al.* 1999, p. 6).

Shah *et al.* (1999) instructed subjects on the tasks required and then provided them with a simple line graph for task practice. Holland's (1992) experiment used a computer sounding a tone for correct or incorrect answers, therefore, subjects could learn as they progressed in the trial.

Guthrie *et al.* (1993) ran a tutorial prior to the trial to ensure all subjects were familiar with a computer keyboard. No other instruction or practice questions were provided.

Instructions on the booklet cover were used by Meyer and Shinar (1992), however, as a second experiment conducted by these authors involved high school students, this group also received the same instructions and a fifteen minute training session about scatter plots and correlation.

The experimental design used by Lee and MacLachlan (1986) provided a warm-up period for subjects to become familiar with 3D graphs and the trial equipment. Hollands and Spence (1992) provided a practice booklet for subjects for completion prior to the trial. Subjects received feedback from the investigator on completion of the practice session.

The qualitative assessment used by Carpenter and Shah (1998) incorporated subject instruction intertwined with the evaluated test. Subjects were presented with sixteen core graphs; and an additional twelve 'filler' graphs used for instruction. Only the answers provided to the core graphs counted toward the subject's evaluation. The lower difficulty of the filler graphs was a deliberate strategy to minimise participants' frustration. To keep subjects focussed on the trial's main theme (identifying relationships), they were told the values shown in the two graphs need not be identical (although they were).

4.5 Complexity in the graphical display

While complexity in the graphical display was commented upon in many studies, it was only *controlled* in a few. Part of the reason for the low level of attention to complexity may have been due to uncertainty in its definition. Casali and Gaylin (1998) attempted to provide some outline of complexity but they acknowledged the definition is open to interpretation.

> *Complexity level is in the eyes of the beholder and may be a function of many variables such as the number of data sets represented, the precision of information to be conveyed (e.g. exact versus ball-park), the number of intersecting or overlapping graph lines, the resolution of the scale, the distance from the ordinate to the graphed data and many others* (Casali and Gaylin 1998, p. 37)

The experiment conducted by Casali and Gaylin (1998) was a within-subject fixed variable with two levels of complexity, set at 'low' and 'high'. All low complexity data sets had three series (e.g. three lines, sets of bars) per graph. The questions were also easier: identifying the maximum or minimum, determining which set of data was increasing or decreasing, and trend questions which asked the reader to determine the data set that was increasing or decreasing the most. In the high complexity graphs five or six series were used and contained more difficult questions: whether the data value was the second, third etc. highest value within a given set and trend questions requiring the reader to identify which series was increasing or decreasing at certain time intervals.

In Meyer *et al.* (1997) complexity was similarly coded as either 'low' or 'high' and was defined in terms of the regularity of trends or the number of data points showing the various level of the dependent variable. Carswell and Ramzy (1997, p. 62) controlled for complexity by the number and type of departures from linearity.

4.6 Questions and questioning techniques to evaluate comprehension

The underlying method for evaluating graph comprehension used in many of the studies reviewed was to show the subject a graphical display and then ask questions based on that display. With the exception of Bertin's (1980) and Gillespie's (1993) untested recommendations, the approaches documented in this section were used in study designs. However, the use of questioning techniques in experimental studies does not guarantee a *good question* or a *good questioning technique,* although, to help those following in their footsteps, it is hoped that authors would have noted relevant limitations or flaws identified during their trials.

Bertin's (1980) theme was that a graph's main aim is to answer two questions, and if it does that the graph is successful. Bertin's questions, which he claimed were the basic test for graph understanding, were:

a *What are the x and y components of the table of data?*

b *What are the groups of elements in x and the groups of elements in y that the data generate?* (Bertin 1980, p. 585).

These general prescriptions have been taken further and explored in more detail by other authors; nonetheless they are a starting point. Bertin identified two questions that should not be asked because they are not related to the aim of the graph and will, apparently, lead to confusion and error:

a *What do you see?*

b *What do you prefer?* (Bertin 1980, p. 585).

While this might be true, as noted, Bertin's recommendations were never tested on subjects and therefore there are no outcomes suggesting whether they are (are not) successful evaluators of graph understanding. Gillespie's (1993) point is that questions used to evaluate comprehension of written text are not so different from the questions that evaluate a graph. She says the common questions are:

What is the main idea?

What are the supporting details?

What is the purpose of the graph?

How are the details inter-related?

What are the meanings of the symbols?

Such questions may ask students to extrapolate, interpolate, determine trends, compare amounts, determine differences, determine purposes, summarise, make projections, analyse data, draw conclusions and solve problems (Gillespie 1993, p. 352).

Although Gillespie is not citing questions she used with subjects, there is common ground with other studies that have used similar comprehension questions in the field.

Gillespie's thoughts are replicated in the themes identified elsewhere in the literature. In essence, these are that the questioning techniques move from evaluations of components of the whole toward an overall evaluation of the whole. This process has different labels in the literature. Leinhardt *et al.* (1990) call it the 'local / global' framework, Wickens *et al.* (1994) refers to the process in terms of the level of integration while others (see Casali and Gaylin 1988) use no name, although the same process is used: moving along a scale from low complexity questions asking about specific issues (e.g. point reading) to more complex concepts requiring the reader to identify trends and relationships.

Using a model of cognition, Casali and Gaylin (1988) identified four basic interpretation tasks required from the graph reader. These tasks, from simple point reading to more complex trend interpretation, were then investigated by specific questions. The interpretation task (italicised) and the type of question that might be asked follows:

a *Point reading*
eg an exact numerical value.

Point comparison
eg evaluating two or more points and determine the greater than or less than relationship.

c *Trend reading* e.g. detecting increasing, decreasing, cyclical or constant trends over time.

d *Trend comparison*
eg the discrimination between greater than and less than relationships between two or more data sets over time; (Casali and Gaylin 1988, p. 35).

The last component of Casali and Gaylin's tasks (trend comparison) was the focus of Carswell *et al.*'s (1991) experiments where subjects performed two tasks aimed at identifying the level of understanding of trend directions. In the first task, subjects were given a deck of thirty graphs and instructed to create two piles of graphs: one for graphs showing increasing trends and the second for graphs with a decreasing trend.

The second task required the subject to identify whether the trend changed direction. While proceeding through the deck, subjects were told to speak out when the trend changed direction and to identify how the direction changed (e.g. from increasing to decreasing or vice versa). Carswell (1991) also tested reader detection of magnitude by asking subjects to determine the size of one graphical element relative to another.

The experiment conducted by Carswell *et al.* (1991) aimed to gauge whether 2D or 3D graphs are better at enabling subjects to retain information over time. Subjects were shown a slide show of various graph types with statistics addressing a variety of issues and then required to complete a quiz that included questions on specific values and trends.

Wickens *et al.* (1994) used three question techniques in their trial. They termed questions displayed on the computer screen as 'on-line' questions that could be answered from the on-screen graphical display. Three

groups of questions were used that differed in the level of attention and information integration:

a Low information integration questions were specific to one category of an outcome variable (*What is the earnings value of the blue company?*).

b Medium information integration used two outcome variables (*Is the green company's debt value higher than its earnings value?*) or focussing on one outcome variable and two independent variables (*how much greater is blue's price than red's price?*).

c High information integration, more than one outcome and independent variable (*Which company has the highest total value of all three variables?*) (Wickens *et al.* 1994, p. 48).

Leinhardt *et al.* (1990) conducted a study dealing with both the learning side of graph understanding and the tests that could be used to evaluate comprehension. Although school students aged nine to fourteen were the target group, the testing suggestions may be useful because of the applicability of the model. Leinhardt *et al.'s* (1990) 'local' and 'global' approach was not unique in the literature; later researchers, including Guthrie *et al.* (1993) and Meyer *et al.* (1997), cited below, have used similar models.

Leinhardt *et al.* (1990) say that interpretation is a process whereby the student makes

> *sense or gains meaning from a graph (or portion of a graph) …. Interpretation can be global and general or it can be local and specific. Thus a student may be trying to decide issues of pattern (eg What happens to x as y increases?), continuation (eg interpolation or extrapolation of a graph), or rate (eg How do the bacteria change every 5 hours at a fixed temperature?); or determining when specific events or conditions are met (eg What is the minimum?)* (Leinhardt *et al.* 1990, p.8).

Leinhardt *et al.* believe that qualitative interpretations of graphs are also important. Specifically, this requires looking at the whole graph and assessing relationships between variables and detecting patterns. While global features can be assessed quantitatively or qualitatively, they say local features are usually only assessed quantitatively (Leinhardt *et al.* 1990, p.10).

To test qualitative knowledge, Leinhardt *et al.*'s questions asked the reader to identify which graph best described a given statement. An example provided in their paper showed four graphs: where the 'y' axis showed the *height of plants* and the 'x' axis the *size of pots*. The reader was asked to identify the graph that best represents the statement, for example: choose a graph that shows … *As the pot size increases, the plant height decreases* (1990, p. 11).

Guthrie *et al.*'s (1993) study also explored the local and global search frameworks and they used a qualitative technique allowing open-ended answers. By using two different types of question they evaluated the subject's understanding of the graph's local and global functions. One set of questions was specific to local tasks and a second set specific to global tasks. Four graphic illustrations were given to subjects accompanied by questions. The local search questions included:

> *How many American made cars were sold in June of 1989?*
>
> *What frequency is the Horseshoe Bat most sensitive to?* (Guthrie *et al.* 1993, p.194)

The global search questions included:

> *What is the pattern of US vehicle sales over the course of one year?*
>
> *How does the auditory reception of the three bats differ?* (Guthrie *et al.* 1993 p.194)

Each subject was asked to verbalise their thinking as they formulated their answers. The subject's spoken comments and final answers were tape-recorded for later coding. The coding frame for the open-ended responses was based on:

a *Goal formation* that included the subject's verbalising of the question and the sub-components of the question.

b *Category selection* that included elements of the graph referred to by the subject, such as a particular column.

c *Element extraction* that included comments indicating the subject was accessing an element in the graph such as an axis label and encoding the information.

d *Integration* that included comments indicating the subject was combining information from the graph and prior knowledge on the topic.

e *Self correction* that included comments indicating the subject was correcting an earlier statement.

f *Reading text* that included direct reading of text in the graph such as headings.

g *Inferences* that included statements indicating the subject was identifying casual or logical relationships in the graph.

h *Simple abstraction* was the *lowest level of abstraction made by subjects,* for example describing a trend for a single category

i *Complex abstraction* consisted of *higher order rules and generalisations that summarised relations across two or more categories of information within a graph or illustration* (Guthrie *et al.* 1993, p. 193).

After completing their first trial, Guthrie *et al.*'s (1993) second experiment also used a coding framework for classifying subjects' answers, however, this time the framework was collapsed into four categories that ranged from level 1 answers that were incorrect in every respect to level 4 answers that were correct and accurate with supporting details and explanations.

Carswell and Ramzy (1997) also used qualitative techniques. In their experiment, subjects gave written assessments of the data sets that were scored for:

1 *overall number of propositions pertaining to the data set as a whole (global content)*

2 *number of propositions describing relations within a subset of the data (local content)*

3 *number of references to specific data values (numeric content)* (Carswell and Ramzy 1997, p. 61)

The experiment also tested accuracy and speed of the subject's response to *directed* questions. These questions required specific answers and were scored on accuracy and speed of task completion. The difference between the two questioning techniques was described, allowing subjects to choose the information they take from the display as opposed to prompting subjects to extract specific information (Carswell and Ramzy 1997, p. 62).

Carpenter and Shah (1998) also used an experiment where the subjects' qualitative answers were evaluated. In this study, students were used as subjects and were selected based on the level of previous experience using graphs. One group consisted of less experienced undergraduate students and the second group consisted of more experienced graduate students.

The qualitative assessment used by Carpenter and Shah (1998) incorporated subject instruction (see section 4.4). Subjects were presented with sixteen core graphs that counted toward the subject's evaluation. An additional twelve graphs were presented as fillers; these graphs did not count toward the subject's evaluation. The presentation method was to display a graph to the subject who would then articulate a description. The graph was then removed and a second graph displayed. Subjects were asked if the relationships shown in the first graph were similar to those shown in the second graph. To focus subjects on the theme of the graph, the investigators told subjects that the graph values did not have to be identical (although they were). The subjects' answers were then rated on content using a classification system for each variable in the graphs. A second judge coded about half the descriptions and the two judges' scores were compared.

In a later series of experiments Shah *et al.* (1999) simply asked subjects to describe what they saw when presented with a graph. Each subject was shown multiple graphs differing in presentation but based on the same data set The subjects' answers were placed into four categories using a coding framework. In a second experiment, statements to which subjects were required to answer 'true' or 'false' accompanied the graphs.

Culberston and Powers (1959) designed twenty-five graphs that were all based on the same model graph but each changed to disguise the similarity and manipulate the graph variables. Each graph had four units and each unit was broken into three elements. An element was the smallest part of the graph: a line, bar segment, pie segment etc. A unit was a group of three elements eg three bar segments = one unit. Each graph was presented to subjects with a series of four to seven multiple-choice questions. The questions required the reader to interpret the graph by making evaluations or comparisons between elements. The questions required the reader to:

a *estimate the relative length of four units*

b *estimate the quantity of an element originating at the zero line of the graphs*

c *estimate the quantity of an element originating at some point other than zero on segmented graphs*

d *judge the relative length of two different elements within the same unit*

e *judge the relative length of two differently labelled or keyed elements in different units*

f *judge the relative length of two similarly labelled or keyed elements in different units*

g *judge the difference between two differently labelled or keyed elements in different units* (Culbertson and Powers 1959, p. 98).

The test administered in this study allowed for two or more controlled comparisons on eleven graph variables (outlined below); the comparison graphs were identical except for one variable.

a Identification of elements: by (1) labels, (2) keys and (3) pictorial symbols.

b Presentations of quantities by figures on (4) elements and by (5) a grid on the graph axis.

c Bars or lines by using (6) discrete bars and (7) continuous lines.

d Comparison between (8) segments arrangements (parts of a total) and (9) grouped (all elements start at zero);

e Two ways of presenting parts of a whole using (10) pie charts for percentages and (11) segmented bars for percentages.

The comparisons were made using t-tests on differences between the mean scores of each group (for more on analysis techniques see section 4.7) (Culbertson and Powers 1959).

The development of questions addressing reader comprehension by Meyer *et al.* (1997) could be described as a graduated version of the 'local' / 'global' framework. The authors identified what they saw as five different variables displayed in the graph and sought to test these variables using five levels of questioning. The category of question (in italics), sourced from Meyer *et al.* and some examples of population health questions that might be used follow:

a *Reading the exact value of a single point* (eg How many Hepatitis A notifications were recorded in year 3?).

b *Comparing two points that belong to the same data series but that have different values on the x axis* (eg When were more Hepatitis notifications recorded, in year 3 or year 4?);

c *Comparing two points that have the same value on the x axis but that belong to different data series* (eg Were more Hepatitis A or Hepatitis C notifications recorded in year 3?)*;*

d *Reading the trend of a data series* (eg Is the general trend of Hepatitis notifications increasing or decreasing?)*;*

e *Identifying the highest value of a data series* (eg In which year was the largest number of Hepatitis notifications recorded?) (Meyer *et al.* 1997, p. 272).

In an earlier experiment, Meyer and Shinar (1992) asked subjects to identify the estimated correlation (as a specific value) for each displayed scatter plot. The questionnaire was a printed booklet with a random insertion of regression lines into the graphs and a random allocation of various scatter plot distributions. In this design, the question presented to each subject was identical, however, the graph associated with each question was randomised between subjects in each of four subject groups (lecturers, first/second year students, fourth year students and high school students).

Greaney and MacRae (1997) asked their subjects to identify out-of-range (outside specific limits) variables in sequences of displays on a computer screen. Although not explicit, it appears that the trial did not use written questions, rather the subject, through manipulation of a computer mouse, identified out-of-range points.

The questioning technique used by Ritter and Coleman (1995) used two tests, each containing different versions of one question: one version was said to represent higher order thought processes. The tests were administered at the beginning and end of the student term. An interesting aspect of this technique is that subjects selected a graph providing the best fit for the displayed *data*.

The questioning technique used by Simkin and Hastie (1987) asked subjects to make either a discrimination comparison or a proportion judgement. A computer was used to present the displays and record subjects' answers: time and accuracy of response were measured.

The experimental design used by Lee and MacLachlan (1986) used a recorded voice to ask respondents to provide an answer to a business scenario: the question needed the subject to refer to a displayed graph for the

answer. The experimental design alternated the graph type between 2D and 3D images and between a scattergram and a block graph. The point of the pre-recorded voice was to ensure that the question was asked of all groups in exactly the same way.

Hollands and Spence (1992) conducted a similar experiment using questions to test for subject identification of variation in proportions and differences in rates (increasing, staying the same, or decreasing). For the proportion task, respondents were given a base question *What proportion is P of the whole at time T?* (p. 317) which varied in the complexity of calculating the proportion (see pp. 317-18 for more detail). There were variations in the graphs used in the trial: there were six booklet types, each contained a graph of one type and depicted one rate of change. The rate of change task used a within subject design, three graph types and two rates of change.

In the same year Hollands (1992) published a study where subjects completed a straightforward task: identify which of two graphs showed the larger (absolute) proportion. The computer responded with tones for correct and incorrect answers so there was a learning component for subjects during the trial. In a second trial the question was more discriminatory: which is the larger proportion with respect to the whole.

4.6.1 Questions using a holistic approach for evaluating graph comprehension

Rather than graph elements, the questioning technique used by Henry (1993) was one of few that took a holistic approach and tested the reader's ability to decode information to answer overall questions about the displayed graph. The study aimed to identify differences between the comprehension of tables and graphs and used a variant of the 'local' / 'global' framework. For example, task-orientated (global) questions were used such as *Based on this report, does this school division do a better job of preparing students for work or preparing students for college?* (Henry 1993, p. 68). More data specific questions were included as multiple-choice questions that required comparisons of data. Subjects were also asked for their opinion of the display's format in an open-ended question.

Henry's (1993) trial also presented subjects with a graph and then asked them to identify which, of a series of statements, best represents the information shown in that graph. This was also the approach used by Eggan *et al.* (1978), who prepared five bar graphs showing the results of a bean growing experiment. Each of the graphs showed one variable that affected plant growth and were constructed so that the amount of information in each chart was equalised. The subjects were given a one-page sheet describing the plant growing experiment and an associated graph, which was the subject of the test. Evaluation took two forms. Firstly, the subject was asked to *read two generalisations and indicate whether one, both, or neither were true, based on the data in the graph* (Eggan *et al.* 1978, p. 212).

The second level of the evaluation involved a group of four questions that addressed the subject's general examination of the graph. The questions addressed the following issues:

a *Mid specific* – asked about the height of the plants at the end of one or two weeks

b *End specific* – asked about the height at the end of the experiment

c *Conditional* – asked about the relative effectiveness of different growing conditions

d *Generalisation* – required students to judge the validity of generalisations about the experiment (Eggan *et al.* 1978, p. 212).

The experiment conducted by Sparrow (1989) stated that the study design was to test for overall differences in comprehension between different types of graph (specifically pie, line and bar graphs) and would not be appropriate for identifying differences between graph elements. Nonetheless, the 'local' / 'global' framework is evident in the question design. Sparrow's trial used printed forms containing the test graphics, as opposed to the more controlled computer experiments popular with other authors. Subjects were undergraduate students in accountancy who were expected to be familiar with the topic of the display.

Display booklets were given to subjects and as the experiment contained an element of recall, subjects had ten minutes to digest the material before they were collected. Subjects then answered specific questions about the display.

Sparrow (1989) categorised his questions into the following groups:

> *Information about specifics*:
> eg How much did product Y sell in 1985?
>
> *Information about limits*:
> eg In which year were product X's sales at their lowest? Which product sold more in 1985?
>
> *Information about conjunction*:
> eg In which year did product Y first sell more than product X?
>
> *Information about accumulation*:
> eg In which year were total sales (for products X, Y and Z) highest?
>
> *Information about trends*:
> eg Which product's sales would you describe as generally rising?
>
> *Information about proportion*:
> eg What proportion of product Y's sales were made in 1985? (Sparrow 1989, pp. 53-54).

The technique used by Sparrow also meant that the type of graph provided to subjects could not answer some questions. He said:

> *the ten subjects in the pie chart group were unable to give specific information (eg How much did Product 4 sell in 1984?) since this information was not abstractable from pie charts. This group performed the worst in this respect.*
> (Sparrow 1989, p. 53).

Asking questions that cannot be answered from the display material may not be a worthwhile method for evaluating comprehension.

Not all authors were convinced of the acceptability of the holistic approach. Cleveland and McGill (1987), who have considerable experimental publications in this field, argue that trials using whole graphs (for example comparing pie with bar graphs) would mean every issue would require a new experiment and deduction. A better approach to take, they say, is breaking the complex structures of graphs *into smaller pieces, attempting to understand the pieces, and then inferring the properties of the graph forms from an understanding of the pieces and their interactions* (Cleveland and McGill, 1987, p. 196).

4.7 Analysis techniques

In Henry's (1993) study the collected results were examined using analysis of variance techniques and tested for possible interactions. Accuracy was measured as a percentage of correct answers. Opinion of the display was measured by whether the subject 'liked' it; the between-subject study design did not allow for within subject comparison of graphs and tables displays.

Wickens *et al.* (1994) used analysis of variance on the response times between the graph display and providing an answer. Apart from time, the other variables were display type and level of animation used. In their two trials, the analysis performed by Meyer and Shinar (1992) used four-way analysis of variance. The group (lecturer versus student or fourth year student versus high school student) was a between-subject variable while the components of each test (correlation level, shape of the data point cloud and presence or absence of a regression line) were within-subject variables. The dependent variable was the mean estimate of correlation. Comparisons were made between the subject's estimated value for correlation, the actual correlation value and between the values (or ranges of values) provided by each group. Greaney and MacRae (1997) also used analysis of variance based on repeated measures of the mean change in each subject's score for each experimental factor.

The analysis conducted by Hollands and Spence (1992) was based on measurements of time to complete the tasks (measured by a stopwatch) and accuracy, which was based on the mean number of incorrect judgements. The task assessing rates, was analysed using a two-way *(graph-type x rate of change)* (1992, p. 318), within subject analysis of variance.

Meyer *et al.* (1997) analysed reaction times through a five-way analysis of variance using: display (table, line graph, bar graph), task (as per above questions), complexity (high or low) and experimental block (one of three) as independent variables. The three blocks each contained thirty trials that covered all possible combinations in the displays. Accuracy was analysed as a dichotomous variable using logistic regression. The exception was for questions on trends that, at times, approached 100 per cent accuracy; only percentages of correct answers were presented (Meyer *et al.* 1997).

In the computer-based experiment conducted by Gillan and Lewis (1994) three types of graph were presented to respondents and each graph had four versions. The analysis provided basic descriptive statistics and regression analysis using response time as the dependent variable and the tested processing steps as the independent variables.

Shah *et al.* (1999) asked subjects to describe what they saw when presented with a graph display; these answers were then coded. A second experiment presented a series of statements with each graph to which subjects answered 'true' or 'false'. The authors were not clear in the specific statistical technique used to compare the answers, however, it was apparent that the test statistic was the *proportion* of subjects rating statements correctly or providing a certain coded description.

Poulton's (1985) trial requiring subjects to identify the exact value of a data point measured the difference between the true value of the data point and the subject's estimate. The resulting errors were the basis of analysis (see also Casali and Gaylin 1988).

The analysis performed by Guthrie *et al.* (1993) in the first of two trials used significance tests to compare means. The statistical process was guided by five questions:

Question 1 Do local search tasks evoke more reports of category selection than global search tasks?

Question 2 Do global search tasks evoke more reports of abstractions than local search tasks?

Question 3 Are student reports of component processes in the extended cognitive model positively correlated with the quality of their answers to questions?

Question 4 What are the levels of performance on search of graphs and illustrations?

Question 5 What are the main processing deficits of students who perform poorly on global search tasks? (Guthrie *et al.* 1993, p. 192).

The second trial aimed to detect differences between local and global abstractions in the task presented to subjects. Factor analysis was used with a two-factor solution to test the independence of each local and global task. A test to detect the difference between the performance (based on the proportion of correct answers) on local and global tasks used multivariate analysis of variance.

Ritter and Coleman (1995) used pre- and post-testing of subjects using two tests that were based on the same data, although both had different question content. The 'exposure' was not explicitly identified in the study. It appears to have been, however, the student instruction provided during the term. The pre- and post-test results were compared using Fisher's Exact test.

The experimental design used by Lee and MacLachlan (1986) analysed the accuracy of each response as well as the time each subject required to provide a response. For qualitative analysis, the trial also recorded the comments made by subjects during the trial. Quantitative assessments were made using means and a two tailed t-test.

4.8 Potential interaction

Culbertson and Powers (1959) identified and measured potential interaction in their study. Subjects' aptitude scores were measured and correlated with graph comprehension scores. There was a moderate correlation between aptitude and graph comprehension when aptitude was measured by verbal reasoning, numerical reasoning and abstract reasoning. A statistically significant relationship existed between each area of aptitude and graph comprehension. The authors also found that the score for any of the three aptitude tests was related to graph comprehension to about the same degree that it was related to the other aptitude scores. However, there was no significant correlation between aptitude scores and any particular graph variable (ie a line graph versus a bar graph).

Ferry *et al.* (1999) also identified potential interaction in terms of educational background. They found that subjects with a science background were better able to interpret graphs than students from other faculties who were enrolled in teacher education. Although the study found that formal studies in education had no significant effect on the ability of students to interpret graphs *preservice teachers who have completed an undergraduate science degree prior to commencing the teacher education program were better at interpreting graphs… than those who have not completed such a degree* (Ferry *et al.* 1999, p. 5). This does not confirm the conclusion drawn by Cleveland and McGill (1984) that there was no statistically significant difference in the measurement of accuracy between the technical and non-technical subjects. Therefore, it should caution against the combination of technical and non-technical subjects as though they were a homogenous group.

4.9 Recommendations

The studies included in this review provide guidance for the current research to evaluate reader comprehension of graphs amongst members of the employed health workforce in NSW. More specific detail on the research can be found in Volume II.

The following recommendations were based on the studies included in the review. The evidence level of these studies (1 to 3) has been identified and a brief discussion of relevant issues follows each recommendation.

4.9.1 Recommendations for study design

- **A multiple number of treatment arms in a Randomised Control Trial (RCT). Recommend two arms in the RCT:
Arm 1 the controls –
the published graphs (no modifications)
Arm 2 the treatments –
the published graphs with selected 'best practice' and other modifications**

Several studies used a RCT for the experimental design (see Meyer and Shinar 1992, Henry 1993, Lee and MacLachlan 1986). The studies did not explicitly state why this design was chosen, however a key advantage of a RCT is the expectation that known and unknown confounders will be randomly allocated between arms. Confounders were specifically identified by Culberston and Powers (1959) and Ferry *et al.* (1999). They included aptitude scores in verbal, numerical and abstract reasoning and educational background.

There was limited discussion on the optimum number of treatment arms that might be used. However, Sparrow (1989) commented that trials showing subjects the same information (i.e. the same data) presented in different graph styles (pie, line, bar etc.) might be sufficient for determining the difference in comprehension between graph type, but would not identify differences in specific graph elements. If the study aims to determine specific elements that increase reader comprehension, the logical conclusion is that multiple forms of the one graph must be produced, each having a changed element. This was implicit in many of the studies, particularly those using computer displays. However, Hollands and Spence (1992) and Meyer and Shinar (1992) used both a study design that varied elements in the display graphs and used printed booklets. Henry's (1993) study (also using printed booklets) was a RCT with each arm of the trial having a variation in the display material. The limitation in Henry's (1993) study to these recommendations is that the design was testing for differences in comprehension between graphs and tables.

The choice for the study design becomes one of choosing the number of treatment arms and cost. More treatment arms allow more elements to be individually tested. However, this choice comes at increased implementation cost in terms of graph design, booklet printing and administration. A recommended compromise between these issues is a two arm RCT.

- **Printed booklets**

The literature has demonstrated many studies using controlled laboratory conditions with computer equipment displaying sequences of graphs to subjects (see Meyer *et al.* 1997, Wickens *et al.* 1994 and Hollands 1992 for examples). These conditions allowed the investigators to ensure the display and the display conditions were identical. Importantly it also allowed the investigators to show subjects a series of graphs, each showing a change to a single element within the graph. Therefore, differences in subject response could be associated with changes in specific graph elements. However, subjects performing tasks using computer-generated graphs is not a practical component for the current study's protocol, which will involve the random recruitment of subjects from around the State of NSW. The logistical problem of ensuring consistency of electronic displays used by all subjects and the associated cost prohibit the use of this technique. However, laboratory controlled, computer based trials were not always used. In particular, studies were found that presented subjects with graphs in printed booklets (see Sparrow 1989 and Hollands and Spence 1992). The pragmatic advantages of this presentation are that (i) the investigators control the displays seen by all subjects, (ii) booklets are cost effective to produce (iii) booklets can be easily and cost effectively distributed to subjects around the State and (iv) subjects who lose or misplace booklets can have replacements sent within a reasonable time-frame. The disadvantages of this system are (i) limitation to the number of adjustments that can be made to individual graphs and (ii) a booklet printed in monochrome cannot test the impact of colour in graphs.

4.9.2 Recommendations for subjects and subject selection

- **The members of the defined population should be identified in a sample frame and randomly selected for inclusion in the study.**
- **Once selected, subjects must be followed-up with appropriate reminders to maximise the response rate.**

Many studies used subjects selected from university (see Meyer *et al.* 1997, Sparrow 1989, Shah *et al.* 1999 for examples) with small sample sizes. The studies using larger sample sizes included Henry's (1993) trial that sampled from five defined populations. The reporting of a response rate in this study identified a concern implicit with population studies: non-response bias. To minimise this bias, protocols should be in place to encourage all subjects to complete the questionnaire.

- **Subjects must not be visually impaired and must be able to understand sufficient English to complete the questionnaire.**

A common requirement of studies in the review was that subjects had standardised vision (see Carswell 1991 and Carswell *et al.* 1991 for examples). Implicit in the studies was that subjects had a sufficient understanding of English to complete the trial.

- **Subjects should be instructed to answer the questions from the information in each graph, not from their own experience of the topic.**

Although some studies selected subjects based on the level of prior knowledge of graphs Cleveland and McGill (1984) found no statistically significant differences between subjects differentiated by their experience using graphs. However, in terms of the topic contained in each graph, there were differences in approach. Some (see Carpenter and Shah's 1998 study) used a topic for graph presentation that was not common knowledge. Others (see Culberston and Powers 1959) used a topic that was familiar to subjects with the explicit instruction that subjects were to answer the questions from the information in each graph, not their own knowledge.

- **The instructions to subjects completing the questionnaire must be clear and concise; convey a sense of the trial's importance and encourage participation. A cover letter from the appropriate NSW Health official emphasising the later points would be a benefit to the study.**

A recommendation to convey the study's importance to subjects, the need for participation and concentration during the trial was specifically stated by Cleveland and McGill (1987). While the laboratory-controlled experiments in this review often conducted practice sessions to prepare subjects for the evaluated trial, this is not a practical option for a broader population based survey. However, clear instructions and a motivational letter from NSW Health should provide a satisfactory alternative.

- **Complexity should be controlled in the graph displays for possible use during analysis. A suitable definition should be used to allocate graphs into low or high complexity categories.**

Complexity was not often controlled in the studies in this review. However, the advantage of categorising graphs according to their complexity is that there may be benefits during analysis. Examples of definitions of complexity can be found in section 4.5.

4.9.3 Recommendations for questionnaire design

- **The measurement for evaluation should be accuracy preferably measured using closed-ended questions.**

The two common measures of comprehension were time to complete a task and accuracy of the answer. However, Kruskal (1982) specifically warned against using time as a measure of comprehension because, he stated, in real situations there is not a specific time constraint applying to the graph reader. Although the use of open-ended questions is a possibility for the questionnaire, suitable coding frames and crosschecking would also need to be developed in association. This raises issues of time and cost constraints for data entry and analysis.

- Question development should be made in the context of local and global components of the display graph. Specifically, questions should move from those dealing with local frameworks (reading an exact numerical value and comparing specific points) to global frameworks (trend reading and trend comparison).

The local and global frameworks formed a common theme for developing questions to test reader comprehension in the publications (see Leinhardt *et al.* 1990, Wickens *et al.* 1994, Casali and Gaylin 1988, Sparrow 1989, Gillespie 1993, Henry 1993 and Guthrie *et al.* 1993). Section 4.6 has specific examples of the local / global questions. The advantage of using this framework as the basis for questionnaire design, apart from its frequent use in other studies, is the guidance it provides for specific question wording.

Bibliography

Bertin J 1980, The basic test of the graph: a matrix theory of graph construction and cartography, PA Kolers, ME Wrolstad & H Bouma (eds), *Processing of Visible Language 2*, New York: Plenum Press.

Carpenter PA, Shah P 1998, A Model of the Perceptual and Conceptual Processes in Graph Comprehension, *Journal of Experimental Psychology Applied*, 1998 Vol 4 No. 2 75-100.

Carswell CM 1991, Boutique data graphics: perspectives on using depth to embellish data displays, *Proceedings of the Human Factors Society 35th Annual Meeting* (1532-1536) Human Factors and Ergonomics Society: Santa Monica CA.

Carswell CM, Frankenberger S & Bernhard D 1991, Graphing in depth: perspectives on the use of three-dimensional graphs to represent lower dimensional data, *Behaviour and Information Technology* 10: 459-474.

Carswell CM & Ramzy C 1997, Graphing small data sets: should we bother?, *Behaviour and Information Technology* 16: 61-71.

Casali J, Gaylin KB 1988, Selected graph design variables in four interpretation tasks: a microcomputer-based pilot study, *Behaviour and Information Technology,* 7 No 1, 31-49

Cleveland WS & McGill R 1984, Graphical perception: theory, experimentation and application to the development of graphical methods, *Journal of American Statistical Association* 79 September: 531-554.

Cleveland WS & McGill R 1987, Graphical Perception: The Visual Decoding of Quantitative Information on Graphical Displays of Data, *Journal of the Royal Statistical Society* 150(Part 3): 192-229.

Cleveland WS 1993, A Model for studying Display Methods of Statistical Graphics, **Journal of Computational and Statistical Graphics** 3: 1-21.

Culbertson HM, Powers DR 1959, A study of graph comprehension difficulties, *Audio-Visual Communication Review* 7: 97-100.

Eggan P, Kauchak D, Kirk S 1978, The effects of generalisations as cues on the learning of information from graphs, *Journal of Educational Research* 71(4): 211-213.

Ferry B, Hedberg J, Harper B 1999, *Developing computer-based cognitive tools that assist learners to interpret graphs and tables*, University of Wollongong, obtained from web site. http://www.swin.edu.au/aare/99pap/fer99092.htm

Gillan DJ, Lewis R 1994, A componential model of human interaction with graphs:: I linear regression modelling, *Human Factors* 36(3): 419-440.

Gillespie CS 1993, Reading graphic displays: what teachers should know**,** *Journal of Reading* February 36(5): 350-354

Greaney J, MacRae AW 1997, Visual search and the detection of abnormal readings in graphical displays, *ACTA Psychologica* 95 165-179.

Guthrie JT, Weber S, Kimmerly N 1993, Searching Documents: Cognitive Processes and Deficits in Understanding Graphs, Tables and Illustrations, *Contemporary Educational Psychology* 18, 186-22.1

Henry GT 1993, Using graphical displays for evaluation, *Evaluation Review* 17(1): 60-78.

Hollands JG 1992, Alignment, Scaling, and Size Effects In Discrimination of Graphical Elements, *Proceedings of Human Factors Society 36th Annual Meeting.*

Hollands JG and Spence I 1992, Judgements of Change and Proportion in Graphical Perception, *Human Factors* 34(3), 313-334.

Kruskal WH 1982, Criteria for judging statistical graphs, *Utilitas Mathematica* 21B: 283-310.

Lee ML and MacLachlan J 1986, The Effects of 3D Imagery on Managerial Data Interpretation, *MIS Quarterly*, September, 257-268.

Leinhardt G, Zaslavsky O & Stein MK 1990, Functions, graphs and graphing: tasks, learning and teaching, *Review of Educational Research* 60: 1-64.

Meyer J, Shinar D & Leiser D 1997, Multiple Factors that Determine Performance with Tables and Graphs, *Human Factors* 39(2): 268.

Meyer J, & Shinar D 1992, Estimating Correlations from Scatterplots, *Human Factors* 34(3), 335-349.

Poulton EC 1985, Geometric illusions in reading graphs, *Perception and Psychophysics* 37, 543-548.

Ritter D and Coleman SL 1995, Assessing the Graphing Skills of Pre-Service Elementary Teachers, *JCST Research and Teaching*, May, 388-391.

Shah P, Carpenter PA 1998, Conceptual Limitations in Comprehending Line Graphs, *Journal of Experimental Psychology* March 124(1): 43.

Shah P, Mayer RE & Hegarty M 1999, Graphs as aids to knowledge construction: signalling techniques for guiding the process of graph comprehension, *Journal of Educational Psychology* December 91(4): 690.

Schnotz W, Picard E, Hron A 1993, How do successful and unsuccessful learners use text and graphics? *Learning and Instruction* 3: 181-199.

Schutz HG 1961, An Evaluation of Methods for Presentation of Graphic Multiple Trends, *Human Factors* 3: 108-119.

Simkin D & Hastie R 1987, An information-processing analysis of graph perception, *Journal of the American Statistical Association* 82: 454-465.

Sparrow JA 1989, Graphical displays in information systems: some properties influencing the effectiveness of alternate forms, *Behaviour and Information Technology* 8: 43-56.

Wickens CD, Merwin DH & Lin EL 1994, Implications of Graphics Enhancement for the Visualisation of Scientific Data: Dimensional Integrity, Stereopsis, Motion and Mesh, *Human Factors* 36: 44-61.

Appendix 1
A graph classification system

Characteristic	Comment
Measures	Seven standard measures were used for classification criteria: frequency,<br>rates, proportions, central tendency (means and medians), ratios, life expectancy and risk.
Aim of the graph	Expresses the aim intended by the author. Cleveland (1994:p221) says: "When a graph is constructed, information is encoded. The visual decoding of his encoded information is graphical perception. The decoding is the vital link, the raison d´etre." In a similar vein Kosslyn (1994: p 271) states "a good graph forces the reader to see the information the designer wanted to convey"<br>As the author's intent when encoding the graph cannot always be determined, the 'likely' aims are identified in this document. The aim is therefore expressed as a function of the variables that are used in the graph. An acknowledgement is made of the subjectivity used to identify what these aims might be.
Type of graph used	Identifies:<br>• the primary type of graph: eg bar, histogram, line, area, pie etc<br>• the sub-group type of graph (if applicable): eg exploded pie, stacked bar graph, vertical bar, horizontal bar, three dimensional.
Definition of graph style	A definition of the graph 'type' identified in the previous point. The definition contains the standard characteristics and uses of the graph 'type'.
Frequency of graph usage	Three levels of usage in the reviewed publications were identified:<br>• low: Less than 30 examples in the reviewed publications<br>• medium: 31 to 100 examples in the reviewed publications<br>• high: 101 or more examples in the reviewed publications.
Outcome variables	Also known as the dependent or response variable. This is the variable plotted on the graph that may change in response to changes in the independent variables described below. The aim of the graph is usually to visually demonstrate a relationship between the independent variable and the outcome variable. For example, a graph of blood pressure against salt intake would label 'blood pressure' as the outcome or dependent variable and 'salt intake' would be the independent variable. For a graph showing the count of disease notifications over time, the count of notifications is the outcome variable. The outcome variable could be discrete, continuous, categorical, ranked, binomial, multinomial etc.
Independent variables	Also known as covariate, explanatory or predictor variable. These are variables displayed in the graph and related to the outcome variable. For instance, using the example of blood pressure against salt intake, salt intake would be the independent variable. The independent variable could be discrete, continuous, categorical, ranked, binomial, multinomial etc.
Statistical concepts	These are the statistical concepts communicated by the graph to the reader. For example, is the graph showing a simple count of an occurrence at one point in time or a trend occurring over multiple time-periods? The statistical concepts include crude incidence rates, age standardised incidence rates, prevalence rates, counts, variance, confidence intervals, statistical significance, trends, seasonal variations, temporal variations, etc.

Characteristic	Comment
Type of comparison	This identifies the type of comparisons that can be made with the graph and was identified by the answer to the question: "What is communicated?". Or otherwise stated: "What types of questions can be answered using the graph?".<br>Graphs with one outcome variable and one independent variable can answer one question: "What is the relationship between the outcome and independent variable?". Graphs with one outcome and two independent variables can answer three questions: "What is the specific relationship between the outcome variable and each of the two independent variables?" (2 questions) and: "What is the overall relationship between the outcome variable and the two independent variables?" (one question).
Colour and shading of series line / bar etc.	The colours or patterns used to define each data series on the graph.
Titles	The level of description provided in the title:<br>• whether it provides an adequate definition of the graph<br>• whether the title stands alone or needs to be seen in context.<br>The titles were categorised according to:<br>• the topic of the graph (eg asthma)<br>• an aspect of that topic (eg prevalence of wheezing in last twelve months)<br>• population type (eg school aged children)<br>• the age group (eg 5 to 9 years)<br>• the independent variable (eg indigenous status and sex)<br>• the place (eg Queensland)<br>• the reference period (eg 1998)
Data series legend or data series titles	Identifies:<br>• the use of legends to identify the outcome and independent variable(s) or<br>• the use of series titles, that is, labels located on or point to the data series<br>• whether the legend or title stands alone or need to be viewed in context.
Font types and font size	The fonts applying to titles, labels, axis titles and other graph notation.<br>The font **style** was sub-classified as:<br>• a serif font or<br>• a sans serif font.<br>The font **size** was sub-classified as:<br>• small: that is, smaller than the font size used in the document's text<br>• medium: that is, the same size as used in the document's text<br>• large: that is, larger than the font size used in the document's text.
Scales, gridlines and tick marks	Identifies the use of scales, that is, whether logarithmic scales have been used, whether the axis has been scaled from a neutral point (eg zero) etc. The use of gridlines and tick marks is also noted.
Line thickness	The line thickness on the border of bar, pie and area graphs or the line used in a line graph.
Comment on text interpretations	Identifies:<br>• whether the text associated with the graph explains to the reader how to interpret the graph<br>• whether a prior knowledge of the graph is assumed<br>• whether tables have been used which assist the interpretation of the graph.

Characteristic	Comment
Sources	Identifies the Australian health publications where examples of the graph were found.
Abbreviations	Identifies whether the graph uses abbreviations.
Consistency between and within health publications	Identifies whether the same graph style has been used consistently between health publications and within the same publication. Similarities and differences are highlighted.
Other notes	General notes and a limited evaluation of the graphs.

SHPN (HSP) 060049
