# Supplementary material for: Communicating population health statistics through graphs: a randomised controlled trial of graph design interventions
Source: BMC Med. 2006 Dec 20;4:33. doi: 10.1186/1741-7015-4-33 (PMC1766925; doi:10.1186/1741-7015-4-33)
Supplement: Additional file 2 — Full report of the experimental study. Contains the published report with complete results and appendices showing the control and intervention booklets and questionnaire [file 1741-7015-4-33-S2.pdf]

NSW HEALTH

Better health graphs

Volume 1

A report of an experimental study of interventions for improving graph comprehension

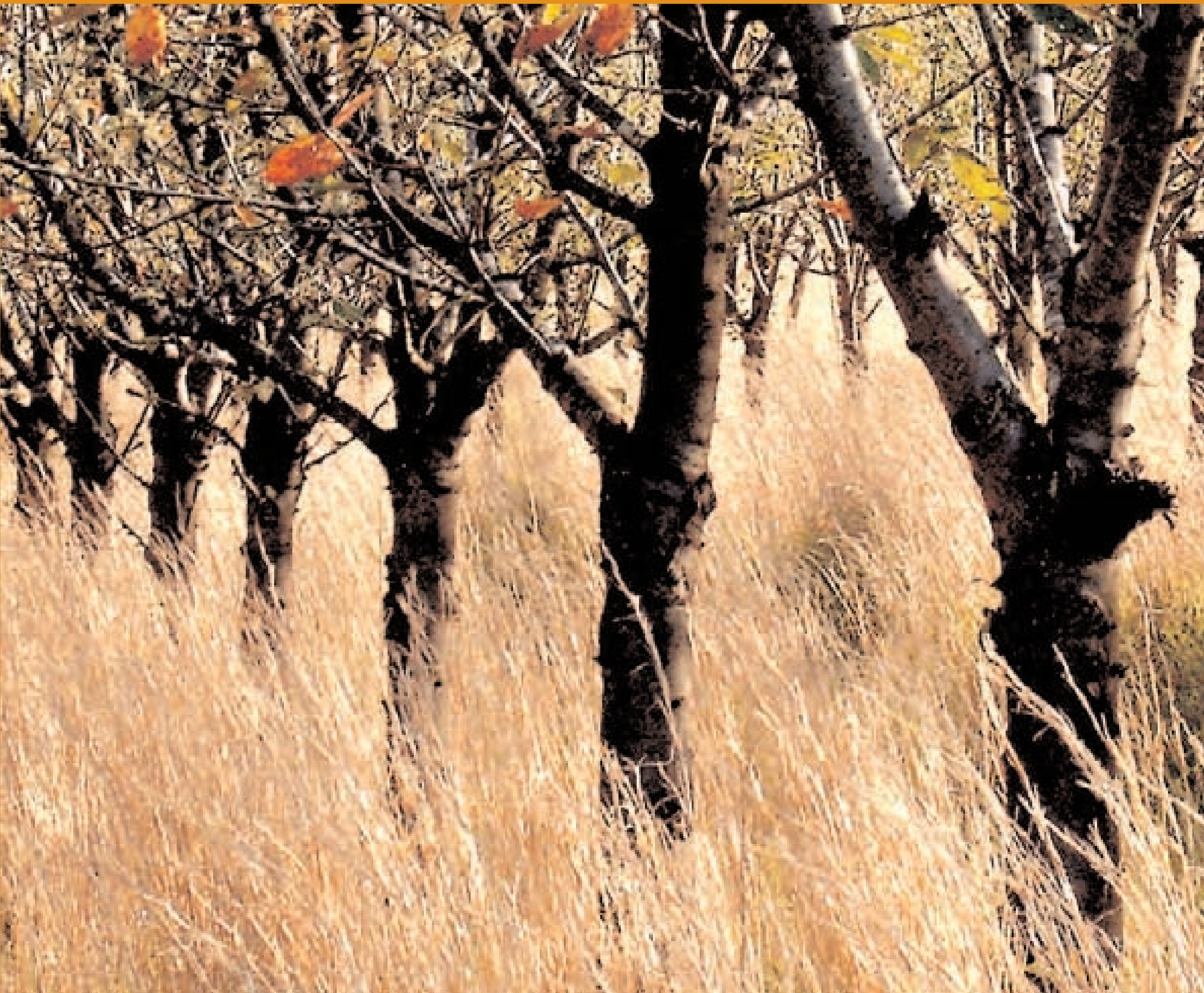

Copyright © NSW Department of Health, July 2006

This work is copyright. It may be reproduced in whole or in part, subject to the inclusion of an acknowledgment of the source and no commercial usage or sale.

State Health Publication No: HSP 060048
ISBN 0 7347 3922 2

suggested citation:
Centre for Epidemiology and Research and Hunter Valley Research Foundation, *Better Health Graphs (Volume 1): A report of an experimental study of interventions for improving graph comprehension*. Sydney: NSW Department of Health, 2006.

produced by:
Centre for Epidemiology and Research
Population Health Division
NSW Department of Health
Locked Mail Bag 961
North Sydney NSW 2059 Australia
Tel: 61 2 9391 9408
Fax: 61 2 9391 9232

further copies of this publication can be obtained
from the NSW Department of Health website at:
www.health.nsw.gov.au

Contents

Acknowledgments..3

Executive summary.......................................5

Introduction..7

Methods 8

Results..10

Discussion..18

Recommendations 21

Conclusion 23

References 24

Appendix 1.
The control booklet of graphs

Appendix 2.
The intervention booklet of graphs

Appendix 3.
The questionnaire

Acknowledgments

This project was jointly funded by the Commonwealth Department of Health and Ageing and the Centre for Epidemiology and Research, NSW Department of Health. The project was conducted under the National Population Health Information Development Plan (Australian Institute of Health and Welfare, 1999). The Hunter Valley Research Foundation conducted the project under contract to the NSW Department of Health. These final reports represent the collaborative work of the Hunter Valley Research Foundation and staff from the Centre for Epidemiology and Research, in particular:

The Hunter Valley Research Foundation

- Mr Andrew Searles
- Ms Robin Mcdonald

Centre for Epidemiology and Research, NSW Department of Health

- Mr David Muscatello

The Centre for Epidemiology and Research would like to acknowledge the valuable assistance of the additional members of the project Steering Committee:

- Dr Tim Churches, Centre for Epidemiology and Research
- Dr Paul Jelfs, Australian Institute of Health and Welfare
- Dr Louisa Jorm, Centre for Epidemiology and Research
- Ms Jill Kaldor, Centre for Epidemiology and Research

Acknowledgment of graphs reproduced for the survey

The following documents were used to provide example graphs used as control graphs in the study. Permission to reproduce the graphs is gratefully acknowledged.

Illustration A
Australian Institute of Health and Welfare (AIHW) 2000, Australasian Association of Cancer Registries, *Cancer in Australia 1997, Incidence and mortality data for 1997 and selected data for 1998 and 1999*, AIHW Cat. no. CAN 10, Canberra, reproduced with permission from the Australian Institute of Health and Welfare.

Illustrations B and C
Department of Human Services 1999, *Victorian burden of disease study: Morbidity*, Public Health Division, Department of Human Services, Melbourne, reproduced with permission from the Victorian Department of Human Services.

Illustration D
Department of Human Services 2000, *Victorian burden of disease study: Mortality*, Public Health Division, Department of Human Services, Melbourne, reproduced with permission from the Victorian Department of Human Services.

Illustration E
Queensland Health 2001, *Health indicators for Queensland: Central Zone 2001*, Public Health Services, Queensland Health, Brisbane, reproduced with permission from the State of Queensland (Queensland Health).

Illustration F
NSW Health 2000, *The health of the people of NSW – Report of the Chief Health Officer 2000*, NSW Health Department, Sydney, reproduced with permission from the NSW Department of Health.

Illustration G
Condon JR, Warman G, Arnold L (editors) 2001, *The health and welfare of Territorians*, Epidemiology Branch, Territory Health Services, Darwin 2001, reproduced with permission from Northern Territory Department of Health and Community Services.

Illustration H
Ridolfo B, Sereafino S, Somerford P and Codde J, *Health measures for the population of Western Australia: Trends and comparisons*, Health Department of Western Australia, Perth 2000, reproduced with permission from the Health Department of Western Australia.

Illustration I
NSW Health 2000, *The health of the people of NSW – Report of the Chief Health Officer 2000*, NSW Health Department, Sydney, reproduced with permission from the NSW Department of Health.

Illustration J
Kee C, Johanson G, White U, McConnell J 1998, *Health indicators in the ACT*, Epidemiology Unit, ACT Dept of Health and Community Care: Health series No. 13, ACT Government Printer, ACT, reproduced with permission from the ACT Department of Health and Community Care.

Illustration K
Australian Bureau of Statistics (ABS) and the Australian Institute of Health and Welfare (AIHW) 2001, *The health and welfare of Australia's Aboriginal and Torres Strait Islander peoples*, ABS Cat. no. 4704.0, AIHW Cat. no. IHW 6, Canberra 2001 (www.abs.gov.au), reproduced with permission from the Australian Bureau of Statistics and the Australian Institute of Health and Welfare.

Illustration L
Coats MS, Tracey EA, *Cancer in NSW: Incidence and mortality 1999 featuring 30 years of cancer registration*, Cancer Council NSW, Sydney 2001, reproduced with permission from the NSW Department of Health.

Executive summary

Introduction

This project aimed to recommend ways to improve the graphical communication of population health statistics to a broad audience.

It was conceived to explore the hypothesis that much of the statistical information presented in graphical form in official population health publications is poorly understood by people who are not trained in public health, epidemiology or statistics.

The Centre for Epidemiology and Research (CER) of the New South Wales (NSW) Department of Health, Australia, was the lead agency in the project. It was developed under the National Publication Health Information Development Plan,[1] and was co-funded by the Australian Government Department of Health and Ageing and CER's Program for Enhanced Population Health Infostructure (PEPHI). CER contracted the project to the Hunter Valley Research Foundation (HVRF) – a not-for-profit research institution based in Newcastle, New South Wales, Australia. A working group that consisted of representatives from HVRF, CER and the Australian Institute of Health and Welfare supported the project.

The project had two parts: a literature review and an experimental study. The literature review examined available evidence regarding graph readability. It is available as Volume 2 of this report at www.health.nsw.gov.au.

Methods

The experimental study is reported here. It was a double-blind, randomised, controlled trial that tested a variety of changes to the design of existing graphs. The population studied included staff members of the NSW public sector health system, regardless of employment type. Respondents were randomly assigned to receive either a 'control' or 'intervention' booklet of 12 graphs, and an identical questionnaire asking 39 questions relating to the interpretation of the graphs. The 'control' graphs were replicas of graphs used in Australian population health publications. The 'intervention' graphs included one or two changes to the control graphs that were hypothesised to improve comprehension of the graph. Questions were targeted to specific changes, where possible. The success of the intervention was measured as a prevalence ratio of the proportion of correct answers in the two groups.

Results

The overall response rate was 67%. Demographic characteristics were similar between the control and intervention groups, although the intervention group were more likely to rank themselves as more frequent graph users and as having good visual ability.

For the control graphs, the proportion of subjects responding correctly to the 39 interpretation questions ranged from 13% to 97%. Questions requiring an understanding of confidence intervals (32%) and age standardisation (37%) had poor comprehension rates. (Table 2). There were seven tasks with comprehension rates of at least 90%.

In terms of the effect of the interventions, the tasks which benefited most from an intervention were: changing a pie chart to a bar graph and point reading the magnitude of a single category (prevalence ratio 3.6, 95% CI 2.8-4.6); changing the y axis of a graph so that the upward direction represented an increase instead of a decrease in the plotted quantity and judging the direction of a trend (2.9, 95% CI 2.1-9.9); including a footnote to explain an acronym and performing a task that requires knowledge of the meaning of the acronym (2.5, 95% CI 1.6-3.8); and making the axis range of two adjacent graphs match and comparing the size of a difference between the two series shown on each graph (2.0, 95% CI 1.7-2.4). Only one intervention had a clear negative impact.

Success at comprehending the control graphs was generally lower in subjects without university qualifications, although an exception was the pie chart, where twice as many non university-educated as university-educated control subjects correctly estimated the magnitude of a category within a pie chart. For subjects without a university education, the generally lower success for the control charts was complemented by a generally greater difference in the prevalence of

correct answers between the intervention and control groups, although there were no statistically significant differences in prevalence ratios between the two education groups.

Discussion

Our findings are of benefit from two perspectives. First, we were able to quantify the proportion of readers who could extract some typical statistical interpretations from a sample of graphs used in Australian official health publications. Second, we were able to measure the benefits associated with particular interventions in a broad sample of readers.

The most dramatic result of the study related to a graph showing that Aboriginal people in a region of Australia had an increased risk of mortality compared with the general Australian population. A combination of interventions that included a simple title and explanatory words, rather than numbers, on the vertical axis more than halved the proportion of subjects who did not grasp that Aboriginal people had a higher mortality risk.

Less than 60% of subjects could answer a question that required an understanding that disease incidence refers to the rate of *new cases* of disease in a period of time. Using a non-technical label for incidence had a statistically significant benefit.

Two statistical techniques and concepts occur frequently in population health graphs: age standardisation and confidence intervals. Respondents found it difficult to understand these concepts. Simple explanatory footnotes offered improvements of up to 2.5-fold, but there remained a large proportion who were unable to make the required interpretations.

We tried two interventions aimed at reducing the volume of information to be interpreted. Reducing the number of layers in a stacked layer graph did not offer a benefit. Removing an independent (categorisation) variable from a vertical bar graph raised the comprehension rate by 20% for one task.

A line graph and a grouped vertical bar graph of multiple disease trends by year performed equally well for point-reading tasks, but the line graph produced a marginal improvement in trend judgement in subjects without a university education. Among university-educated subjects, a 'population pyramid' represented as a horizontal format line graph improved broad comparison of the shape of the population distribution by sex.

Bar graphs out-performed dot graphs, particularly among those without university qualifications. A stacked layer graph worked well for some tasks requiring interpretation of trend and broad comparisons, but worked poorly for a task requiring the estimation of a difference between the absolute rate at two points along a layer.

For simple quantitative tasks such as identifying minimum and maximum categories or making comparisons where the differences were distinct, a pie chart performed as well as a bar chart. It performed poorly for point readings of the displayed quantity, but this deficiency could potentially be overcome by labelling the relevant quantity on each pie segment.

For tasks comparing the relative magnitude of quantities between two adjacent graphs, a matching scale range on each graph greatly improved comprehension.

We found strong evidence for ensuring that higher values of the quantity presented on the graph be in the upward direction, even if this means the numerical labels are decreasing in the upward direction.

Recommendations

- Use plain, non-technical language in the graph title and graph components
- Use the minimum number of sub-categories (independent variables) necessary
- Use conventional line or bar graphs where possible
- Recognise that the interpretation of confidence intervals and age standardisation requires technical knowledge
- Assist readers to interpret ratios
- Ensure that quantities (not labels) increase from the bottom to the top or left to right
- If using pie charts, label the quantity represented by each segment on or near the segment
- If presenting graphs in pairs, ensure the axes have the same ranges
- Use line graphs to represent trend information rather than bar graphs

Introduction

This project aimed to recommend ways to improve the graphical communication of population health statistics to a broad audience.

It was conceived to explore the hypothesis that much of the statistical information presented in graphical form in official population health publications is poorly understood by people who are not trained in public health, epidemiology or statistics.

The Centre for Epidemiology and Research (CER) of the New South Wales (NSW) Department of Health, Australia, was the lead agency in the project. It was developed under the National Publication Health Information Development Plan,[1] and was co-funded by the Australian Government Department of Health and Ageing and CER's Program for Enhanced Population Health Infostructure (PEPHI).

The Centre for Epidemiology and Research contracted the project to the Hunter Valley Research Foundation (HVRF) – a not-for-profit research institution based in Newcastle, New South Wales, Australia. A working group that consisted of representatives from HVRF, CER and the Australian Institute of Health and Welfare supported the project.

The project had two parts: a literature review and an experimental study. The literature review examined available evidence regarding graph readability. It is available as a Volume 2 of this report at www.health.nsw.gov.au.

This report summarises the findings of the experimental study and presents its major recommendations for improving the graphical presentation of population health statistics.

Methods

Study design

The study was designed as a double-blind, randomised, controlled trial, with data collected through a self-completed questionnaire. Subjects were randomly assigned to receive either a 'control' or an 'intervention' booklet of graphs. Both groups received an identical questionnaire that explored subjects' understanding of the meaning of the graphs.

Study subjects were blinded to their control or intervention status. Study personnel and researchers were blinded to the status of respondents until after data analysis occurred. Each respondent group was assigned an arbitrary group identifier that did not reveal their status, even while analysis of the results was being undertaken. Data entry personnel were blinded to the respondent status, as they were not shown the graph booklet that was returned with the questionnaire. The status of each group was only revealed after analysis was complete.

Control and intervention graphs

The 'control' booklet (Appendix 1) contained 12 examples of graphs that were reproduced from their original publication. Graphs were chosen to represent the kind of information commonly presented in Australian national and State population health publications. They covered a range of different graph styles and numeric measures, including population size, disease incidence rates, disease prevalence, incidence rate ratios, and risk of developing disease. Statistical concepts, such as age standardisation and confidence intervals, were presented in some graphs.

Graphs for the 'intervention' booklet (Appendix 2) presented the same statistical information as those in the control booklet, but were subject to one or more changes. The changes were chosen in an effort to improve comprehension of the statistical information depicted by the graph. They were selected on the basis of findings from the literature review for which evidence was limited, after considering the nature of graphs used in population health publications. To limit the number of graphs and thus respondent workload, more than one change was made to some graphs. In some cases, these changes were collectively intended to improve understanding, while in others, they were chosen to be as independent as possible.

The details of each intervention are described in Table 2, along with reduced scale images of both versions of the 12 graphs studied.

Questionnaire

The questionnaire (Appendix 3) contained several questions relating to each of the graphs, 39 questions in total. The questions were designed to assess how well subjects understood the information presented in the graphs, and to specifically assess the impact of each of the changes made for the intervention booklet.

The questionnaire also collected demographic details, as follows: education level, preferred language, age group, and sex. Respondents were also asked how frequently they used graphs, their work title, and to rate their visual ability to read the graphs presented.

Study sample

The study population included all employees of the NSW public sector health system, regardless of the nature of their work. This population was chosen for the following reasons:

- it was anticipated that there would be a poor response rate from the general public
- there was a readily available sampling frame of public sector health employees
- public sector health employees are an important audience for population health statistics

The sampling frame included those employees whose contact details were listed on one of five telephone directory databases, that listed employees of the main NSW Department of Health administration, an urban regional Area Health Service (AHS), a mixed urban/rural AHS, and two rural AHSs. Six hundred and fifty subjects were randomly selected from the combined directories. The directories were not restricted by occupation and

included medical, allied health, managerial, clerical, policy, maintenance, and other occupations. Those people who no longer worked at the position indicated in the database were excluded.

The 650 subjects were then divided randomly into two groups of 325; the intervention and control groups. Each subject was posted a package containing a cover letter signed by the NSW Chief Health Officer inviting their participation, a questionnaire booklet, a control or intervention graph booklet, and a reply-paid envelope. Up to six follow-up reminder calls were made to non-responders. These calls also allowed ineligible subjects to be identified. Ineligible subjects were those who no longer worked for the health service or who were unknown at the available contact address.

Analysis

Unanswered questions were treated as incorrectly answered. A prevalence of correct answers to an interpretation task, that is, a ‘comprehension rate’, was calculated in the control and intervention groups. The effect of the interventions on each task was assessed by calculating the prevalence ratio of the comprehension rate in the intervention and control groups with a 95% confidence interval (CI). Analysis was conducted using SPSS version 10.

Results

Response rate and study sample

Of the 650 subjects selected, 543 were eligible, and of these, 366 returned completed questionnaires, giving an overall response rate of 67% (intervention group 67%, control group 66%).

Sex, age, preferred language, education and work position were similarly distributed between the control and intervention arms of the study. Intervention subjects were somewhat more likely to rate themselves as frequent graph users than control subjects and were more likely to rate themselves as having good visual ability (Table 1).

Comprehension of the unaltered (control) graphs

Of the 39 interpretation tasks for the 12 graphs, the proportion of subjects responding correctly ranged from 13% for a task requiring specific knowledge of an acronym, to 97% for a task identifying the largest category in a pie chart. Other tasks with a poor comprehension rate included judging the direction of a trend in a line graph in which the y axis represented an increasing quantity in the downward direction (21% answered correctly), and estimating a point reading of a quantity from a pie chart (26%). Questions requiring an understanding of confidence intervals (32%) and age standardisation (37%) also had poor comprehension rates (Table 2).

There were seven tasks with comprehension rates of at least 90%. These included: choosing the largest (97%) and smallest (91%) categories and comparing the magnitude of two categories (95%) from a pie chart; determining the largest category from a dot graph (94%); choosing the category with the lowest proportion at a single point along the x axis in a grouped vertical bar graph (94%); and broad judgements of the collective relative magnitude by sex and rurality of bars on a vertical bar graph with bars subdivided first by sex and then by rurality (93% for sex and 90% for rurality) (Table 2).

Effect of interventions

In terms of the prevalence ratio of correct answers between the control and intervention groups, the tasks which benefited most from an intervention were: changing a pie chart to a bar graph and point reading the magnitude of a single category (prevalence ratio 3.6, 95% CI 2.8-4.6); changing the y axis of a graph so that the upward direction represented an increase instead of decrease in the plotted quantity and judging the direction of a trend (2.9, 95% CI 2.1-9.9); including a footnote to explain an acronym and perform a task that requires knowledge of the meaning of the acronym (2.5, 95% CI 1.6-3.8); and making the y axis range of two adjacent graphs match and comparing the size of a difference between the two series shown on each graph (2.0, 95% CI 1.7-2.4) (Table 2).

The only intervention that had a clear negative impact was a combination of reducing the number of layers on a stacked layer graph and inserting a footnote explaining the meaning of a layer's thickness. For a task of judging the direction of trend in one layer, the prevalence ratio was 0.8 (95% CI 0.7-0.9) (Table 2).

Influence of education

Success at comprehending the control graphs was generally lower in subjects without university qualifications. The largest differences were for the following tasks: judging the statistical significance of the difference between two categories using confidence intervals (16% of non-university educated controls versus 40% of university-educated controls); understanding the influence of age standardisation on graph interpretation (23% versus 44%); and judging the relative magnitude of risk between two series on a graph when the upward direction on the y axis represents reducing risk (32% versus 58%).

An exception was the pie chart, where twice as many non university-educated as university-educated control subjects correctly estimated the magnitude of a category within a pie chart (40% versus 19%).

For subjects without a university education, the generally lower success for the control charts was complemented by a generally greater impact of the interventions, although there were no statistically significant differences in prevalence ratios between the two education groups. The greatest differences were for the interventions applied to the dot graph with confidence intervals ("hi-lo-close" graph), which was changed to a horizontal bar graph with confidence intervals and a footnote was included for interpreting the confidence intervals. The prevalence ratio for correctly interpreting the statistical significance of the difference between two categories on the graph was 2.5 (95% CI 1.3-4.9) for subjects without a university education compared with 1.6 (95% CI 1.2-2.0) for subjects with a university education. For interpreting whether a category was higher or lower than a reference line representing the average of all categories on this graph, the prevalence ratio was 2.3 (95% CI 1.6-3.3) for those without a university education and 1.4 (95% CI 1.2-1.7) for those with a university education.

Among university educated subjects, there was a marginal reduction in the comprehension rate for one task using a graph with a dual intervention. The interventions were: changing a horizontal divided bar graph with two bars for each sex to a side-by-side divided bar graph with the sides representing each sex; and including a footnote explaining acronyms used in the graph. The task involved comparing the relative magnitude of the two segments within a single bar in both the control and intervention graph (prevalence ratio 0.9, 95% CI 0.8-1.0). The latter task did not require an understanding of the acronyms, but the bar segments represented the quantities labelled by the acronyms, so the extra reading introduced by the footnote may have added complexity or confusion for some readers.

Table 1. Sample characteristics

		Intervention group		Control group		
**Characteristic**	**Category**	**Number (N=176)**	**Per cent**	**Number (N=187)**	**Per cent**	**p-value**
Sex	Male	53	30.1%	47	25.1%	0.26
Age	Under 34 years	37	21.0%	41	21.9%	0.54
	35-54 years	109	61.9%	106	56.7%	
	55 years and over	27	15.3%	36	19.3%	
Preferred Language	English	171	97.2%	183	97.9%	0.53
Education	University qualification	116	65.9%	124	66.3%	0.83
Work position*	Clinical	61	34.7%	76	40.6%	0.53
	Public health/policy	36	20.5%	35	18.7%	
	Other	72	40.9%	70	37.4%	
Frequency of graph use	Often	55	31.3%	44	23.5%	
	Occasionally or never	118	67.0%	141	75.4%	0.09
Self-rated visual ability	Good	122	69.3%	110	58.8%	
	Average or poor	48	27.3%	74	39.6%	0.02

*Work position: Clinical=doctors, nurses, allied health dealing with patients; non-clinical public health/policy=health-related but not dealing directly with patients; other=non-health admin, computing, clerical, maintenance etc.

Category totals may not add to 100% because of missing responses

Table 2. Comparison of the proportion of correct answers between the intervention ("Int.") and control ("Con.") groups for each intervention tested, and by highest level of education attained.

			All respondents			Non university-qualified			University-qualified		
**Graph description**	**Intervention(s)**	**Interpretation task**	**Int. % (N=176)**	**Con. % (N=187)**	**Ratio (95% C.I.)**	**Int. % (N=56)**	**Con. % (N=57)**	**Ratio (95% C.I.)**	**Int. % (N=116)**	**Con. % (N=124)**	**Ratio (95% C.I.)**
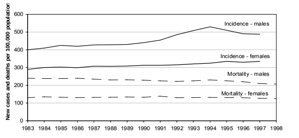 Source: Cancer in Australia 1997, AIHW & AACR 2000.<br>Illustration A: Trends in age-standardised incidence and mortality rates for all cancers (excluding non-melanocytic skin cancers), Australia, 1983-1998	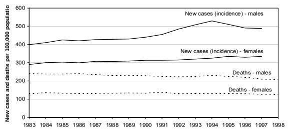 Note: age-standardised rates allow comparisons over years and between males and females. Different age-standardised rates are not due to differences in the relative proportions of older or younger people in each year or sex.<br>Source: Cancer in Australia 1997. AIHW & AACR 2000.<br>Illustration A: Trends in age-standardised incidence and death rates for all cancers (excluding non-melanocytic skin cancers), Australia, 1983-1998	Understand the meaning of a point reading of an incidence rate	80.7%	57.2%	1.4 (1.2-1.6)	76.8%	45.6%	1.7 (1.2-2.3)	81.9%	62.9%	1.3 (1.1-1.5)
Line graph of age-standardised incidence and death rates (vertical axis) for all cancers, by sex and year (horizontal axis).	1. Plain series labels: changed "Incidence..." to "New cases (incidence)..." and "Mortality..." to "Deaths..."<br><br>2. Footnote explaining how to interpret age standardised rates.	Understand the influence of age standardisation on comparisons between incidence rates for males and females.	58.0%	36.9%	1.6 (1.3-2.0)	42.9%	22.8%	1.9 (1.1-3.3)	65.5%	44.4%	1.5 (1.2-1.9)
Illustration B: Incident DALY Rates per 1,000 Population by Mental Disorder, Age and Sex, Victoria 1996<br>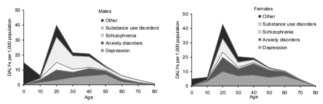	Illustration B: Incident DALY Rates per 1,000 Population by Mental Disorder, Age and Sex, Victoria 1996<br>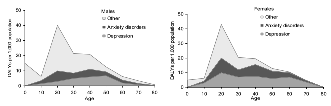 Note: The thickness of the shaded layer = DALYs per 1,000 population for that disorder	For a single disorder, estimate the difference between incidence rates between two age points.	57.4%	57.8%	1.0 (0.8-1.2)	51.8%	47.4%	1.1 (0.8-1.6)	60.3%	63.7%	0.9 (0.8-1.2)
A pair of stacked line graphs (area or layer graphs) of incident rates of disability-adjusted life-years (DALYs) (vertical axis) for selected mental disorders, by age (horizontal axis). Each graph in the pair represented males and females, respectively.	1. Reduced the number of categories of mental disorders from five to three, with the removed categories combined into the 'other' category.<br><br>2. Footnote explaining what the thickness of a layer represents.	Compare an incidence rate reading for a disorder between two sexes.	85.2%	88.2%	1.0 (0.9-1.1)	83.9%	82.5%	1.0 (0.9-1.2)	87.1%	90.3%	1.0 (0.9-1.1)
		Understand the shape of the incidence rate trend by age for one disorder and one sex.	69.9%	84.0%	0.8 (0.7-0.9)	58.9%	80.7%	0.7 (0.6-0.9)	75.0%	86.3%	0.9 (0.8 - 1.0)
		Understand that the topmost series represents the overall rate and compare the overall rate for the same age range between the two graphs.	89.2%	85.6%	1.0 (1.0-1.1)	89.3%	87.7%	1.0 (0.9-1.2)	90.5%	83.9%	1.1 (1.0-1.2)

Table 2 (Continued). Comparison of the proportion of correct answers between the intervention ("Int.") and control ("Con.") groups for each intervention tested, and by highest level of education attained.

Graph description	Intervention(s)	Interpretation task	All respondents Int. % (N=176)	All respondents Con. % (N=187)	All respondents Ratio (95% C.I.)	Non university-qualified Int. % (N=56)	Non university-qualified Con. % (N=57)	Non university-qualified Ratio (95% C.I.)	University-qualified Int. % (N=116)	University-qualified Con. % (N=124)	University-qualified Ratio (95% C.I.)
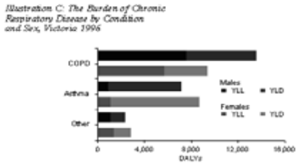	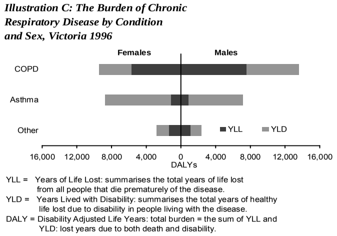	Compare the magnitude of YLL and YLD for a single disease category and sex.	65.9%	74.9%	0.9 (0.8-1.0)	69.6%	71.9%	1.0 (0.8-1.2)	64.7%	77.4%	0.8 (0.7-1.0)
A horizontal divided bar graph of disease burden in disability adjusted life years (DALY) (horizontal axis), for three categories of respiratory disease (vertical axis) and sex for a single year. DALY bars were divided into years of life lost (YLL) and years lived with a disability (YLD), because DALY = YLL+YLD. Sex was represented as adjacent bars within each category. Different shading was used for YLLs, YLDs and sex.	1. Changed the graph type to a side-by-side divided bar graph, each side representing a single sex. The same shading was used for both males and females.	Know that YLD represents "disability burden" and select the disease with the highest disability burden for a single sex.	32.4%	12.8%	2.5 (1.6-3.8)	33.9%	10.5%	3.2 (1.4-7.5)	31.9%	14.5%	2.2 (1.3-3.6)
	2. Footnote explaining acronyms YLL, YLD and DALY, and stating that DALYs are the sum of YLL and YLD.	Within a single disease category, compare the magnitude of YLLs between sexes.	85.8%	88.8%	1.0 (0.9-1.1)	83.9%	89.5%	0.9 (0.8-1.1)	87.9%	88.7%	1.0 (0.9-1.1)
		Select the disease with the highest DALY value for a single sex.	83.0%	67.9%	1.2 (1.1-1.4)	80.4%	61.4%	1.3 (1.0-1.7)	85.3%	71.8%	1.2 (1.0-1.4)
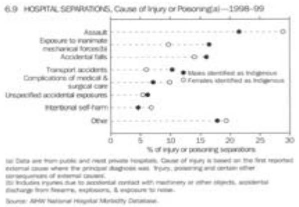	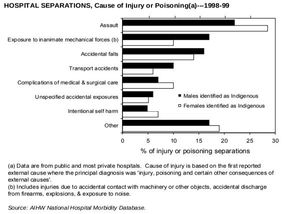	Judge which sex had the greater proportion for a single injury category.	93.8%	89.3%	1.1 (1.0-1.1)	92.9%	78.9%	1.2 (1.0-1.4)	94.8%	95.2%	1.0 (0.9-1.1)
A dot graph of the proportion of hospital separations (horizontal axis) by causes of injury and poisoning (vertical axis) in a time period, by sex. Sex was represented by a shaded or non-shaded dot, and each dot was connected to the vertical axis by a dashed line.	Changed the graph type to a horizontal bar graph with sex represented by differently shaded bars. The bars for each sex appeared adjacently for each injury category along the vertical axis.	Judge which injury category had the greatest proportion of hospital separations within a single sex.	96.0%	94.1%	1.0 (1.0-1.1)	94.6%	89.5%	1.1 (1.0-1.2)	97.4%	97.6%	1.0 (1.0-1.0)

Table 2 (Continued). Comparison of the proportion of correct answers between the intervention ("Int.") and control ("Con.") groups for each intervention tested, and by highest level of education attained.

			All respondents			Non university-qualified			University-qualified		
Graph description	Intervention(s)	Interpretation task	Int. % (N=176)	Con. % (N=187)	Ratio (95% C.I.)	Int. % (N=56)	Con. % (N=57)	Ratio (95% C.I.)	Int. % (N=116)	Con. % (N=124)	Ratio (95% C.I.)
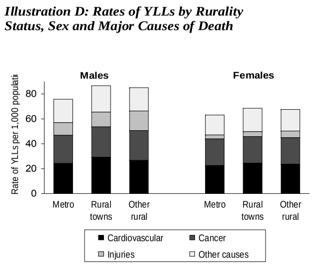	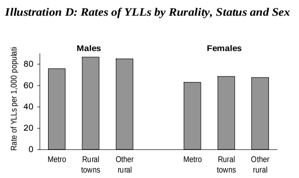	Read the total rate of YLLs for a single geographic category and sex.	93.8%	80.2%	1.2 (1.1-1.3)	89.3%	71.9%	1.2 (1.0-1.5)	96.6%	83.9%	1.2 (1.1-1.3)
Vertical divided bar graph of the mortality burden in years of life lost (YLL) rates (vertical axis) by three geographic categories and sex (horizontal axis), with each bar divided into four major disease groups. The geographic categories were presented in two groups by sex on the horizontal axis.	Removed one independent variable, the disease groupings, resulting in undivided bars. This also resulted in no legend and a shorter title as only overall totals were now represented by each bar.	Broad judgement of the relative magnitude of overall YLL rates between metropolitan and rural geographic categories, regardless of sex.	94.9%	90.4%	1.1 (1.0-1.1)	94.6%	84.2%	1.1 (1.0-1.3)	95.7%	94.4%	1.0 (1.0-1.1)
		Broad judgement of the relative magnitude of overall YLL rates between sexes, regardless of geographic category.	92.6%	92.5%	1.0 (0.9-1.1)	89.3%	84.2%	1.1 (0.9-1.2)	94.8%	96.0%	1.0 (1.0-1.1)
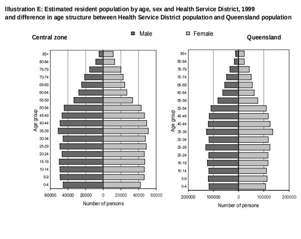	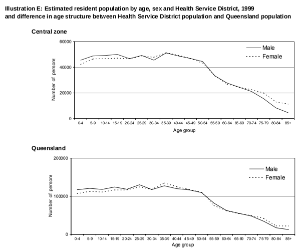	Broad comparison between males and females of the overall population count across a range of age groups, for one geographic area.	90.3%	78.1%	1.2 (1.1-1.3)	85.7%	77.2%	1.1 (0.9-1.3)	93.1%	78.2%	1.2 (1.1-1.3)
A pair of pyramid-style side-by-side bar graphs ('population pyramids') showing population counts (horizontal axis) by age group (vertical axis) and sex for two geographic areas. The geographic area on the leftmost graph was a zone within the other geographic area.	For each geographic area, the population counts for each sex were represented as two series on a horizontal format line graph.	Broad comparison of the total population size of the two geographic regions, regardless of age or sex.	78.4%	41.2%	1.9 (1.6-2.3)	73.2%	29.8%	2.5 (1.6-3.8)	81.9%	46.8%	1.8 (1.4-2.2)
		Broad comparison of the population size of younger and older segments of the population for both geographic areas.	89.2%	85.6%	1.0 (1.0-1.1)	83.9%	80.7%	1.0 (0.9-1.2)	92.2%	87.9%	1.1 (1.0-1.1)

Table 2 (Continued). Comparison of the proportion of correct answers between the intervention ("Int.") and control ("Con.") groups for each intervention tested, and by highest level of education attained.

Graph description	Intervention(s)	Interpretation task	All respondents Int. % (N=176)	All respondents Con. % (N=187)	All respondents Ratio (95% C.I.)	Non university-qualified Int. % (N=56)	Non university-qualified Con. % (N=57)	Non university-qualified Ratio (95% C.I.)	University-qualified Int. % (N=116)	University-qualified Con. % (N=124)	University-qualified Ratio (95% C.I.)
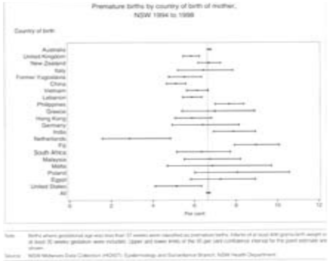	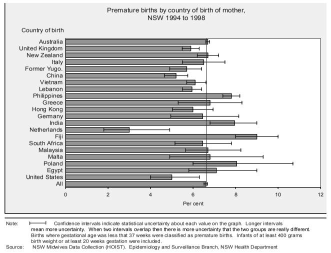	Interpret the statistical significance of the difference in proportions represented by two bars with clearly overlapping confidence ranges.	54.5%	31.6%	1.7 (1.4-2.2)	39.3%	15.8%	2.5 (1.3-4.9)	62.9%	40.3%	1.6 (1.2-2.0)
A dot graph with 95% confidence intervals ("hi-lo-close graph") comparing the proportion of births that were premature (horizontal axis) by the mother's country of birth (vertical axis). A vertical reference line indicated the overall proportion for all mothers.	1. Changed the graph type to a horizontal bar graph.<br><br>2. Footnote giving a practical explanation of confidence intervals and their interpretation.	Judge whether the proportion among mothers born in one country was higher or lower than that of the other country, where the difference is distinct.	91.5%	84.5%	1.1 (1.0-1.2)	92.9%	71.9%	1.3 (1.1-1.5)	91.4%	90.3%	1.0 (0.9-1.1)
		Judge whether the proportion among mothers born in one country was higher or lower than all mothers overall.	79.5%	50.3%	1.6 (1.4-1.9)	80.4%	35.1%	2.3 (1.6-3.3)	80.2%	58.1%	1.4 (1.2-1.7)
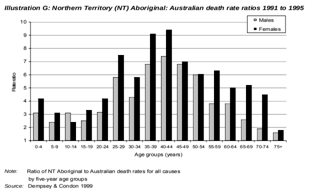	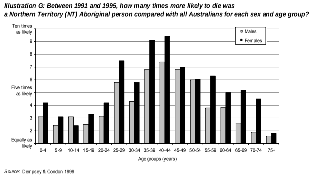	Broad judgement of whether Aboriginal people overall had a higher risk of death than most Australians.	82.4%	58.8%	1.4 (1.2-1.6)	69.6%	38.6%	1.8 (1.3-2.6)	90.5%	69.4%	1.3 (1.2-1.5)
A vertical bar graph comparing the ratio of death rates (vertical axis) between non-Aboriginal people of a geographic region with those of the country's overall population over a period of years, by age (horizontal axis) and sex. The two sexes were displayed as adjacent bars for each age group.	1. Plain graph title stating the primary question the graph answered: "…how many times more likely to die was an Aboriginal person compared with all Australians for each sex and age group?", instead of "…Aboriginal: Australian death rate ratios…".<br><br>2. Changed the vertical axis labels indicating ratios of 1, 5 and 10 to "Equally as likely", "Five times as likely" and "Ten times as likely" respectively.	For a specific age group and sex, read the point estimate of the ratio of the the two population groups' rates.	83.0%	55.6%	1.5 (1.3-1.7)	69.6%	36.8%	1.9 (1.3-2.8)	91.4%	65.3%	1.4 (1.2-1.6)
		Understand the meaning of a death rate ratio for a specific age group and sex.	84.7%	59.9%	1.4 (1.2-1.6)	71.4%	42.1%	1.7 (1.2-2.4)	92.2%	69.4%	1.3 (1.2-1.5)

Table 2 (Continued). Comparison of the proportion of correct answers between the intervention ("Int.") and control ("Con.") groups for each intervention tested, and by highest level of education attained.

Graph description	Intervention(s)	Interpretation task	All respondents Int. % (N=176)	All respondents Con. % (N=187)	All respondents Ratio (95% C.I.)	Non university-qualified Int. % (N=56)	Non university-qualified Con. % (N=57)	Non university-qualified Ratio (95% C.I.)	University-qualified Int. % (N=116)	University-qualified Con. % (N=124)	University-qualified Ratio (95% C.I.)
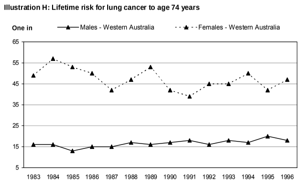	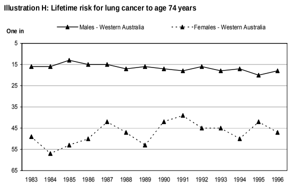	Judge the relative magnitude of risk between the sexes at one point along the horizontal axis.	79.5%	48.7%	1.6 (1.4-1.9)	66.1%	31.6%	2.1 (1.4-3.2)	87.1%	58.1%	1.5 (1.3-1.8)
Line graph of the lifetime risk of experiencing lung cancer (vertical axis) by year (horizontal axis) and sex. Risk labels on the vertical axis were expressed as a number, x, where the number represented a one in x risk. Numbers increased from the bottom to the top of the axis, such that higher values meant lower risk.	The vertical axis was reversed so that labels went from a large number at the bottom to a smaller number at the top. This meant that graph values towards the top of the graph represented higher risk.	For a single sex, judge the direction of the trend over time in terms of increasing or decreasing risk.	60.2%	20.9%	2.9 (2.1-9.9)	62.5%	19.3%	3.2 (1.8-5.7)	58.6%	21.8%	2.7 (1.9-3.9)
		Read the point estimate of the risk for a single sex in a single year.	90.9%	85.6%	1.1 (1.0-1.1)	78.6%	77.2%	1.0 (1.0-1.4)	97.4%	91.1%	1.1 (0.9-1.1)
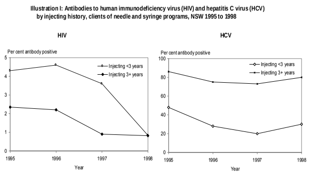	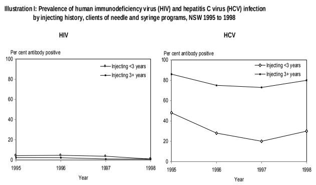	Broad judgment of whether both viruses had the same relative difference in prevalence between the two injecting history groups in a single year. This required a comparison both within and between the two graphs.	90.9%	45.5%	2.0 (1.7-2.4)	89.3%	35.1%	2.5 (1.8-3.7)	93.1%	51.6%	1.8 (1.5- 2.2)
A pair of graphs showing the prevalence (vertical axis) of having antibodies to human immunodeficiency virus (HIV) and hepatitis C virus (HCV) among clients of needle and syringe programs, by year (horizontal axis) and two categories of injecting history. The two graphs represented HIV and HCV respectively.	1. Made the vertical axis on both graphs cover the same range, 0-100%. Because the prevalence of HIV infections was very low, this vertically compressed the series in the left graph.<br><br>2. Plainer graph title: "Prevalence of human immunodeficiency virus (HIV) and hepatitis C virus (HCV) infection…" instead of "Antibodies to human immunodeficiency virus (HIV) and hepatitis C virus (HCV)…".	Judge which injecting history group had a lower prevalence of HCV infection over the years shown on the graph.	80.7%	75.9%	1.1 (1.0-1.2)	78.6%	66.7%	1.2 (1.0-1.5)	81.9%	79.8%	1.0 (0.9-1.2)
		Broad judgment of the which virus had a greater prevalence of infection in a single year, regardless of injecting history. This required a comparison between the two graphs.	92.0%	63.6%	1.5 (1.3-1.6)	87.5%	47.4%	1.9 (1.4-2.5)	94.8%	73.4%	1.3 (1.2-1.5)
		Read a point estimate of HCV infection prevalence for a single year and injecting history category.	71.0%	73.3%	1.0 (0.9-1.1)	64.3%	63.2%	1.0 (0.8-1.3)	74.1%	78.2%	1.0 (0.8-1.1)

Table 2 (Continued). Comparison of the proportion of correct answers between the intervention ("Int.") and control ("Con.") groups for each intervention tested, and by highest level of education attained.

Graph description	Intervention(s)	Interpretation task	All respondents Int. % (N=176)	All respondents Con. % (N=187)	All respondents Ratio (95% C.I.)	Non university-qualified Int. % (N=56)	Non university-qualified Con. % (N=57)	Non university-qualified Ratio (95% C.I.)	University-qualified Int. % (N=116)	University-qualified Con. % (N=124)	University-qualified Ratio (95% C.I.)
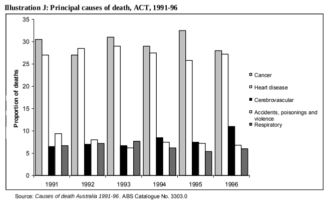	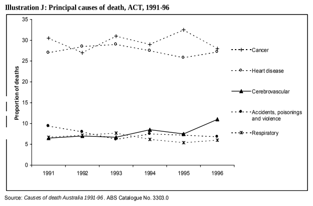	Read the point estimate of the proportion of deaths caused by a disease category in one year.	83.0%	82.9%	1.0 (0.9-1.1)	78.6%	73.7%	1.1 (0.9-1.3)	86.2%	88.7%	1.0 (0.9-1.1)
A vertical bar graph showing the proportion of deaths (vertical axis) contributed by each of five major disease categories, by year (horizontal axis). The bars for the disease categories were grouped together at each year along the horizontal axis.	Changed the graph type to a line graph with each series representing the trend in each disease category over time.	Judge which disease category had the lowest proportion of deaths in a single year.	96.6%	94.1%	1.0 (1.0-1.1)	96.4%	87.7%	1.1 (1.0-1.2)	97.4%	97.6%	1.0 (1.0-1.0)
		Judge which disease category had the most increasing trend in the proportion of deaths over the period of the graph.	83.5%	76.5%	1.1 (1.0-1.2)	75.0%	56.1%	1.3 (1.0-1.8)	89.7%	85.5%	1.1 (1.0-1.2)
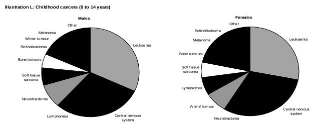	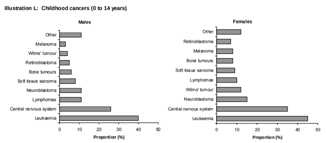	Identify the cancer category accounting for the largest proportion of cancers in a single sex.	97.7%	96.8%	1.0 (1.0-1.1)	96.4%	93.0%	1.0 (1.0-1.1)	99.1%	100.0%	1.0 (1.0-1.0)
A pair of pie charts showing the proportional contribution of individual cancer categories to all cancers in children. Each graph represents a single sex.	Changed the graph type to a horizontal bar graph with cancer category on the vertical axis and proportion as a percentage on the horizontal axis.	Identify the larger of two categories for a single sex.	96.6%	95.2%	1.0 (1.0-1.1)	94.6%	93.0%	1.0 (1.0-1.1)	98.3%	97.6%	1.0 (1.0-1.1)
		Identify the sex having the greater contribution of the same cancer category. This required comparison across the two graphs.	95.5%	63.6%	1.5 (1.3-1.7)	92.9%	80.7%	1.2 (1.0-1.3)	97.4%	56.5%	1.7 (1.5-2.0)
		Identify the cancer category accounting for the smallest proportion of cancers in a single sex.	96.6%	90.9%	1.1 (1.0-1.1)	94.6%	91.2%	1.0 (0.9-1.2)	98.3%	91.9%	1.1 (1.0-1.1)
		Estimate the point reading of the proportion of cancers for a single category for a single sex.	92.0%	25.7%	3.6 (2.8-4.6)	91.1%	40.4%	2.3 (1.6-3.1)	93.1%	19.4%	4.8 (3.4-6.9)

Discussion

We believe this is the first randomised, controlled trial assessing interventions aimed at increasing readers' ability to understand statistical information about population health. In fact, the evidence-base for graph comprehension and related cognitive processes in general is largely limited to studies conducted in laboratory settings with small groups of subjects, usually university students. We are aware of only one other study that randomly selected subjects from a defined population, and it had a response rate of 50%.[2] Further, we found only a limited number of randomised, controlled study designs in the graph literature.[2,3,4]

Our findings are of benefit from two perspectives. First, we were able to quantify the proportion of readers who could extract some typical statistical interpretations from a sample of graphs used in Australian official population health publications. Depending on the graph and the specific interpretation sought, the proportion of readers able to correctly interpret the graphs ranged from as few as 13% to as many as 97%. Second, we were able to quantify the impact on comprehension levels achieved through the simple changes we applied to the graphs. This resulted in a maximum three to four-fold increase in the proportion of readers who correctly extracted specific information from the graphs.

Titles and labels

While recommendations have been made about graph titles or captions and labels,[5,6,7,8,9] there is little evidence relating to techniques for making their content easily understood.

The most dramatic result of the study related to a vertical bar graph showing that Aboriginal people in a region of Australia had an increased risk of mortality at every age compared with the general population; in some age groups the increase was almost ten-fold. More than 40% of control subjects (60% of those without university qualifications) were unable to determine from the graph the simple fact that Aboriginal people had a higher risk of death. A combination of interventions that included a simple title expressing the question that was answered by the graph and the addition of words on the vertical axis that directly related to the title, more than halved the proportion of subjects who did not grasp this fact.

People working in public health and epidemiology regard the concept of disease incidence as quite commonplace. However, we found that less than 60% of all subjects, and less than half of non university-qualified subjects, could answer a question that required an understanding that disease incidence refers to the rate of *new cases* of disease in a period of time. Simply changing the label on the incidence rate series from 'Incidence…' to 'New cases (incidence)...' had a statistically significant benefit in both university and non-university qualified subjects.

Footnotes

To our knowledge, there is no literature on whether graph readers understand statistical concepts used in graphs, despite some recommendations being available.[7,9] Two statistical techniques and concepts occur frequently in population health graphs: age standardisation and confidence intervals. We hypothesised that interpretive tasks requiring an understanding of these concepts would be difficult for people without specialist knowledge. This was borne out, with the effect of age standardisation being understood by only 23% and 44% of non university-qualified and university qualified subjects respectively. For a task requiring the interpretation of overlapping confidence limits, the proportions were 16% and 40% respectively. We further hypothesised that a footnote providing a simple, practical explanation of the concepts and their interpretation, could improve the level of understanding, and this was also borne out, with improvements of up to 2.5-fold in one of the tasks among non-university qualified subjects.

A footnote explaining acronyms that would not be known to a general audience increased the correct response to an interpretation task by between two and three-fold depending on level of education.

However, not all footnotes are successful. The explanatory footnote that we added to a stacked layer graph (which differs from other graph types because values for the component categories cannot be read directly from the axis) had no benefit for any of the interpretative tasks we investigated and in fact had a detrimental effect on one task among non-university educated subjects. We speculate that this particular footnote confused rather than assisted many readers.

Volume of information

Reducing unnecessary information in graphs should improve reader performance,[10,11,12] but by how much? We tried two interventions aimed at reducing the volume of information to be interpreted.

First, we reduced the number of categories for which results were presented in the stacked layer graph. This did not offer a benefit for the interpretations we investigated.

Second, we completely removed an independent (categorisation) variable from a vertical bar graph that originally presented results for a quantity against three independent variables within the one graph. Without the intervention, the graph was reasonably well understood with the lowest proportion of correct answers being 72% among non university-qualified subjects for a task requiring the estimated total quantity represented by one of the bars. Despite this, the intervention raised comprehension by 20% even among university-educated subjects.

Graph types

We investigated the relative value of line and bar graphs for displaying information that is plotted against a categorical x axis that represents a numeric quantity, such as year or age. A line graph and a grouped bar graph of multiple disease trends by year performed equally well for point-reading tasks, but the line graph produced a marginal improvement in trend judgement in subjects without a university education. This is as expected; bar graphs encourage discrete rather than trend-based comparisons,[13] although bar graphs have been found to be versatile.[14,15]

The ‘population pyramid’ is a popular choice for representing the age distribution by sex of a population. It is in fact a vertically oriented side-by-side bar graph. It can however, also be represented as a horizontal format line graph with two series, each series showing the population size by age for each sex. Among, surprisingly, university-educated subjects only, the line graph improved broad comparison of the shape of the population distribution by sex.

Dot graphs have been proposed as an improvement on bar graphs.[16] We found that a bar graph with 95% confidence intervals clearly out-performed dot graphs with 95% confidence intervals (sometimes called ‘hi-lo-close” graphs), particularly among those without university qualifications. For another type of dot graph that had each dot connected by a dashed line to the x axis, but no confidence intervals, a horizontal bar graph performed equally well, and even showed a marginal improvement for those without a university education. Given that bar graphs are probably more familiar to general readers and given their ready availability in common statistical software products, we would recommend the use of bar graphs over dot-based graphs.

The stacked layer graph worked well for some tasks requiring interpretation of trend and broad comparisons, as expected,[7] but worked poorly for a task requiring the estimation of a difference between the absolute rate at two points along a layer. This highlights the unsuitability of these graphs for communicating absolute levels of a quantity because point estimates for a single category cannot be read directly from the axis.

Pie charts are often derided because their non-linear format inhibits precise estimation of statistical quantities.[17,18] However, they do provide a visual representation of how each category contributes to the whole.[7] This is not easily achieved with other graph styles. The difficulty of estimating specific quantities or judging subtle differences from pie charts was borne out in this study. For simple quantitative tasks such as identifying minimum and maximum categories or making comparisons where the differences were distinct, the pie chart performed as well as a bar chart. If an important aim is to visually represent how each category contributes to the whole, then a useful recommendation would be to use pie charts but ensure the actual quantities are labelled on each segment of the pie chart.

Scales and axes

Several of our graphs explored the consequences of using differing scales in adjacent graphs. Many respondents, particularly those without university qualifications, appeared to answer questions based on visual relativities rather than from studying the labels on the axes. For tasks comparing the relative magnitude of quantities between the two graphs, a matching scale range on each graph greatly improved comprehension. If comparisons between adjacent graphs are important then the same axis range should be used to avoid confusion. This is consistent with Kosslyn's recommendation,[7] and should serve as a qualification of Cleveland's recommendation that data should fill the graph space.[6] If such comparisons are not important, then the two graphs should be presented with a clear visual separation.

We found strong evidence for ensuring that higher values of the quantity presented on the graph be in the upward direction, even if this means the numerical labels are decreasing in the upward direction. This situation can arise when the risk of experiencing a disease is expressed as 'one in *x*', and *x* is the quantity graphed, because, for example, a one in 20 risk is larger than a one in 50 risk. Although this finding may be culturally-specific, it would be reasonable to assume that for a horizontally oriented graph, the left to right direction should represent increasing values.

Limitations of the study

Several issues need to be borne in mind when considering the findings of our study.

Despite the randomised design, there were differences between the control and intervention groups in terms of self-rated visual ability and frequency of graph use. Intervention subjects were somewhat more likely to rate themselves as frequent graph users than control subjects and were more likely to rate themselves as having good visual ability. However, the observed differences may reflect the fact that many of the intervention graphs were more easily understood than the control graphs. These questions were asked at the end of the questionnaire, and intervention subjects may have felt more comfortable rating themselves more highly on these characteristics.

The results we obtained would be an overestimate of levels of comprehension that would be achieved in the general population. People working in public health and policy-related areas represented approximately one-fifth of respondents. These employees would be most likely to require information on population health statistics for their work. Many other people in the health system would have a professional understanding of health and medicine. Two-thirds of respondents in our study had university qualifications, compared with approximately one-fifth of persons aged 25-64 in Australia.[19]

The graphs we used were taken out of the context of their original report and we recognise that much of the explanatory information required to understand the graph may have been contained in the surrounding text. Nevertheless, if readers unfamiliar with the subject are required to hunt for explanatory information, they may weary of obtaining knowledge about population health. Publishers of scientific journals often require graphs to be able to 'stand alone', and we support this objective, but would add that for documents that are intended for a public audience, the graphs should stand-alone for a broad sector of the reading population.

Finally, because in some cases we made more than one change to the intervention graph, we could not completely isolate the impacts of each of the changes made. However, we aimed to minimise this difficulty by making the questions as specific as possible to the anticipated effects of each of the changes we made. This approach balanced respondent burden with the need to test the effects of a number of changes.

Recommendations

Use plain, non-technical language in the graph title and graph components

Techniques that could be considered include:

- Express the graph title as a simple question that is answered by the graph.
- Express technical terms in non-technical terms followed by the technical term in parentheses.
- Replace numeric axis labels with descriptive text that explicitly states the meaning of the quantities they represent.
- Explain complex concepts in a simply worded footnote.
- Don’t use acronyms unless their meaning is clearly labelled in close proximity to the graph.

Use the minimum number of sub-categories (independent variables) necessary

The graph examples we examined used a variety of techniques to delineate the quantities expressed for different population groups. The techniques included plotting the equivalent graphs as a pair for males and females, grouping graph bars along the x axis according to sex or disease category, and/or dividing bars into segments according to some sub-categorisation.

While these techniques increase the volume of information that can be communicated, they also increase the visual complexity of the information. For example, dividing bars into segments, mean that the quantity expressed by the length of the segment cannot be read directly from the y axis, and because the reference position of the segments varies from one bar to the next, comparison of length is hindered.

If the difference between two quantities is more important than the quantities themselves, consider plotting a graph of the differences, rather than the two individual quantities.

Use conventional line or bar graphs where possible

Often simpler graph styles communicate as well as, or better than, more complex or less common designs. Graphs that can be simplified include ‘population pyramids’, dot graphs with confidence limits (‘hi-lo-close’) graphs and dot graphs which connect the dots to the x axis.

Recognise that the interpretation of confidence intervals and age standardisation requires technical knowledge

Consider methods of simplifying the interpretation of these concepts. Simple footnotes help dramatically, but not completely.

Assist readers to interpret ratios

Using plain, non-technical titles and labels as described above, assist readers to recognise that a ratio represents the number of times bigger the numerator quantity is than the denominator quantity. This can apply to rate ratios or relative risks, for example.

Ensure that quantities (not labels) increase from the bottom to the top or left to right

This is a problem when graphing disease risk expressed as ‘One in x’, for example, where a higher value of x means a lower risk. The graph should be drawn so that risk increases from the bottom to top or left to right, depending on the orientation of the graph. This means the numeric labels on the risk axis will increase in the opposite direction to risk, but this will ensure that readers will interpret relative changes within the graph in the correct direction.

If using pie charts, label the quantity represented by each segment on or near the segment

Pie charts do have limitations for comparing relative magnitudes, but are useful for conveying part-to-whole relationships. Although not tested in our study, it is likely that the limitations can be overcome by labelling the quantities represented by each segment on the graph itself. If the part-to-whole relationship is not important, a bar graph will serve just as well.

If presenting graphs in pairs, ensure the axes have the same ranges

Graphs that are presented in pairs or groups imply that they have a relationship. Visual comparisons will take precedence over the details of axis labels, so ensure that visual impressions are meaningful by using the same axis ranges.

Use line graphs to represent trend information rather than bar graphs

For some readers, a line graph performs better than a bar graph for assessing trends, without affecting other tasks. This applies to graphs where the x axis gives the opportunity to assess trends over time, age or some other continuous or ordinal variable.

Conclusion

Our study provided new evidence to support a range of recommendations about how to improve the design of population health graphs. These provide a clear opportunity to improve delivery of public health messages through graphs to a wider sector of the population. Fortunately, this can be achieved through greater simplicity rather than greater complexity.

However, it is clear that, regardless of graph design, concepts such as age standardisation and confidence intervals were not understood by the majority of subjects, regardless of their level of education. This is a vexed problem, because these concepts are crucial to accurate interpretation of statistical information in population health and epidemiology. There remains, therefore, an opportunity for inventive thought on delivering the messages implied by these manipulations without increasing the complexity of the graph.

References

1 Australian Institute of Health and Welfare and the National Public Health Information Working Group 1999, National Public Health Information Development Plan, Canberra: Australian Institute of Health and Welfare.

2 Henry GT 1993, Using graphical displays for evaluation, *Evaluation Review*; 17(1): 60-78.

3 Meyer J, Shinar D 1992, Estimating Correlations from Scatterplots, *Human Factors*; 34(3): 335-349.

4 Lee ML, MacLachlan J 1986, The Effects of 3D Imagery on Managerial Data Interpretation, *MIS Quarterly*; September: 257-268.

5 Schmidt CF 1983, *Statistical Graphs Design Principles and Practices*, New York: John Wiley and Sons.

6 Cleveland WS 1994, *The Elements of Graphing Data*, Murray Hill NJ: AT and T Bell Laboratories.

7 Kosslyn SM 1994, *Elements of Graph Design*, New York: WH Freeman and Company.

8 Gillan DJ 1994, A Componential Model of Human Interaction with Graphs: I Linear Regression Modelling, *Human Factors*; 36(3): 419-440.

9 Gillan DJ, Wickens CD, Hollands JC, Carswell CM 1998, Guidelines for Presenting Quantitative Data in HFES Publications, *Human Factors*: 40(1): 28-41.

10 Schutz HG 1961, An Evaluation of Methods for Presentation of Graphic Multiple Trends, *Human Factors*; 3: 108-119.

11 Casali JG, Gaylin KB 1988, Selected Graph Design Variables in Four Interpretation Tasks: A Microcomputer-Based Pilot Study, *Behaviour and Information Technology*; 7(1): 31-49.

12 Kosslyn SM 1989, Understanding Charts and Graphs, *Applied Cognitive Psychology*; 3: 185-226.

13 Zacs J, Tversky, B 1999, Bars and lines: A study of graphic communication, *Memory and Cognition*; 27(6): 1073-1079.

14 Shah P, Mayer RE, Hegarty M 1999, Graphs as Aids to Knowledge Construction: Signaling Techniques for Guiding the Process of Graph Comprehension, *Journal of Educational Psychology*; 91(4): 690-702.

15 Carswell CM, Ramzy C 1997, Graphing Small Data Sets: Should We Bother? *Behaviour and Information Technology*; 16: 61-71.

16 Cleveland WS, McGill R 1984, Graphical perception: theory, experimentation and application to the development of graphical methods, *Journal of American Statistical Association*; 79: 531-554.

17 Tufte ER 1983, *The Visual Display of Quantitative Information*, Cheshire CT: Graphics Press, 1983.

18 Cleveland WS, McGill R 1985, Graphical Perception and Graphical Methods for Analyzing Scientific Data, *Science*; 229: 828-833.

19 Australian Bureau of Statistics 2005, Australian Social Trends 2005 (Catalogue 4102.0). Canberra: Australian Bureau of Statistics.

Appendix 1.
The control booklet of graphs

Booklet of graphs

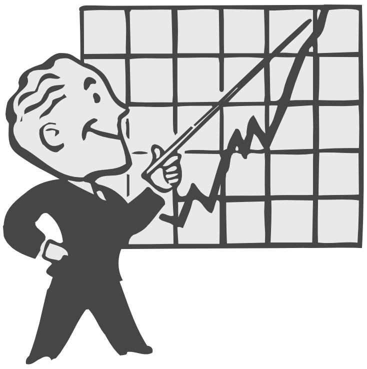

Better health graphs project

Introduction

The NSW Department of Health has asked the Hunter Valley Research Foundation (HVRF) to conduct a study to determine guidelines for designing informative and useful graphs. Graphs are an important tool for communicating health related information. Your participation in completing the accompanying questionnaire will be greatly appreciated.

Instructions

This booklet contains examples of different health graphs. You do not need to know the topic of the graph. In fact we ask you to answer all questions from the information in each graph, not from any knowledge you may have on the subject.

Please write all answers in the questionnaire booklet supplied.

Any questions?

Should you have any questions regarding this research, feel free to contact Andrew Searles at the HVRF on (02) 4969 4566 (extension 525). Alternatively, call David Muscatello of NSW Health on (02) 9391 9408.

Illustration A

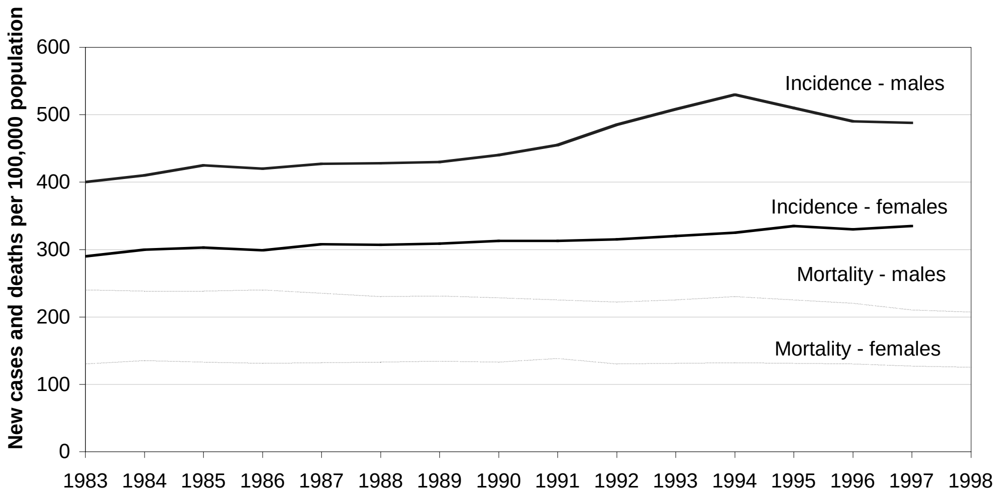

Source: Cancer in Australia 1997, AIHW & AACR 2000.

Illustration A: Trends in age-standardised incidence and mortality rates for all cancers (excluding non-melanocytic skin cancers), Australia, 1983-1998

Illustration B

Illustration B: Incident DALY Rates per 1,000 Population by Mental Disorder, Age and Sex, Victoria 1996

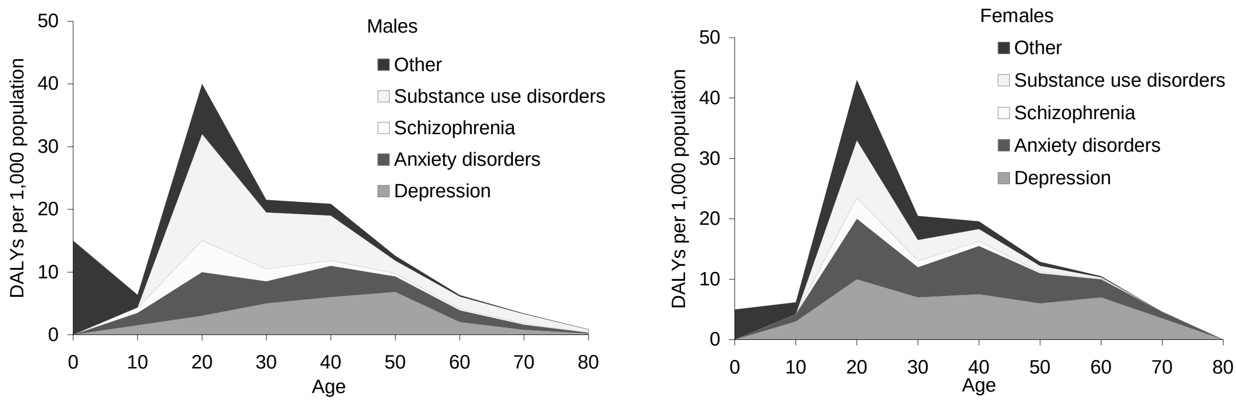

Illustration C

Illustration C: The Burden of Chronic Respiratory Disease by Condition and Sex, Victoria 1996

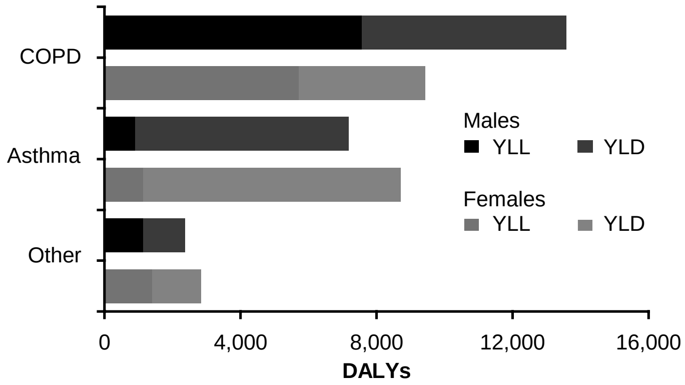

Illustration D

Illustration D: Rates of YLLs by Rurality Status, Sex and Major Causes of Death

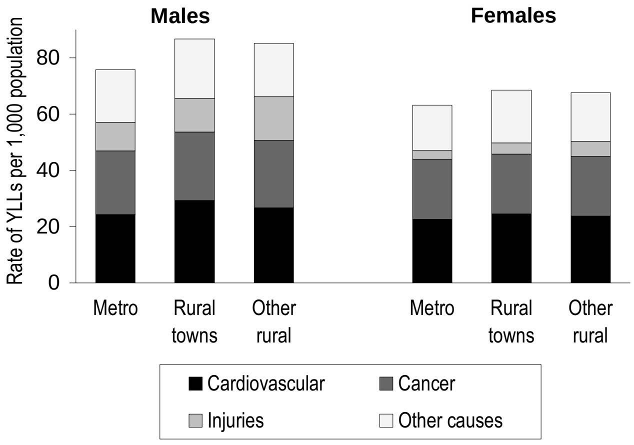

Illustration E

Illustration E: Estimated resident population by age, sex and Health Service District, 1999 and difference in age structure between Health Service District population and Queensland population

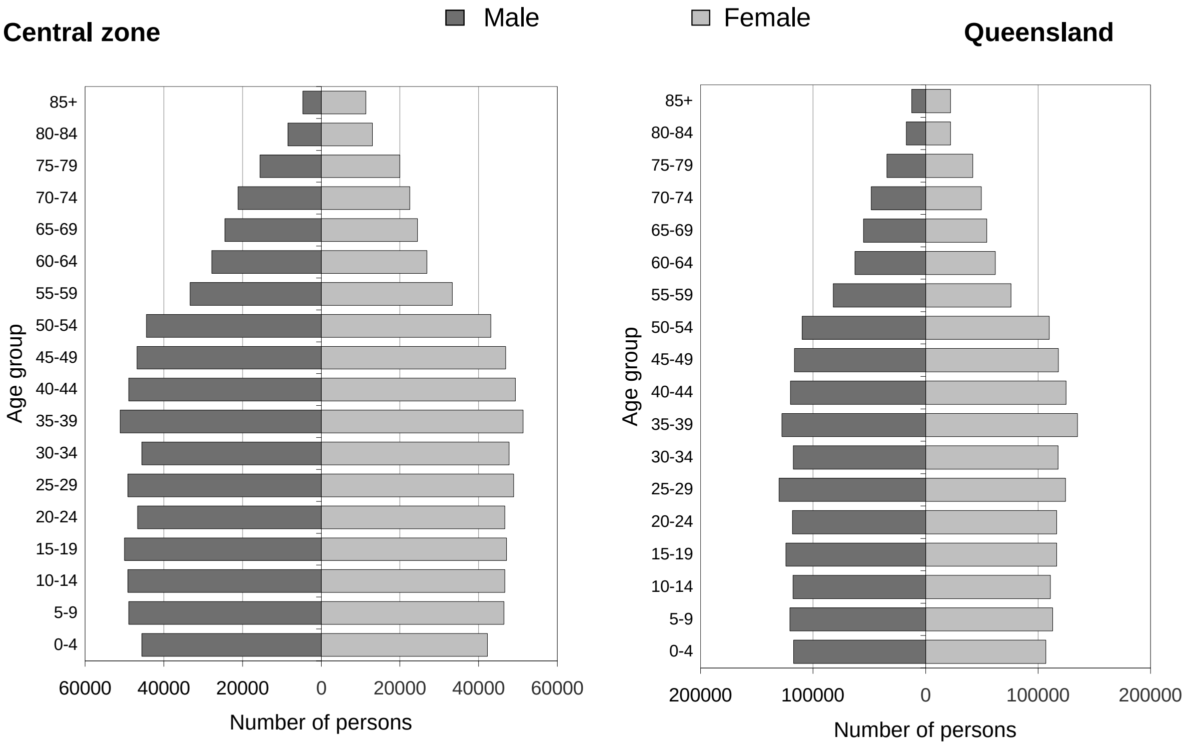

Illustration F

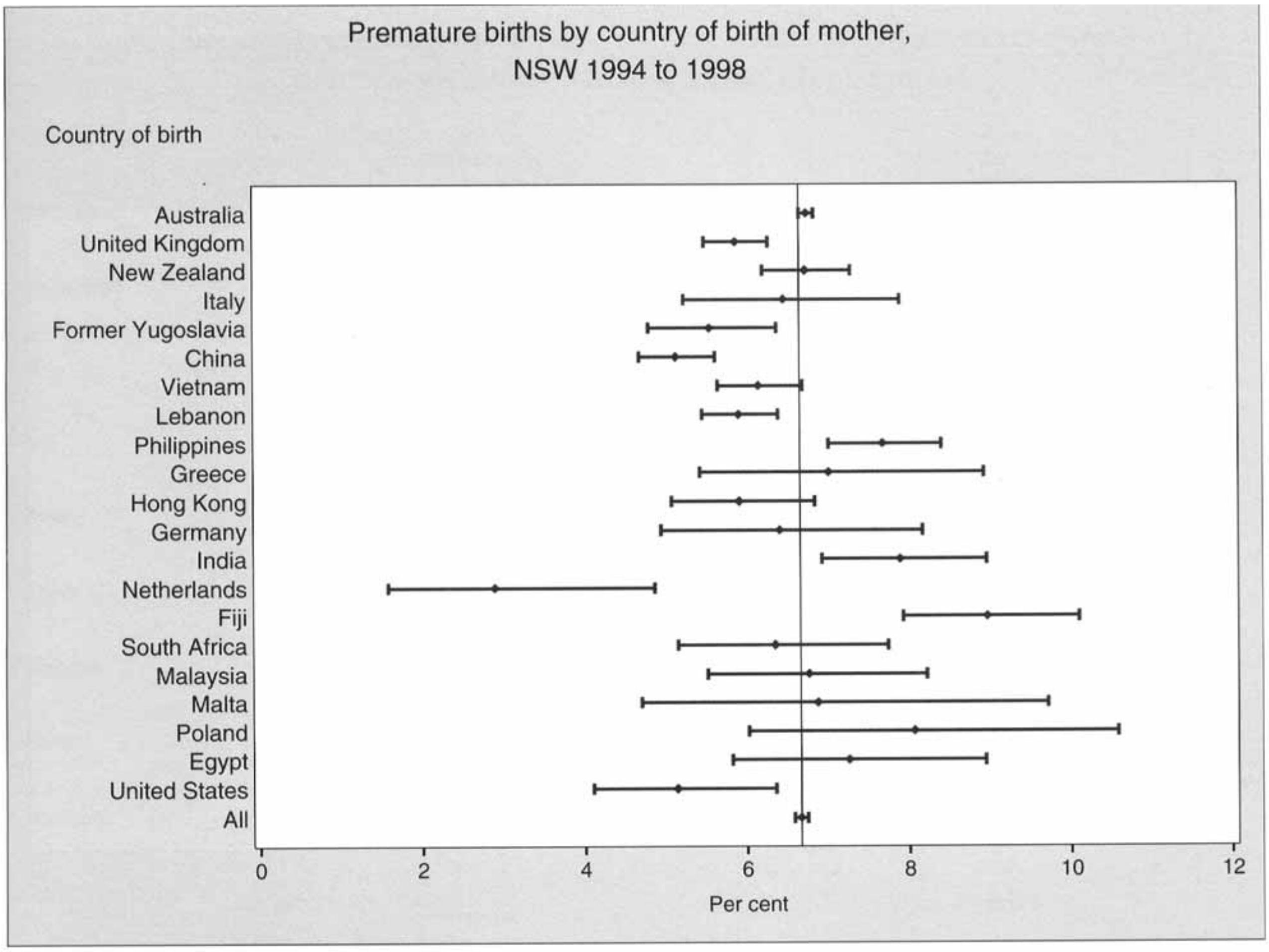

Note: Births where gestational age was less than 37 weeks were classified as premature births. Infants of at least 400 grams birth weight or at least 20 weeks gestation were included. Upper and lower limits of the 95 per cent confidence interval for the point estimate are shown.

Source: NSW Midwives Data Collection (HOIST). Epidemiology and Surveillance Branch, NSW Health Department.

Illustration G

Illustration G: Northern Territory (NT) Aboriginal: Australian death rate ratios 1991 to 1995

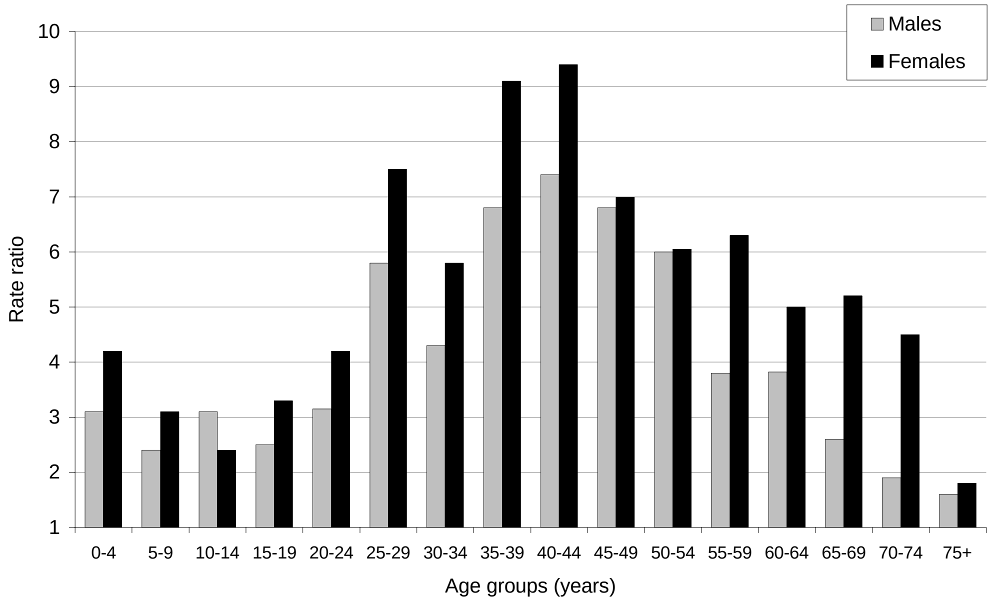

Note: Ratio of NT Aboriginal to Australian death rates for all causes by five-year age groups

Source: Dempsey & Condon 1999

Illustration H

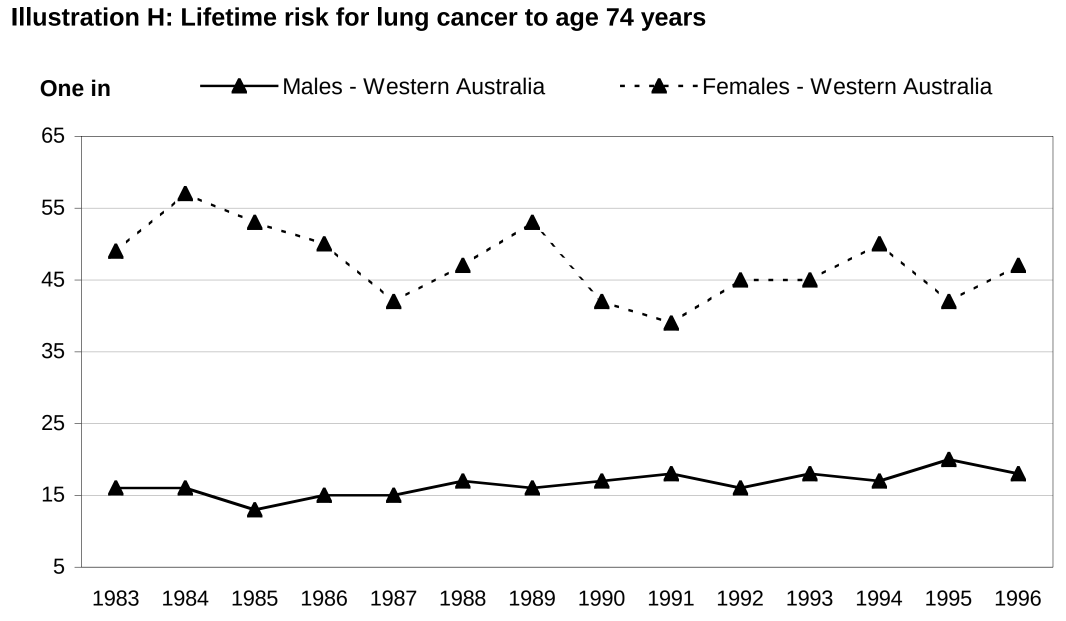

Illustration I

Illustration I: Antibodies to human immunodeficiency virus (HIV) and hepatitis C virus (HCV) by injecting history, clients of needle and syringe programs, NSW 1995 to 1998

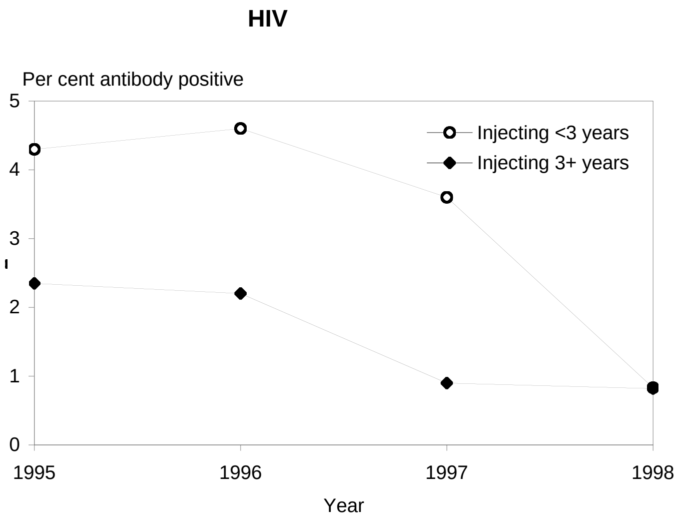

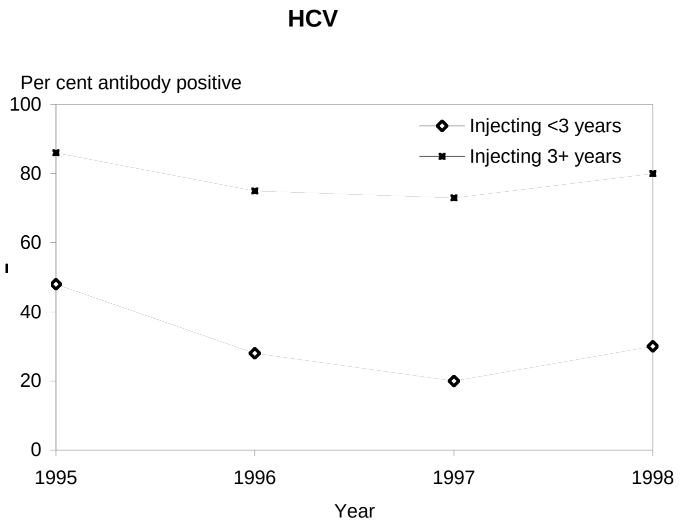

Illustration J

Illustration J: Principal causes of death, ACT, 1991-96

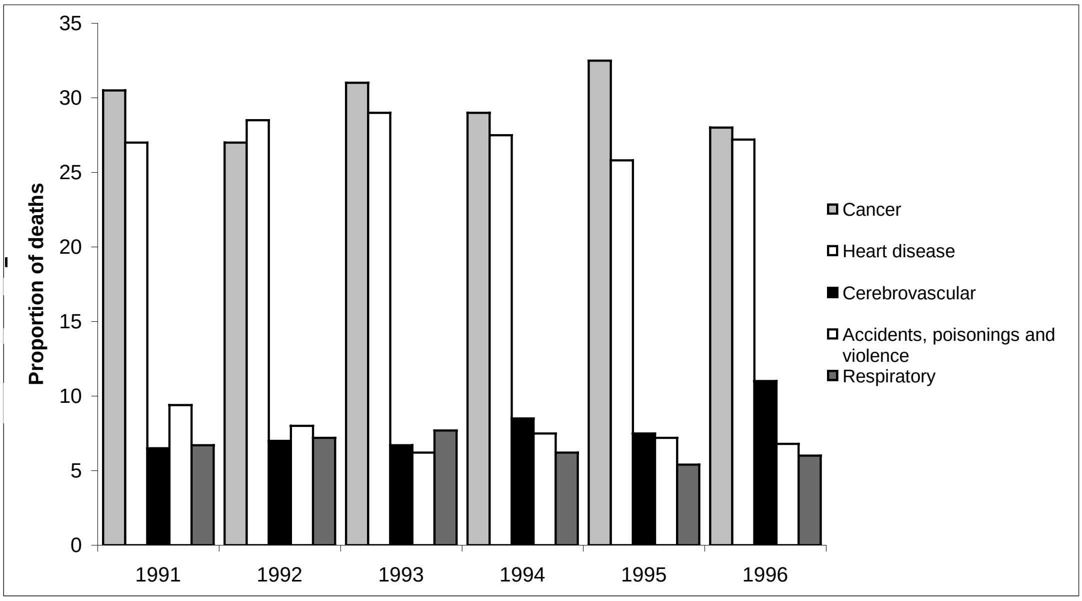

Source: *Causes of death Australia 1991-96*. ABS Catalogue No. 3303.0

Illustration K

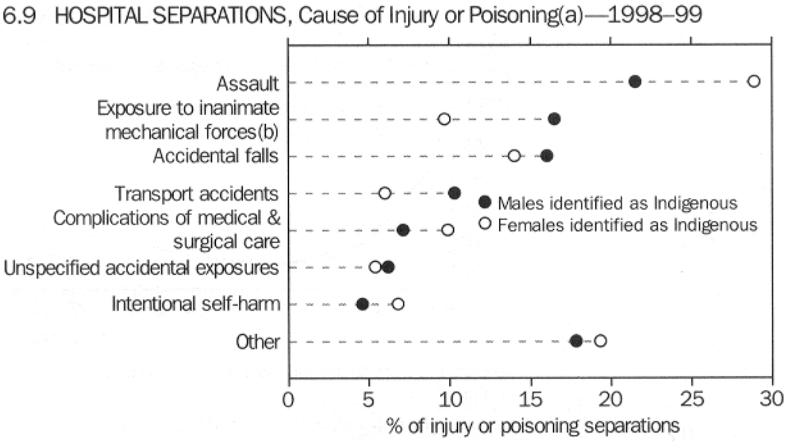

(a) Data are from public and most private hospitals. Cause of injury is based on the first reported external cause where the principal diagnosis was 'Injury, poisoning and certain other consequences of external causes'.

(b) Includes injuries due to accidental contact with machinery or other objects, accidental discharge from firearms, explosions, & exposure to noise.

Source: AIHW National Hospital Morbidity Database.

Illustration L

Illustration L: Childhood cancers (0 to 14 years)

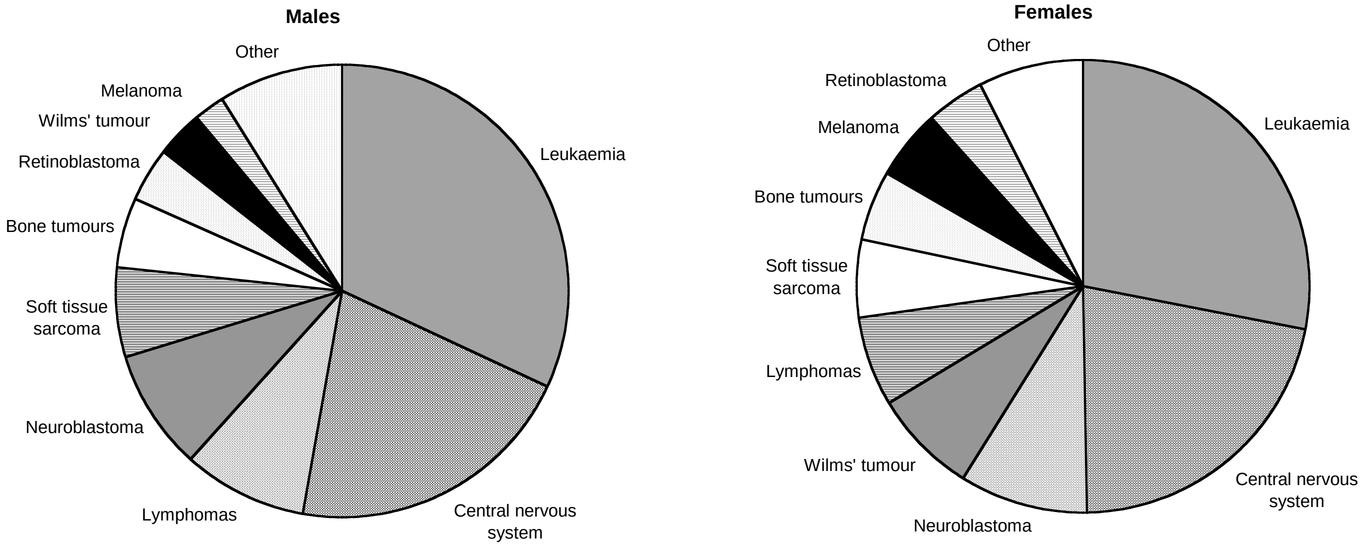

Appendix 2.
The intervention booklet of graphs

Booklet of graphs

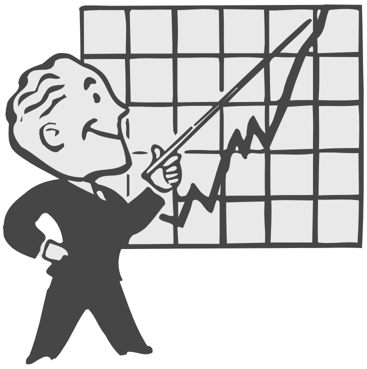

Better health graphs project

Introduction

The NSW Department of Health has asked the Hunter Valley Research Foundation (HVRF) to conduct a study to determine guidelines for designing informative and useful graphs. Graphs are an important tool for communicating health related information. Your participation in completing the accompanying questionnaire will be greatly appreciated.

Instructions

This booklet contains examples of different health graphs. You do not need to know the topic of the graph. In fact we ask you to answer all questions from the information in each graph, not from any knowledge you may have on the subject.

Please write all answers in the questionnaire booklet supplied.

Any questions?

Should you have any questions regarding this research, feel free to contact Andrew Searles at the HVRF on (02) 4969 4566 (extension 525). Alternatively, call David Muscatello of NSW Health on (02) 9391 9408.

Illustration A

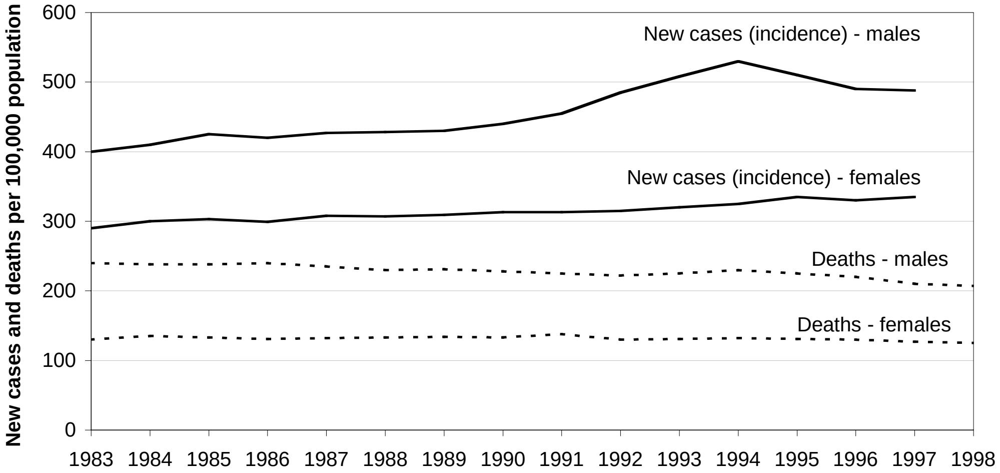

Note: age-standardised rates allow comparisons over years and between males and females. Different age-standardised rates are *not* due to differences in the relative proportions of older or younger people in each year or sex.

Source: Cancer in Australia 1997. AIHW & AACR 2000.

Illustration A: Trends in age-standardised incidence and death rates for all cancers (excluding non-melanocytic skin cancers), Australia, 1983-1998

Illustration B

Illustration B: Incident DALY Rates per 1,000 Population by Mental Disorder, Age and Sex, Victoria 1996

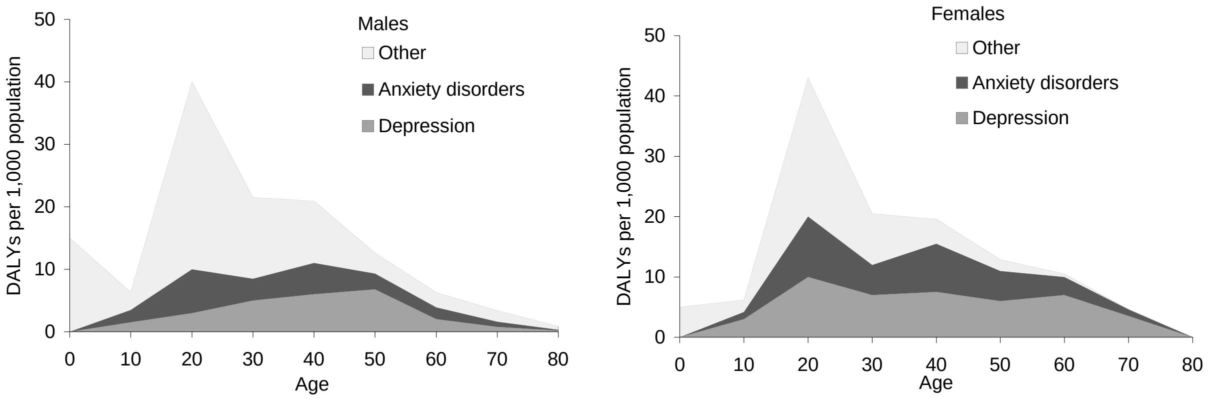

Note: The thickness of the shaded layer = DALYs per 1,000 population for that disorder

Illustration C

Illustration C: The Burden of Chronic Respiratory Disease by Condition and Sex, Victoria 1996

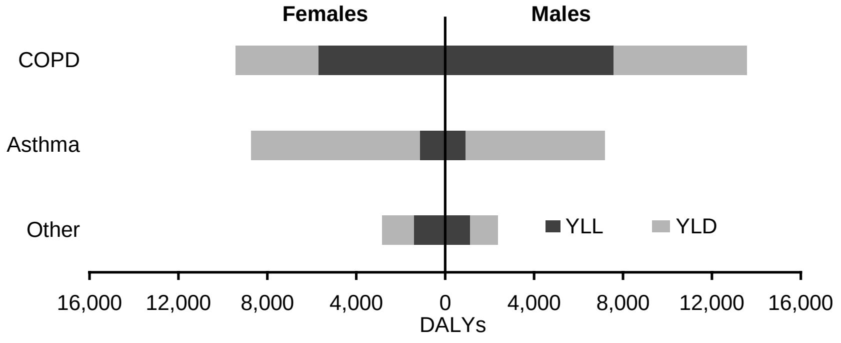

YLL = Years of Life Lost: summarises the total years of life lost from all people that die prematurely of the disease.
YLD = Years Lived with Disability: summarises the total years of healthy life lost due to disability in people living with the disease.
DALY = Disability Adjusted Life Years: total burden = the sum of YLL and YLD: lost years due to both death and disability.

Illustration D

Illustration D: Rates of YLLs by Rurality, Status and Sex

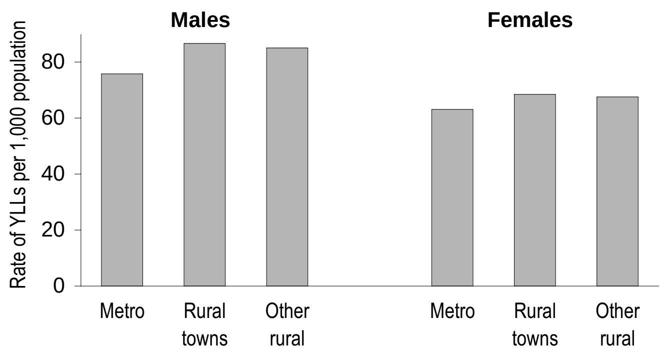

Illustration E

Illustration E: Estimated resident population by age, sex and Health Service District, 1999 and difference in age structure between Health Service District population and Queensland population

Central zone

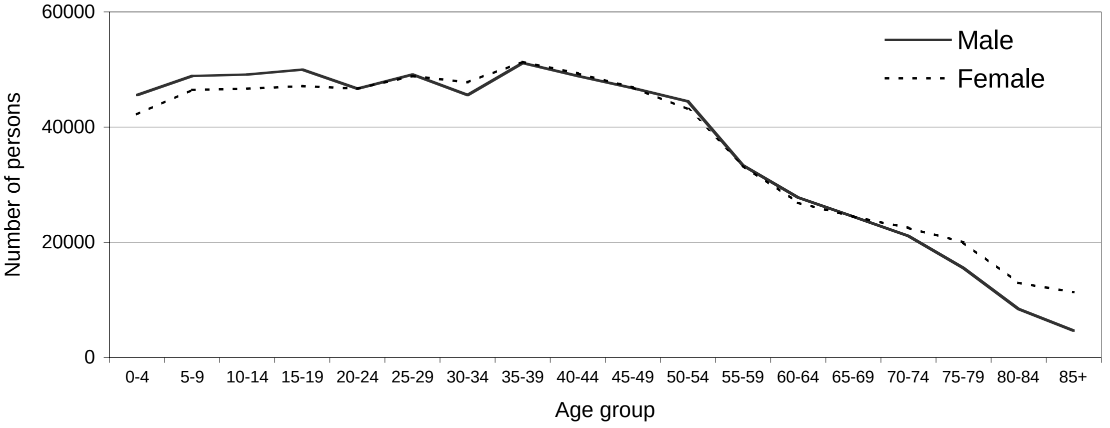

Queensland

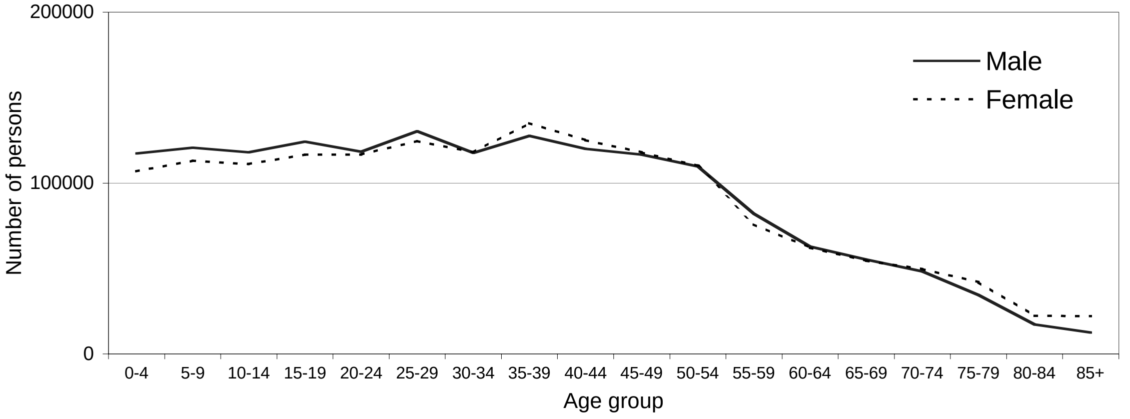

Illustration F

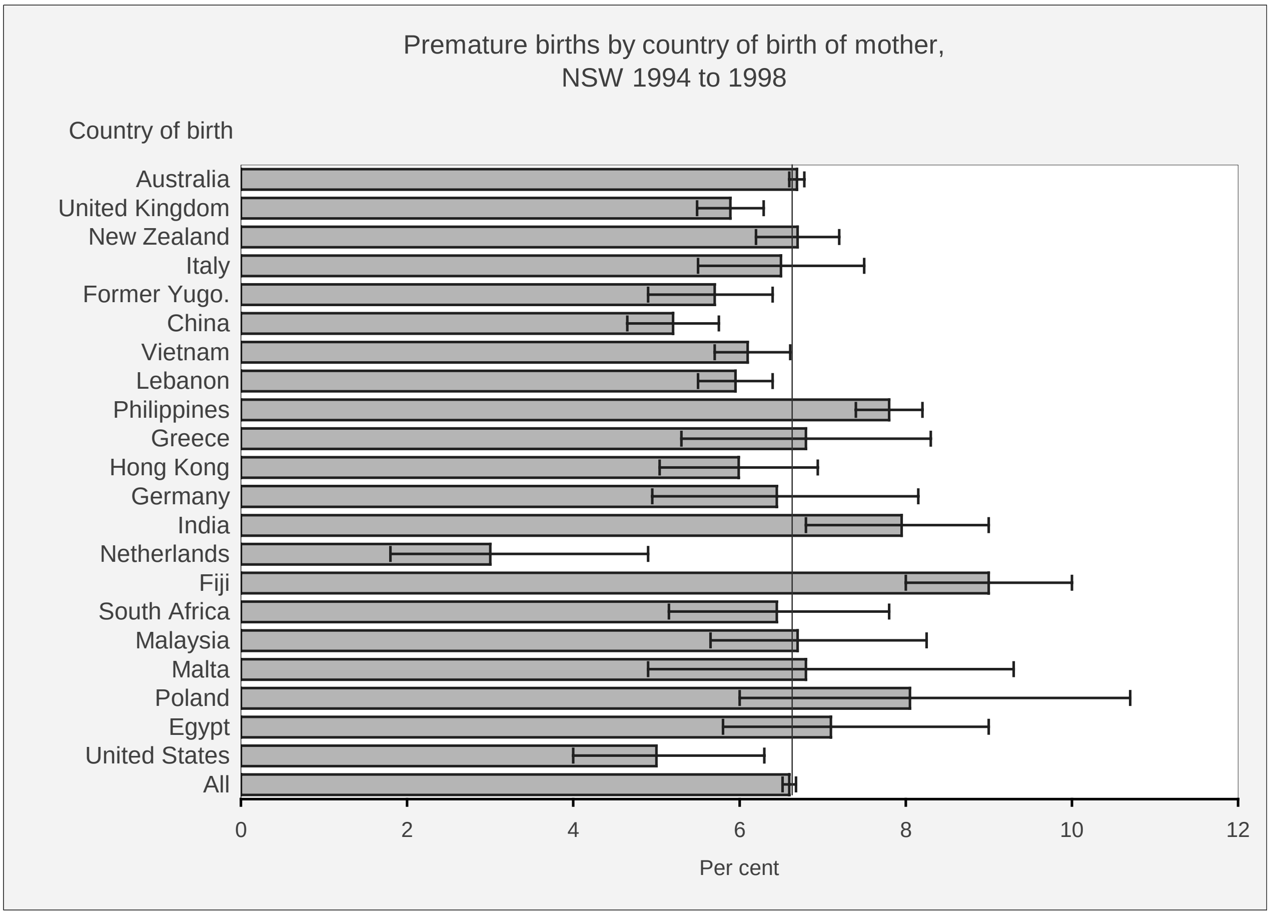

Note: Confidence intervals indicate statistical uncertainty about each value on the graph. Longer intervals mean more uncertainty. When two intervals overlap then there is more uncertainty that the two groups are really different. Births where gestational age was less that 37 weeks were classified as premature births. Infants of at least 400 grams birth weight or at least 20 weeks gestation were included.

Source: NSW Midwives Data Collection (HOIST). Epidemiology and Surveillance Branch, NSW Health Department

Illustration G

Illustration G: Between 1991 and 1995, how many times more likely to die was a Northern Territory (NT) Aboriginal person compared with all Australians for each sex and age group?

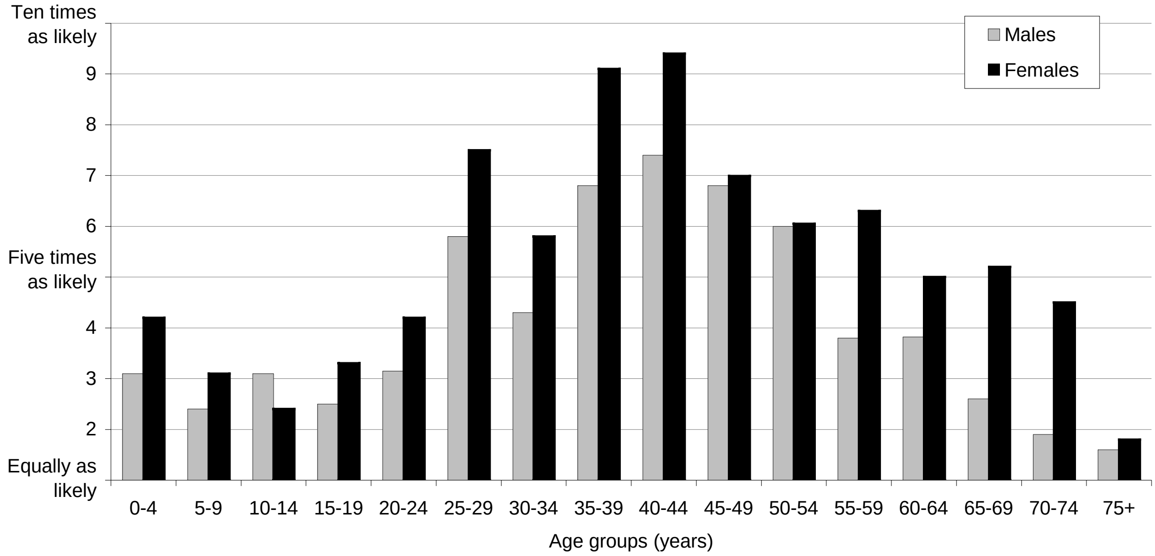

Source: Dempsey & Condon 1999

Illustration H

Illustration H: Lifetime risk for lung cancer to age 74 years

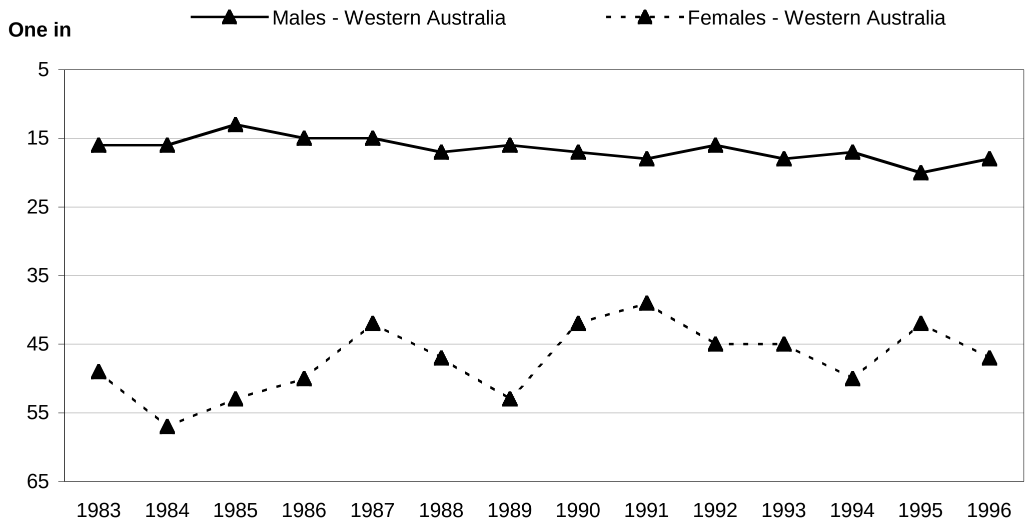

Illustration I

Illustration I: Prevalence of human immunodeficiency virus (HIV) and hepatitis C virus (HCV) infection by injecting history, clients of needle and syringe programs, NSW 1995 to 1998

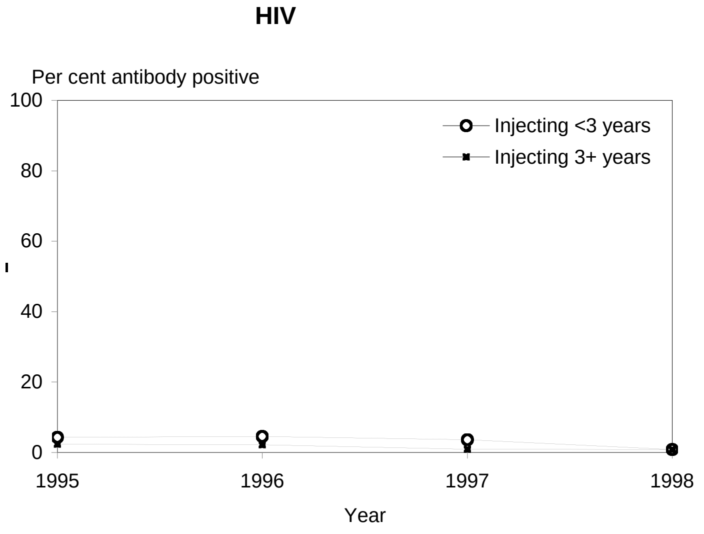

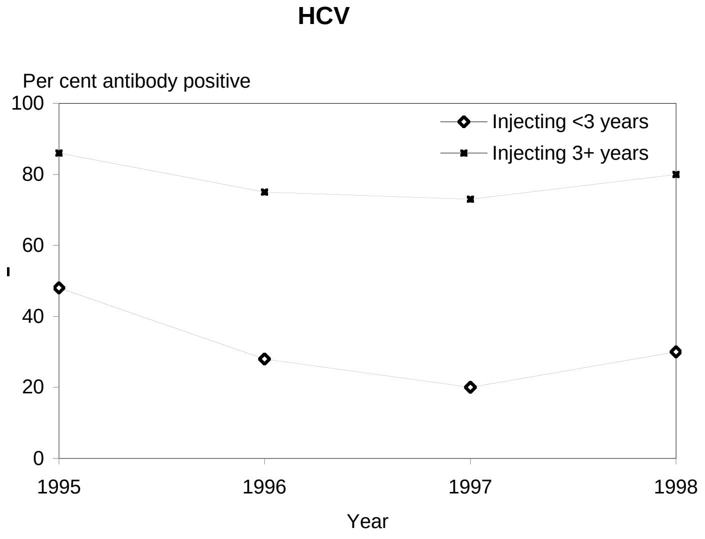

Illustration J

Illustration J: Principal causes of death, ACT, 1991-96

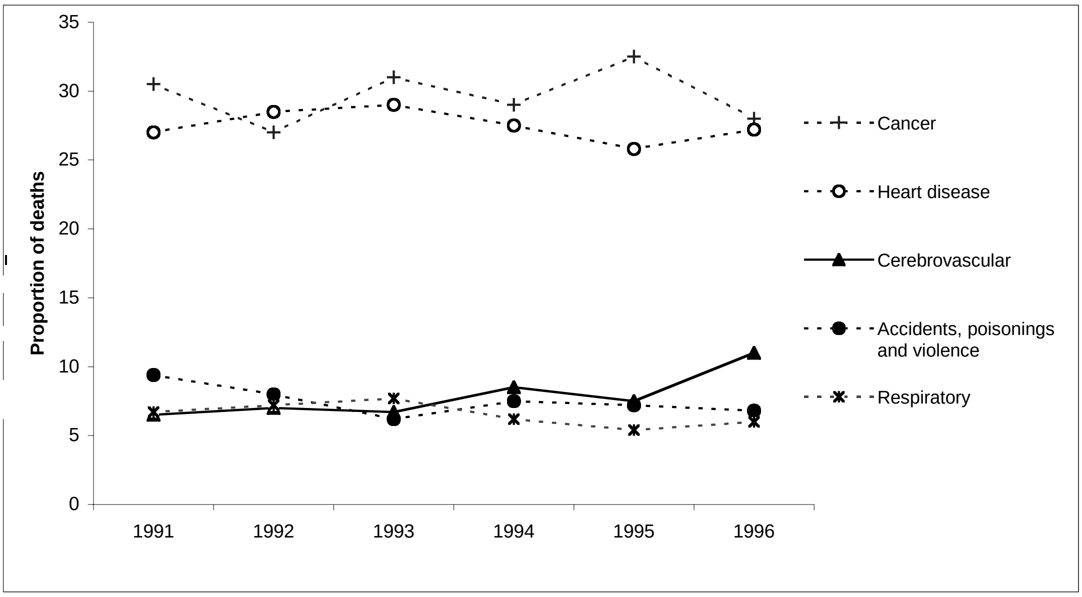

Source: *Causes of death Australia 1991-96* . ABS Catalogue No. 3303.0

Illustration K

HOSPITAL SEPARATIONS, Cause of Injury or Poisoning(a)---1998-99

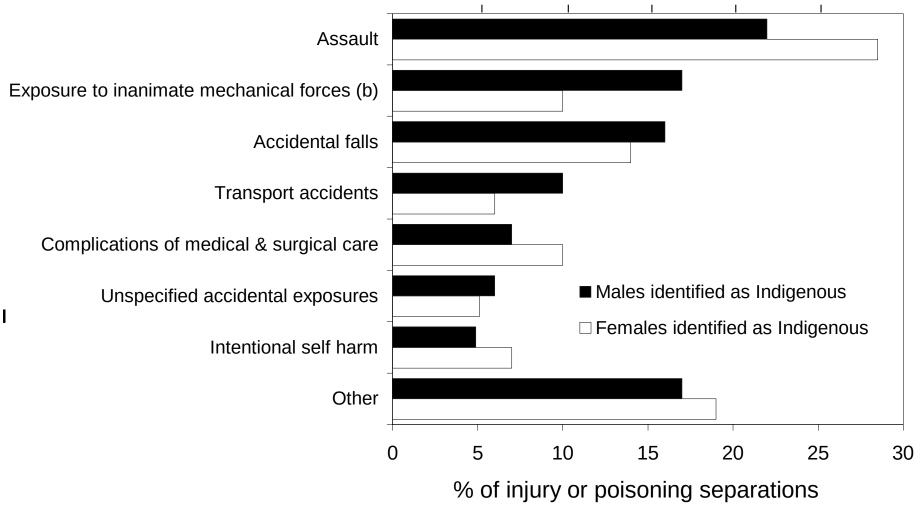

(a) Data are from public and most private hospitals. Cause of injury is based on the first reported external cause where the principal diagnosis was 'injury, poisoning and certain other consequences of external causes'.
(b) Includes injuries due to accidental contact with machinery or other objects, accidental discharge from firearms, explosions, & exposure to noise.

Source: AIHW National Hospital Morbidity Database.

Illustration L

Illustration L: Childhood cancers (0 to 14 years)

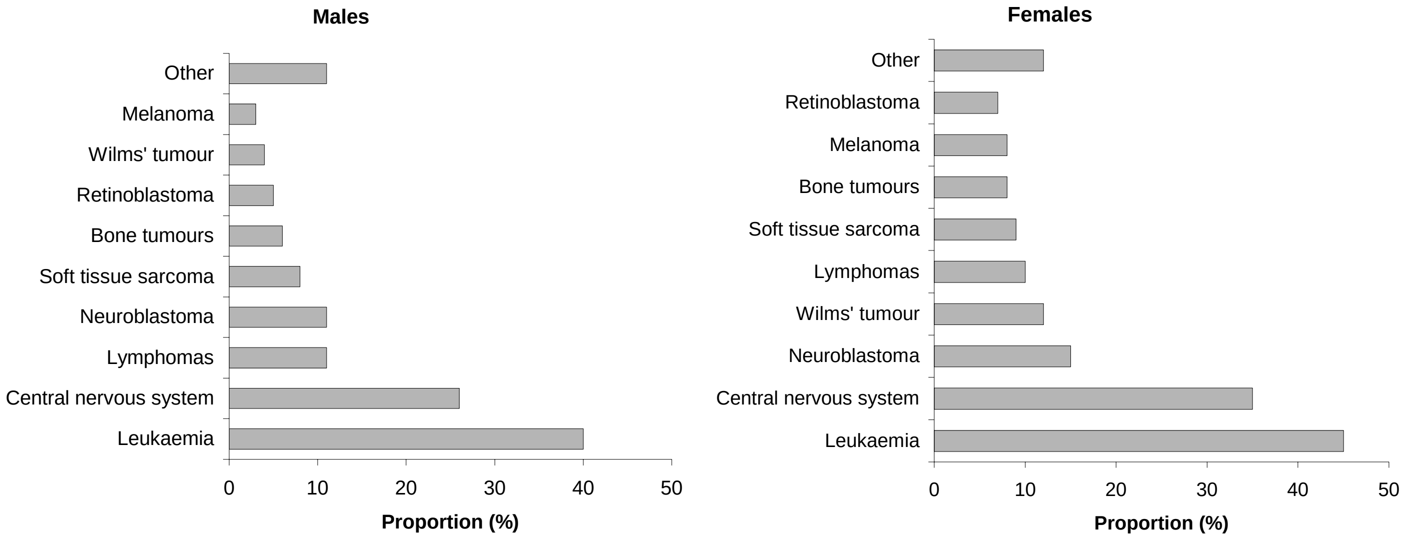

Appendix 3. Questionnaire

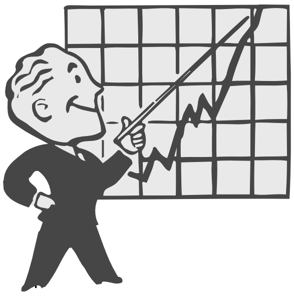

Questionnaire

Better health graphs project

Introduction

The NSW Department of Health has asked the Hunter Valley Research Foundation (HVRF) to conduct a study to determine guidelines for designing informative and useful graphs. Graph design can determine whether the reader correctly interprets the information contained in the graph. This *questionnaire* and *booklet of graphs* is one component of this study. ***Please note that this questionnaire is not a test***. Even very experienced people can have trouble understanding graphs that are not designed properly. Your answers will help us identify what aspects of graphs are hard to understand so that we can develop guidelines to improve published graphs. **We appreciate you taking the time to answer these questions even though some might seem difficult.**

Why your participation is essential

To make recommendations for the design of a good graph we need to know how people interpret different styles of graph. Even if you are not a frequent graph user, your input is valuable.

How to use this questionnaire

The questionnaire asks questions about the graphs in the *booklet of graphs*. Please write your answers in the questionnaire. The questionnaire should only take 20 minutes to complete.

Instructions

You may use any tool that you might use in real life to make interpretations. That is, any technique (ruler, pen etc.) that you already use when interpreting a graph in health publications or other media (e.g. newspapers).

Knowledge of the topic in each graph is *not* a requirement of the study. Answer the questions from the information in each graph, not your knowledge of the subject.

Returning the completed questionnaire

Please post your completed questionnaire in the self addressed, reply paid envelope to:

The Researcher
The HVRF: Graph Project
PO Box 3023
Hamilton DC NSW 2303

Any questions?

Should you have any questions regarding this research, feel free to contact Andrew Searles at the HVRF on (02) 4969 4566 (ext 525). Alternatively, call David Muscatello at NSW Health on (02) 9391 9408.

Please record your start time (below) and finish time (at the end of the questionnaire) as we would like to record how much time you required to complete the survey.

Start time: ______________________

Referring *to Illustration A* in your booklet of graphs

Approximately, how long did you take to answer the questions about illustration A ?
Minutes: _______
Seconds: _______

Qa1) Which statement best describes the incidence rate of female cancer in 1997?

Circle the number of your answer:

1 Out of every 100,000 females, there were 330 who were newly diagnosed with cancer
2 Out of every 100,000 females, there were 330 with cancer
3 For every 100,000 females, there were an additional 330 who had cancer
9 Don't know

Qa2) As the graph uses "age-standardised" data, which of the following statements is the most correct?

Circle the number of your answer:

1 Age-standardisation means differences between the rates of cancer in males and females could be due to differences in the pattern of ages in the male and female populations
2 Age-standardisation means differences between the rates of cancer in males and females are ***not*** due to differences in the pattern of ages in the male and female populations
3 Age-standardisation means that a single figure represents the rate of new cases of cancer in males and females
9 Don't know

Referring *to Illustration B* in your booklet of graphs

> Approximately, how long did you take to answer the questions about illustration B ?
> Minutes: _______
> Seconds: _______

Qb1) **What is the (approximate) difference in DALYs per 1,000 population between *female* anxiety disorders at age 20 and at age 60?**

Please write your answer here: ________________________ 9 Don't know

Qb2) **At age 30, which gender has the higher incident DALY rate for anxiety disorders, males or females?**

Circle the number of your answer:

1 Males
2 Females
9 Don't know

Qb3) **Which statement best reflects the trend in *male* depression?**

Circle the number of your answer:

1 Peaks at age 20, drops to age 30, remains stable to age 40, then declines
2 Rises to a peak at age 50, then declines
3 Fluctuates throughout the age groups
9 Don't know

Qb4) **Compared with females, males are less likely to develop mental disorders at 60 or more years of age?**

Circle the number of your answer:

1 True
2 False
9 Don't know

Referring *to Illustration C* in your booklet of graphs

Approximately, how long did you take to answer the questions about illustration C ?
Minutes: _______
Seconds: _______

Qc1) For male COPD (chronic obstructive pulmonary disease), which is the larger value, YLL or YLD?

Circle the number of your answer:

1 YLL
2 YLD
9 Don't know

Qc2) For males, which respiratory disease caused the highest <u>disability</u> burden?

Circle the number of your answer:

1 Other respiratory
2 Asthma
3 COPD (chronic obstructive pulmonary disease)
4 Cannot be answered from the graph
9 Don't know

Qc3) For asthma, do males or females have the greatest burden from deaths (YLL)?

Circle the number of your answer:

1 Males
2 Females
9 Don't know

Qc4) Which disease has the greatest overall burden (DALYs) for females?

Circle the number of your answer:

1 COPD (chronic obstructive pulmonary disease)
2 Asthma
3 Other respiratory
9 Don't know

Referring *to Illustration D* in your booklet of graphs

Qd1) What was the approximate total rate of YLLs for females in rural towns?

Circle the number of your answer:

1 60
2 65
3 80
9 Don't know

Approximately, how long did you take to answer the questions about illustration D ?
Minutes: _______
Seconds: _______

Qd2) Correct or incorrect?

Circle the number of your answer:

Statement A: Rural areas had higher rates of YLLs compared with metropolitan areas?

1 Correct
2 Incorrect
9 Don't know

Statement B: Males had lower rates of YLLs than females, regardless of where they lived

1 Correct
2 Incorrect
9 Don't know

Referring *to Illustration E* in your booklet of graphs

Approximately, how long did you take to answer the questions about illustration E?
Minutes: _______
Seconds: _______

Qe1) In Queensland, males aged 19 or less outnumber females aged 19 or less.

Circle the number of your answer:

1 True
2 False
9 Don't know

Qe2) Which is the most accurate statement for this illustration?

Circle the number of your answer:

1 The two graphs show that Central Zone has more people than Queensland
2 The two graphs show that Central Zone has less people than Queensland
3 Cannot answer from the graph
9 Don't know

Qe3) Which is the most accurate statement for this illustration?

Circle the number of your answer:

1 Both Central Zone and Queensland have more younger people (aged 19 or less) than older people (aged 60+)
2 Both Central Zone and Queensland have more older people (aged 60+) than younger people (aged 19 or less)
3 Cannot answer from the graph
9 Don't know

Referring *to Illustration F* in your booklet of graphs

Approximately, how long did you take to answer the questions about illustration F ?
Minutes: _______
Seconds: _______

Qf1) **Can we be certain that mothers born in Greece and those born in the Philippines really differed from each other in their chance of having a premature birth?**

Circle the number of your answer:

1 Yes
2 No
9 Don't know

Qf2) **Comparing mothers born in the Philippines with those born in Lebanon:**

Circle the number of your answer:

1 Mothers born in the Philippines had a higher proportion of premature births
2 Mothers born in the Philippines had a lower proportion of premature births
9 Don't know

Qf3) **Mothers born in Lebanon had a lower proportion of premature births than mothers born in Australia?**

1 True
2 False
9 Don't know

Referring *to Illustration G* in your booklet of graphs

Approximately, how long did you take to answer the questions about illustration G?
Minutes: _______
Seconds: _______

Qg1) This graphs shows that, compared with most Australians ….

Circle the number of your answer:

1 NT Aboriginal people have a higher risk of death
2 NT Aboriginal people have a similar risk of death
3 NT Aboriginal people have a lower risk of death
9 Don't know

Qg2) For the age group 70-74 how many times greater is the risk of a NT Aboriginal women dying compared with all Australian women in the same age group?

Circle the number of your answer:

1 4.0
2 4.5
3 5.0
9 Don't know

Qg3) For the age group 45-49 the approximate value for females is 7. Which of the following best describes the meaning of this result for people aged 45-49:

Circle the number of your answer:

1 Compared with Aboriginal males, Aboriginal females are seven times more likely to die
2 The risk of a Northern Territory Aboriginal female dying is seven times as high as an Australian female overall
3 Seven Northern Territory Aboriginal males die for every 1 Aboriginal female
9 Don't know

Referring *to Illustration H* in your booklet of graphs

Approximately, how long did you take to answer the questions about illustration H?
Minutes: _______
Seconds: _______

Qh1) In 1996, would a male or a female have been more likely to develop lung cancer in Western Australia?

Circle the number of your answer:

1 A female
2 A male
9 Don't know

Qh2) What is the direction of male lifetime risk for lung cancer?

Circle the number of your answer:

1 Slightly increasing risk
2 Slightly decreasing risk
3 Steady (no trend)
9 Don't know

Qh3) In 1993, what was the lifetime risk for females?

Please write your answer here: One in ________________________ 9 Don't know

Referring *to Illustration I* in your booklet of graphs

Approximately, how long did you take to answer the questions about illustration I?
Minutes: _______
Seconds: _______

Qi1) In 1997, approximately what proportion of clients who had been injecting for less than 3 years had HCV infection?

Please write your answer here: ________________________ 9 Don't know

Qi2) Which group had the lower prevalence of HCV infection between 1995 and 1998?

Circle the number of your answer:

1 Those injecting for 3 or more years
2 Those injecting less than 3 years
3 Both have the same prevalence
9 Don't know

Qi3) Which infection was more prevalent among injecting drug users in 1996?

Circle the number of your answer:

1 HIV
2 HCV
3 HIV and HCV were about the same
9 Don't know

Qi4) In 1997, the gap in prevalence between short and long term injectors was approximately the same for HIV and HCV?

Circle the number of your answer:

1 True
2 False
9 Don't know

Referring *to Illustration J* in your booklet of graphs

Approximately, how long did you take to answer the questions about illustration J?
Minutes: _______
Seconds: _______

Qj1) Approximately what proportion of deaths were due to cancer in 1996?

Please write your answer here: ________________________ 9 Don't know

Qj2) In 1995, the lowest proportion of deaths was associated with ….

Circle the number of your answer:

1 Cancer
2 Heart disease
3 Accidents, poisonings and violence
4 Respiratory
9 Don't know

Qj3) Which cause of death shows the most increasing trend between 1991 and 1996?

Circle the number of your answer:

1 Cancer
2 Heart disease
3 Cerebrovascular
4 Respiratory
9 Don't know

Referring *to Illustration K* in your booklet of graphs

Approximately, how long did you take to answer the questions about illustration K?
Minutes: _______
Seconds: _______

Qk1) Does intentional self harm account for a greater proportion of hospital separations for indigenous males or indigenous females?

Circle the number of your answer:

1 Males
2 Females
3 Both are the same
9 Don't know

Qk2) What is the most common cause of hospital separations for injuries in indigenous males?

Circle the number of your answer:

1 Transport accidents
2 Complications of medical and surgical care
3 Assault
9 Don't know

Referring *to Illustration L* in your booklet of graphs

Approximately, how long did you take to answer the questions about illustration L?
Minutes: _______
Seconds: _______

QL1) What is the most common childhood cancer for <u>males</u>?

Please write your answer here: ________________________ 9 Don't know

QL2) For females, are there more neuroblastomas or central nervous system cancers?

Circle the number of your answer:

1 Central nervous system
2 Neuroblastomas
3 Both are the same
9 Don't know

QL3) Do males or females have a greater proportion of central nervous system cancers?

Circle the number of your answer:

1 Males
2 Females
3 Both are the same
9 Don't know

QL4) What is the least common cause of cancer in females?

Circle the number of your answer:

1 Melanoma
2 Retinoblastoma
3 Bone tumours
9 Don't know

QL5) Approximately what proportion of childhood cancers for girls does melanoma account for?

Please write your answer here: ________________________ 9 Don't know

These questions will help ensure our sample included a range of people

DEM1) In what language would you have felt most comfortable completing this questionnaire?

Circle the number of your answer:

1 English

2 Other *Please identify your preferred language:* ________________________

DEM2) What is the highest level of education you have completed?

Circle the number of your answer:

1 Never attended school

2 Primary school only

3 Secondary school (Up to year 12 / 6th form / HSC / Leaving Certificate)

4 TAFE or equivalent technical qualification

5 University or CAE

6 Postgraduate studies

8 Other *Please identify:* ________________________

DEM3) How frequently do you use graphs in your daily activities?
(This includes graphs that you might interpret or create yourself. They might be for work or non-work activities such as reading a newspaper or for your studies).

Circle the number of your answer:

1 Never

2 Rarely (i.e. less than a few times a year)

3 Occasionally (i.e. a few times a year to less than once a month)

4 Often (i.e. at least once a month)

DEM4) How would you rate your *visual* ability to see the detail in the graphs in this study?
(This refers to your ability to see the labels and diagrammatic detail either unaided, or if you have corrected vision, with eye glasses, contact lenses or other aides).

Circle the number of your answer:

1 Good (could read all labels and notes on the sample graphs – even when the font size was small)

2 Average (could read labels and notes on the sample graphs – with slight difficulty)

3 Poor (had difficulty reading labels and notes on the sample graphs)

DEM5) What is your age category?

1 Under 24

2 24 to 34

3 35 to 44

4 45 to 54

5 55 to 64

6 65 and over

Continued over the page

These questions will help ensure our sample included a range of people

DEM6) And your gender?

Circle the number of your answer:

1 Male

2 Female

DEM7) How would you describe your current work position?

Please write your occupation in the space below

____

DEM8) If you have completed the questionnaire in one sitting (and provided a start time on page 2), please answer 6a. If the questionnaire was completed over multiple sittings please answer 6b.

6a) Finish time: ______________________

6b) Approximately how much time in total did you need to complete this questionnaire?

Please write the length of time in minutes here: ________________________

Thank you for your help!

Please return your completed questionnaire in the reply paid envelope.

SHPN (HSP) 060048
